# Landscape of transcriptomic interactions between breast cancer and its microenvironment

**DOI:** 10.1038/s41467-019-10929-z

**Published:** 2019-07-15

**Authors:** Natalie S. Fox, Syed Haider, Adrian L. Harris, Paul C. Boutros

**Affiliations:** 1 Ontario Institute for Cancer Research, Toronto, ON M5G 0A3 Canada; 2 Department of Medical Biophysics, University of Toronto, Toronto, ON M5G 1L7 Canada; 3 Department of Oncology, Weatherall Institute of Molecular Medicine, University of Oxford, Oxford, OX3 9DS UK; 4 The Breast Cancer Now Toby Robins Research Centre, The Institute of Cancer Research, London, SW7 3RP UK; 5 Department of Pharmacology and Toxicology, University of Toronto, Toronto, ON M5S 1A8 Canada; 6 Department of Human Genetics, University of California, Los Angeles, CA 90095 USA; 7 Department of Urology, University of California, Los Angeles, CA 90024 USA; 8 Broad Stem Cell Research Center, University of California, Los Angeles, CA 90095 USA; 9 Institute for Precision Health, University of California, Los Angeles, CA 90095 USA; 10 Jonsson Comprehensive Cancer Center, University of California, Los Angeles, CA 90024 USA

**Keywords:** Cancer, Breast cancer, Cancer microenvironment, Computational biology and bioinformatics, Data acquisition

## Abstract

Solid tumours comprise mixtures of tumour cells (TCs) and tumour-adjacent cells (TACs), and the intricate interconnections between these diverse populations shape the tumour’s microenvironment. Despite this complexity, clinical genomic profiling is typically performed from bulk samples, without distinguishing TCs from TACs. To better understand TC–TAC interactions, we computationally distinguish their transcriptomes in 1780 primary breast tumours. We show that TC and TAC mRNA abundances are divergently associated with clinical phenotypes, including tumour subtypes and patient survival. These differences reflect distinct responses of TCs and TACs to specific somatic driver mutations, particularly *TP53*. These data further elucidate how the molecular interplay between breast tumours and their microenvironment drives aggressive tumour phenotypes.

## Abstract (web summary)

The transcriptomic profile of tumour-adjacent cells provides important information about tumour context but its clinical utility is unclear. Here, in breast cancer, Fox et al. show that the mRNA abundances of tumour and tumour-adjacent cells hold prognostic information.

## Introduction

Solid tumours are not homogeneous masses of cancer cells. Rather, tumour cells (TCs) are intermingled with adjacent ones, including fibroblasts, immune and inflammatory cells, fat cells and endothelial cells. These cells are not cancerous themselves, but the complex interplay between TCs and tumour-adjacent cells (TACs) can create a microenvironment favourable for tumour progression^1^. Indeed the failure to recapitulate this microenvironment and its interplay is one reason cancer cell lines, organoids and xenografts can be difficult to generate and are imperfect avatars for in vivo tumours^2–4^.

In breast cancer, mRNA abundance has clear clinical utility, both to define subtypes with predictive value for treatment response, and to model the aggressiveness of a tumour by predicting patient survival^5,6^. These profiles appear to have been driven largely by TC information, although we continue to gain insights into the roles TACs play in cancer initiation, progression and response to treatment. As a result, it remains unclear how TAC and TC transcriptomes inter-relate. Some groups have used micro-dissection to obtain TAC or stromal mRNA abundance profiles, and developed candidate biomarkers from these^7–10^. It is attractive to imagine transcriptomic biomarkers that integrate TC and TAC information to better understand tumour behaviour in the context of its cellular microenvironment.

Although most large-scale genomic studies aim to investigate pure TC populations, the practicalities of tumour sampling results in bulk mixtures of TCs and TACs^10,11^. Sample purity can be improved using techniques like laser capture micro-dissection^12^, but these can be costly, involve handling that increases the potential for errors and are infeasible with some samples because of unintentional drying and other artefacts^13^. Single-cell sequencing may eventually resolve these issues, but is currently neither accurate enough for clinical usage nor technically feasible on routine clinical samples, and remains relatively slow and costly^14^.

As an alternative approach, in silico techniques for purifying cancer mRNA abundance profiles, called deconvolution algorithms, have been developed^11,15–18^. Deconvolution algorithms purify TC mRNA abundance from confounding TAC mRNA abundance, thereby reducing inter-sample heterogeneity and increasing statistical power. Further, their negligible cost and speed makes them attractive for clinical studies. As a result, these have been adopted in some recent large-scale discovery studies^19,20^. Indeed there is evidence that they improve the accuracy of clinical prediction tools^17,21^. But overall the clinical utility of TAC mRNA profiles, and their relationship with the key somatic driver mutational events occurring in tumour genomes, remains largely uncharacterised.

To fill this gap, we evaluate the landscape of TC and TAC transcriptomes in a cohort of 1780 primary breast tumours. We comprehensively evaluate the synergy between TC and TAC mRNA abundance in clinical decision-making, and quantify the value of prognostic TAC-derived biomarkers. These data quantify how specific somatic driver mutations dysregulate their tumour microenvironments, and how TCs and TACs respond differently to a specific somatic driver event. Taken together, these results paint a landscape of genome–TC–TAC interactions that demonstrate an important approach for improving our understanding of the evolution and aggression of primary tumours.

## Results

### Deconvolving TC and TAC mRNA abundance

**Fig. 1:**
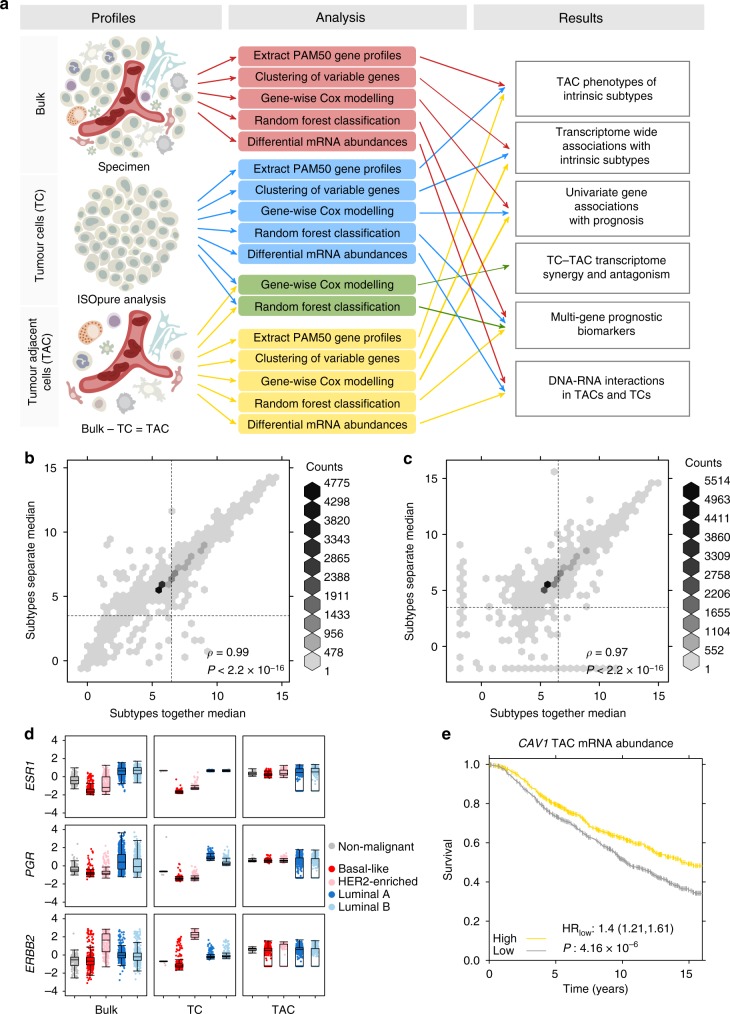
Overview of profile comparison analyses. **a** Three different mRNA profile types (left: bulk, TC and TAC) were used in analysis (middle) and reported in the results (section specified on right). For the boxes and arrows, red represents bulk mRNA abundance, blue represents TC mRNA abundance and yellow represents TAC mRNA abundance. Green represents both TC and TAC together. **b** The change in median TC mRNA abundance per gene when deconvolution was performed with subtypes together or with subtypes separated. **c** The change in median TAC mRNA abundance per gene when deconvolution was performed with subtypes together or with subtypes separated. **d** The mRNA abundance scaled over all patients for each subtype and each of bulk, TC and TAC mRNA abundances for the receptor genes: *ESR1*, *PGR* and *ERBB2*. **e** *CAV1* TAC mRNA abundance was median dichotomised and tested for prognosis using univariate Cox proportional hazards modelling

To obtain TC- and TAC- specific mRNA abundance from bulk cancer samples, we augmented the deconvolution algorithm called ISOpure, which applies a non-cancerous reference panel to deconvolve different cell populations^17^. Previously published ISOpure deconvolves TC mRNA abundance profiles and estimates the sample tumour purity. We extended ISOpure to extract and quantify TAC mRNA abundance profiles as well as TC mRNA abundance profiles, where TAC mRNA abundance profiles comprise the mixture of non-cancerous cell populations located proximally to the tumour. TAC deconvolution capability is available in the latest version of ISOpureR on CRAN. We applied this updated algorithm to 1780 primary breast tumours, one per patient^22^, creating a landscape of paired TC and TAC transcriptomes. These data were then used to evaluate both single gene and multi-gene prognostic associations, TC–TAC biomarker synergy, microenvironmental hallmarks of breast cancer subtypes and the microenvironmental consequences of specific driver mutations (Fig. 1a). We used the TCGA cohort of 1014 primary breast tumours with RNA-Seq mRNA abundance profiles for validation of these analyses. The median mRNA abundance per gene was correlated between data sets for bulk, TC and TAC mRNA abundance profiles (Supplementary Fig. 1).

ISOpure is a two-step process. In the first step, ISOpure uses the average mRNA abundance profile of the cohort to estimate the proportions of TCs in each bulk sample. In the second step, individual TC mRNA abundance profiles are created using the estimated purities from step one. To this we added a third step, which creates TAC mRNA abundance profiles from the purity estimates and bulk and TC mRNA abundance profiles. As breast cancer is a heterogeneous disease with subtypes that have unique molecular profiles, clinical outcomes and treatment responses, there may be subtype-associated bias for ISOpure deconvolution. To avoid this, we performed TC–TAC devolution separately for each intrinsic breast cancer subtype (PAM50), thereby using a unique average mRNA abundance profile per subtype of the disease^23^ (Supplementary Fig. 2A). We benchmarked the variation between global analysis and subgroup-based analysis (Supplementary Fig. 2B). The average mRNA abundance for each gene was highly concordant between the two approaches (*ρ*_bulk_ = 0.99, *ρ*_TC_ = 0.97; Fig. 1b, c). The PAM50 classifications from these TC mRNA abundance profiles agree with the original Curtis et al.^22^ classifications for ~ 75% of patients, within variation reported between PAM50 and immunohistochemical classification^24^ (Supplementary Table 1). Thus, the mRNA abundance profiles only vary slightly dependent on the cohort used for running ISOpure and, for all analysis, we used the mRNA abundance profiles deconvolved per subtype.

To assess the effectiveness of our deconvolution, we first examined the three receptor genes associated with molecular subtypes of breast cancer: the oestrogen receptor (*ESR1*), progesterone receptor (*PGR*), and human epidermal growth factor receptor 2 (*ERBB2*). Patients with basal-like breast cancer, including most triple receptor-negative tumours, are well-known to have lower tumour mRNA abundances of all three receptors. By contrast, patients with HER2-enriched breast cancer have elevated *ERBB2* and those with luminal breast cancers have elevated *ESR1* and *PGR*. As expected, these trends were all confirmed in bulk transcriptomes, clarified in TC transcriptomes and absent in TAC transcriptomes (Fig. 1d). As a further control we evaluated stromal caveolin-1 (*CAV1*), which is a prognostic biomarker in breast cancer^25,26^. We confirmed this association using TAC mRNA abundance (Fig. 1e). We further verified the TAC-specific of *CAV1* expression: only 1/1780 patients exhibited any TC expression of *CAV1*, as expected.

### The landscape of breast cancer purity

Across our cohort, we identified a strikingly broad range of tumour purity, ranging from 6 to 88%. The median tumour had 59% of the bulk sample estimated to be TCs, and there was good concordance between ISOpure and pathology estimates of tumour cellularity (Supplementary Fig. 3A). Some individual tumours showed large divergence between pathology and molecular estimates: these may in part reflect the challenges faced by visual area-based assessment in reflecting the differing volumes and RNA concentrations in TCs and TACs^17,27^. ISOpure tended to estimate higher purity than pathologists in tumours with higher grades and in basal-like tumours. By contrast, pathologists tended to estimate higher purity in older patients, in normal-like breast tumours and in ER + tumours (Supplementary Fig. 3C, Supplementary Table 2). ISOpure purity estimates varied modestly between subtypes, with basal-like and luminal B samples having higher purity (*p* <2.2 × 10^−16^, one-way analysis of variance (ANOVA); Supplementary Fig. 3B; Supplementary Table 3; Supplementary Data 1). We tested purified non-malignant adjacent normals as a negative control and 73/75 (97%) had an estimated purity below 0.1. By contrast, only two (0.1%) of the breast cancer samples had estimated purity below 0.1 (Supplementary Fig. 3B).

### TAC phenotypes of intrinsic subtypes

Breast cancer subtypes are an integral part of breast cancer research and clinical management. To ascertain the TAC phenotypes of the intrinsic breast cancer subtypes, we started with the widely-used PAM50 subtyping scheme^23^. We grouped patients based on similar mRNA abundance for the PAM50 genes to evaluate whether these genes which reflect subtype differences in TCs also distinguish the subtypes in TACs. The breast cancer subtypes were well-segregated by bulk mRNA abundances of PAM50 genes and the TAC mRNA abundance of PAM50 genes separated the luminal subtypes from the others (Supplementary Fig. 4A–C), verifying that the transcriptome of the tumour microenvironment varies with clinical subtype.

**Fig. 2:**
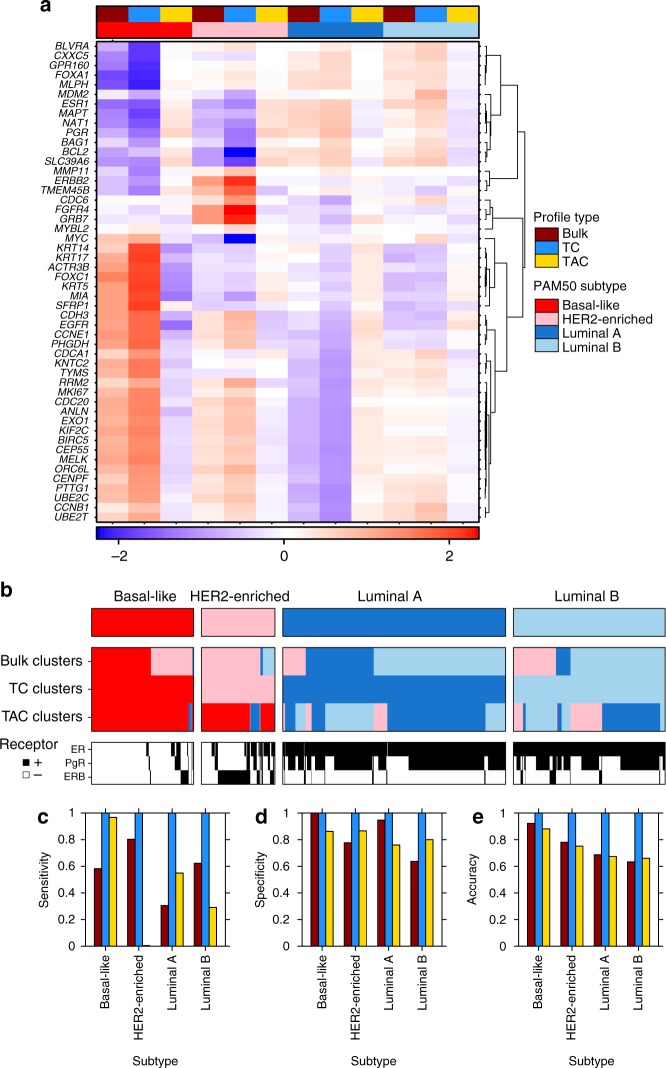
PAM50 associations. For genes in the PAM50 biomarker, columns compare the average mRNA abundance for patients of each subtype. The mRNA abundance for each gene are scaled across all patient profiles for each profile type **a**. Concordance between PAM50 subtypes and patients clusters based on highly variant genes (genes with standard deviation > 1.0 in at least one mRNA abundance type) are shown in **b**. PAM50 classifications in the top bar are from the METABRIC paper. Receptor status of ERB, PgR and ER are also shown for each patient. Statistics describing classification alignment are shown in **c**, **d** and **e**

To assess whether the individual PAM50 genes had increased or decreased relative mRNA, we compared their mean mRNA abundances between subtypes (Fig. 2a). We confirmed that the PAM50 genes cluster similarly to canonical gene-groupings^23^. Mean bulk and TC mRNA abundances were similar for each subtype and generally the TC mRNA abundance was an intensified version of the bulk mRNA abundance, in particular for patients with basal-like, HER2-enriched and luminal A breast cancers. However for the PAM50 genes, TAC mRNA abundance did not show similar relative mRNA abundance patterns between subtypes compared to TC and bulk mRNA abundance. *BCL2*, a well-established prognostic biomarker in early breast cancer^28^, showed reduced mRNA abundance in HER2-enriched TCs relative to other subtypes, but slightly elevated mRNA abundance in HER2-enriched TACs (*p* < 2.2 × 10^−16^, one-way ANOVA; Supplementary Fig. 4D). For each individual PAM50 gene, TC mRNA abundances tended to recapitulate bulk mRNA abundance but TAC mRNA abundance differed per subtype. These mRNA differences between bulk and TC profiles lead to 88% agreement for classifications based on PAM50 methodology. PAM50 subtypes based on TAC mRNA abundance agreed with the other classifications for only 19% of patients. Overall, the PAM50 genes manifest differently in TC and TAC mRNA abundance with TC mRNA abundance for PAM50 genes an excellent surrogate for the intrinsic subtypes of breast cancer and PAM50 TAC mRNA less associated with subtypes.

The intrinsic subtypes of breast cancer should be reflected across genes other than the PAM50 genes and there could be a better surrogate for the subtypes using TAC mRNA. There are hundreds of gene whose TAC mRNA abundance were associated with the intrinsic breast cancer subtypes (Supplementary Table 4). For example, *KRT13* had higher TAC mRNA abundance in HER2-enriched breast tumours (Supplementary Fig. 5A). Correlation of TC and TAC mRNA abundances for the same gene varied by subtype illustrating that the relationships between TCs and TACs can be subtype specific (Supplementary Fig. 5B–F). To further assess how TAC profiles correspond with the intrinsic TC subtypes of breast cancer, we clustered the mRNA abundance from the most variable genes into four subtypes. The percentage of patients correctly classified ranged from 51% (bulk) to 48% (TAC) to 100% (TC). TAC mRNA abundance is clearly defining different subtypes than TC mRNA abundance (Fig. 2b–e). Again, TC mRNA abundance clusters had complete overlap with the intrinsic subtypes indicating that our computational separation procedure kept mRNA profiles distinct (Supplementary Fig. 5G). Of the four TAC clusters, aggressive tumours (HER2-enriched and basal-like) were a single cluster while the other three clusters were comprised of mixtures of luminal A and B tumours (Supplementary Fig. 5G). Genes that had differential mRNA abundance between the cluster of patients with aggressive tumours and the other three clusters were enriched for genes involved in the immune system (*q* =  2.46 × 10^−5^, hypergeometric test), MicroRNAs in cancer (*q* = 3.29 × 10^−5^, hypergeometric test) and cell–cell signalling (*q* = 2.36 × 10^−5^, hypergeometric test; Supplementary Data 2). Altogether PAM50 subtypes are associated with specific TAC mRNA profiles, many other genes outside the PAM50 scheme are important to explain TAC mRNA abundance breast cancer subtypes.

### Univariate gene associations with prognosis

**Fig. 3:**
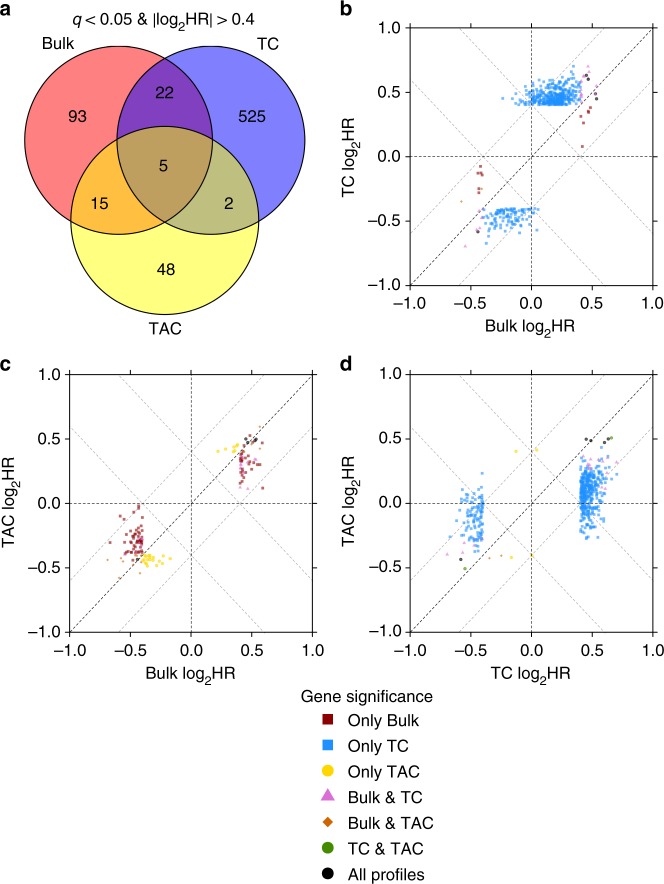
Comparison of univariate prognostic results between profiles. The number of genes that are prognostic using a threshold of Wald *q*-value < 0.05 and |log_2_HR| > 0.4 **a**. The relationship between corresponding hazard ratios is shown in **b**–**d**. Points are coloured for which profiles they are significant in (colours follow similar colour mixing to the Venn diagram). **b**–**d** Dashed black lines show where the genes are not prognostic (*y* = 0, *x* = 0) and where genes have equal prognosis in each profiles (*y* = *x*). Grey dashed lines indicate a difference of 0.4 between the log_2_HR from each profile (*y* = *x*−0.4, *y* = *x* + 0.4, *y* = −*x*−0.4, *y* = −*x* + 0.4)

After showing TAC mRNA associates with the clinical phenotypes of breast cancer subtypes, we next assessed whether TC and TAC mRNA abundances were differentially associated with overall patient survival (OS). Using univariate Cox proportional hazards modelling, we evaluated the association of each gene’s TC and TAC mRNA abundances with OS. Relative to bulk (unpurified) samples, TCs consistently showed a large increase in number of genes associated with OS, in addition to a smaller of number of genes whose TAC mRNA abundances were associated with OS (Supplementary Fig. 6A). At a threshold of *q* < 0.05 (Wald test), purification more than quadrupled the number of genes associated with OS from 135 in bulk samples to 617 from TAC and TC mRNA profiles. Unexpectedly, there were drastically more genes with TC mRNA abundances associated with OS than TAC mRNA abundance. Low numbers of prognostic TAC mRNA abundances was likely a consequence of TAC mRNA abundance being a mixed population of cells and not that the microenvironment does not have important prognostic mRNAs. Most genes (94%) were prognostic in a single profile type (Fig. 3a), independent of the statistical threshold used (Supplementary Fig. 6B). Genes showing TC-survival associations were enriched for methylation, cell cycle and DNA repair pathways (Supplementary Data 2), whereas TAC-survival associations were not. Thus TCs and TACs transcriptomes represent distinct biological processes that have distinct associations with patient outcome.

We assessed whether bulk mRNA abundance prognostic associations were retained in TC and TAC mRNA. As expected, gene-wise hazard ratios were well-correlated between bulk and TC mRNA abundances (*ρ* = 0.55, *p* < 2.2 × 10^−16^, Spearman’s correlation; Supplementary Fig. 6C), as were those between bulk and TAC mRNA abundances (*ρ* = 0.62, *p* < 2.2 × 10^−16^, Spearman’s correlation; Supplementary Fig. 6D). Association with poor or good outcome was also consistent between profiles. Bulk and TC mRNA abundance shared 19 genes associated with good OS (i.e., HR > 1) and eight associated with poor OS (i.e., HR < 1; Fig. 3b). Bulk and TAC mRNA abundance shared 8 and 12 genes whose high mRNA abundance associated with good and poor OS respectively (Fig. 3c). There were 93 genes showing bulk-survival associations but no association with TC or TAC mRNA abundance. These were enriched for six biological processes including negative regulation of gene expression and translation (Supplementary Data 2). To complete the characterisation of the three profile types, we assessed whether TC and TAC were recapitulating the same biological signal (Supplementary Fig. 6E). We found that seven genes had both TAC and TC mRNA abundance associated with OS (Fig. 3d; Supplementary Data 3). These included *LARP1* (HR_bulk_ = 1.4, HR_TC_ = 1.5, HR_TAC_ = 1.4) and *AK3* (HR_bulk_ = 0.74, HR_TC_ = 0.67, HR_TAC_ = 0.65). Therefore TC and TAC mRNA capture gene-survival associations not seen in bulk mRNA-profiling studies.

Some of the genes significant in the entire cohort showed specificity to the intrinsic subtypes of breast cancer (Supplementary Data 3, Supplementary Fig. 7). *LARP1* and *AK3* are associated with OS in luminal A and luminal B breast cancers (Supplementary Data 3). Taken together, TC and TAC mRNA abundance retained most gene associations with OS identified using bulk mRNA abundance and also uncovered gene associations with OS, which can be subtype-specific or subtype-independent.

### TC–TAC transcriptome synergy and antagonism

**Fig. 4:**
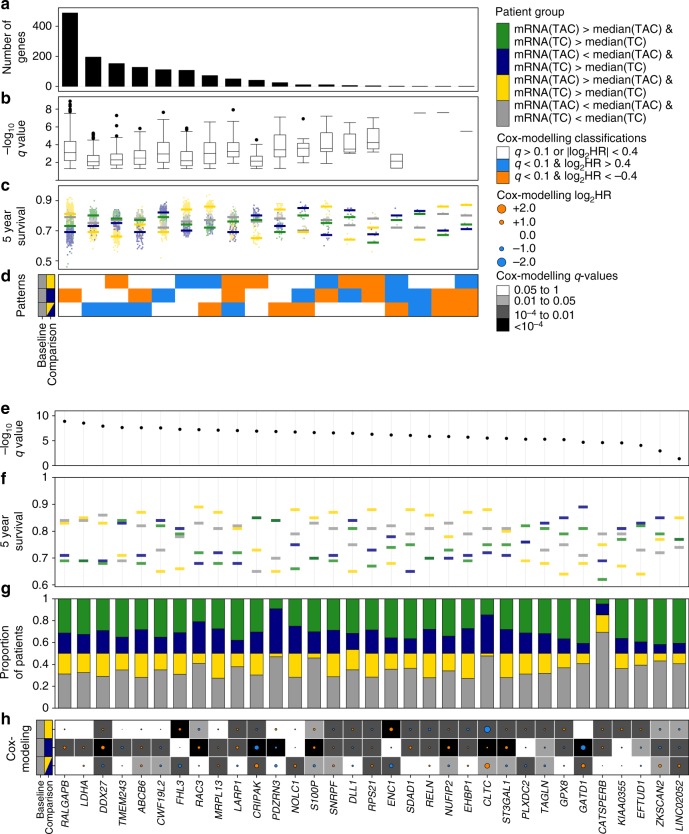
TC–TAC gene interactions. Genes that have significant differences between their TC and TAC mRNA abundance (terms with Wald *q*-value < 0.1 & |log_2_HR| > 0.4 from modelling the TC–TAC interaction) are grouped by Cox modelling results (**a**–**d**) and the two genes from each group with the smallest log-rank *q* are elaborated on (**e**–**h**). Gene classifications are described by the number of significant genes in each classification (**a**), log-rank *q* values (**b**), the proportion of patients who were alive at 5 years (**c**) with the median 5-year survival shown with the line and the Cox modelling results pattern that defines the group of genes (**d**). Selected genes are described again by log-rank *q* value (**e**) and 5-year survival (**f**) this time of the individual genes, also the proportion of patients in each patient group (**g**) and HR and the Wald *q* values from the post hoc Cox modelling (**h**)

After assessing TC and TAC mRNA abundance separately, we investigated how TC and TAC mRNA abundance profiles relate to one another. For each gene, patients were classified into four mutually exclusive groups: mRNA abundance below the median for both profiles, mRNA abundance above the median for both profiles, and the two cases where mRNA abundance was above the median in one but below the median in the other profile. Genes associated with a difference in OS between these four groups (*q* < 0.05, log-rank test) were assessed for statistical TC–TAC interactions in predicting OS. This analysis identified 1408 genes that had at least one of TC mRNA, TAC mRNA or the interaction between the two significantly associating with OS (|log_2_HR| > 0.4, *q* < 0.1, Wald test; Fig. 4a–d), including both additive, saturation and synergistic relationships (Fig. 4e–h).

*CRIPAK* was an example of a saturation relationship between TC and TAC mRNA abundance. *CRIPAK* has been reported as a negative regulator of *PAK1*^29^. Patients with low *CRIPAK* TC mRNA abundance and low TAC mRNA abundance for *CRIPAK* had the worst OS and having either high TC or TAC *FGD3* mRNA abundance was associated with better OS (HR_TC_ = 0.48 & *q* = 4.3 × 10^−7^, HR_TAC_ = 0.76 & *q* = 0.019, HR_interaction_ = 1.4 & *q* = 1.9 × 10^−4^, Wald test; Supplementary Fig. 8). Patients with high *CRIPAK* in both TC and TAC mRNA abundance had prognosis equivalent to patients with high *CRIPAK* in TC but low TAC mRNA abundance; therefore, high TC mRNA abundance was saturating the relationship and no improvement to prognosis was gained by high TAC mRNA abundance. These associations were also identified in HER2-enriched and luminal B breast cancer patients (Supplementary Fig. 9).

*SDAD1* was one of the genes where separating bulk mRNA into TC and TAC mRNA improved risk stratification. The bulk mRNA abundance of *SDAD1* was not associated with OS (HR_bulk_ = 1.0 & *q* = 0.85, Wald test). However, both TC and TAC mRNA abundance were individually prognostic, but in opposite directions (HR_TC_ = 1.6 & *q* = 1.4 × 10^−4^, HR_TAC_ = 0.73 & *q* = 0.040, HR_interaction_ = 1 & *q* = 0.97, Wald test; Supplementary Fig. 8). *SDAD1* was an additive relationship. There was no interaction between the survival relationship of TC and TAC mRNA abundance. *SDAD1* mRNA abundance was not identified as prognostic in any of the individual subtype (Supplementary Fig. 9). *SDAD1* TC mRNA and TAC mRNA abundance appear to affecting outcome through different pathways.

An example of a gene that shows cooperation between TC and TAC mRNA is *FHL3*. The four-and-a-half LIM (FHL) protein family regulate cell proliferation, differentiation and apoptosis through interacting with Smad proteins^30^. *FHL3* had a statistically-significant antagonistic interaction between TC and TAC mRNA abundance levels when predicting OS (HR_interaction_ = 0.60 & *q* = 2.5 × 10^−3^, Wald test). High levels of *FHL3* TAC mRNA abundance was associated with poor OS (HR_TC_ = 1.6 & *q* = 2.3 × 10^−5^, Wald test) but TC mRNA (HR_TAC_ = 0.93 & *q* = 0.60, Wald test). Patients with low mRNA abundance for TC and high mRNA abundance TAC for *FHL3* had the worst OS. Interestingly, patients with high TC and TAC *FHL3* had and low TAC *SHCBP1* had equivalent OS to patients with low TC *FHL3* suggesting high TC *FHL3* and low TAC *FHL3* mRNA levels might be required for bad prognosis (Supplementary Fig. 8). In luminal B patients, high TAC mRNA and low TC mRNA abundance was associated with worse OS for patients with high TC (Supplementary Fig. 9). *FHL3* could be a case of high TC mRNA abundance rescuing the effect of high TAC mRNA abundance.

By grouping genes based on whether TC and TAC mRNA abundance associated with OS, we could assess the overall relationships that exist between the tumour and the microenvironment and overall survival (Fig. 4a–d). Of the genes whose TC or TAC mRNA abundance were associated with OS (either in combination or independently), 43% (600/1408 genes) showed effects only in their TC mRNA abundance. Only 11% (149/1408) of the genes associated with OS showed effects in only their TAC mRNA abundance. There was interplay for 45% (568/1408) of the genes, where TC and TAC mRNA abundances showed a statistical interaction in predicting OS (Supplementary Data 3). Altogether, these data demonstrate that TC and TAC mRNA abundance have intertwined associations for predicting OS and bulk mRNA abundance can hide TC and TAC prognostic associations.

### Multi-gene prognostic biomarkers

**Fig. 5:**
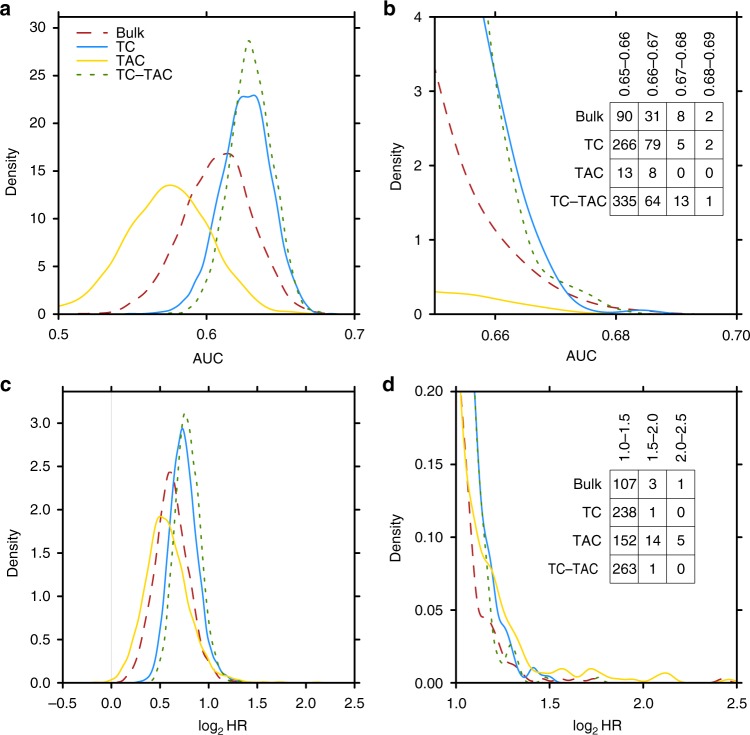
Biomarker generation. Test set results from biomarkers independently generated on each set of profiles. Performance measured using AUC performance (**a**) and log_2_HR (**c**) for 5000 randomly generated biomarkers. Zoom in of the best performing signatures by each metric are shown in **b** and **d**

We demonstrated association with PAM50 subtypes, which themselves are prognostic, as well as gene-wise association of TC and TAC mRNA abundance with overall survival. Both results suggest that other prognostic biomarkers may also be generated from purified data. To test this hypothesis, we generated multi-gene biomarkers from bulk, TC-only, TAC-only and TC + TAC mRNA abundance profiles using machine-learning approaches. We split the data set into a training set of 1430 patients, which were used to create the biomarkers using three-fold cross validation, and a withheld test set of 350 patients for evaluating model performance. The mRNA abundance profiles for each fold and the test sets were independently deconvolved. We sampled the distribution of possible multi-gene biomarkers using three fold cross validation to train random forest models based on random selections of 50 genes per model. The TC mRNA biomarkers were better predictors of OS than bulk mRNA biomarkers and TAC mRNA biomarkers were generally the worse predictors. This held both for overall predictions accuracy (Fig. 5a, b) and for the effect-size of a biomarker (Fig. 5c, d). Genes did not have consistent performance in different profile types. The top biomarkers are composed of different genes in each profile type and we see no association in gene performance between bulk mRNA abundance profiles and either TC or TAC mRNA abundance profiles (Supplementary Fig. 10). Therefore, TAC mRNA abundance have prognostic utility but it is not additive with TC mRNA abundance (Supplementary Table 5).

### DNA–RNA interactions in TACs and TCs

**Fig. 6:**
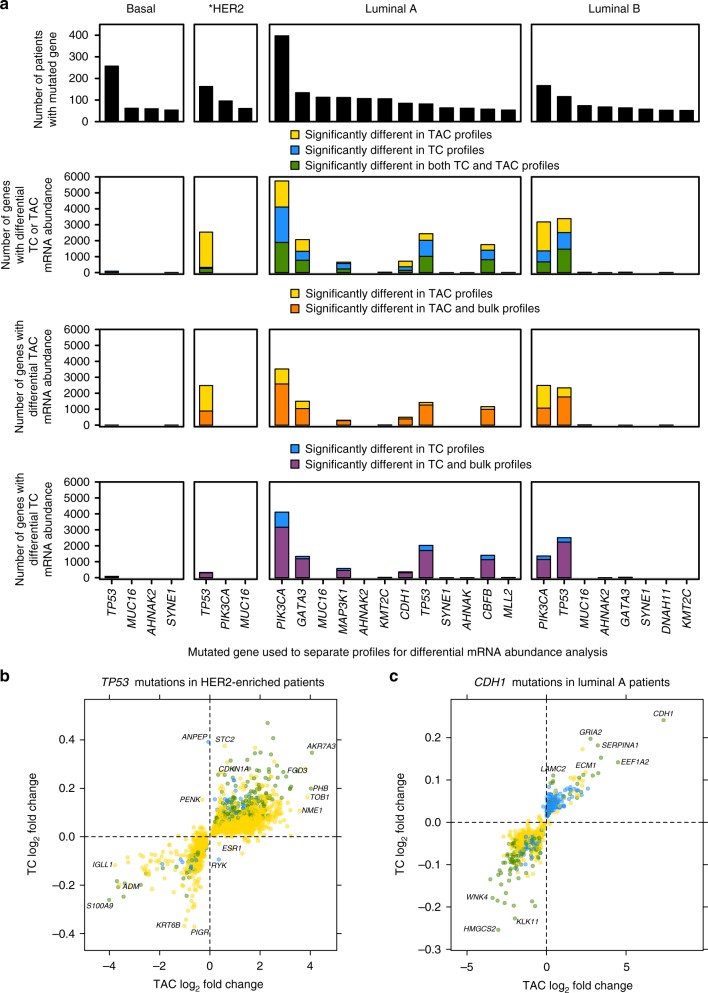
Differential mRNA abundance associated with mutated genes. Counts of genes with differential mRNA abundance associated with mutated genes in each subtype for TAC and TC mRNA abundance (**a**, bottom three panels). The *x* axis are the mutated genes used in the analysis ordered by the frequency that the gene was mutated in each subtype (**a**, top panel). *HER2 = HER2-enriched. Specific genes are elaborated on showing the differences between TC and TAC mRNA abundance fold changes associated with *TP53* mutations in HER2-enriched patients (**b**) and *CDH1* mutations in patients with luminal A breast cancers (**c**)

Given we showed prognostic benefit to separating TC and TAC mRNA from bulk mRNA, we sought to investigate whether we could find mutations associated with mRNA abundance changes. We focused on genes with DNA mutations known to be implicated in cancer^31^. For each gene, we tested for differential mRNA abundance associated with non-silent SNV in the cancer gene. Among the 27 cases of > 50 patients with the same mutated gene within a subtype, seven cases had *cis* mRNA abundance effects where the mutations affected mRNA abundance of that same gene. In luminal B breast cancers, *PIK3CA*, *GATA3* mutations associated with higher *cis* mRNA abundance and *TP53* mutations with decreased *TP53* mRNA abundance. In luminal A breast cancers, *GATA3* mutations associated with higher *GATA3* mRNA abundance and *CDH1*, *TP53*, *CBFB* mutations associated with decreased *cis* mRNA abundance. (Supplementary Data 4). In addition, there were 2433 TC mRNA abundance associations and 5502 TAC mRNA abundance associations that would have been missed if analysis was performed on bulk profiles alone. Subtype associations varied: *MUC16* and *AHNAK2* only associated with mRNA abundance differences in luminal B breast cancers, *PIK3CA* only affected mRNA in luminal A and luminal B breast cancer patients but not HER2-enriched breast cancer patients and *TP53* consistently associated with mRNA abundance differences (Fig. 6a). We used a TCGA breast cancer data set of 902 patients with SNV and mRNA abundance data for validation of the associations. Of the 27 cases we originally tested, *TP53* for basal-like and HER2-enriched breast cancer, *CDH1* for luminal A breast cancer and *PIK2CA* for luminal A and luminal B were each mutated in > 50 patients in the TCGA cohort. The majority of mRNA abundance fold changes correlated between the two data sets (Supplementary Fig. 11).

*TP53* mutations associated with differential mRNA abundance in all four of the subtypes. However, the number of genes with differential mRNA abundance associated with *TP53* mutations varied by subtype. Of the 230 patients with HER2-enriched breast cancer, 163 had *TP53* mutations. When we tested differential mRNA abundance associated with *TP53* mutation in patients with HER2-enriched disease, there were 326 TC mRNA abundance changes and 2483 TAC mRNA abundance changes associated with *TP53* mutations. There were 267 genes with both differential TAC mRNA abundance and TC mRNA abundance associations. For example, mutated *TP53* associated with lower *PHB* mRNA abundance in TACs and TCs (log_2_FC_TAC_ = 4.0, *q* = 2.1 × 10^−4^; log_2_FC_TC_ = 0.19, *q* = 0.026, linear model; Fig. 6b) and with decreased TAC mRNA abundance for *ESR1* (log_2_FC_TAC_ = 0.88, *q* = 0.012; log_2_FC_TC_ = −0.050, *q* = 0.93, linear model). Genes with TAC mRNA abundance differences in patients with *TP53* mutations were associated with various biological processes including negative regulation of blood vessel diameter (*q* = 0.034, hypergeometric test), morphogenesis of a branching epithelium (*q* = 5.3 × 10^−3^, hypergeometric test) and extracellular structure organisation (*q* = 0.032, hypergeometric test; Supplementary Data 4). Finally, there were no *cis* effects of *TP53* in the TAC mRNA abundance in HER2-enriched breast cancers (Supplementary Fig. 12A–C). *TP53* mutations associated with TC and TAC mRNA abundance changes with different effects depending on the breast cancer subtype (Supplementary Fig. 12D, Supplementary Data 4).

We investigated associations with *CDH1* since cadherin is a known cell–cell adhesion protein and its loss results in a change in its invasion pattern. Out of 687 patients with luminal A disease, 85 had *CDH1* mutations. There were 495 TAC mRNA abundance changes associated with *CDH1* mutations in patients with luminal A breast cancer. Of those, 141 genes had both differential TC mRNA abundance and differential TAC mRNA abundances. *CDH1* had strong similar *cis* effects in both TC and TAC mRNA abundance (log_2_FC_TAC_ = 7.3, *q* = 6.1 × 10^−38^; log_2_FC_TC_ = 0.24, *q* = 1.4 × 10^−13^, linear model). Effects on other genes included *PTP4A2* which had differential TAC mRNA abundance (log_2_FC_TAC_ = 0.22, *q* = 0.037; log_2_FC_TC_ = −0.0037, *q* = 0.89, linear model; Supplementary Fig. 12E–F). These genes with differential TAC mRNA abundance associated with mutated *CDH1* were associated with various biological processes including cell surface receptor signalling (*q* = 1.3 × 10^−7^, hypergeometric test), inflammatory response (*q* = 1.1 × 10^−4^, hypergeometric test), innate immune response (*q* = 0.010, hypergeometric test) and positive regulation of T-cell proliferation (*q* = 4.7 × 10^−6^, hypergeometric test; Fig. 6c, Supplementary Data 4). These TAC mRNA changes suggest interplay between non-silent *CDH1* mutations and immune cell dysregulation in the tumour microenvironment. Together, *CDH1* in luminal A breast cancer and *TP53* mutations in HER2-enriched breast cancers demonstrate that different somatic drivers aberrations lead to distinct microenvironmental architectures.

## Discussion

Tumours are complex mixtures of benign and malignant cells, which together form an interconnected tumour environment. The combination of multiple cell populations will need to be considered to truly understand tumour progression and the origins of clinical phenotypes. Here, we show significant differences between bulk, TC and TAC mRNA abundance associations. Using TC mRNA instead of bulk mRNA increased the robustness of biomarker generation. It increased both prognostic power of random multi-gene biomarkers and the number of univariately prognostic genes, demonstrating that there was a better chance of any selected biomarker associating with prognosis and therefore challenging the sensitivity of new biomarkers. Furthermore, we developed biomarkers with roughly equal prognostic associations in both TC and TAC profiles, yet different genes associated with prognosis (Fig. 3d, Supplementary Fig. 10). Although multi-gene biomarkers misclassified a number of patients, our approach produced sufficiently accurate biomarkers to compare the different types of mRNA profiles. METABRIC is sufficiently large to minimise the over-fitting and over-estimation effect common in smaller datasets. Secondly, cases that were incorrectly predicted are possibly enriched in patients for which most biomarkers fail^32^. Overall, computational deconvolution of TC and TAC mRNA creates options for enhanced biomarker discovery.

Although TCs and TACs were more-uniform cell populations than bulk samples, it is important to note that each profile was still a heterogeneous population of cells with distinct microenvironmental pressures^33^. Breast tumours are often multi-clonal^34^, therefore TC mRNA abundances are a mixture of different subclonal populations weighted by their individual prevalences. TAC mRNA abundance is similarly a mixture of different cell types, for instance immune cells and fibroblasts, under distinct microenvironmental stresses^35,36^. This mixture of distinct TAC profiles may explain in part the large per gene variation of TAC mRNA abundances. Single-cell sequencing may one day provide an ideal way to understand the heterogeneity of both TCs and TACs, and their interactions. To date however, there are no single-cell technologies capable of profiling thousands of clinical samples with tractable costs, making computational strategies on bulk data attractive strategies to uncover TC–TAC associations.

There are many computational methods for deconvolving bulk transcriptomic profiles. Some, like Cibersort^37^, quantify specific known cell populations. The method used here, by contrast, evaluates the contribution of the full tumour microenvironment, including cell populations with unknown transcriptomes, such tumour-adjacent stroma cells. The TAC profiles generated here provide significant information not found in either bulk profiles nor cell type proportions, including different subtype associations, survival associations and relationships with somatic mutations. Thus, it appears that there are benefits to both general category of deconvolution approaches to help expand our characterisation of tumours and their microenvironments. Indeed these data highlight the need for improved methods that combine cell type agnostic and cell type-specific strategies, and the development of benchmarking strategies to quantitatively assess the accuracy of different methods in different biological contexts.

As TAC mRNA abundance is not statistically additive with TC mRNA abundance in multi-gene biomarkers, it could mean that the mRNA abundance associations originate from the same source, possibly somatic mutations. Along with prognostic associations, computationally separating TC and TAC mRNA revealed mRNA abundance differences linked to genomic drivers. We showed TC and TAC mRNA abundance can help to better understand the effect of driver mutations in different subtypes and which subtypes non-silent mutations may or may not have an effect. For example, mutated *PIK3CA* showed many associations with differential mRNA abundance in the luminal breast cancers but no associations were detected in HER2-enriched breast cancers. Within specific subtypes, we looked for functional associations. For example, mutated *CDH1* was associated with differential TAC mRNA abundance for genes involved in immune activation and proliferation suggesting non-silent *CDH1* mutations may dysregulate immune cells in the tumour microenvironment. These examples hint that we can use these relatively low-cost computational approaches to better understand the tumour microenvironment.

Although we find evidence of microenvironment dysregulation through tumour mutations, further experimental work is still needed to understand the mechanisms and to validate these findings. This could involve evaluating the microenvironment in in vitro and in vivo model systems with loss of E-cadherin^38^ or, similarly, other cancer genes where we have identified associations. Or even simpler, if we compare predicted results to primary tumours and cell lines, we expect TC mRNA abundance results to be closer to cell lines than to primary tumours. Computational analysis of TC and TAC mRNA associations can help guide experimental exploration of tumour microenvironment associations.

These results that motivate further studies into TC–TAC interactions and the contribution of TAC transcriptome changes to cancer development and progression. There are many existing datasets in which the approach demonstrated here could be used to explore TAC biology, particularly in a pathway context^39^. Overall our results suggest that in silico derived TC and TAC mRNA abundance are an advantageous framework to explore the complex transcriptional network between tumours, their microenvironments and cancer aggression.

## Methods

### Data sets

Analysis was performed on the METABRIC cohort which contains 1991 patients each with a primary fresh frozen breast cancer specimen that had mRNA abundance profiled using Illumina HT-12 v3 microarrays^22^. These profiles are referred to as the bulk profiles. METABRIC annotation includes overall survival, pathologist determined purity estimates and PAM50 subtype classifications. Six patients with NC (not classified) for their subtype classification were excluded. Also excluding normal-like breast cancers, 1780 patients were used for the main analysis. The cohort also has mRNA profiles from Illumina HT-12 v3 microarrays for 144 adjacent normal breast tissue samples from a subset of the patients with breast cancer samples. Age and ethnicity distributions of the samples in the full set and the subset were similar. The METABRIC data set includes the relative mRNA abundances of 19,877 genes. The METABRIC cohort also includes targeted sequencing data covering 173 genes frequently mutated in breast cancer (i.e., candidate driver genes) with somatic SNVs predictions^31^.

For validation, we used the breast cancer samples from the Cancer Genome Atlas (TCGA), downloaded from the Broad GDAC Firehose (https://gdac.broadinstitute.org/), release 28 January 2016. There were 1014 patients with mRNA abundance profiles, which were from Illumina HiSeq rnaseqv2 level 3 RSEM normalised profiles. Genes with >75% of samples with zero reads were filtered out. SNV profiles were based on TCGA-reported MutSig v2.0 calls. There were 20,531 genes with mRNA abundance from the RNA-Seq and 18,825 overlapped with METABRIC mRNA abundance profiles. There were 978 patients with mutated gene information for the 173 genes METABRIC had SNV data for.

### TC profiles

The TC mRNA abundance profiles were deconvolved from the bulk cancer mRNA abundance profiles in METABRIC and a panel of 144 non-cancerous breast tissue mRNA abundance profiles using ISOpure^17^ run on MATLAB release 2010b. Patients were grouped using METABRIC PAM50 classifications and each subtype was independently run through ISOpure using the same panel of non-cancerous profiles. ISOpure deconvolution was performed with mRNA abundance in normal-space but all analysis was performed with mRNA abundance in log_2_-space.

To create TC mRNA abundance profiles, the ISOpure algorithm from Quon et al.^17^ was used as described without any adjustments. The algorithm is a two-step process. In the first step the tumour purity for each sample is estimated and in the second step the mRNA abundance profiles are estimated using the purities from step one. In order to estimate the samples’ tumour purities, the average sample for the cohort is used. As breast cancer subtypes are considered separate diseases, one average per subtype was more appropriate than treating all patients as having the same disease. We used a different average for each subtype by running each subtype as a separate run.

For a negative control, we randomly split the 144 normal samples from the METABRIC data set into 75 samples that were run through ISOpure for purification as the negative control and the remaining 69 samples continued as the normal panel for the negative control ISOpure run. This was performed as one ISOpure run.

### Purity and TAC profile estimation

Purity estimates are concurrently estimated when estimating the TC mRNA abundance using ISOpure. The TAC mRNA abundance profiles associated with the tumour were estimated in normal-space using:

1 $${\mathbf{b}} = p\,{\mathrm{x}}\,{\mathbf{t}} + \left( {1 - p} \right)\,{\mathrm{x}}\,{\mathbf{s}}$$

where **b** is the bulk METABRIC mRNA abundance, **t** is ISOpure estimated TC mRNA abundance, *p* is ISOpure’s estimate of the proportion of the sample that is tumour and **s** is the TAC mRNA abundance we want to estimate. In order to transform the TAC mRNA abundance profiles into log_2_-space, any value below 0 was set to the minimum mRNA abundance from the bulk and TC mRNA abundance profiles prior to log_2_ transformation. R version 3.2.1 was used. The code has also been integrated in to ISOpureR (≥ v1.1.0) as the function ISOpure.calculate.tac() and is available through CRAN.

### Comparing ISOpure and pathologist purity estimates

As pathologist purity estimates are categorical, we conservatively selected patients with higher ISOpure and those with higher pathologist estimates. Low-moderate cellularity is described as <40% of cells being TCs. Moderate cellularity is described as 40–70% TCs. High cellularity is >70% TCs. We defined patients as having higher ISOpure estimates if they were moderate cellularity and had ISOpure estimates >95%, or low cellularity and had ISOpure estimates >65%. We defined patients as having higher pathologist estimates if they were moderate cellularity and had ISOpure <15% or high cellularity with ISOpure estimates <45%. There were 89 patients with higher pathologist purity estimates and 23 patients with higher ISOpure purity estimates. Each group was statistically compared to the remaining 1,668 patients (Supplementary Table 1).

### PAM50 analysis

The mRNA abundance were scaled by subtracting the mean and dividing by the standard deviation per profile type for each gene. Clustering in heatmaps was performed using the DIANA algorithm with 1—Pearson correlation distances for hierarchical clustering on only PAM50 genes. PAM50 classification was performed in the R statistical environment (v3.4.3) using the genefu (v2.8.0) package.

The R package limma^40^ (v. 3.32.10) was used to find genes with differential mRNA abundance between each subtype and the other three subtypes. The ten genes with the highest rankings minimum ranking from the adjusted *p* values and the absolute log_2_ fold change were selected for each subtype.

### Subtype clustering

Consensus clustering was performed using R package ConsensusClusterPlus (v1.8.1) on genes that had a standard deviation greater than one for any of bulk, TC or TAC mRNA abundance. Clustering parameters included ward linkage, Jaccard distance metric, 0.8 feature and item sub-sampling and a seed of 17.

### Identifying unexpressed genes

As the METABRIC data set is all female patients, genes on chromosome Y can be used to identify a threshold for genes that were not expressed. For the METABRIC data set, chromosome Y genes had maximum values of ~ 6.5, which was consistent between bulk, tumour and TAC mRNA abundance (Supplementary Fig. 6F). Unexpressed genes were thus defined as those with intensity <6.5 in all patients.

### Univariate survival analysis

For each gene, patients were dichotomised by the gene’s mRNA abundance. Median mRNA abundance level was used for dichotomisation unless the median was below the unexpressed threshold and there were at least 79 patients above the unexpressed threshold (80% power for HR 2), in which case the unexpressed threshold was instead used to dichotomise the gene. High and low abundance groups were compared by univariate Cox proportional hazards modelling for survival to 5 years. This was repeated for each type of profile independently and the resulting Ward *p* values were false discovery rate (FDR) adjusted. Only genes that passed the Cox assumptions (coxzph *p* > 0.1) and were above the unexpressed threshold for at least one patient were considered significant if they met the HR and Ward *q* value thresholds. If the median mRNA abundance was below the unexpressed threshold and there were less than 79 patients above the unexpressed threshold then the gene was removed from the significant gene results. Cox modelling was performed in the R statistical environment (v3.2.1) using the survival package (v2.38–3).

### Functional profiling/ pathway analysis

To determine associated biological processes, entrez gene ids were converted to gene symbols. Unique gene symbols were then input into the web version (http://biit.cs.ut.ee/gprofiler/) of g:Profiler^41^ (v r1732_e89_eg36) using the default options for *Homo sapiens* except outputting results to “Textual (download)”. Hypergeometric test with g:SCS adjustment are used to calculate *q* values within g:Profiler^41^.

### Intersecting proportion of significant genes for *p* value sensitivity

Out of the smaller gene list, the proportion that was also found in the larger gene list.

2 $${\mathrm{Intersecting}}\,{\mathrm{proportion}} = |A \cap B|/\min |A|,|B|$$

### Gene-wise TC–TAC interactions

For each gene, patients were classified into one of four groups: high mRNA abundance in both TC and TAC profiles, high mRNA abundance using TC profiles but low mRNA abundance using TAC profiles, low mRNA abundance using TC profiles but high mRNA abundance using TAC profiles or low mRNA abundance in both profiles. High and low mRNA abundance groups were defined using the same dichotomisation criteria as the univariate survival analysis. Using a log-rank test, groups were assessed for a difference in survival up to 5 years. All log-rank *p* values were FDR adjusted and, if *q* value was below 0.05, Cox proportional hazards modelling was performed with the formula:

3 $${\mathrm{survival}}\sim TAC + TC + TC\,{\mathrm{x}}\,TAC$$

The Ward *p* values for each of the three term from the Cox model were FDR adjusted. Only terms that passed the Cox assumptions (coxzph *p* > 0.1) and were not unexpressed across all patients were considered significant if they met the HR and *q* value thresholds. Cox modelling was performed in the R statistical environment (v3.2.3) using the survival package (v2.38–3).

### Random biomarker distributions generation

The same random 5,000 biomarkers were compared on bulk mRNA abundance, TC mRNA abundance, TAC mRNA abundance and TC + TAC mRNA abundance. For each biomarker, we selected 50 random genes and used a random forest classifier to predicted survival at 5 years on each of the profile types. For TC + TAC, the 50 mRNA abundance profiles from both TC and TAC were used in the model. The mtry parameter and the random forest classification threshold were optimised using three-fold cross validation on 1430 patients to maximise AUC. Biomarkers were tested on a withheld 350 patients. Random forests were run with 10,000 trees. Other than gene selection, everything was independent on each profile type. Biomarker generation was performed in the R statistical environment (v3.2.) using the randomForest (v4.6–12) and pROC (v1.8) packages.

TC and TAC mRNA abundance profiles for biomarker generation were deconvolved for each fold separately for cross-validation, all folds together for training the model and on the withheld test set. As before subtypes were run independently per subtype, however the open-source ISOpureR (v1.0.19) was used, which generates numerically indistinguishable results and allows greater parallelisation^42^. ISOpure step 1 was run using the seed 374 and step 2 was run with the seed 719.

### Differential mRNA abundance associated with mutated genes

For each of the genes with non-silent mutations in the METABRIC Dataset^31^, genes with non-silent SNVs in >50 patients were individually used to separate patients into those with and without the mutated gene. The R package limma^40^ (v3.32.10), linear models with FDR adjustment, was used to determine genes with differential mRNA abundance associated with the mutated gene for each mRNA abundance profile.

For TCGA, only the cases of subtype and mutated gene that had been analysed in METABRIC were considered. Of those, five had >50 patients with the gene mutated in that subtype.

### Visualisation

All plotting was performed in the R statistical environment (v3.4.3) using the lattice (v0.20–38), latticeExtra (v0.6–28), RColorBrewer (v1.1–2) and cluster (v2.0.7–1) packages via the BPG plotting framework^43^ (v5.9.8).

### Reporting summary

Further information on research design is available in the Nature Research Reporting Summary linked to this article.

## Supplementary information

Supplementary Information

Description of Additional Supplementary Files

Supplementary Data 1

Supplementary Data 2

Supplementary Data 3

Supplementary Data 4

Supplementary Data 5

Supplementary Data 6

Supplementary Data 7

Supplementary Data 8

Supplementary Code 1

Reporting Summary

## Data Availability

The METABRIC data^22,31^ referenced during the study are available from https://www.ebi.ac.uk/ega/datasets/EGAD00010000162 and https://github.com/cclab-brca websites. The TCGA BRCA data sets are available from the https://gdac.broadinstitute.org/ website, release 28 January 2016. The TAC and TC mRNA abundance profiles are provided in Supplementary data sets 5–8. All the other data supporting the findings of this study are available within the article and its supplementary information files and from the corresponding author upon reasonable request.

## Code availability

Knitr documentation for analysis and all figures is available in the supplementary code. The function to create TAC profiles is available in ISOpureR (≥ v1.1.0) [https://cran.r-project.org/web/packages/ISOpureR/index.html]. For analysis, version numbers are specified within each part of the methods. Generally, R statistical environement v3.2.1—v3.4.3 was used with the R packages: genefu (v2.8.0), limma (v. 3.32.10), ConsensusClusterPlus (v1.8.1), survival (v2.38–3), randomForest (v4.6–12), pROC (v1.8) and lattice (v0.20–38), latticeExtra (v0.6–28), RColorBrewer (v1.1–2) and cluster (v2.0.7–1) packages via the BPG package (v5.9.8).
