# Supplementary material for: Landscape of transcriptomic interactions between breast cancer and its microenvironment
Source: Nat Commun. 2019 Jul 15;10:3116. doi: 10.1038/s41467-019-10929-z (PMC6629667; doi:10.1038/s41467-019-10929-z)
Supplement: Supplementary file 1 — Supplementary Information [file 41467_2019_10929_MOESM1_ESM.pdf]

Supplementary Information for “Landscape of transcriptomic interactions between breast cancer and its microenvironment”

Fox *et al.*

Supplementary Figures

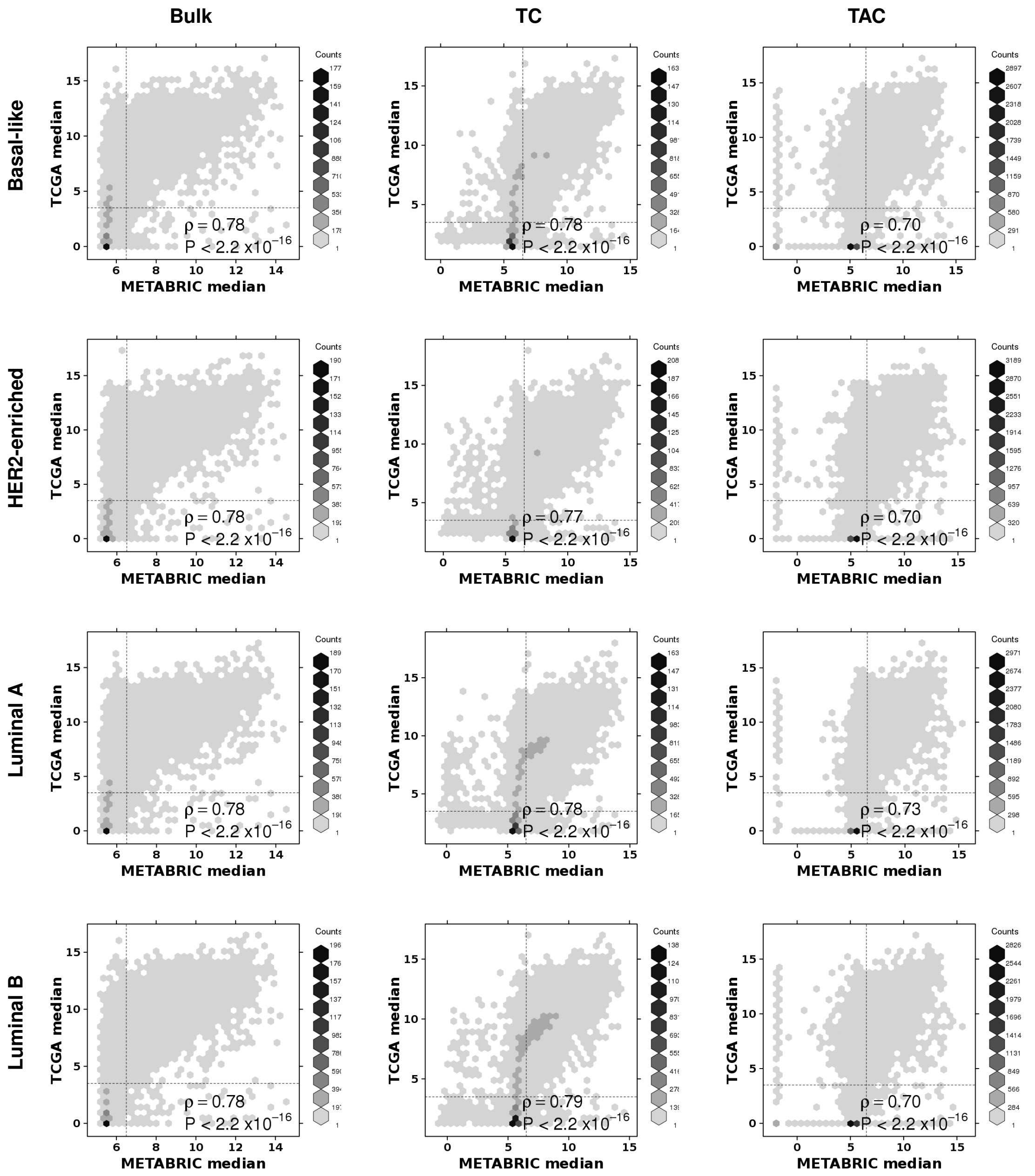

Supplementary Figure 1 | METABRIC and TCGA mRNA abundance correlation. Correlation of the median per gene for bulk, TC and TAC mRNA abundance between METABRIC and TCGA.

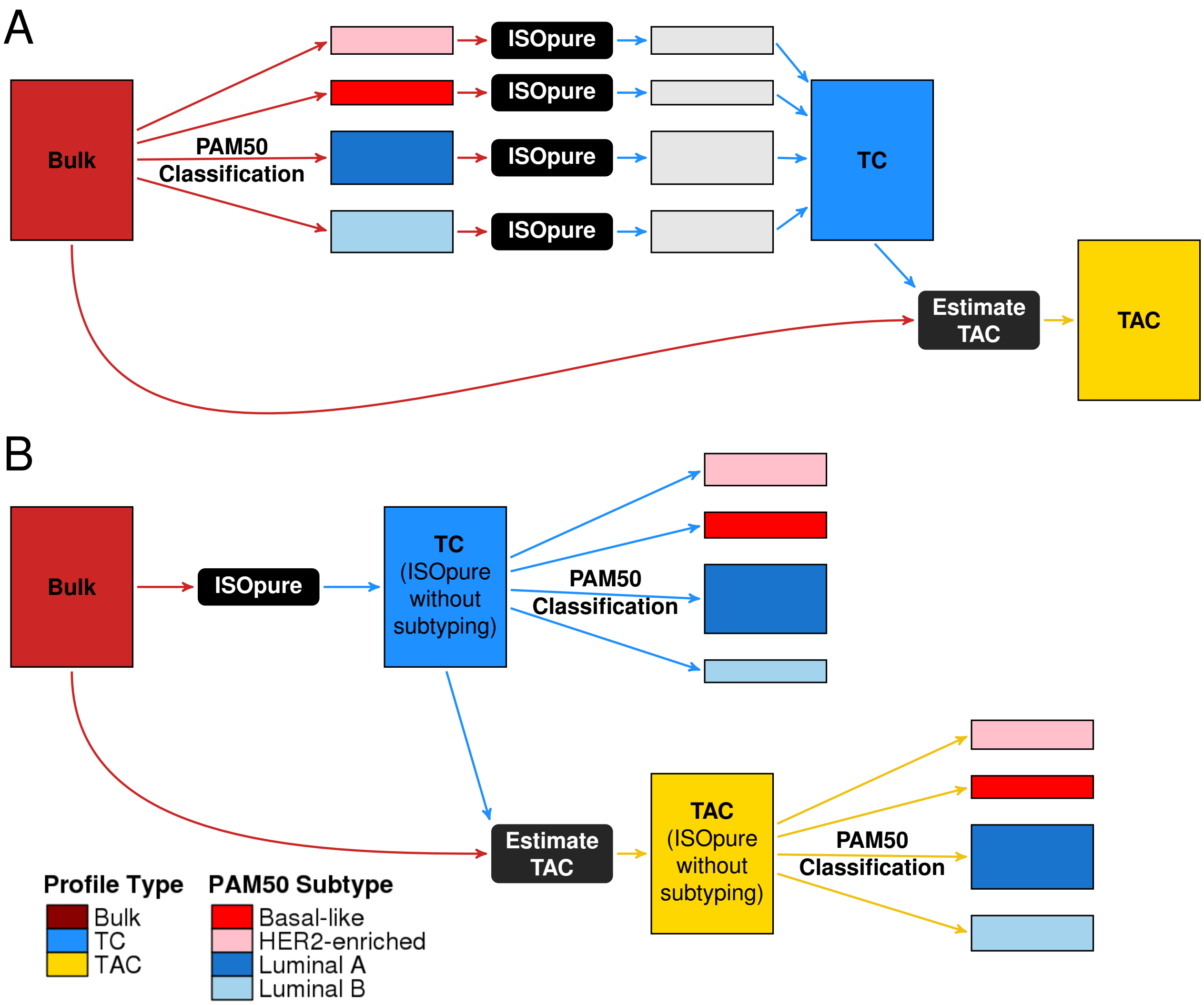

Supplementary Figure 2 | Deconvolution approach. (A) TC mRNA abundance were deconvolved by separating bulk mRNA abundance by PAM50 classifications and running ISOpure independently for each subtype. TAC mRNA abundance were then estimated using the bulk and TC mRNA abundance. (B) For comparison we also classified patients using TC and TAC mRNA abundance deconvolved without running ISOpure independently for each subtype.

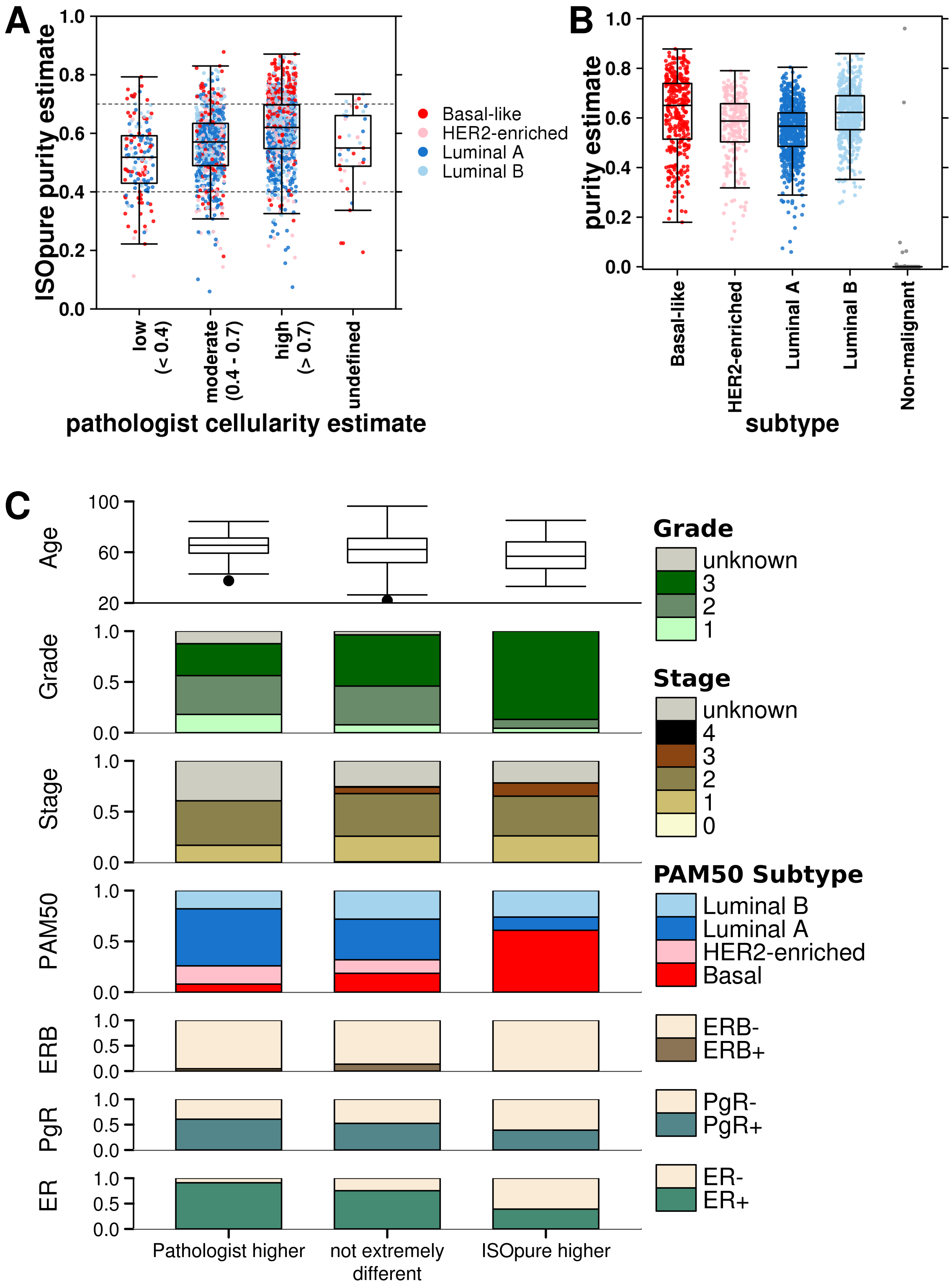

Supplementary Figure 3 | Purity estimates. (A) Association between ISOpure purity estimates and pathology cellularity estimates. Tukey boxplots are shown. (B) Purity estimate differences between subtypes. Non-malignant samples are adjacent normal samples that should have no or very few tumour cells. Tukey boxplots are shown. (C) Associations with pathologist or ISOpure estimating higher purity. The distribution of age, tumour stage and grade, patient PAM50 subtype assignment and receptor status of ERB, PgR and ER associated with large differences in purity estimates from pathologists and ISOpure. Pathologists assigning much higher purity estimates than ISOpure (ISOpure was 0.25 less than 0.4 for moderate pathologist cellularity or 0.7 for high pathologist cellularity; 89 patients had these purity estimates) or ISOpure assigning much higher purity estimates than pathologists (ISOpure was 0.25 more than 0.7 for moderate pathologist cellularity or 0.4 for low pathologist cellularity; 23 patients had these purity estimates) were compared to the 1,668 patients that did not have these extreme differences in their purity estimates. With the exception of age, the y-axis is showing the proportion of the group.

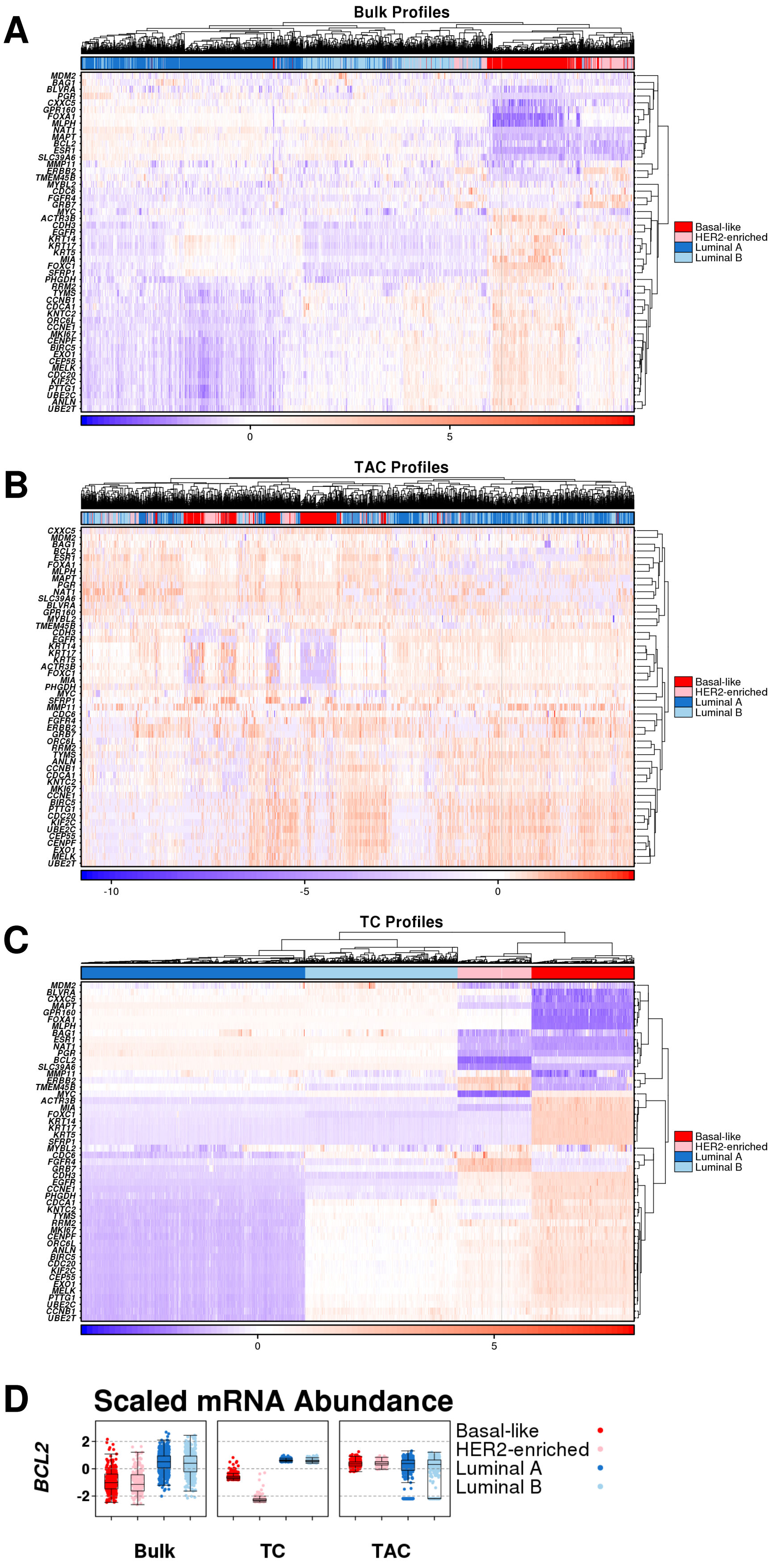

Supplementary Figure 4 | PAM50 mRNA abundance. (A-C) Patient clusters using PAM50 gene mRNA abundance for bulk mRNA abundance (A), TAC mRNA abundance (B) and TC mRNA abundance (C). Covariate bar on top of the heatmap shows the PAM50 subtype classification from the METABRIC publication. (D) The mRNA abundance scaled over all patients for each subtype and each of bulk, TC and TAC mRNA abundance for the *BCL2*.

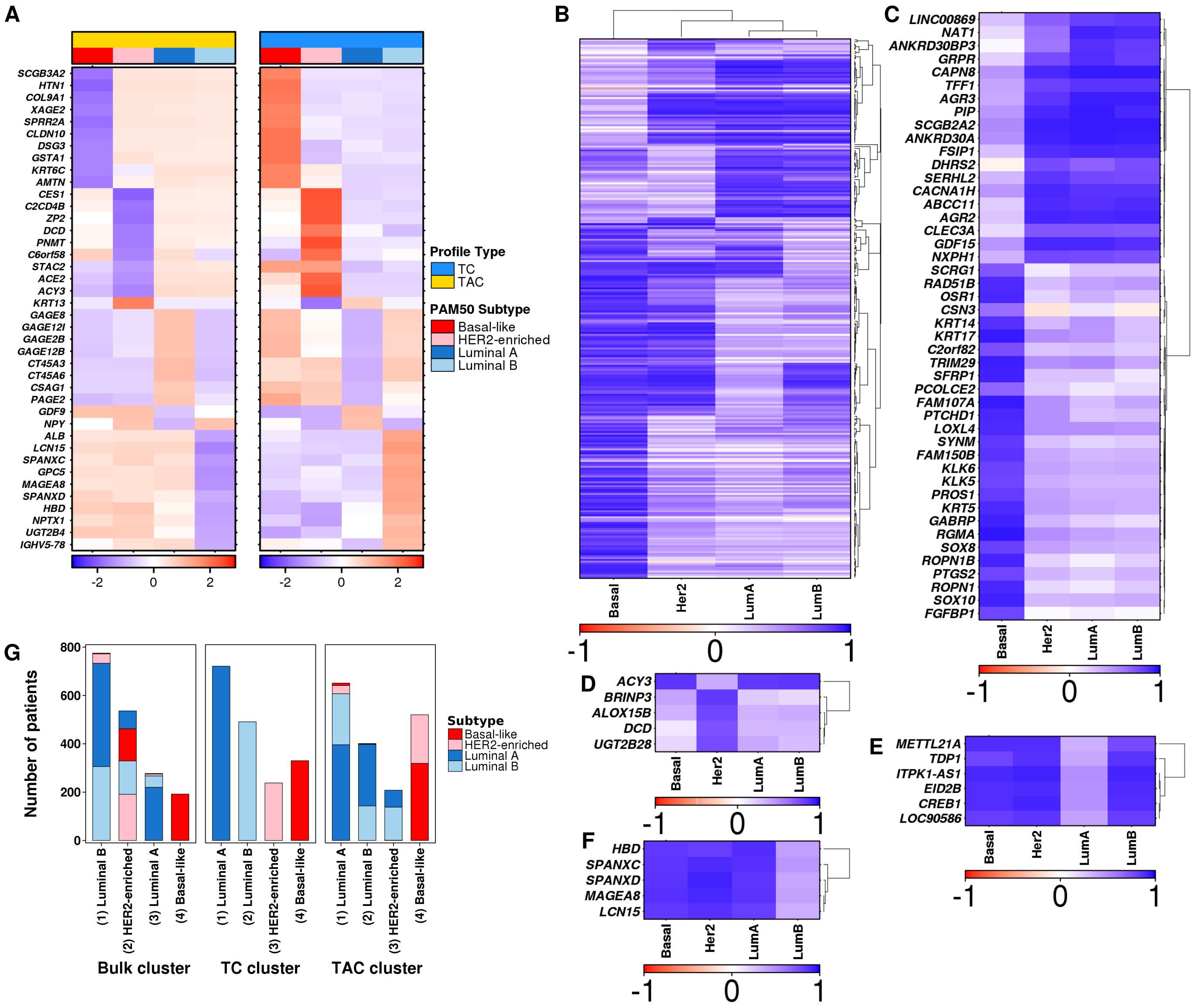

Supplementary Figure 5 | TAC mRNA abundance subtype associations. Genes with TAC mRNA abundance associated with the intrinsic breast cancer subtypes. For each subtype, the top 10 genes with the largest difference in mRNA abundance between each subtype and the other three subtypes was selected. (B-F) Subtype differences between TC-TAC correlations. Genes with correlations between their TC and TAC mRNA abundance that differ by at least 0.4 between subtypes were shown in (B). Genes where the TC-TAC mRNA levels correlation for basal-like (C), HER2-enriched (D), luminal A (E), and luminal B (F) breast cancer patients differs from the all the other subtypes by at least 0.4. (G) Subtype labelling for profile clusters. Along the x-axis are groups created by running consensus clustering independently on the most variable genes (genes with standard deviation > 1.0 in at least one mRNA abundance type) each of bulk, TC and TAC mRNA abundance. The cluster id number is shown in brackets followed by the subtype label assigned to the cluster. Each barplot shows the number of patients from each subtype that were clustered into each group. Subtype classification from the METABRIC publication was used for colouring the bars. ISOpure emphasizes similarities across samples run together, which led to emphasizing the differences in TC mRNA abundance across these subtypes. Therefore there was artificially high concordance of TC mRNA abundance PAM50 classification with the breast cancer subtypes. However, there was significant overlap between subtypes of the TC mRNA abundance of individual genes so at least at the level of individual genes, TC mRNA abundance did not align perfectly with the experimental groups. Furthermore, the tightening of each subtypes' TC mRNA abundance distribution to more clearly show the known hormone receptor-subtype relationships in breast cancer suggests that TC mRNA abundance was more accurately recapitulating the cancer's mRNA.

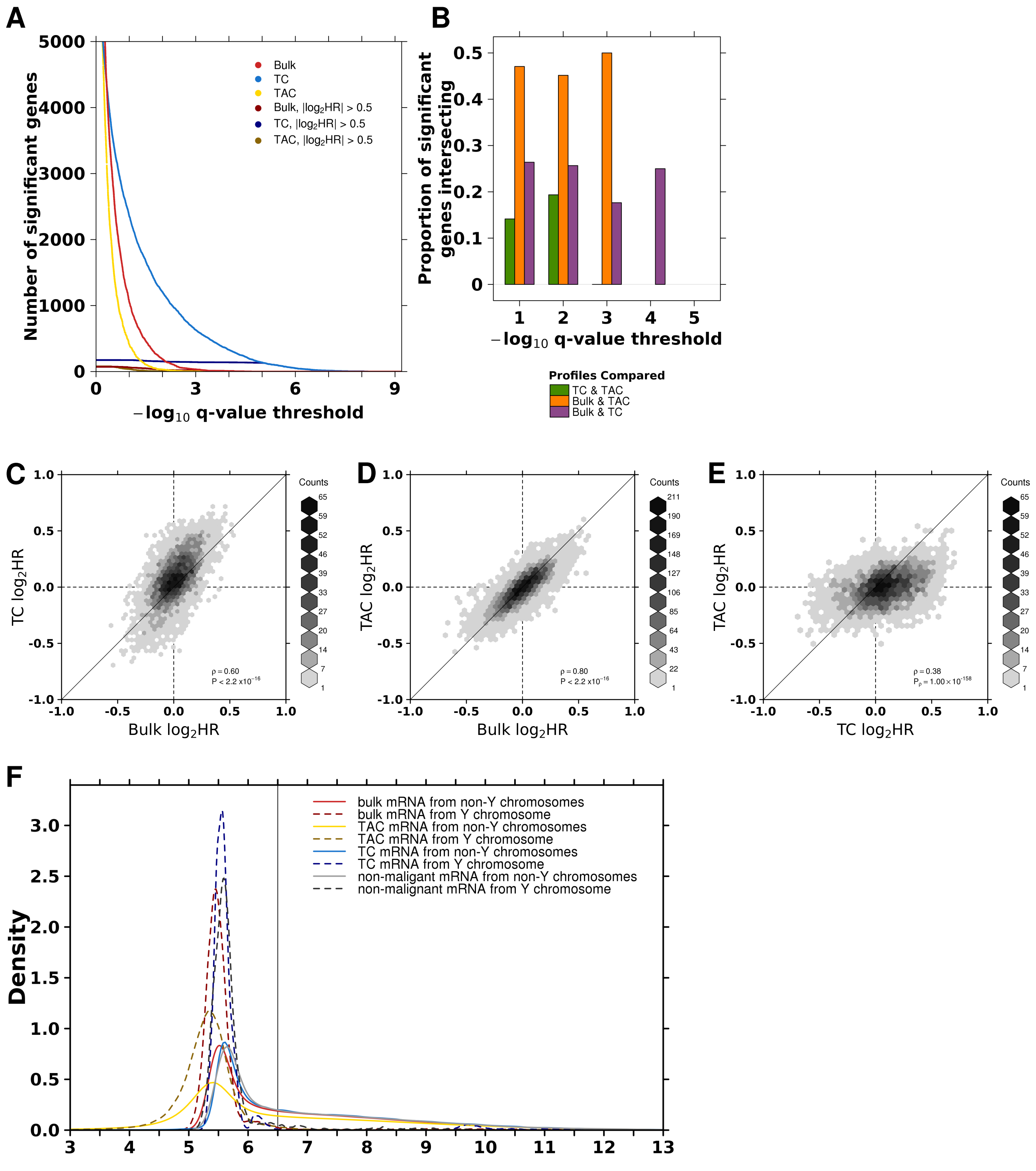

Supplementary Figure 6 | Univariate Cox-modeling analysis. (A) The number of significant genes given different Wald q-value thresholds. Unexpressed genes and genes that did not pass the Cox modeling assumptions (coxzph p > 0.1) are not included in all counts. The two sets of curves show the q-value thresholds in concert with three different HR thresholds. The first set has no restrictions on the HR, the second defines significant as q-value $<$ x and $|\log_2\text{HR}| > 0.5$. (B) The proportion of significant genes that agree between gene lists (intersect of the lists divided by the min number of significant genes between the two lists) at different q-value thresholds. (C-E) The correlation of $\log_2$HR for all genes that passed the proportional hazard assumptions in both profile types before significance filtering. Spearman's correlation is reported. (F) Unexpressed mRNA abundance threshold. The mRNA abundance for genes on the Y chromosome (dashed lines) and genes not on the Y chromosome (solid lines) for bulk mRNA abundance (red), TC mRNA abundance (blue), TAC mRNA abundance (gold) and non-malignant mRNA abundance (gray). Non-malignant samples are adjacent normal samples.

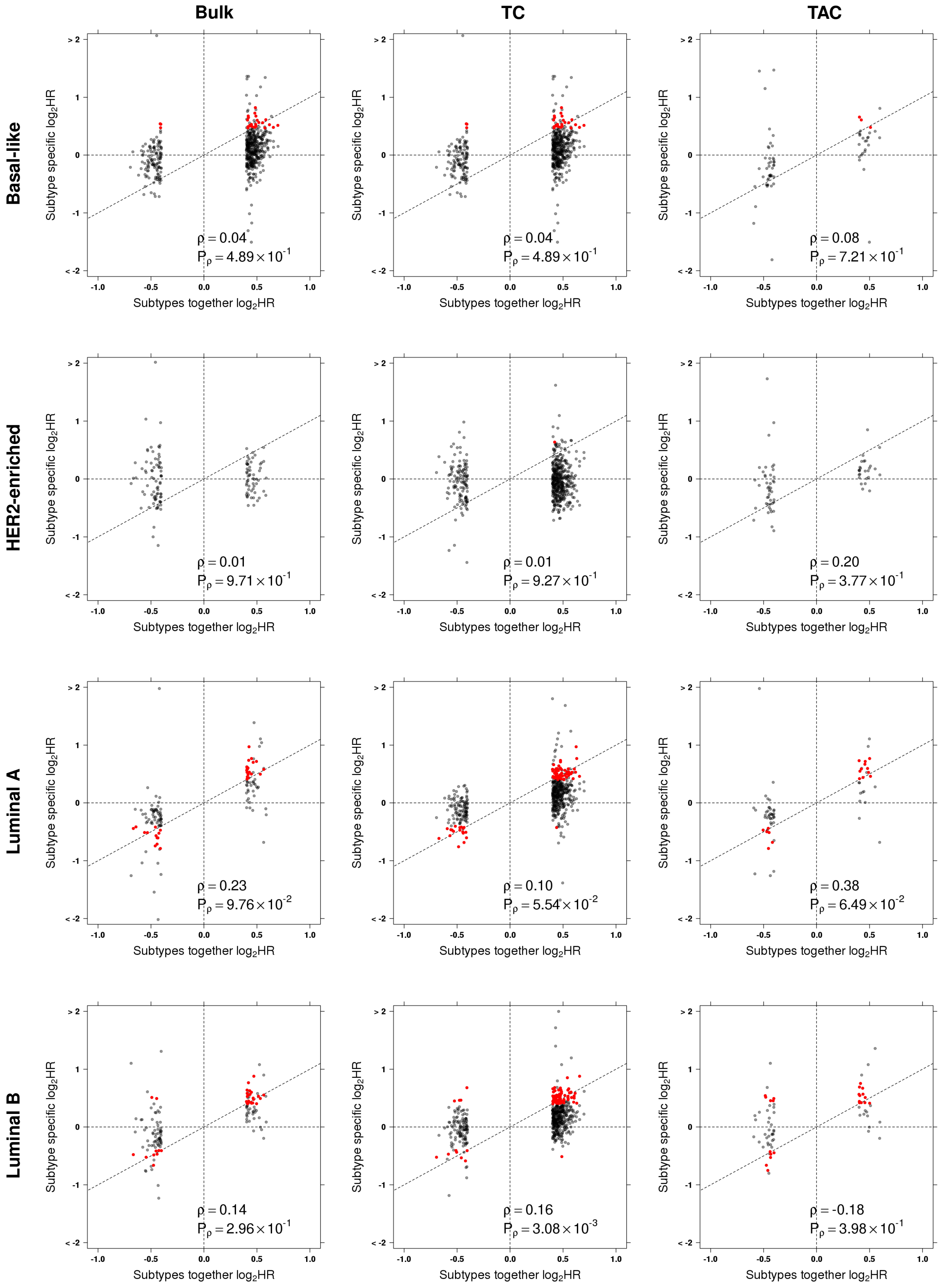

Supplementary Figure 7 | Correlation of univariate Cox modelling hazard ratio between subtype specific analysis and all patients. The correlation of $\log_2$HR for all genes that passed significance filtering using all patients and the $\log_2$HR from the same univariate analysis per subtype. The genes that are significant in both are shown in red. Spearman's correlation is reported.

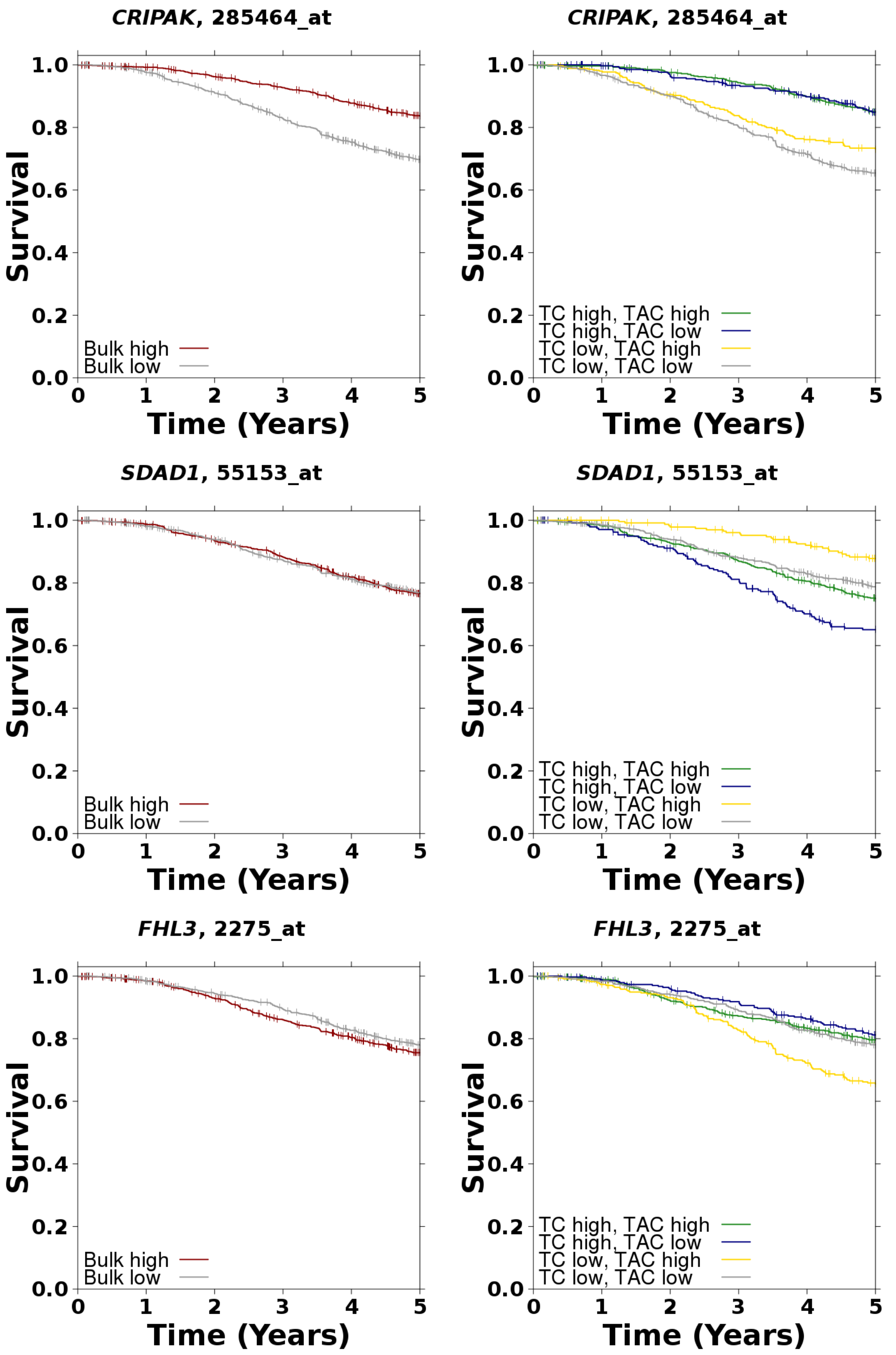

Supplementary Figure 8 | *CRIPAK*, *SDAD1*, *FHL3* prognosis. Kaplan-Meier curves for *CRIPAK*, *SDAD1*, *FHL3* comparing bulk profiles to the relationship between TC and TAC mRNA abundances.

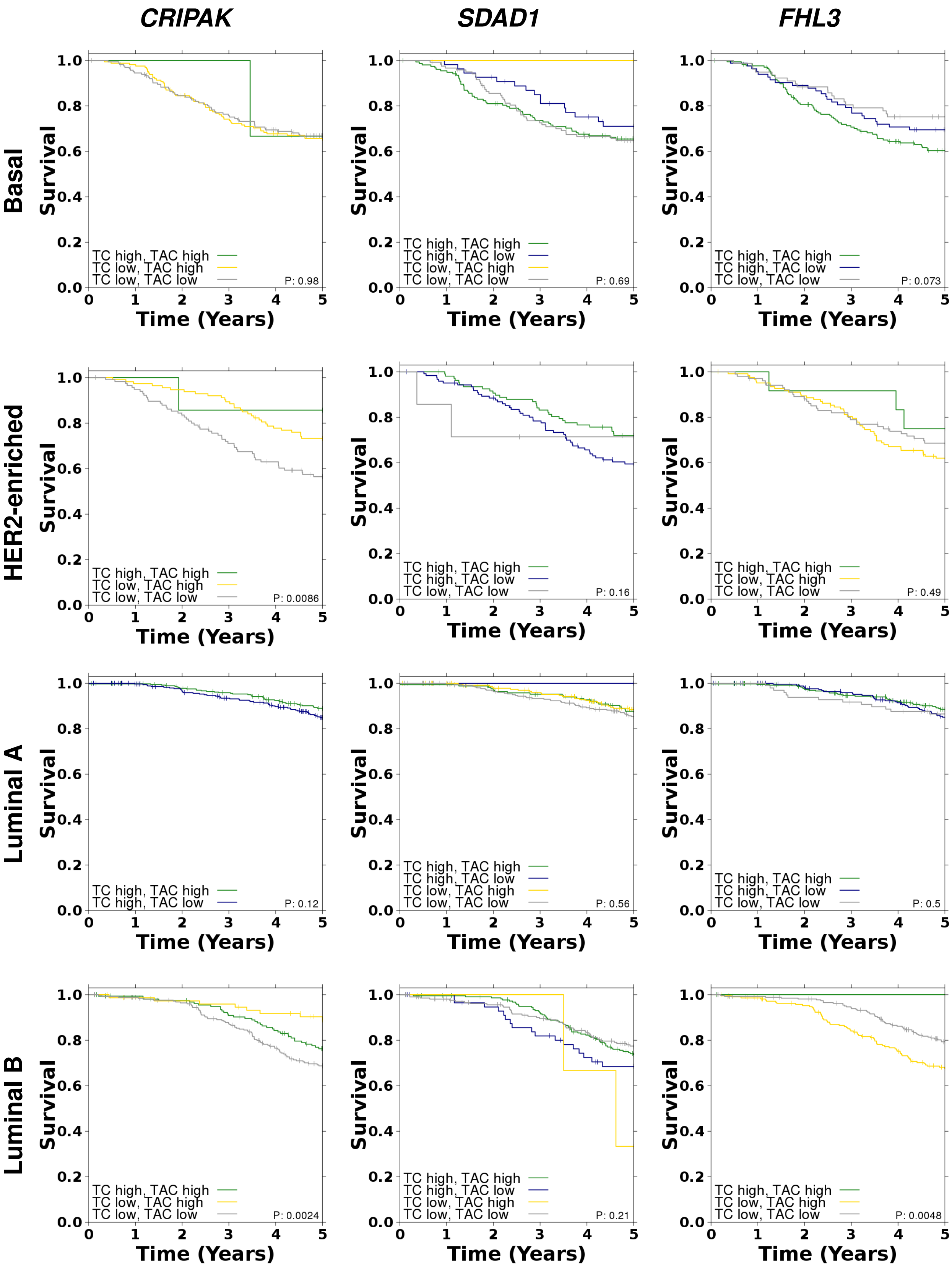

Supplementary Figure 9 | *CRIPAK*, *SDAD1*, *FHL3* prognosis per subtype. Kaplan-Meier curves for *CRIPAK*, *SDAD1*, *FHL3* showing TC and TAC mRNA abundance association with prognosis in each intrinsic subtype. Logrank p-values are shown in the bottom right corner of each plot.

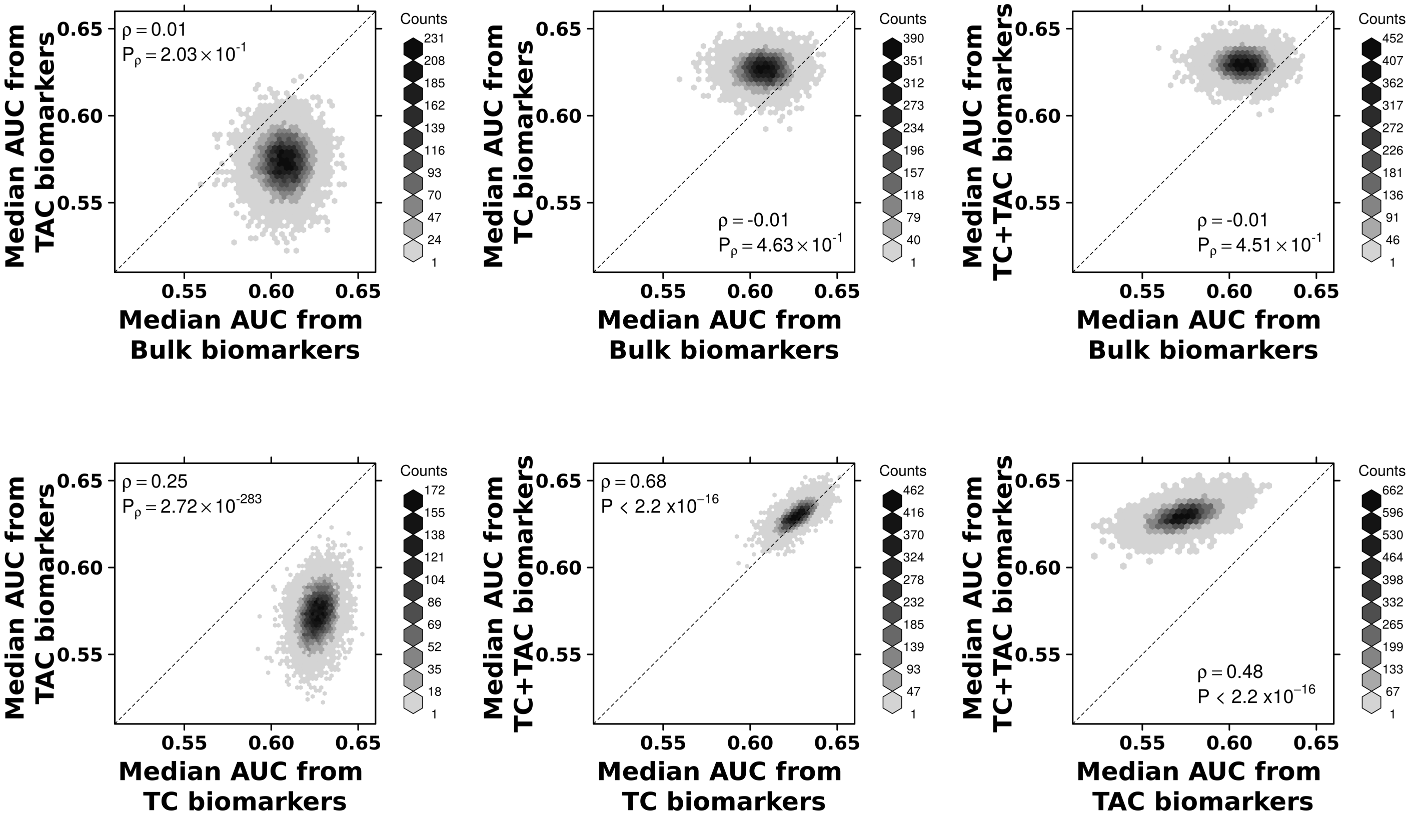

Supplementary Figure 10 | Gene contribution to multi-gene signatures. Representing each gene with the median AUC from all the signatures that contained that gene, we compared between mRNA abundance profile types whether genes contributed relatively the same to signature performance. Spearman's correlations are reported.

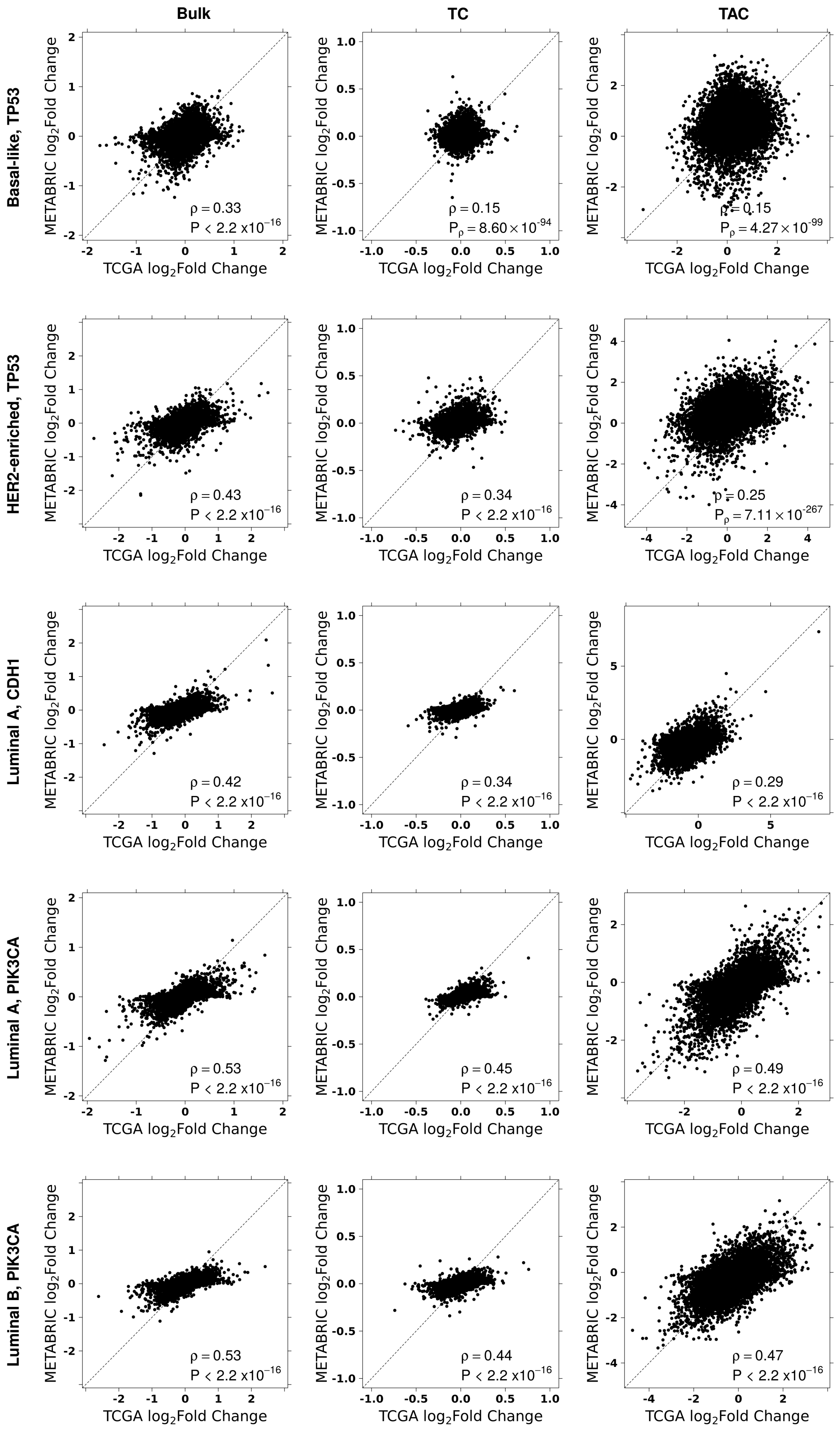

Supplementary Figure 11 | METABRIC and TCGA SNV association correlations. Correlation of $\log_2$ fold change in bulk, TC and TAC mRNA association with commonly mutated genes. Spearman's correlations are reported.

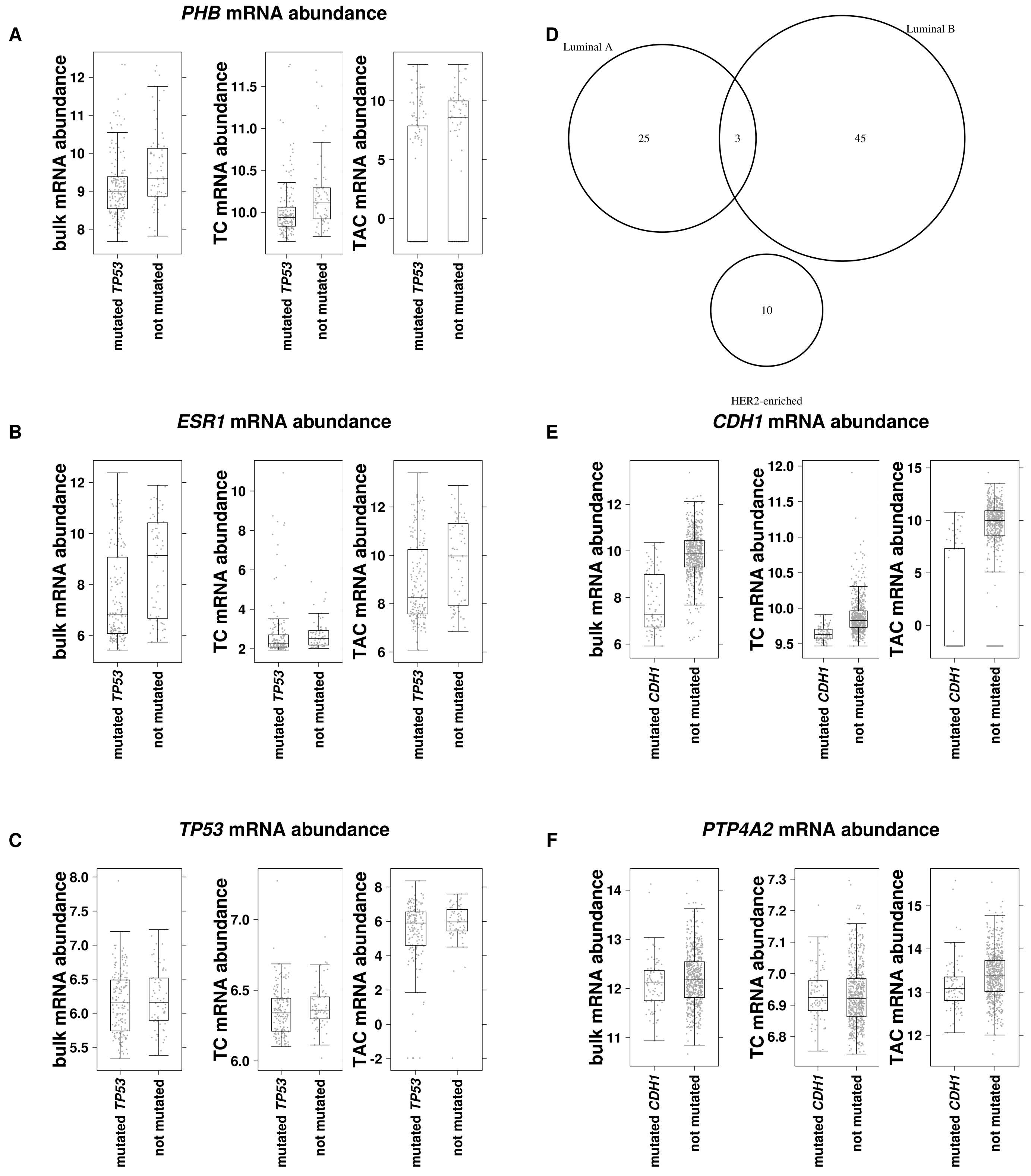

Supplementary Figure 12 | mRNA abundance differences associated with *TP53* and *CDH1*. (A-C) Bulk, TC and TAC mRNA abundance for *PHB* (A), *ESR1* (B) and *TP53* (C) for patients with and without *TP53* mutations in HER2-enriched breast cancer. Tukey boxplots are shown. (D) Comparison of the biological processes from g:Profiler[1] results for genes with differential TAC mRNA abundance associated with *TP53* mutations ($q < 0.05$, linear model with FDR adjustment) in HER2-enriched, basal-like, luminal A and luminal B breast cancers. (E-F) Bulk, TC and TAC mRNA abundance for *CDH1* (A) and *PTP4A2* (B) for patients with and without *CDH1* mutations in luminal A breast cancer. Tukey boxplots are shown.

Supplementary Tables

Supplementary Table 1 | PAM50 classification agreement. Comparison of the intrinsic molecular subtypes of breast cancer classifications using the TC and TAC mRNA abundance and the intrinsic subtype classifications from the METABRIC paper.

**Profiles**	**Number of patients classified the same as the METABRIC paper**				
	**All**	**Basal-like**	**HER2-enriched**	**Luminal A**	**Luminal B**
Deconvolved TC (one ISOpure run)	1341 (75%)	302 (92%)	196 (82%)	642 (89%)	201 (41%)
Deconvolved TC (separate runs per subtype)	1780 (100%)	330 (100%)	238 (100%)	721 (100%)	491 (100%)
Deconvolved TAC (one ISOpure run)	1443 (81%)	309 (94%)	202 (85%)	622 (86%)	310 (63%)
Deconvolved TAC (separate runs per subtype)	348 (20%)	68 (21%)	18 (8%)	179 (25%)	83 (17%)

Supplementary Table 2 | Associations with pathologist or ISOpure estimating higher purity. The results of assessing statistical associations with pathologists assigning much higher purity estimates than ISOpure (ISOpure was 0.25 less than 0.4 for moderate pathologist cellularity or 0.7 for high pathologist cellularity; 89 patients had these purity estimates) or ISOpure assigning much higher purity estimates than pathologists (ISOpure was 0.25 more than 0.7 for moderate pathologist cellularity or 0.4 for low pathologist cellularity; 23 patients had these purity estimates). The 1,668 patients that did not have these extreme differences in their purity estimates were used to assess differences in age, tumour stage and grade, patient PAM50 subtype assignment and receptor status of ERB, PgR and ER. Significant associations are shown in red in the table.

Group compared to similar	Association tested	Test	p-value	Notes
Pathologist higher	Age	t-test	1.5×10^{-3}	Older patients
Pathologist higher	Missing grade data	Proportion test	1.7×10^{-4}	More missing data
Pathologist higher	Grade (missing data removed)	X^2-test	1.5×10^{-4}	More grade 1, less grade 3
Pathologist higher	Missing stage data	Proportion test	5.3×10^{-3}	More missing data
Pathologist higher	Stage	X^2-test	0.083	
Pathologist higher	Subtype: Luminal B	Proportion test	0.050	
Pathologist higher	Subtype: Luminal A	Proportion test	3.7×10^{-3}	
Pathologist higher	Subtype: HER2-enriched	Proportion test	0.27	
Pathologist higher	Subtype: Basal	Proportion test	0.016	Less Basal patients
Pathologist higher	ERB	Proportion test	0.020	
Pathologist higher	PgR	Proportion test	0.16	
Pathologist higher	ER	Proportion test	1.2×10^{-3}	More ER+ patients
ISOpure higher	Age	t-test	0.16	
ISOpure higher	Grade (missing data removed)	X^2-test	0.71	
ISOpure higher	Grade	X^2-test	3.9×10^{-3}	More grade 3, less grade 2
ISOpure higher	Missing stage data	Proportion test	0.87	
ISOpure higher	Stage	X^2-test	0.78	
ISOpure higher	Subtype: Luminal B	Proportion test	1	
ISOpure higher	Subtype: Luminal A	Proportion test	0.016	Less Luminal A patients
ISOpure higher	Subtype: HER2-enriched	Proportion test	0.12	
ISOpure higher	Subtype: Basal	Proportion test	1.15×10^{-6}	More Basal patients
ISOpure higher	ERB	Proportion test	0.11	
ISOpure higher	PgR	Proportion test	0.29	
ISOpure higher	ER	Proportion test	1.9×10^{-4}	Less ER+ patients

Supplementary Table 3 | Number of patients. The number of patients in each of the intrinsic molecular subtypes of breast cancer and their purity distributions.

**Subtype**	**Number of patients**	**Purity range**	**Purity mean & standard deviation**
basal-like	331	0.18 - 0.88	0.62 ± 0.15
HER2-enriched	240	0.11 - 0.79	0.56 ± 0.13
luminal A	721	0.06 - 0.8	0.55 ± 0.11
luminal B	492	0.26 - 0.86	0.62 ± 0.11
not cancer (negative control)	75	0 - 0.96	0.02 ± 0.13

Supplementary Table 4 | Number of genes with TAC mRNA abundance associated with subtype. Using the conservative thresholds of absolute $\log_2$ fold change associated with the subtype > 2 and q < 1 x 10^4 (linear model), the number of genes with TAC mRNA abundance associated with PAM50 subtype classifications.

**Subtype**	**Number of genes**
basal-like	617
HER2-enriched	205
luminal A	85
luminal B	307

Supplementary Table 5 | Multi-gene signature performance. Comparison of the random multi-gene signatures.

	Bulk	TC	TAC	TC+TAC
Median random multi-gene AUC	0.608	0.627	0.573	0.630
Median random multi-gene HR	1.53	1.67	1.46	1.72
Percent of random signatures AUC > 0.65	2.6%	7.0%	0.4%	8.3%

Supplementary References

1. Reimand, J. *et al.* g:Profiler—a web server for functional interpretation of gene lists (2016 update). *Nucleic Acids Res.* gkw199 (2016). doi:10.1093/nar/gkw199
