# Supplementary material for: Landscape of transcriptomic interactions between breast cancer and its microenvironment
Source: Nat Commun. 2019 Jul 15;10:3116. doi: 10.1038/s41467-019-10929-z (PMC6629667; doi:10.1038/s41467-019-10929-z)
Supplement: Supplementary file 2 — Description of Additional Supplementary Files [file 41467_2019_10929_MOESM2_ESM.pdf]

Description of Additional Supplementary Files

File Name: Supplementary Data 1
Description: **Patient ISOpure purity estimates.** The ISOpure purity estimates for each patient.

File Name: Supplementary Data 2
Description: **Significant pathway and Gene Ontology results for genes differentiating aggressive TAC subtype and univariate survival associations.**

(A) g:Profiler results for genes whose TAC mRNA abundance were differentially expressed in the aggressive TAC subtype compared to the other three subtypes. (B) g:Profiler results for genes whose TC mRNA abundance were univariately associated with prognosis but bulk and TAC mRNA abundance were not associated with prognosis. (C) g:Profiler results for genes whose TC mRNA abundance were univariately associated with prognosis but bulk and TAC mRNA abundance were not associated with prognosis.

File Name: Supplementary Data 3
Description: **Gene-wise Cox modelling results.**

(A) Genes with significant univariate TC Cox modelling and significant univariate TAC Cox modelling. TC and TAC HR, HR 95% confidence interval, Wald p-value, FDR adjusted Wald q-value and Cox proportion hazards p-value of univariate Cox modelling for 7 genes whose TC and TAC mRNA abundance were significantly associated with prognosis (green or black in **Figure 3D**). (B) Genes with significant univariate Cox modelling on the individual subtypes. TC and TAC HR, HR 95% confidence interval, Wald p-value, FDR adjusted Wald q-value and Cox proportion hazards p-value of univariate Cox modelling for genes that significantly associate with prognosis on the patients from a single subtype. The same dichotomization thresholds were used as on all patients together. (C) Genes with significant TC-TAC interactions. The HR, Wald p-value, FDR adjusted Wald q-value and Cox proportion hazards p-value of the interaction term of Cox modelling survival ~ TC + TAC + TC x TAC for the genes that had a significant interaction term.

File Name: Supplementary Data 4
Description: **Mutated genes differential mRNA abundance associations.**

(A) Mutated genes cis differential mRNA results. The $\log_2$ fold change and adjusted p-values from limma analysis looking at the mutated gene's mRNA abundance. (B) g:Profiler results for genes with differential TAC mRNA abundance associated with TP53 mutations ($q < 0.05$, linear model with FDR adjustment) in HER2-enriched breast cancers. (C) g:Profiler results for genes with differential TAC mRNA abundance

associated with TP53 mutations ($q < 0.05$, linear model with FDR adjustment) in luminal A breast cancers. (D) g:Profiler results for genes with differential TAC mRNA abundance associated with TP53 mutations ($q < 0.05$) in luminal B breast cancers. (E) g:Profiler results for genes with differential mRNA abundance associated with CDH1 mutations ($q < 0.05$, linear model with FDR adjustment) in luminal A breast cancers.

File Name: Supplementary Data 5
Description: **METABRIC TC mRNA profiles.** The TC mRNA abundance profiles in $\log_2$ space for the METABRIC patients. Patients are columns and genes, specified by Entrez IDs, are rows.

File Name: Supplementary Data 6
Description: **METABRIC TAC mRNA profiles.** The TAC mRNA abundance profiles in $\log_2$ space for the METABRIC patients. Patients are columns and genes, specified by Entrez IDs, are rows.

File Name: Supplementary Data 7
Description: **TCGA TC mRNA profiles.** The TC mRNA abundance profiles in $\log_2$ space for the TCGA patients. Patients are columns and genes, specified by Entrez IDs, are rows.

File Name: Supplementary Data 8
Description: **TCGA TAC mRNA profiles.** The TAC mRNA abundance profiles in $\log_2$ space for the TCGA patients. Patients are columns and genes, specified by Entrez IDs, are rows.

File Name: Supplementary Code 1
Description: **Analysis code.** Knitr documentation for R analysis and figures.
