# Supplementary material for: Landscape of transcriptomic interactions between breast cancer and its microenvironment
Source: Nat Commun. 2019 Jul 15;10:3116. doi: 10.1038/s41467-019-10929-z (PMC6629667; doi:10.1038/s41467-019-10929-z)

Supplementary Code for "Landscape of transcriptomic interactions between breast cancer and its microenvironment"

Fox *et al.*

Contents

Page

1 Example for running ISOpure using R . . . 3

2 Figure 1 B & C - ISOpure approach comparison of mRNA abundance . . . 5

3 Figure 1D - Breast Cancer Receptors . . . 8

4 Figure 1E - *CAV1* TAC prognosis . . . 11

5 Supplementary Figure 1 - TCGA va Metabric comparison of mRNA abundance . . . 13

6 Supplementary Figure 3A-B - Purity Estimates . . . 17

7 Supplementary Figure 3C - Associations with Pathologist or ISOpure estimating higher purity . . . 20

8 Running Clustering Patients . . . 28

9 Figure 2A - PAM50 gene variation . . . 30

10 Figure 2 - Clustering highly variant genes to recapitulate breast cancer subtypes . . . 33

11 Supplementary Figure 4A-C - Clustered PAM50 mRNA abundance . . . 42

12 Supplementary Figure 4D - *BCL2* mRNA abundance per subtype . . . 46

13 Supplmentary Figure 5A - Genes with TAC mRNA abundance associated with subtypes . . . 49

14 Supplementary Figure 5B-F - Subtype differences between TC-TAC correlations . . . 54

15 Supplementary Figure 5G - Subtype labeling for profile clusters . . . 60

16 Running Univariate Survival Analysis . . . 64

17 Figure 3 - Clustering highly variant genes to recapitulate breast cancer subtypes . . . 67

18 Supplementary Figure 6A-B - p-value sensitivity for univariate analysis . . . 77

19 Supplementary Figure 6C-E - Univariate Cox modelling Hazard ratio correlation . . . 81

20 Supplementary Figure 6F - mRNA distributions . . . 85

21 Supplementary Figure 7 - Correlation of univariate Cox modelling hazard ratio between subtype specific analysis and all patients 87

22 Running TC-TAC Interaction Survival Analysis 94

23 Figure 4 - TC-TAC gene interactions 98

24 Supplementary Figure 8 - *FHL3*, *SDAD1*, *CRIPAK* prognosis 111

25 Supplementary Figure 9 - *FHL3*, *SDAD1*, *CRIPAK* prognosis per subtype 115

26 Create Random Multi-gene Biomarkers 120

27 Run Test Set Performance Random Multi-gene Biomarkers 126

28 Figure 5 - Biomarker generation 132

29 Supplementary Figure 10 - Gene contribution to multi-gene biomarkers 139

30 Figure 6 - Differential mRNA abundance associated with mutated genes 144

31 Supplementary Figure 11 - Differential mRNA abundance validation 157

32 Supplementary Figure 12A,B,C,E,F - mRNA abundance differences associated with *TP53* and *CDH1* . 164

33 Supplmentary Figure 12D - Overlap between biological processes associated *TP53* mutations 168

1 Example for running ISOpure using R

Set up the environment

```
library(ISOpureR);
library(yaml);
dataset.name <- 'Metabric';
```

Yaml file name

```
yaml.file <- paste0(dataset.name,'.yaml');
```

Read yaml with file information

```
dataset.files <- yaml.load_file(yaml.file);
```

Load clinical annotation

```
patient.anno <- read.table(dataset.files$clinical.annotation.file,sep='\t',header=TRUE);
```

Load panel of normal mrna abundance profiles

```
normal.mrna.panel <- read.table(
  dataset.files$normal.panel.bulk.mrna.abundance.file,
  header=FALSE,
  sep='\t'
  );
```

Convert the mRNA abundance in normal space

```
normal.mrna.panel <- 2^as.matrix(normal.mrna.panel);
```

Load panel of bulk sample mRNA abundance

```
bulk.mrna <- read.table(
  dataset.files$bulk.mrna.abundance.file,
  header=FALSE,
  sep='\t'
  );
```

Convert the mRNA abundance in normal space

```
bulk.mrna <- 2^as.matrix(bulk.mrna);
```

Run each subtype separately through isopure

```
purity.estimates <- NULL;
tc.mrna <- NULL;
for(subtype in c('Basal','Her2','LumA', 'LumB')) {
  # run isopure step 1
  set.seed(374);
```

```
  ISOpureS1model <- ISOpure.step1.CPE(
    bulk.mrna[,patient.anno$patient_id[patient.anno$Pam50Subtype == subtype]],
    normal.mrna.panel
    );
  # purity estimates
  purity.estimates <- c(purity.estimates,ISOpureS1model$alphapurities);
  # run isopure step 2
  set.seed(719);
  ISOpureS2model <- ISOpure.step2.PPE(
    bulk.mrna,
    normal.mrna.panel,
    ISOpureS1model
    );
  # tumour mRNA abundance estimates
  tc.mrna.normal.space <- ISOpureS2model$cc_cancerprofiles;
  # convert to log space
  tc.mrna <- cbind(tc.mrna,round(log2(tc.mrna.normal.space),digits=5));
  }
```

Write results to file

```
write.table(
  matrix(purity.estimates,nrow=length(purity.estimates)),
  file=dataset.files$isopure.purity.file,
  sep='\t',
  col.names=FALSE,
  row.names=FALSE,
  quote=FALSE
  );
write.table(
  tc.mrna,
  file=dataset.files$tc.mrna.file,
  quote=FALSE,
  row.names=TRUE,
  col.names=TRUE,
  sep='\t'
  );
```

Calculate tumour adjacent cell mRNA abundance estimates

```
tac.mrna.normal.space <- ISOpure.calculate.tac(
  as.matrix(bulk.mrna),
  as.matrix(tc.mrna.normal.space),
  as.numeric(purity.estimates)
  );
```

Convert to log space

```
tac.mrna <- round(log2(tac.mrna.normal.space),digits=5);
```

Write the tumour adjacent cell mRNA abundance to file

```
write.table(
  tac.mrna,
  file=dataset.files$tac.mrna.file,
  quote=FALSE,
  row.names=TRUE,
  col.names=TRUE,
  sep='\t'
  );
```

2 Figure 1 B & C - ISOpure approach comparison of mRNA abundance

Set up the environment

```
library(BoutrosLab.plotting.general);
library(yaml);
dataset.name <- 'Metabric';
profile.types <- c('tc','tac');
```

Load the data corresponding to each dataset

```
dataset.files <- list();
patient.anno <- list();
rna.data.matrix <- list();
```

Read yaml with file information

```
dataset.files <- yaml.load_file(paste0(dataset.name,'.yaml'));
```

Load clinical annotation

```
patient.anno <- read.table(
  dataset.files$clinical.annotation.file,
  sep='\t',
  header=TRUE
  );
```

Remove Normal-like patients

```
patient.anno <- patient.anno[which(!patient.anno$subtype %in% c('Normal-like','unknown')),];
```

Load the mRNA abundance results from ISOpure run per subtype

```
rna.data.matrix$separate <- list();
for(profile.type in profile.types) {
  rna.data.matrix$separate[[profile.type]] <- read.table(
    dataset.files[[paste0(profile.type,'.mrna.abundance.file')]],
    header=TRUE,
    sep='\t'
    );
```

```
  rna.data.matrix$separate[[profile.type]] <-
    rna.data.matrix$separate[[profile.type]][,intersect(
      as.character(patient.anno$patient_id),
      colnames(rna.data.matrix$separate[[profile.type]])
      )];
  }
```

Load the mRNA abundance results from ISOpure run together

```
rna.data.matrix$together <- list();
for(profile.type in profile.types) {
  rna.data.matrix$together[[profile.type]] <- read.table(
    dataset.files[[paste0(profile.type,'.mrna.abundance.isopure.together.file')]],
    header=TRUE,
    sep='\t'
    );
  rna.data.matrix$together[[profile.type]] <-
    rna.data.matrix$together[[profile.type]][,intersect(
      as.character(patient.anno$patient_id),
      colnames(rna.data.matrix$together[[profile.type]])
      )];
  }
```

Compare median mRNA abundance between ISOpure approaches for all genes found in both

```
for(profile.type in profile.types) {
  common.genes <- intersect(
    rownames(rna.data.matrix$together[[profile.type]]),
    rownames(rna.data.matrix$separate[[profile.type]])
    );
  together.medians <- apply(rna.data.matrix$together[[profile.type]][common.genes,],1,median);
  separate.medians <- apply(rna.data.matrix$separate[[profile.type]][common.genes,],1,median);
  create.hexbinplot(
    formula=y ~ x,
    data=data.frame(
      x=together.medians,
      y=separate.medians
      ),
    filename = paste0('./figure1bc_',profile.type,'.pdf'),
    resolution=100,
    xlab.label=bquote('Subtypes together median'),
    ylab.label=bquote('Subtypes separate median'),
    add.xyline=FALSE,
    abline.front=TRUE,
    abline.h=3.5,
    abline.v=6.5,
    abline.lty=2,
    add.axes=FALSE,
    aspect=1,
    width=9,
```

```
    legend = list(
      inside = list(
        fun = draw.key,
        args = list(
          key = get.corr.key(
            x = together.medians,
            y = separate.medians,
            label.items = c('spearman','spearman.p'),
            alpha.background = 0,
            key.cex = 2.4
            )
          ),
          x = 0.44,
          y = 0.22,
          corner = c(0,1)
        )
      )
    );
  }
```

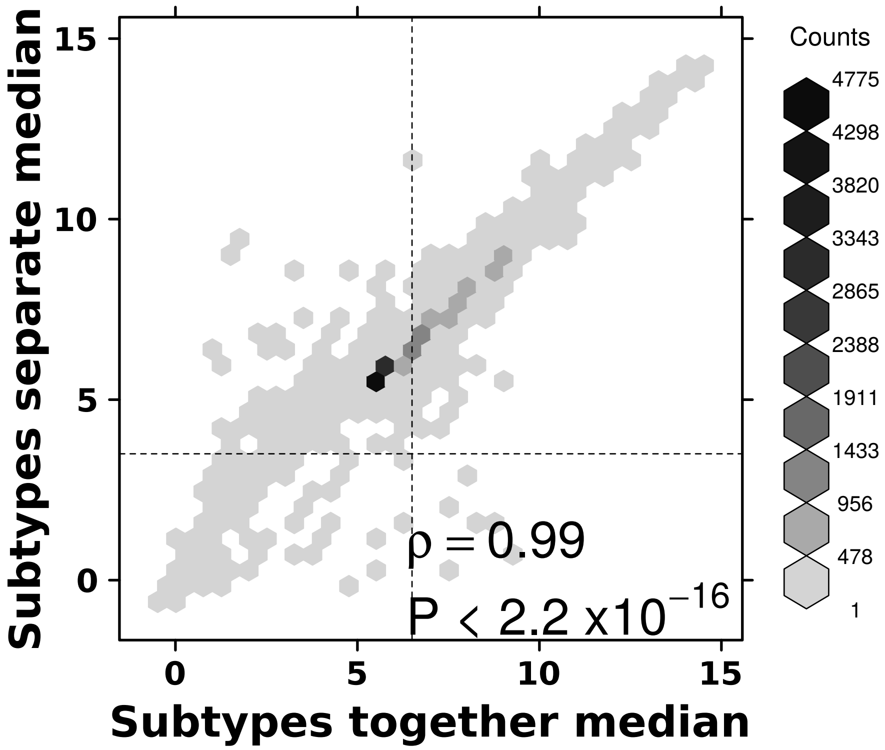

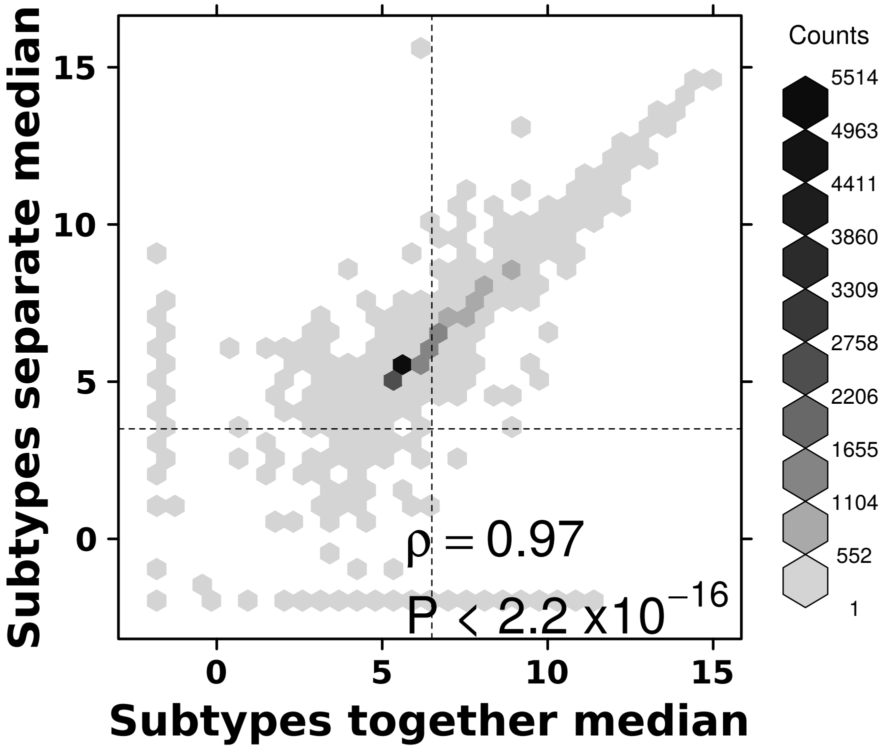

3 Figure 1D - Breast Cancer Receptors

Set up the environment

```
library(BoutrosLab.plotting.general);
library(yaml);
dataset.name <- 'Metabric';
```

Yaml file name

```
yaml.file <- paste0(dataset.name,'.yaml');
```

Read yaml with file information

```
dataset.files <- yaml.load_file(yaml.file);
```

Load clinical annotation

```
patient.anno <- read.table(dataset.files$clinical.annotation.file,sep='\t',header=TRUE);
```

Remove Normal-like patients

```
patient.anno <- patient.anno[which(!patient.anno$subtype %in% c('Normal-like','unknown')),];
```

Load mRNA abundance profiles

```
profile.types <- c('bulk','tc','tac');
rna.data.matrix <- list();
rna.normals <- list();
for(profile.type in profile.types) {
  rna.data.matrix[[profile.type]] <- read.table(
    dataset.files[[paste0(profile.type,'.mrna.abundance.file')]],
    header=TRUE,
    sep='\t'
    );
  rna.normals[[profile.type]] <- read.table(
    dataset.files[[paste0('normal.panel.',profile.type,'.mrna.abundance.file')]],
    header=TRUE,
    sep='\t'
    );
  rna.normals[[profile.type]] <- rna.normals[[profile.type]][c('2099_at','5241_at','2064_at'),];
  rownames(rna.normals[[profile.type]]) <- c('ESR1','PGR','HER2');
  }
```

Subtype colour scheme

```
subtype.colours <- c('red','pink','dodgerblue3','lightskyblue2','forestgreen');
names(subtype.colours) <- c('Basal','Her2','LumA','LumB','Normal');
subtype.colours <- subtype.colours[setdiff(names(subtype.colours),'Normal')];
```

Add the receptor gene mRNA abundance to the table with subtype information

```
for(profile.type in profile.types) {
  new.col <- t(rna.data.matrix[[profile.type]]['2099_at',]);
  colnames(new.col) <- paste0('ESR1.mRNA.',profile.type);
  patient.anno[,paste0('ESR1.mRNA.',profile.type)] <- new.col;
  new.col <- t(rna.data.matrix[[profile.type]]['5241_at',]);
  colnames(new.col) <- paste0('PGR.mRNA.',profile.type);
  patient.anno[,paste0('PGR.mRNA.',profile.type)] <- new.col;
  new.col <- t(rna.data.matrix[[profile.type]]['2064_at',]);
  colnames(new.col) <- paste0('HER2.mRNA.',profile.type);
  patient.anno[,paste0('HER2.mRNA.',profile.type)] <- new.col;
  }
```

Plot the mRNA abundance distributions

```
boxplots <- list();
for(gene in c('HER2','PGR','ESR1')) {
  for(profile.type in c('bulk','tc','tac')) {
    boxplots <- append(boxplots,list(create.boxplot(
      mRNA ~ subtype,
      data.frame(
        mRNA = scale(as.numeric(c(
          rna.normals[[profile.type]][gene,],
          patient.anno[
            patient.anno$Pam50Subtype %in% names(subtype.colours),
```

```
          paste0(gene,'.mRNA.',profile.type)
          ]
        ))),
      subtype = factor(c(
        rep(0,ncol(rna.normals[[profile.type]])),
        patient.anno[
          patient.anno$Pam50Subtype %in% names(subtype.colours),
          'Pam50Subtype'
          ]
        ))
      ),
    add.stripplot = TRUE,
    points.col = c(
      rep('gray',ncol(rna.normals[[profile.type]])),
      subtype.colours[sort(names(subtype.colours))][
        as.numeric(factor(patient.anno[patient.anno$Pam50Subtype %in% names(subtype.colours),
        'Pam50Subtype'
        ]))
      ]),
    use.legacy.settings=TRUE,
    lwd=1.8
    )));
  }
}
```

Compile the boxplots into the final figure

```
create.multiplot(
  boxplots,
  filename = './figure1d.pdf',
  resolution = 200,
  plot.layout=c(3,3),
  panel.widths=c(5,5,5),
  width=11,
  height=8,
  y.relation='same',
  x.relation='free',
  xaxis.labels=NULL,
  ylimits = c(-4.5,4.5),
  xlab.label = c('   Bulk','      TC','    TAC'),
  ylab.label = c('ESR1','PGR','ERBB2'),
  xat=list(1:5,1:5,1:5,c(),c(),c(),c(),c(),c()),
  y.spacing=-0.5,
  right.padding = 30,
  key = list(
    points = list(
      pch = 19,
      col = c('gray','white',subtype.colours[sort(names(subtype.colours))]),
      cex=2
      ),
```

```
    text = list(
      lab = c('Non-malignant','','Basal-like','HER2-enriched','Luminal A','Luminal B'),
      cex=2
      ),
    x = 1,
    y = 0.6,
    padding.text = 2
    ),
  use.legacy.settings=TRUE
  );
```

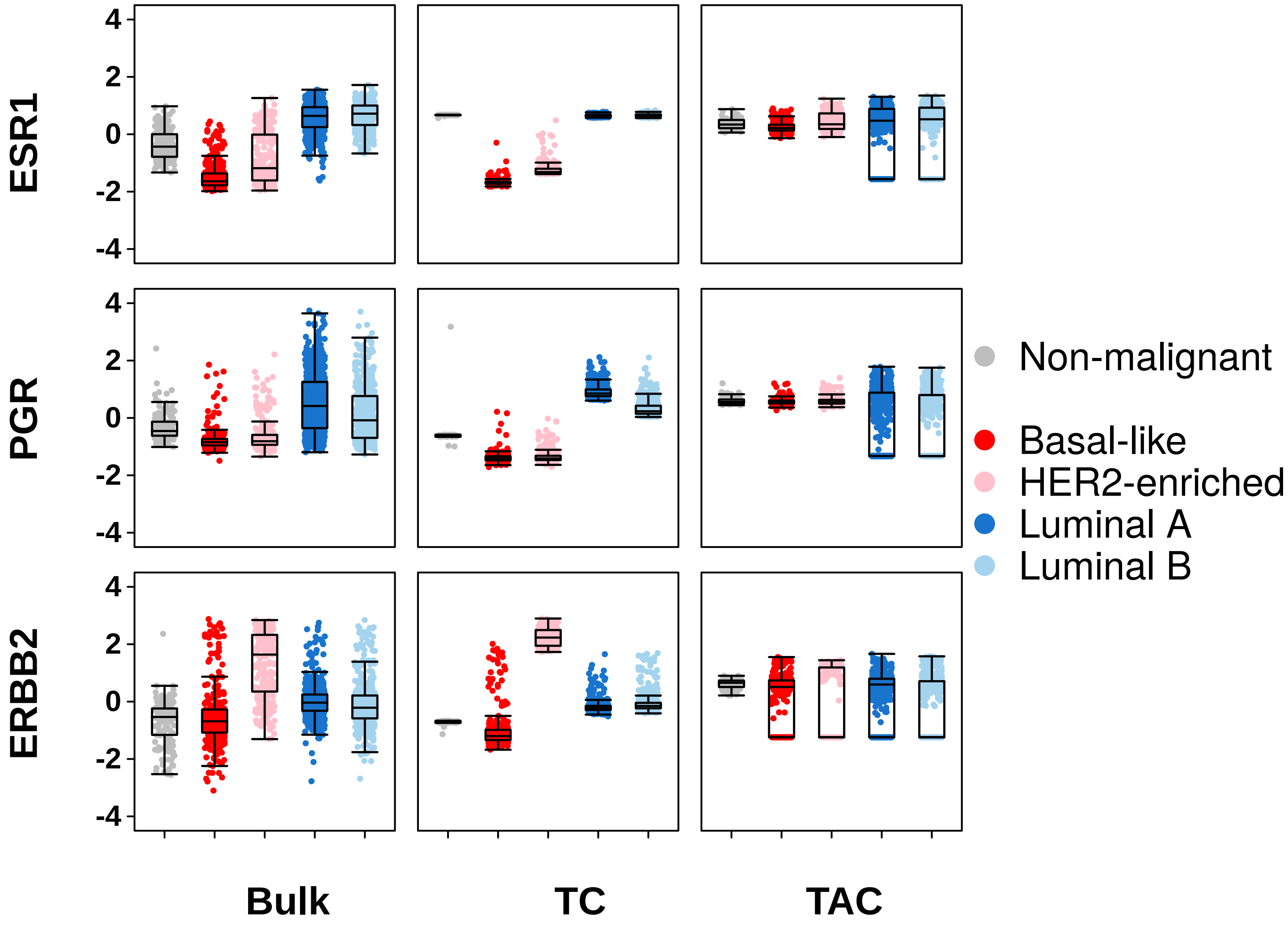

4 Figure 1E - *CAV1* TAC prognosis

Set up the environment

```
library(BoutrosLab.plotting.survival);
library(yaml);
dataset.name <- 'Metabric';
```

Yaml file name

```
yaml.file <- paste0(dataset.name,'.yaml');
```

Read yaml with file information

```
dataset.files <- yaml.load_file(yaml.file);
```

Load mRNA abundance profiles

```
rna.data.matrix <- list();
rna.data.matrix$tac <- read.table(dataset.files$tac.mrna.abundance.file,header=TRUE,sep='\t');
```

Load clinical annotation

```
patient.anno <- read.table(dataset.files$clinical.annotation.file,sep='\t',header=TRUE);
```

Remove Normal-like patients

```
patient.anno <- patient.anno[which(!patient.anno$subtype %in% c('Normal-like','unknown')),];
```

Collect survival data

```
patient.anno <- patient.anno[!is.na(patient.anno$TimeToEvent),];
patient.anno <- patient.anno[!is.na(patient.anno$Event),];
time.to.event <- patient.anno$TimeToEvent;
censoring <- patient.anno$Event;
max.followup.time <- 16;
censoring[which(time.to.event > max.followup.time)] <- 0;
time.to.event[which(time.to.event > max.followup.time)] <- max.followup.time;
time.to.event[time.to.event < 0] <- 0;
```

Specify gene for analysis

```
gene.id <- '857_at';
gene.sym <- 'CAV1';
```

Determine the mRNA abundance threshold to divide patients

```
i <- which(rownames(rna.data.matrix[[1]]) == gene.id);
tac.split <- median(as.numeric(rna.data.matrix$tac[i,]));
if(tac.split < 6.5) {
  tac.split <- 6.5;
  }
```

Divide patients into low and high mRNA abundance

```
patient.groups <- as.numeric(rna.data.matrix$tac[i,rownames(patient.anno)]) >= tac.split;
survival.result <- create.km.plot(
  survival.object = Surv(time.to.event, censoring),
  patient.groups = factor(patient.groups,levels=c(TRUE,FALSE)),
  filename = './figure1e.pdf',
```

```
  xlab.label = 'Time (Years)',
  ylab.label = 'Survival',
  statistical.method = NA,
  ph.assumption.check='warning.and.plot',
  resolution = 120,
  show.risktable = FALSE,
  main='CAV1 TAC mRNA Abundance',
  main.cex=2,
  line.colours=rev(c('gray60','gold')),
  key.groups.labels=c('high','low'),
  risk.labels=c('high','low'),
  xlimits=c(0,16),
  key.stats.cex=2.1,
  key.groups.title.cex=2.1,
  key.groups.cex=2.1
  );
```

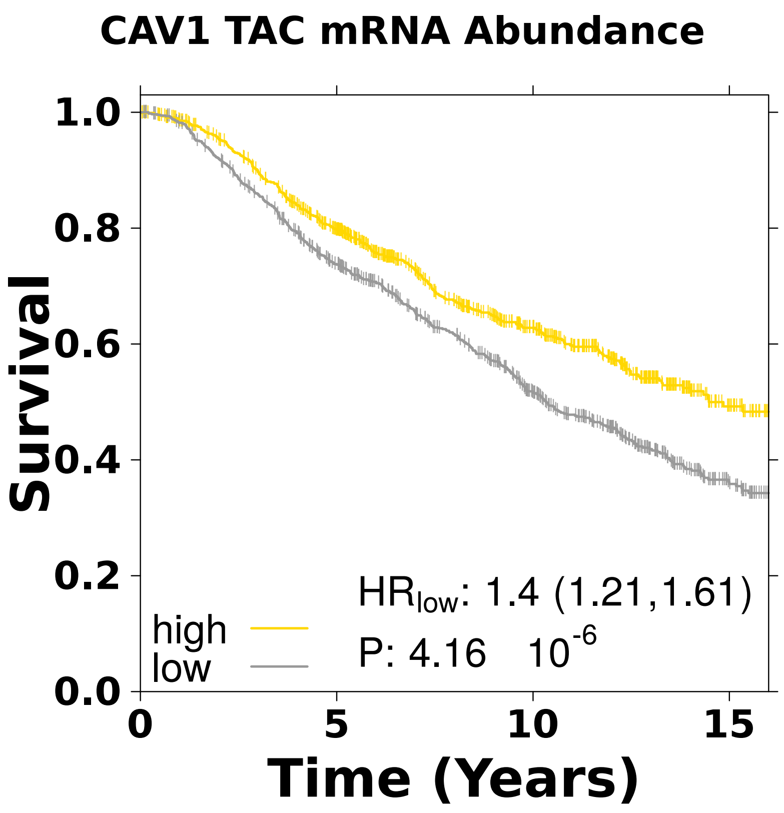

5 Supplementary Figure 1 - TCGA va Metabric comparison of mRNA abundance

Set up the environment

```
library(BoutrosLab.plotting.general);
library(yaml);
dataset.names <- c('Metabric','BRCA');
profile.types <- c('bulk','tc','tac');
```

Load the data corresponding to each dataset

```
dataset.files <- list();
patient.anno <- list();
rna.data.matrix <- list();
for(dataset.name in dataset.names) {
  # read yaml with file information
  dataset.files[[dataset.name]] <- yaml.load_file(paste0(dataset.name,'.yaml'));
  # load clinical annotation
  patient.anno[[dataset.name]] <- read.table(
    dataset.files[[dataset.name]]$clinical.annotation.file,
    sep='\t',
    header=TRUE
    );
  # remove Normal-like patients
  patient.anno[[dataset.name]] <-
    patient.anno[[dataset.name]][
    which(!patient.anno[[dataset.name]]$subtype %in% c('Normal-like','unknown')),
    ];
  # load mRNA abundance results
  rna.data.matrix[[dataset.name]] <- list();
  for(profile.type in profile.types) {
    rna.data.matrix[[dataset.name]][[profile.type]] <- read.table(
      dataset.files[[dataset.name]][[paste0(profile.type,'.mrna.abundance.file')]],
      header=TRUE,
      sep='\t'
      );
    rna.data.matrix[[dataset.name]][[profile.type]] <-
      rna.data.matrix[[dataset.name]][[profile.type]][,intersect(
        colnames(rna.data.matrix[[dataset.name]][[profile.type]]),
        patient.anno[[dataset.name]]$patient_id
        )];
    }
  }
```

Compare median mRNA abundance between Metabric and tcga for all genes found in both

```
for(profile.type in profile.types) {
  common.genes <- intersect(
    rownames(rna.data.matrix$Metabric[[profile.type]]),
    rownames(rna.data.matrix$BRCA[[profile.type]])
    );
  for(subtype in c('Basal-like','HER2-enriched','Luminal A','Luminal B')) {
    metabric.medians <- apply(rna.data.matrix$Metabric[[profile.type]][common.genes,patient.anno$Meta
    brca.medians <- apply(rna.data.matrix$BRCA[[profile.type]][common.genes,patient.anno$BRCA$subtype
    create.hexbinplot(
      formula=y ~ x,
      data=data.frame(
        x=metabric.medians,
        y=brca.medians
```

```
      ),
    filename = paste0('./sfigure01_',profile.type,'_',gsub('-','_',gsub(' ','_',subtype)),'.pdf'),
    resolution=100,
    xlab.label=bquote('METABRIC median'),
    ylab.label=bquote('TCGA median'),
    add.xyline=FALSE,
    abline.front=TRUE,
    abline.h=3.5,
    abline.v=6.5,
    abline.lty=2,
    add.axes=FALSE,
    aspect=1,
    width=9,
    legend = list(
      inside = list(
        fun = draw.key,
        args = list(
          key = get.corr.key(
            x = metabric.medians,
            y = brca.medians,
            label.items = c('spearman','spearman.p'),
            alpha.background = 0,
            key.cex = 2.4
            )
          ),
          x = 0.44,
          y = 0.22,
          corner = c(0,1)
        )
      )
    );
  }
}
```

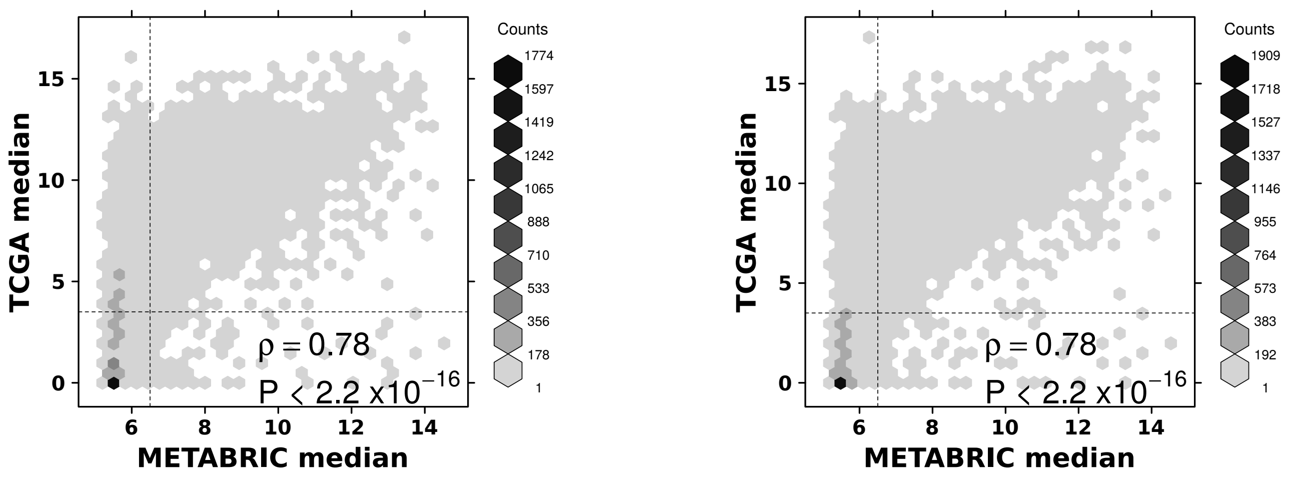

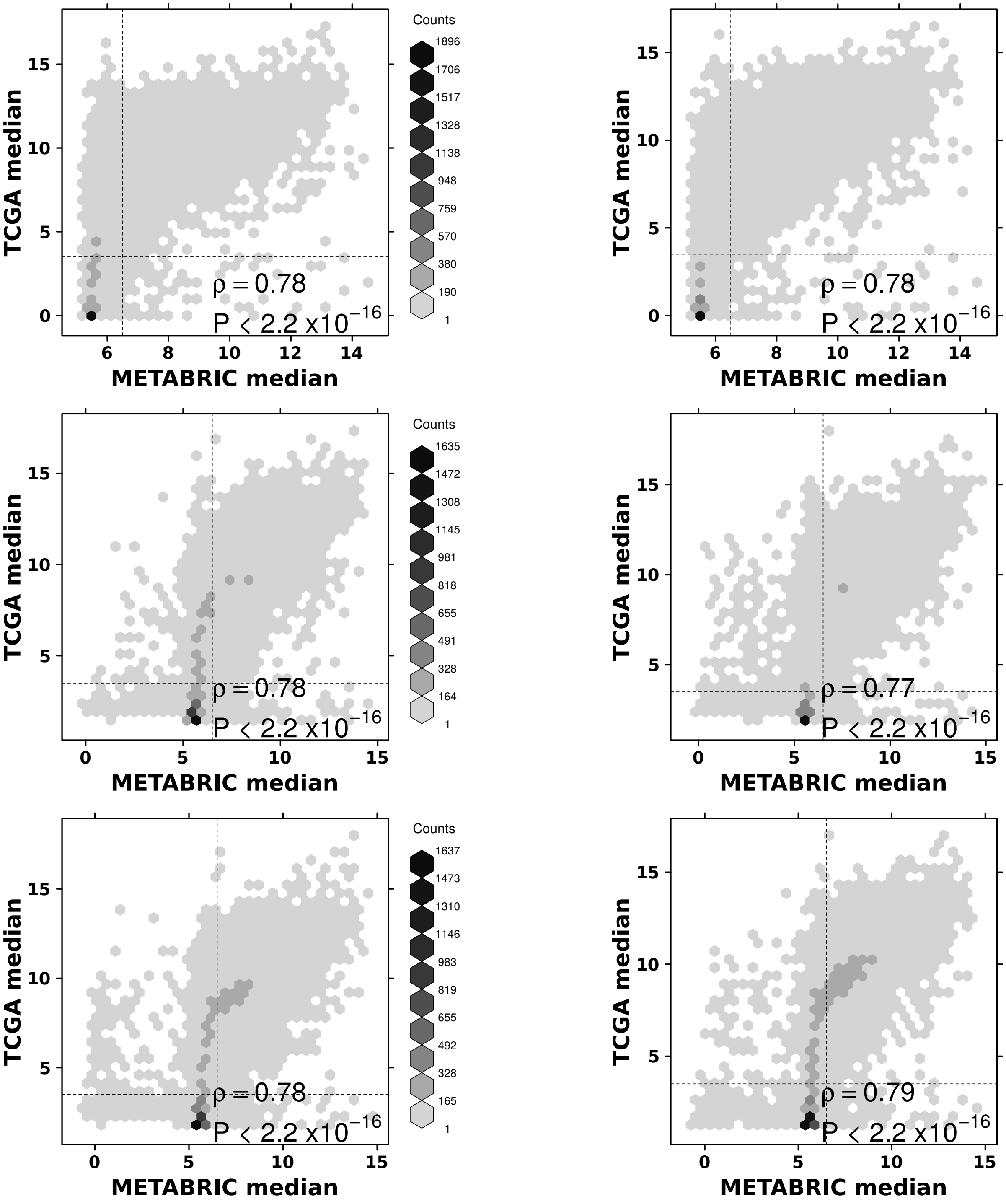

TCGA median
METABRIC median
Counts
1896
1706
1517
1328
1138
948
759
570
380
190
1
$\rho = 0.78$
$P < 2.2 \times 10^{-16}$
TCGA median
METABRIC median
Counts
1964
1768
1571
1375
1179
982
786
590
394
197
1
$\rho = 0.78$
$P < 2.2 \times 10^{-16}$
TCGA median
METABRIC median
Counts
1635
1472
1308
1145
981
818
655
491
328
164
1
$\rho = 0.78$
$P < 2.2 \times 10^{-16}$
TCGA median
METABRIC median
Counts
2082
1874
1666
1458
1250
1042
833
625
417
209
1
$\rho = 0.77$
$P < 2.2 \times 10^{-16}$
TCGA median
METABRIC median
Counts
1637
1473
1310
1146
983
819
655
492
328
165
1
$\rho = 0.78$
$P < 2.2 \times 10^{-16}$
TCGA median
METABRIC median
Counts
1385
1247
1108
970
831
693
555
416
278
139
1
$\rho = 0.79$
$P < 2.2 \times 10^{-16}$

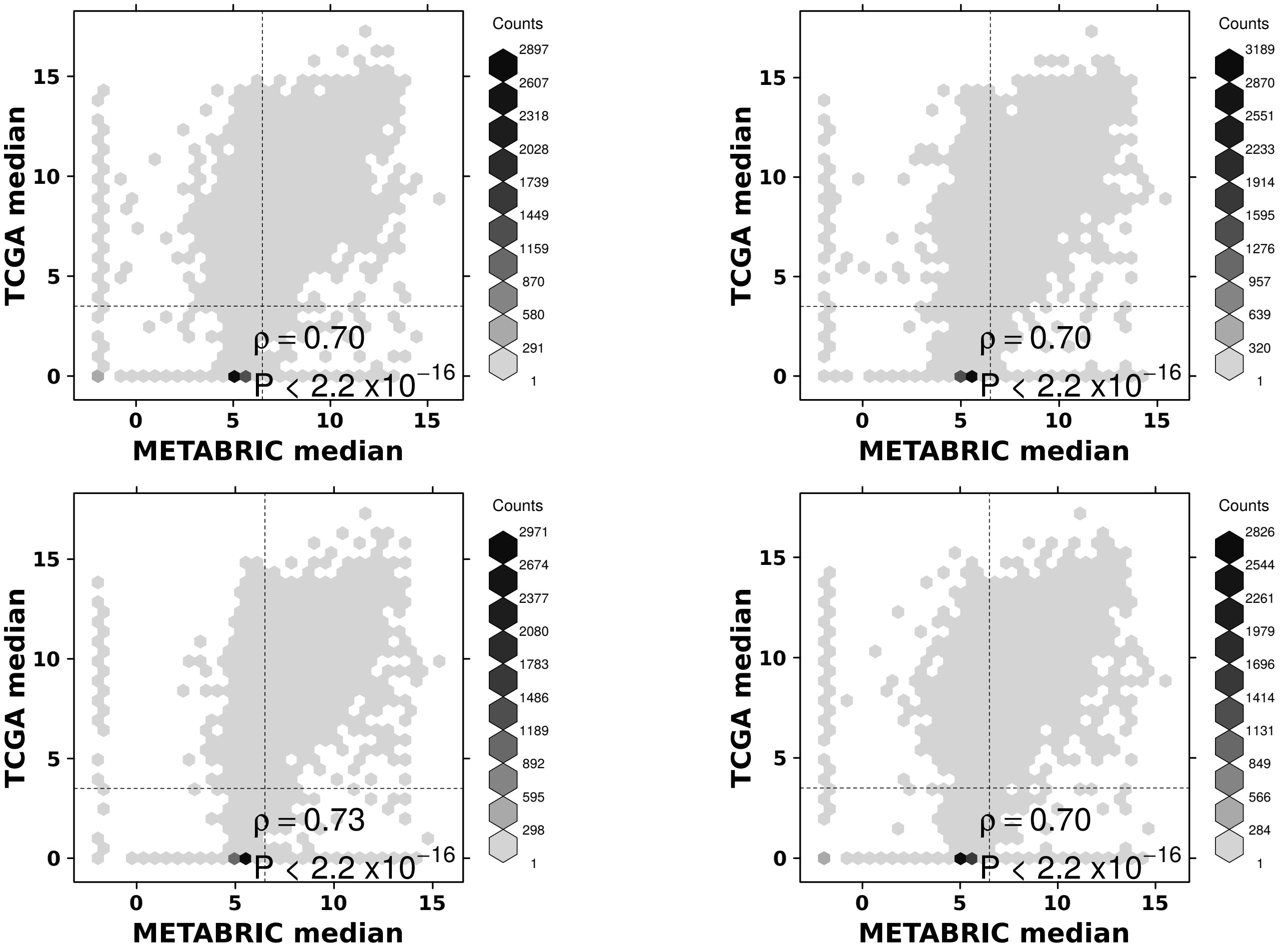

6 Supplementary Figure 3A-B - Purity Estimates

Set up the environment

```
library(BoutrosLab.plotting.general);
library(yaml);
dataset.name <- 'Metabric';
```

Yaml file name

```
yaml.file <- paste0(dataset.name,'.yaml');
```

Read yaml with file information

```
dataset.files <- yaml.load_file(yaml.file);
```

Load clinical annotation

```
patient.anno <- read.table(dataset.files$clinical.annotation.file,sep='\t',header=TRUE);
patient.anno$cellularity <- factor(
  as.character(patient.anno$cellularity),
  levels=c('low','moderate','high','undef')
  );
patient.anno$cellularity[is.na(patient.anno$cellularity)] <- 'undef';
```

Load ISOpure purity estimates

```
purity.estimates <- read.table(dataset.files$isopure.purity.estimate.file,header=TRUE);
patient.anno[rownames(purity.estimates),'purity'] <- purity.estimates$alpha.purity;
patient.anno <- patient.anno[!is.na(patient.anno$purity),];
```

Subtype colour scheme

```
subtype.colours <- c('red','pink','dodgerblue3','lightskyblue2');
names(subtype.colours) <- c('Basal','Her2','LumA','LumB');
```

Normal sample (negative control)

```
normal.sample.purity <- read.table(
  dataset.files$normal.panel.isopure.purity.estimate.file,
  header=FALSE,
  sep='\t'
  );
```

Create the pathologist vs ISOpure estimate plot

```
create.boxplot(
  purity ~ cellularity,
  patient.anno[which(patient.anno$Pam50Subtype %in% c('Basal','Her2','LumA','LumB')),],
  filename = './sfigure3a.pdf',
  ylab.label='ISOpure purity estimate',
  xlab.label='pathologist cellularity estimate',
  xaxis.rot=90,
  resolution=200,
  add.stripplot=TRUE,
  points.col=subtype.colours[patient.anno[
    which(patient.anno$Pam50Subtype %in% c('Basal','Her2','LumA','LumB')),
    'Pam50Subtype'
    ]],
  ylimits=c(0,1),
  lwd=2,
  points.alpha=0.8,
  yat=seq(0,1,0.2),
  xaxis.lab=c('\nlow\n(< 0.4)','\nmoderate\n(0.4 - 0.7)','\nhigh\n(> 0.7)',' undefined'),
  abline.h=c(0.4,0.7),
  abline.lty=2,
  use.legacy.settings=TRUE,
  right.padding = 25,
  width=7.5,
  key = list(
    points = list(
      pch = 19,
      col = subtype.colours[sort(names(subtype.colours))],
      cex=1.5
      ),
```

```
    text = list(
      lab = c('Basal-like','HER2-enriched','Luminal A','Luminal B'),
      cex=1.5
      ),
    x = 1.05,
    y = 0.7,
    padding.text = 2
    )
  );
```

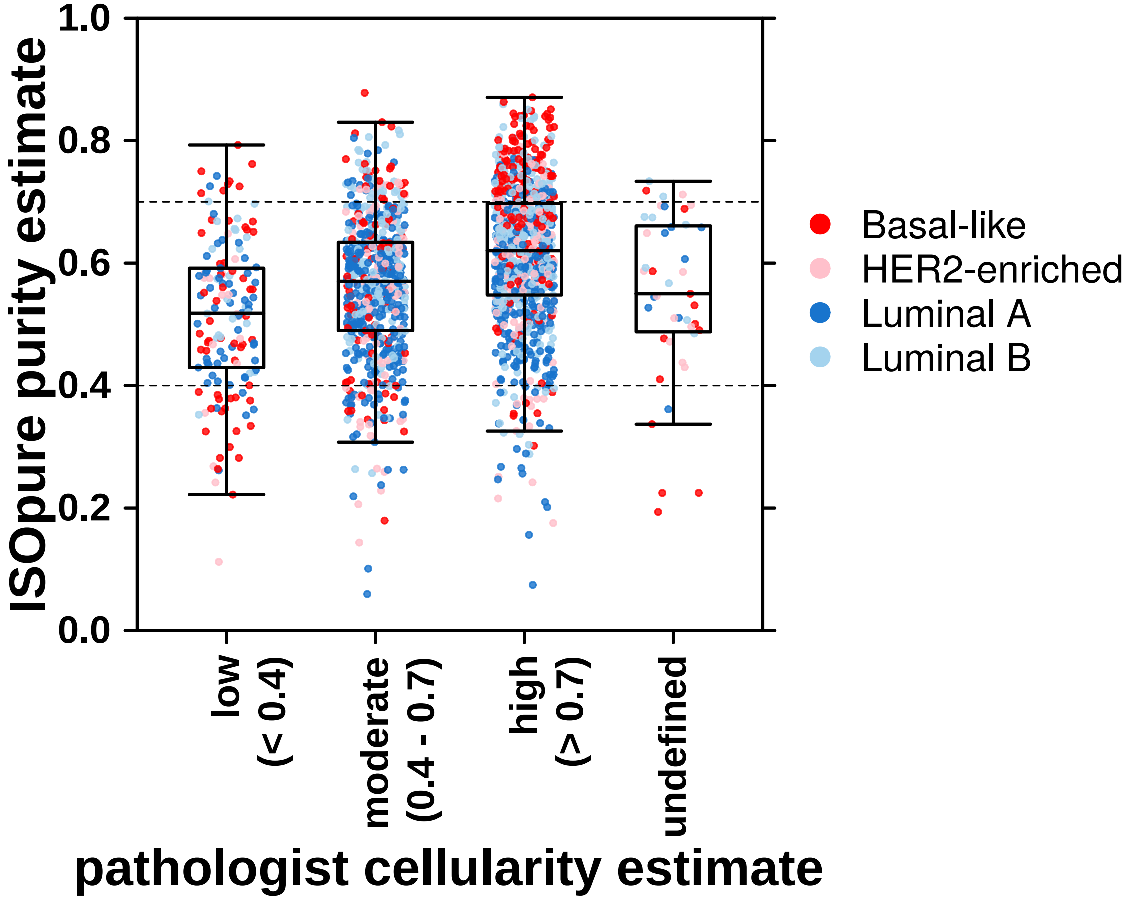

Create the purity estimates by subtype plot

```
patient.anno <- patient.anno[which(!patient.anno$subtype %in% c('Normal-like','unknown')),];
create.boxplot(
  purity ~ subtype,
  data.frame(
    purity = c(patient.anno$purity,as.numeric(normal.sample.purity[,1])),
    subtype = factor(c(
      as.character(patient.anno$subtype),
      rep('none (not cancer)',nrow(normal.sample.purity))
      ))
    ),
  filename = './sfigure3b.pdf',
  ylab.label='purity estimate',
  xlab.label='subtype',
```

```
  xaxis.rot=90,
  resolution=200,
  add.stripplot=TRUE,
  points.col=c(subtype.colours[as.numeric(patient.anno$subtype)],rep('gray50',nrow(normal.sample.puri
  ylimits=c(-0.01,1.01),
  lwd=2,
  points.alpha=0.8,
  yat=seq(0,1,0.2),
  xaxis.lab=c('Basal-like','HER2-enriched','Luminal A','Luminal B','Non-malignant'),
  use.legacy.settings=TRUE,
  width=5.5
  );
```

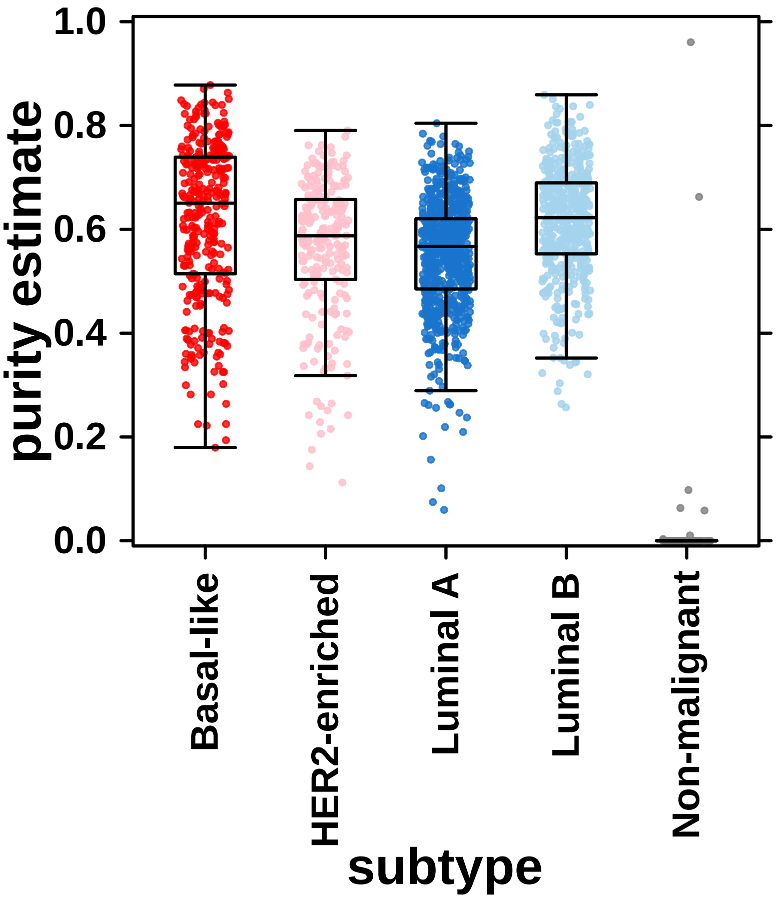

7 Supplementary Figure 3C - Associations with Pathologist or ISOpure estimating higher purity

Set up the environment

```
library(BoutrosLab.plotting.general);
library(yaml);
dataset.name <- 'Metabric';
```

Yaml file name

```
yaml.file <- paste0(dataset.name,'.yaml');
```

Read yaml with file information

```
dataset.files <- yaml.load_file(yaml.file);
```

Load clinical annotation

```
patient.anno <- read.table(dataset.files$clinical.annotation.file,sep='\t',header=TRUE);
```

Load ISOpure purity estimates

```
purity.estimates <- read.table(dataset.files$isopure.purity.estimate.file,header=TRUE);
patient.anno[rownames(purity.estimates),'purity'] <- purity.estimates$alpha.purity;
patient.anno <- patient.anno[!is.na(patient.anno$purity),];
```

Remove Normal-like patients

```
patient.anno <- patient.anno[which(!patient.anno$subtype %in% c('Normal-like','unknown')),];
```

Subtype colour scheme

```
subtype.colours <- c('red','pink','dodgerblue3','lightskyblue2');
names(subtype.colours) <- c('Basal','Her2','LumA','LumB');
```

Group patients based on isopure and pathologist purity estimates

```
patient.anno[['comparison']] <- rep('good.enough',nrow(patient.anno));
low.cellularity.patients <- !is.na(patient.anno$cellularity) & patient.anno$cellularity == 'low';
mod.cellularity.patients <- !is.na(patient.anno$cellularity) & patient.anno$cellularity == 'moderate'
high.cellularity.patients <- !is.na(patient.anno$cellularity) & patient.anno$cellularity == 'high';
patient.anno[mod.cellularity.patients & patient.anno$purity > 0.7+0.25,'comparison'] <- 'molecular.ve
patient.anno[low.cellularity.patients & patient.anno$purity > 0.4+0.25,'comparison'] <- 'molecular.ve
patient.anno[high.cellularity.patients & patient.anno$purity < 0.7-0.25,'comparison'] <- 'pathologist
patient.anno[mod.cellularity.patients & patient.anno$purity < 0.4-0.25,'comparison'] <- 'pathologist.
patient.anno$comparison <- factor(
  as.character(patient.anno$comparison),
  levels=c('pathologist.very.high','good.enough','molecular.very.high')
  );
```

ER barplots

```
plot.data <- cbind(
  comparison=rep(c('pathologist.very.high','good.enough','molecular.very.high'),each=2),
  rbind(
    as.data.frame(
      table(patient.anno[patient.anno$comparison %in% 'pathologist.very.high','ER'])/
      sum(patient.anno$comparison %in% 'pathologist.very.high')
      ),
    as.data.frame(
```

```
      table(patient.anno[patient.anno$comparison %in% 'good.enough','ER'])/
      sum(patient.anno$comparison %in% 'good.enough')
      ),
    as.data.frame(
      table(patient.anno[patient.anno$comparison %in% 'molecular.very.high','ER'])/
      sum(patient.anno$comparison %in% 'molecular.very.high')
      )
    )
  );
plot.data$comparison <- factor(
  as.character(plot.data$comparison),
  levels=c('pathologist.very.high','good.enough','molecular.very.high')
  );
er.barplot <- create.barplot(
  Freq ~ comparison,
  plot.data,
  groups=factor(as.character(plot.data$Var1),levels=c('+','-')),
  col=c('aquamarine4','antiquewhite'),
  stack=TRUE
  );
```

PGR barplots

```
plot.data <- cbind(
  comparison=rep(c('pathologist.very.high','good.enough','molecular.very.high'),each=2),
  rbind(
    as.data.frame(
      table(patient.anno[patient.anno$comparison %in% 'pathologist.very.high','PgR'])/
      sum(patient.anno$comparison %in% 'pathologist.very.high')
      ),
    as.data.frame(
      table(patient.anno[patient.anno$comparison %in% 'good.enough','PgR'])/
      sum(patient.anno$comparison %in% 'good.enough')
      ),
    as.data.frame(
      table(patient.anno[patient.anno$comparison %in% 'molecular.very.high','PgR'])/
      sum(patient.anno$comparison %in% 'molecular.very.high')
      )
    )
  );
plot.data$comparison <- factor(
  as.character(plot.data$comparison),
  levels=c('pathologist.very.high','good.enough','molecular.very.high')
  );
pgr.barplot <- create.barplot(
  Freq ~ comparison,
  plot.data,
  groups=factor(as.character(plot.data$Var1),levels=c('+','-')),
  col=c('cadetblue4','antiquewhite'),
  stack=TRUE
```

```
  );
```

HER2 barplots

```
plot.data <- cbind(
  comparison=rep(c('pathologist.very.high','good.enough','molecular.very.high'),each=2),
  rbind(
    as.data.frame(
      table(patient.anno[patient.anno$comparison %in% 'pathologist.very.high','ERB'])/
      sum(patient.anno$comparison %in% 'pathologist.very.high')
      ),
    as.data.frame(
      table(patient.anno[patient.anno$comparison %in% 'good.enough','ERB'])/
      sum(patient.anno$comparison %in% 'good.enough')
      ),
    as.data.frame(
      table(patient.anno[patient.anno$comparison %in% 'molecular.very.high','ERB'])/
      sum(patient.anno$comparison %in% 'molecular.very.high')
      )
    )
  );
plot.data$comparison <- factor(
  as.character(plot.data$comparison),
  levels=c('pathologist.very.high','good.enough','molecular.very.high')
  );
erb.barplot <- create.barplot(
  Freq ~ comparison,
  plot.data,
  groups=factor(as.character(plot.data$Var1),levels=c('+','-')),
  col=c('burlywood4','antiquewhite'),
  stack=TRUE
  );
```

PAM50 barplots

```
plot.data <- cbind(
  comparison=rep(c('pathologist.very.high','good.enough','molecular.very.high'),each=6),
  rbind(
    as.data.frame(
      table(patient.anno[patient.anno$comparison %in% 'pathologist.very.high','Pam50Subtype'])/
      sum(patient.anno$comparison %in% 'pathologist.very.high')
      ),
    as.data.frame(
      table(patient.anno[patient.anno$comparison %in% 'good.enough','Pam50Subtype'])/
      sum(patient.anno$comparison %in% 'good.enough')
      ),
    as.data.frame(
      table(patient.anno[patient.anno$comparison %in% 'molecular.very.high','Pam50Subtype'])/
      sum(patient.anno$comparison %in% 'molecular.very.high')
      )
```

```
    )
  );
plot.data$comparison <- factor(
  as.character(plot.data$comparison),
  levels=c('pathologist.very.high','good.enough','molecular.very.high')
  );
pam50.barplot <- create.barplot(
  Freq ~ comparison,
  plot.data,
  groups=factor(as.character(plot.data$Var1),levels=c('Basal','Her2','LumA','LumB')),
  col=c('red','pink','dodgerblue3','lightskyblue2'),
  stack=TRUE
  );
```

Stage barplots

```
plot.data <- cbind(
  comparison=rep(c('pathologist.very.high','good.enough','molecular.very.high'),each=6),
  rbind(
    as.data.frame(
      table(patient.anno[patient.anno$comparison %in% 'pathologist.very.high','stage'])/
      sum(patient.anno$comparison %in% 'pathologist.very.high')
      ),
    as.data.frame(
      table(patient.anno[patient.anno$comparison %in% 'good.enough','stage'])/
      sum(patient.anno$comparison %in% 'good.enough')
      ),
    as.data.frame(
      table(patient.anno[patient.anno$comparison %in% 'molecular.very.high','stage'])/
      sum(patient.anno$comparison %in% 'molecular.very.high')
      )
    )
  );
plot.data$comparison <- factor(
  as.character(plot.data$comparison),
  levels=c('pathologist.very.high','good.enough','molecular.very.high')
  );
stage.barplot <- create.barplot(
  Freq ~ comparison,
  plot.data,
  groups=factor(as.character(plot.data$Var1),levels=c('0','1','2','3','4','null')),
  col=c('lightgoldenrodyellow','lightgoldenrod3','lightgoldenrod4','saddlebrown','black','ivory3'),
  stack=TRUE
  );
chisq.test(matrix(c(
  table(patient.anno[patient.anno$comparison %in% 'pathologist.very.high','stage']),
  table(patient.anno[patient.anno$comparison %in% 'good.enough','stage'])
  ),ncol=2))
```

```
Pearson's Chi-squared test

data:  matrix(c(table(patient.anno[patient.anno$comparison %in% "pathologist.very.high",     "stage"]
X-squared = 15.305, df = 5, p-value = 0.009134

chisq.test(matrix(c(
  table(patient.anno[patient.anno$comparison %in% 'molecular.very.high','stage']),
  table(patient.anno[patient.anno$comparison %in% 'good.enough','stage'])
  ),ncol=2))


Pearson's Chi-squared test

data:  matrix(c(table(patient.anno[patient.anno$comparison %in% "molecular.very.high",     "stage"]),
X-squared = 1.9948, df = 5, p-value = 0.8499
```

Grade barplots

```
patient.anno$grade <- as.character(patient.anno$grade);
patient.anno$grade[is.na(patient.anno$grade)] <- 'null';
patient.anno$grade <- factor(patient.anno$grade,levels=c('1','2','3','null'));
plot.data <- cbind(comparison=rep(c('pathologist.very.high','good.enough','molecular.very.high'),each
    rbind(
      as.data.frame(
        table(patient.anno[patient.anno$comparison %in% 'pathologist.very.high','grade'])/
        sum(patient.anno$comparison %in% 'pathologist.very.high')
        ),
      as.data.frame(
        table(patient.anno[patient.anno$comparison %in% 'good.enough','grade'])/
        sum(patient.anno$comparison %in% 'good.enough')
        ),
      as.data.frame(
        table(patient.anno[patient.anno$comparison %in% 'molecular.very.high','grade'])/
        sum(patient.anno$comparison %in% 'molecular.very.high')
        )
      )
    );
plot.data$comparison <- factor(
  as.character(plot.data$comparison),
  levels=c('pathologist.very.high','good.enough','molecular.very.high')
  );
grade.barplot <- create.barplot(
  Freq ~ comparison,
  plot.data,
  groups=factor(as.character(plot.data$Var1),levels=c('1','2','3','null')),
  col=c('darkseagreen1','darkseagreen4','darkgreen','ivory3'),
  stack=TRUE
  );
```

Age boxplots

```
age.boxplot <- create.boxplot(
  age ~ comparison,
  patient.anno
  );
```

Create the legend for the combined plot

```
legend <- legend.grob(
  list(
    legend = list(
      colours=rev(c('darkseagreen1','darkseagreen4','darkgreen','ivory3')),
      title='Grade',
      labels=rev(c('1','2','3','unknown'))
      ),
    legend = list(
      colours=rev(c(
        'lightgoldenrodyellow',
        'lightgoldenrod3',
        'lightgoldenrod4',
        'saddlebrown',
        'black',
        'ivory3'
        )),
      title='Stage',
      labels=rev(c('0','1','2','3','4','unknown'))
      ),
    legend = list(
      colours=rev(c('red','pink','dodgerblue3','lightskyblue2')),
      title='PAM50 Subtype',
      labels=rev(c('Basal','HER2-enriched','Luminal A','Luminal B'))
      ),
    legend = list(
      colours=rev(c('burlywood4','antiquewhite')),
      labels=rev(c('ERB+','ERB-'))
      ),
    legend = list(
      colours=rev(c('cadetblue4','antiquewhite')),
      labels=rev(c('PgR+','PgR-'))
      ),
    legend = list(
      colours=rev(c('aquamarine4','antiquewhite')),
      labels=rev(c('ER+','ER-'))
      )
    ),
    title.just = 'left'
  );
```

Combine barplots

```
create.multiplot(
  list(er.barplot,pgr.barplot,erb.barplot,pam50.barplot,stage.barplot,grade.barplot,age.boxplot),
  filename = './sfigure3c.pdf',
  resolution=200,
  xaxis.labels=c('Pathologist higher','not extremely\ndifferent','ISOpure higher'),
  xaxis.cex=0.8,
  yaxis.cex=0.8,
  width=6,
  height=6,
  y.relation='free',
  panel.heights=c(2,2,2,2,1,1,1),
  ylimits=list(c(0,1),c(0,1),c(0,1),c(0,1),c(0,1),c(0,1),c(20,100)),
  yat=list(c(0,0.5,1),c(0,0.5,1),c(0,0.5,1),c(0,0.5,1),c(0,0.5,1),c(0,0.5,1),seq(20,100,40)),
  style='Nature',
  ylab.label=c('Age    ','','Grade','','Stage','','PAM50','','    ERB','PgR','ER'),
  ylab.cex=1,
  legend = list(right = list(fun = legend)),
  print.new.legend = TRUE,
  use.legacy.settings=TRUE
  );
```

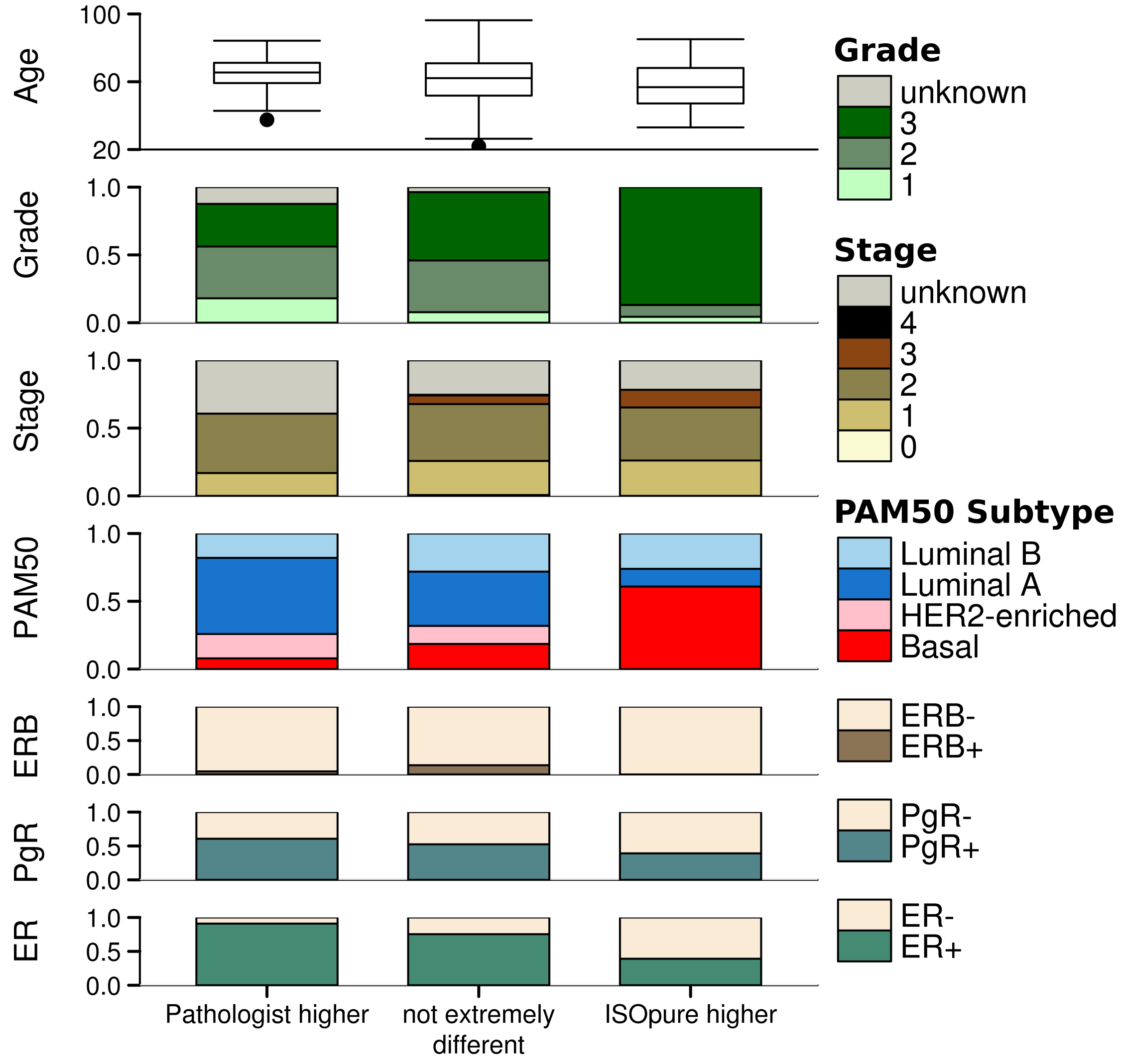

8 Running Clustering Patients

Set up the environment

```
library(ConsensusClusterPlus.custom);
library(yaml);
args <- commandArgs(trailingOnly = TRUE);
dataset.name <- args[1];
profile.type.to.cluster <- args[2];
```

Yaml file name

```
yaml.file <- paste0(dataset.name,'.yaml');
```

Read yaml with file information

```
dataset.files <- yaml.load_file(yaml.file);
```

Load clinical annotation

```
patient.anno <- read.table(
  dataset.files$clinical.annotation.file,
  sep='\t',
  header=TRUE
  );
```

Load mRNA abundance profiles

```
profile.types <- c('bulk','tc','tac');
rna.data.matrix <- list();
for(profile.type in profile.types) {
  rna.data.matrix[[profile.type]] <- read.table(
    dataset.files[[paste0(profile.type,'.mrna.abundance.file')]],
    header=TRUE,
    sep='\t'
    );
  }
```

Determine the most variable the genes to cluster

```
bulk.sd <- apply(rna.data.matrix$bulk,1,sd);
tc.sd <- apply(rna.data.matrix$tc,1,sd);
tac.sd <- apply(rna.data.matrix$tac,1,sd);
higher.variable.genes <- bulk.sd > 1 | tc.sd > 1 | tac.sd > 1;
```

Cluster patients

```
results <- ConsensusClusterPlus(
  d=as.matrix(rna.data.matrix[[profile.type.to.cluster]][higher.variable.genes,]),
  plot='pdf',
  maxK=7,
  distance='jaccard',
  verbose=TRUE,
  writeTable=TRUE,
  title=paste(dataset.name,'_',profile.type.to.cluster,'_ConsensusClusterPlus',sep=''),
  seed=17,
  finalLinkage='ward',
  innerLinkage='ward',
  clusterAlg='hc',
  pFeature=0.8,
  pItem=0.8,
  reps=1000
  );
```

9 Figure 2A - PAM50 gene variation

Set up the environment

```
library(BoutrosLab.plotting.general);
library(yaml);
dataset.name <- 'Metabric';
```

Yaml file name

```
yaml.file <- paste0(dataset.name,'.yaml');
```

Read yaml with file information

```
dataset.files <- yaml.load_file(yaml.file);
```

Load clinical annotation

```
patient.anno <- read.table(dataset.files$clinical.annotation.file,sep='\t',header=TRUE);
```

Load mRNA abundance profiles

```
profile.types <- c('bulk','tc','tac');
rna.data.matrix <- list();
for(profile.type in profile.types) {
  rna.data.matrix[[profile.type]] <- read.table(
    dataset.files[[paste0(profile.type,'.mrna.abundance.file')]],
    header=TRUE,
    sep='\t'
    );
  }
```

Load PAM50 gene list

```
pam50.genes <- read.table(dataset.files$pam50.gene.list.file,sep='\t',header=TRUE,row.names=1);
pam50.genes$probe <- paste0(pam50.genes[, "EntrezID"],'_at');
rownames(pam50.genes) <- as.character(pam50.genes$probe);
```

Subtype colour scheme

```
subtype.colours <- c('red','pink','dodgerblue3','lightskyblue2','forestgreen');
names(subtype.colours) <- c('Basal','Her2','LumA','LumB','Normal');
```

Calculate subtype mRNA abundance average for each pam50 gene and each profile type

```
pam50.data.matrix <- list();
gene.profile.averages <- list();
for(profile.type in profile.types) {
  pam50.data.matrix[[profile.type]] <- rna.data.matrix[[profile.type]][
    intersect(
```

```
      pam50.genes$probe,
      rownames(rna.data.matrix[[profile.type]])
      )
    ,];
  gene.profile.averages[[profile.type]] <- sapply(
    c('Basal-like','HER2-enriched','Luminal A','Luminal B'),
    function(stype) {
      stype.patients <- which(
        colnames(rna.data.matrix[[profile.type]])
        %in%
        patient.anno$patient_id[patient.anno$subtype == stype]
        );
      rowSums(t(scale(t(pam50.data.matrix[[profile.type]])))[,stype.patients])/length(stype.patients)
      }
    );
  colnames(gene.profile.averages[[profile.type]]) <- paste(
    profile.type,colnames(gene.profile.averages[[profile.type]])
    );
  }
```

Combine the scaled profiles to make the matrix for plotting

```
heatmap.data <- cbind(
  gene.profile.averages$bulk,
  gene.profile.averages$tc,
  gene.profile.averages$tac
  );
rownames(heatmap.data) <- as.character(pam50.genes[rownames(heatmap.data),'GeneSymbol']);
```

Legend for the right of the plot

```
cov.legend <- list(
    legend = list(
        colours = c('darkred','dodgerblue','gold'),
        labels = c('Bulk','TC','TAC'),
    title='Profile Type'
        ),
    legend = list(
        colours = c('red','pink','dodgerblue3','lightskyblue2'),
        labels = c('Basal-like','HER2-enriched','Luminal A','Luminal B'),
    title='PAM50 Subtype'
        )
    );
```

Covariate bar for the top of the plot

```
profile.cov <- list(
  rect=list(
    col='transparent',
    fill=rep(c('red','pink','dodgerblue3','lightskyblue2'),each=3),
```

```
    lwd=1.5
    ),
  rect=list(
    col='transparent',
    fill=rep(c('darkred','dodgerblue','gold'),4),
    lwd=1.5
    )
  );
create.heatmap(
  heatmap.data[,order(rep(1:4,3))],
  filename = './figure2a.pdf',
  colour.scheme=c('blue','white','red'),
  cluster.dimensions='both',
  resolution=175,
  same.as.matrix=TRUE,
  yaxis.lab=NA,
  yaxis.cex=0.75,
  colour.alpha=1,
  colourkey.cex=1,
  covariates.top=profile.cov,
  covariate.legend=cov.legend,
  legend.side='right',
  width=8,
  height=7,
  legend.title.just='left',
  use.legacy.settings = TRUE
  );
```

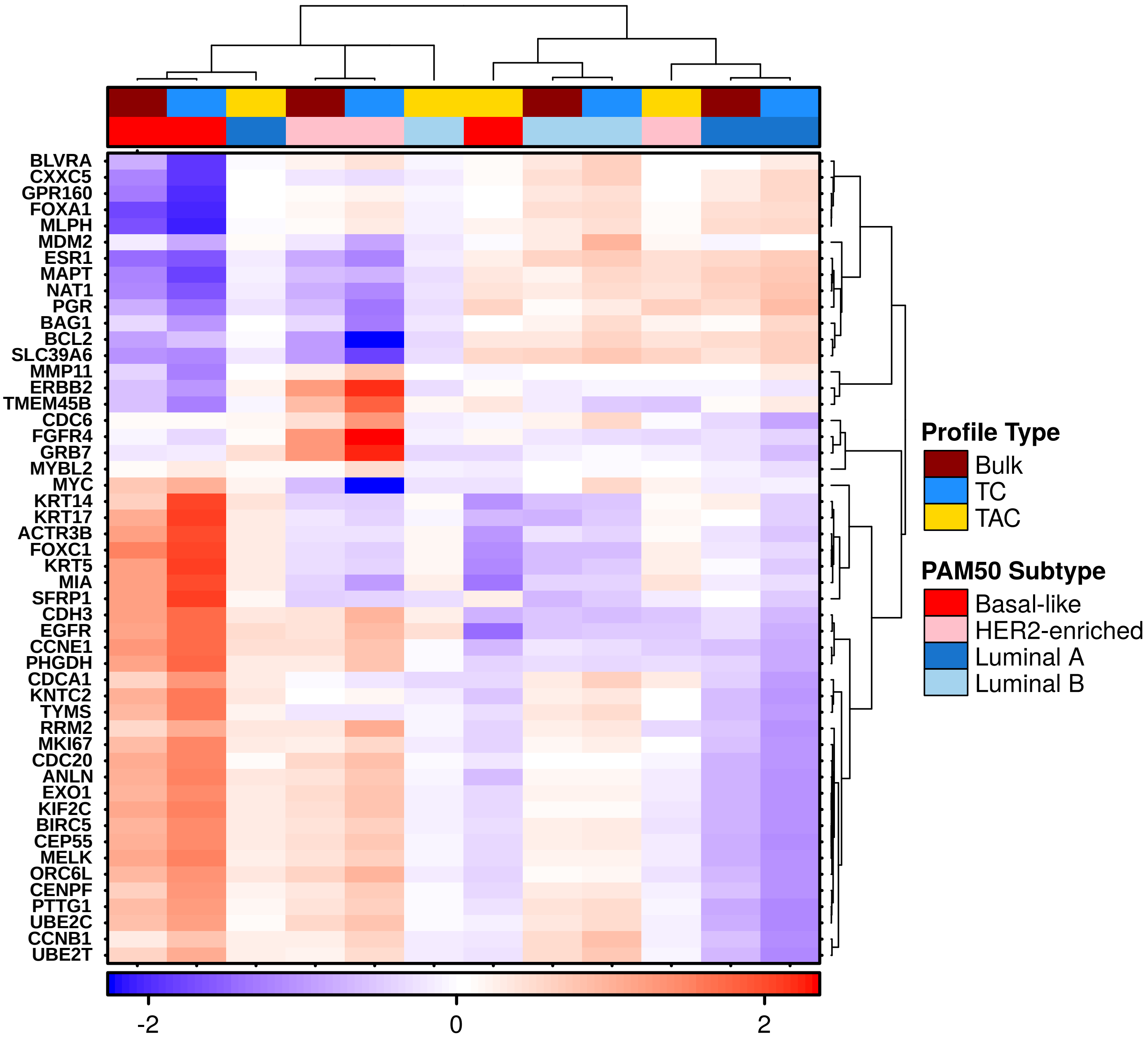

10 Figure 2 - Clustering highly variant genes to recapitulate breast cancer subtypes

Set up the environment

```r
library(BoutrosLab.plotting.general);
library(yaml);
dataset.name <- 'Metabric';
```

Yaml file name

```r
yaml.file <- paste0(dataset.name,'.yaml');
```

Read yaml with file information

```
dataset.files <- yaml.load_file(yaml.file);
subtype.options <- c('Basal-like','HER2-enriched','Luminal A','Luminal B');
subtype.col <- c('red','pink','dodgerblue3','lightskyblue2');
```

Load clinical annotation

```
patient.anno <- read.table(dataset.files$clinical.annotation.file,sep='\t',header=TRUE);
patient.anno <- patient.anno[which(patient.anno$subtype %in% subtype.options),];
cluster.num <- 4;
```

Create list of all possible ways that the subtypes can be assigned to the clusters

```
possible.assignment.orders <- matrix(NA,ncol=4,nrow=24);
matrix.idx <- 1;
for(i1 in 1:4) {
  for(i2 in setdiff(1:4,i1)) {
    for(i3 in setdiff(1:4,c(i1,i2))) {
      for(i4 in setdiff(1:4,c(i1,i2,i3))) {
        possible.assignment.orders[matrix.idx,] <- c(i1,i2,i3,i4);
        matrix.idx <- matrix.idx+1;
        }
      }
    }
  }
barplot.data <- list(
  cluster=rep(1:cluster.num,each=length(subtype.options)),
  subtype=rep(subtype.options,cluster.num)
  );
barplot.data$group <- paste(barplot.data$cluster,barplot.data$subtype,sep=':');
barplot.data <- as.data.frame(barplot.data);
profile.types <- c('bulk','tc','tac');
plots <- list();
xaxis.labels <- list();
patient.assignments <- list();
for(profile.type in profile.types) {
  # load results from clustering profiles
  filename <- paste0(
    sub(paste0('/plots/',dataset.name,'/'),'',dataset.files$parent.output.dir),
    '/results/clustering/',
    dataset.name,
    '_',
    profile.type,
    '_ConsensusClusterPlus/',
    dataset.name,
    '_',
    profile.type,
    '_ConsensusClusterPlus.k=',
    cluster.num,
    '.consensusClass.csv'
    );
```

```
  cluster.results <- read.csv(
    filename,
    header=FALSE
  );
  colnames(cluster.results) <- c('patient','cluster');
  rownames(cluster.results) <- cluster.results$patient;
  cluster.results <- cluster.results[sort(rownames(cluster.results)),];
  # filter to patients that were in the dataset and have subtype information
  intersecting.patients <- intersect(rownames(cluster.results),rownames(patient.anno));
  cluster.results <- cluster.results[intersecting.patients,];
  cluster.results$subtype <- patient.anno[intersecting.patients,'subtype'];
  # initialize the right size for the barplot data
  barplot.data <- cbind(barplot.data, rep(0,cluster.num*length(subtype.options)));
  colnames(barplot.data)[ncol(barplot.data)] <- paste0('count_',profile.type);
  # add the counts for the number of patients from each subtype in each cluster to the barplot data
  barplot.data[
    match(
      names(table(paste(cluster.results$cluster,cluster.results$subtype,sep=':'))),
      barplot.data$group
      ),
    paste0('count_',profile.type)
    ] <- as.numeric(table(paste(cluster.results$cluster,cluster.results$subtype,sep=':')));
  # decide which subtype corresponds to each cluster
  subtype.cat <- c('Basal-like','HER2-enriched','Luminal A','Luminal B');
  possible.count.correct <- apply(
    possible.assignment.orders,
    1,
    function(x) {sum(barplot.data[as.character(barplot.data$subtype) == subtype.cat[x][barplot.data$c
  cluster.subtype.allocations <- subtype.cat[
    possible.assignment.orders[which(possible.count.correct == max(possible.count.correct)),]
    ];
  barplot.data <- cbind(barplot.data, cluster.subtype.allocations[barplot.data$cluster]);
  colnames(barplot.data)[ncol(barplot.data)] <- paste0('cluster_class_',profile.type);
  # subtype assignments for each cluster per patient
  patient.assignments[[paste0('cluster_class_',profile.type)]] <- cluster.subtype.allocations[cluster
  }
```

Calculate the number correctly and incorrectly classified for each subtype

```
class.compare <- data.frame(
  subtype=c('Basal-like','HER2-enriched','Luminal A','Luminal B'),
  count_correct_bulk=rep(NA,4),
  count_misclassified_bulk=rep(NA,4),
  count_correct_tc=rep(NA,4),
  count_misclassified_tc=rep(NA,4),
  count_correct_tac=rep(NA,4),
  count_misclassified_tac=rep(NA,4)
  );
for(profile.type in profile.types) {
  for(i in 1:nrow(class.compare)) {
```

```
    class.compare[i,paste0('count_correct_',profile.type)] <- sum(barplot.data[
      as.character(barplot.data$subtype) == class.compare$subtype[i] &
        as.character(barplot.data[,paste0('cluster_class_',profile.type)]) == class.compare$subtype[i
      paste0('count_',profile.type)
      ]);
    class.compare[i,paste0('count_misclassified_',profile.type)] <- sum(barplot.data[
      as.character(barplot.data$subtype) == class.compare$subtype[i] &
        as.character(barplot.data[,paste0('cluster_class_',profile.type)]) != class.compare$subtype[i
      paste0('count_',profile.type)
      ]);
    }
  }
for(profile.type in profile.types) {
  # sensitivity calculations (TP / all patients in subtype)
  class.compare <- cbind(
    class.compare,
    sapply(
      1:4,
      function(i) {
          class.compare[i,paste0('count_correct_',profile.type)]/
          sum(barplot.data[as.character(barplot.data$subtype) == class.compare$subtype[i],paste0('cou
          }
      )
    );
  colnames(class.compare)[ncol(class.compare)] <- paste0('sensitivity_',profile.type);
  # specificity calculations (TN / all patients not in subtype)
  class.compare <- cbind(
    class.compare,
    sapply(
      1:4,
      function(i) {
        sum(barplot.data[
          as.character(barplot.data$subtype) != class.compare$subtype[i]
          & as.character(barplot.data[,paste0('cluster_class_',profile.type)]) != class.compare$subty
          paste0('count_',profile.type)
          ])/
        sum(barplot.data[as.character(barplot.data$subtype) != class.compare$subtype[i],paste0('count
        }
      )
    );
  colnames(class.compare)[ncol(class.compare)] <- paste0('specificity_',profile.type);
  # accuracy calculations
  class.compare <- cbind(
    class.compare,
    sapply(
      1:4,
      function(i) {
        (sum(barplot.data[
          as.character(barplot.data$subtype) != class.compare$subtype[i]
          & as.character(barplot.data[,paste0('cluster_class_',profile.type)]) != class.compare$subty
```

```
    ])+class.compare[i,paste0('count_correct_',profile.type)])/
    sum(barplot.data[,paste0('count_',profile.type)])
    }
  )
 );
colnames(class.compare)[ncol(class.compare)] <- paste0('accuracy_',profile.type);
# precision calculations
class.compare <- cbind(
 class.compare,
 sapply(
  1:4,
  function(i) {
   class.compare[i,paste0('count_correct_',profile.type)]/
   sum(barplot.data[
    as.character(barplot.data[,paste0('cluster_class_',profile.type)]) == class.compare$subtype
    paste0('count_',profile.type)
    ])
   }
  )
 );
colnames(class.compare)[ncol(class.compare)] <- paste0('precision_',profile.type);
}
```

Create sensitivity per subtype barplot

```
create.barplot(
 sensitivity ~ subtype,
 data.frame(
  sensitivity=c(class.compare$sensitivity_bulk,class.compare$sensitivity_tc,class.compare$sensitivi
  subtype=factor(rep(class.compare$subtype,3))
  ),
 filename = './figure2c.pdf',
 stack=FALSE,
 groups=rep(1:3,each=4),
 col=c('darkred','dodgerblue','gold'),
 xaxis.rot=90,
 ylimits=c(0,1),
 yat=seq(0,1,0.2),
 yaxis.lab=seq(0,1,0.2),
 height=5,
 width=4,
 resolution=200,
 use.legacy.settings=TRUE
 );
```

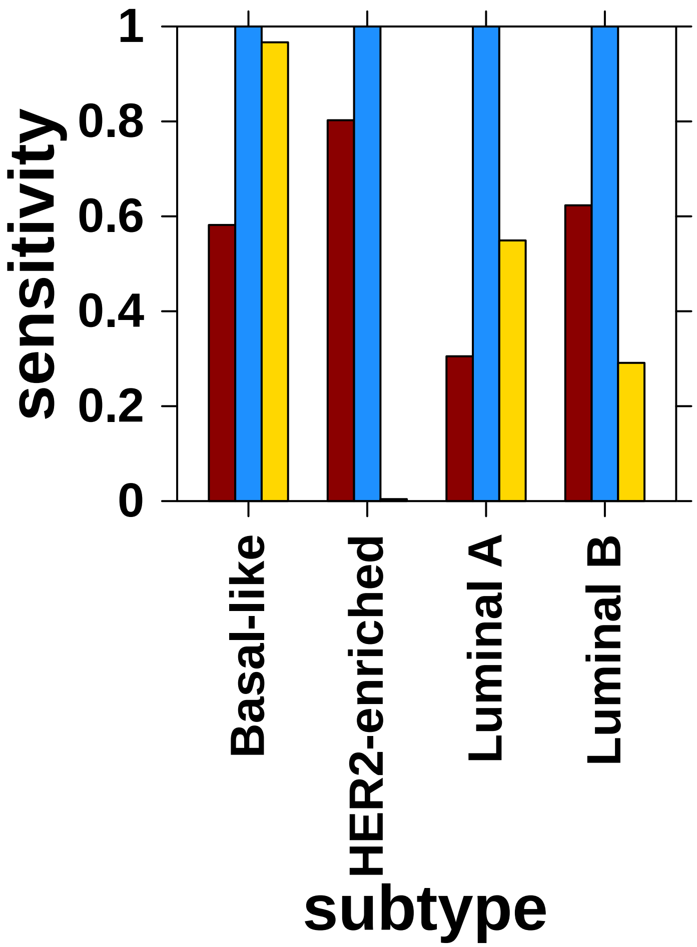

Create specificity per subtype barplot

```
create.barplot(
  specificity ~ subtype,
  data.frame(
    specificity=c(class.compare$specificity_bulk,class.compare$specificity_tc,class.compare$specifici
    subtype=factor(rep(class.compare$subtype,3))
    ),
  filename = './figure2d.pdf',
  stack=FALSE,
  groups=rep(1:3,each=4),
  col=c('darkred','dodgerblue','gold'),
  xaxis.rot=90,
  ylimits=c(0,1),
  yat=seq(0,1,0.2),
  yaxis.lab=seq(0,1,0.2),
  height=5,
  width=4,
  resolution=250,
  use.legacy.settings=TRUE
  );
```

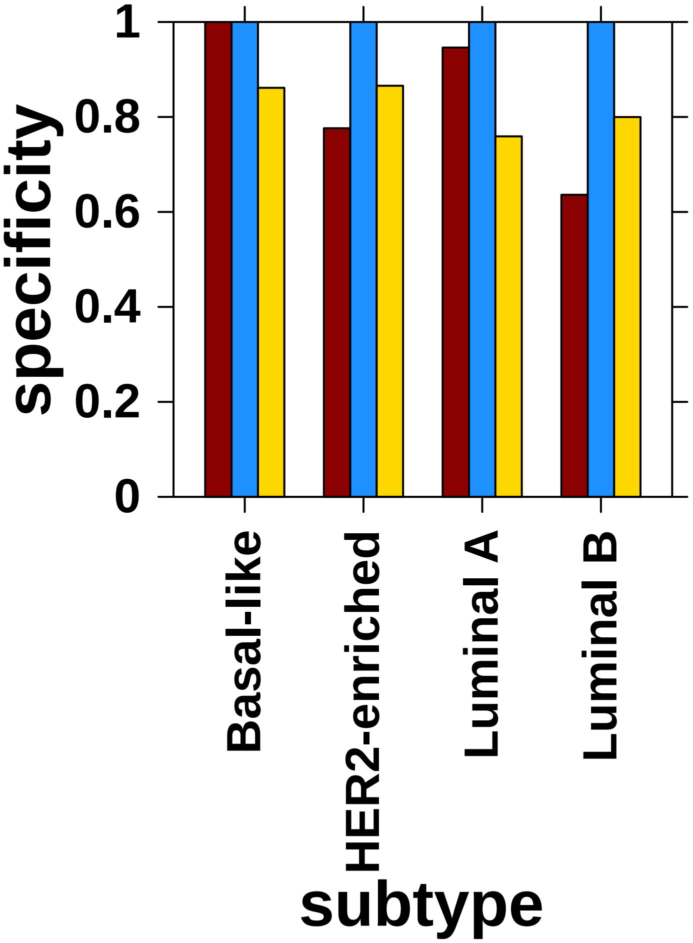

Create accuracy per subtype barplot

```
create.barplot(
  accuracy ~ subtype,
  data.frame(
    accuracy=c(class.compare$accuracy_bulk,class.compare$accuracy_tc,class.compare$accuracy_tac),
    subtype=factor(rep(class.compare$subtype,3))
    ),
  filename = './figure2e.pdf',
  stack=FALSE,
  groups=rep(1:3,each=4),
  col=c('darkred','dodgerblue','gold'),
  xaxis.rot=90,
  ylimits=c(0,1),
  yat=seq(0,1,0.2),
  yaxis.lab=seq(0,1,0.2),
  height=5,
  width=4,
  resolution=250,
  use.legacy.settings=TRUE
  );
```

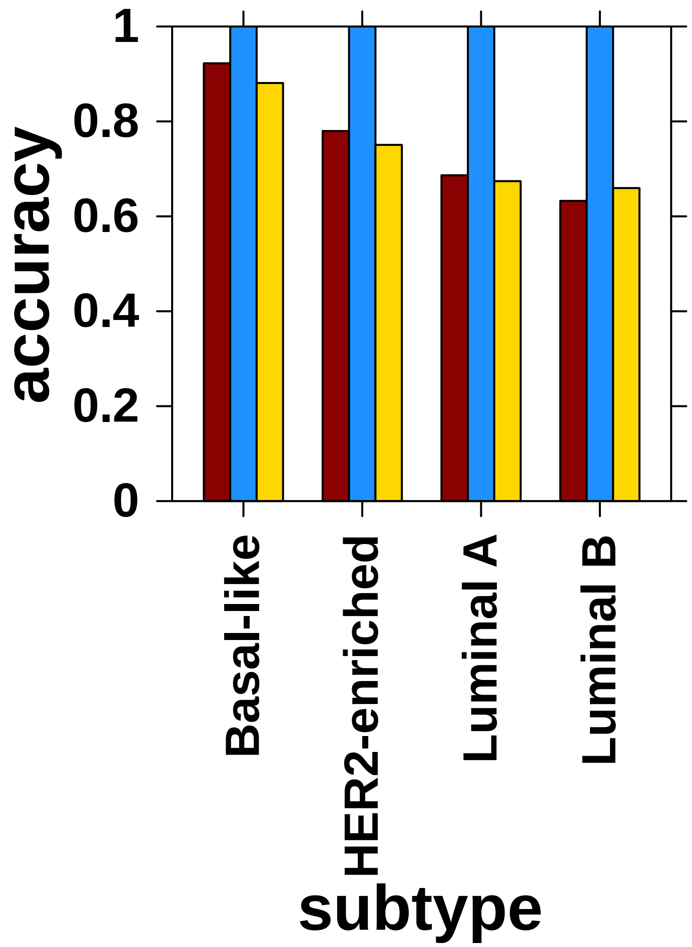

Collect the data for creating heatmaps showing classification

```
heatmap.data <- as.matrix(cbind(cluster.results$subtype,as.data.frame(patient.assignments)));
for(i in 1:ncol(heatmap.data)) {
  heatmap.data[,i] <- factor(heatmap.data[,i],levels=c('Basal-like','HER2-enriched','Luminal A','Lumi
  heatmap.data[,i] <- as.numeric(heatmap.data[,i]);
  }
heatmap.data <- as.data.frame(matrix(as.numeric(heatmap.data),ncol=4));
colnames(heatmap.data) <- colnames(cbind(cluster.results$subtype,as.data.frame(patient.assignments)))
patient.ordering <- order(heatmap.data$cluster_class_tc*1000+heatmap.data$cluster_class_bulk*10+heatm
heatmap.data.reordered <- heatmap.data[patient.ordering,4:1];
subtype.classification.colour.scheme <- c('red','pink','dodgerblue3','lightskyblue2');
plots.list <- list();
```

Create the bottom left heatmap with profile labels specifying eat row

```
plots.list[[1]] <- create.heatmap(
      heatmap.data.reordered[heatmap.data.reordered[,4]==1,-4],
      at=seq(0.5,4.5,1),
      colour.scheme=subtype.classification.colour.scheme,
      print.colour.key=FALSE,
      cluster.dimensions='none',
      xat=NULL,
      yaxis.lab=rev(c('Bulk clusters','TC clusters','TAC clusters'))
```

```
      );
```

Create the other bottom heatmaps with no labels

```
plot.idx <- 2;
for(i in 2:4) {
  plots.list[[plot.idx]] <- create.heatmap(
    heatmap.data.reordered[heatmap.data.reordered[,4]==i,-4],
    at=seq(0.5,4.5,1),
    colour.scheme=subtype.classification.colour.scheme,
    print.colour.key=FALSE,
    cluster.dimensions='none',
    yat=NULL,
    xat=NULL
    );
  plot.idx <- plot.idx + 1;
  }
```

Create the top row of heatmaps

```
for(i in 1:3) {
  plots.list[[plot.idx]] <- create.heatmap(
    heatmap.data.reordered[heatmap.data.reordered[,4]==i,c(4,4)],
    at=seq(0.5,4.5,1),
    colour.scheme=subtype.classification.colour.scheme,
    print.colour.key=FALSE,
    cluster.dimensions='none',
    yat=NULL,
    xat=NULL
    );
  plot.idx <- plot.idx + 1;
  }
plots.list[[plot.idx]] <- create.heatmap(
  heatmap.data.reordered[heatmap.data.reordered[,4]==4,c(4,4)],
  at=seq(0.5,7.5,1),
  colour.scheme=subtype.classification.colour.scheme,
  print.colour.key=FALSE,
  cluster.dimensions='none',
  xat=-1,
  yat=-1
  );
```

Plot the heatmaps together to show comparison of the patient classifications

```
create.multiplot(
  plots.list,
  filename = './figure2b.pdf',
  height=4,
  width=12,
  resolution=500,
```

```
  yaxis.cex=1,
  plot.layout=c(4,2),
  panel.widths=sapply(1:4,function(i){sum(heatmap.data.reordered[,4]==i)/nrow(heatmap.data.reordered)
  panel.heights=c(1,3),
  x.relation='free',
  y.spacing=-0.5,
  x.spacing=-1,
  y.relation='free',
  left.padding=8,
  main='                    Basal-like          HER2-enriched                                     Luminal A
  main.cex=1
  );
```

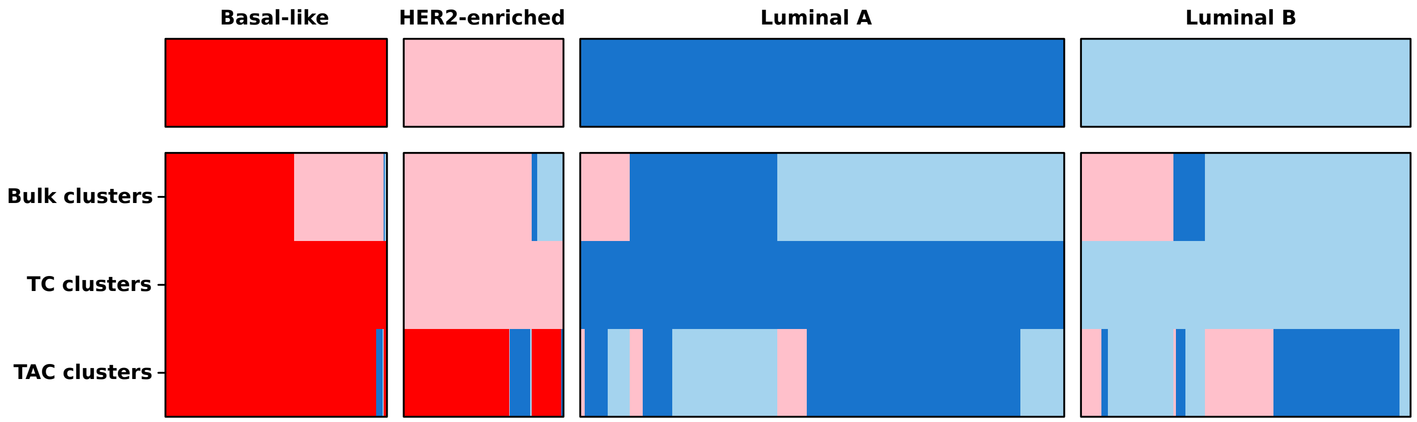

11 Supplementary Figure 4A-C - Clustered PAM50 mRNA abundance

Set up the environment

```
library(BoutrosLab.plotting.general);
library(yaml);
dataset.name <- 'Metabric';
```

Yaml file name

```
yaml.file <- paste0(dataset.name,'.yaml');
```

Read yaml with file information

```
dataset.files <- yaml.load_file(yaml.file);
```

Load clinical annotation

```
patient.anno <- read.table(dataset.files$clinical.annotation.file,sep='\t',header=TRUE);
```

Load mRNA abundance profiles

```
profile.types <- c('bulk','tc','tac');
rna.data.matrix <- list();
for(profile.type in profile.types) {
  rna.data.matrix[[profile.type]] <- read.table(
    dataset.files[[paste0(profile.type,'.mrna.abundance.file')]],
    header=TRUE,
    sep='\t'
    );
  }
```

Load PAM50 gene list

```
pam50.genes <- read.table(dataset.files$pam50.gene.list.file,sep='\t',header=TRUE,row.names=1);
pam50.genes$probe <- paste0(pam50.genes$EntrezID,'_at');
rownames(pam50.genes) <- as.character(pam50.genes$probe);
```

Subtype colour scheme

```
subtype.colours <- c('red','pink','dodgerblue3','lightskyblue2','forestgreen');
names(subtype.colours) <- c('Basal','Her2','LumA','LumB','Normal');
```

Calculate subtype mRNA abundance average for each pam50 gene and each profile type

```
pam50.data.matrix <- list();
for(profile.type in profile.types) {
  pam50.data.matrix[[profile.type]] <-
    rna.data.matrix[[profile.type]][intersect(pam50.genes$probe,rownames(rna.data.matrix[[profile.typ
  }
```

Create the subtype legend for the patient covariate on top of the plots

```
cov.legend <- list(
    legend = list(
        colours = c('red','pink','dodgerblue3','lightskyblue2'),
        labels = c('Basal-like','HER2-enriched','Luminal A','Luminal B')
        )
    );
```

Create a heatmap plot for each profile type

```
for(profile.type in profile.types) {
  # Create the the covariate bar at the top of the plot
  cov.col <- rep('grey',ncol(pam50.data.matrix[[profile.type]]));
  for(i in 1:length(cov.legend$legend$colours)) {
    cov.col[which(
      colnames(rna.data.matrix[[profile.type]])
      %in%
      patient.anno$patient_id[patient.anno$subtype == cov.legend$legend$labels[i]]
      )] <- cov.legend$legend$colours[i];
    }
```

```
profile.cov <- list(
  rect=list(
    col='transparent',
    fill=cov.col,
    lwd=1.5
    )
  );
# Add gene symbols to the rownames if not already there
if(any(grep(',',rownames(pam50.data.matrix[[profile.type]]),invert=TRUE))) {
  rownames(pam50.data.matrix[[profile.type]]) <- paste0(
      as.character(pam50.genes[rownames(pam50.data.matrix[[profile.type]]),'GeneSymbol']),
      ', ',
      sub('_at','',rownames(pam50.data.matrix[[profile.type]]))
      );
  }
create.heatmap(
  t(scale(t(pam50.data.matrix[[profile.type]]))),
  filename = paste0('./sfigure4abc_',profile.type,'.pdf'),
  colour.scheme=c('blue','white','red'),
  cluster.dimensions='both',
  resolution=180,
  same.as.matrix=TRUE,
  yaxis.lab=NA,
  colour.alpha=1,
  colourkey.cex=1,
  covariates.top = profile.cov,
  covariate.legend=cov.legend,
  legend.side='right',
  width=12,
  height=7,
  main=paste(sub('tac','TAC',sub('bulk','Bulk',sub('tc','TC',profile.type))),'Profiles'),
  main.cex=1.5,
  yaxis.cex = 0.81,
  use.legacy.settings = TRUE
  );
}
```

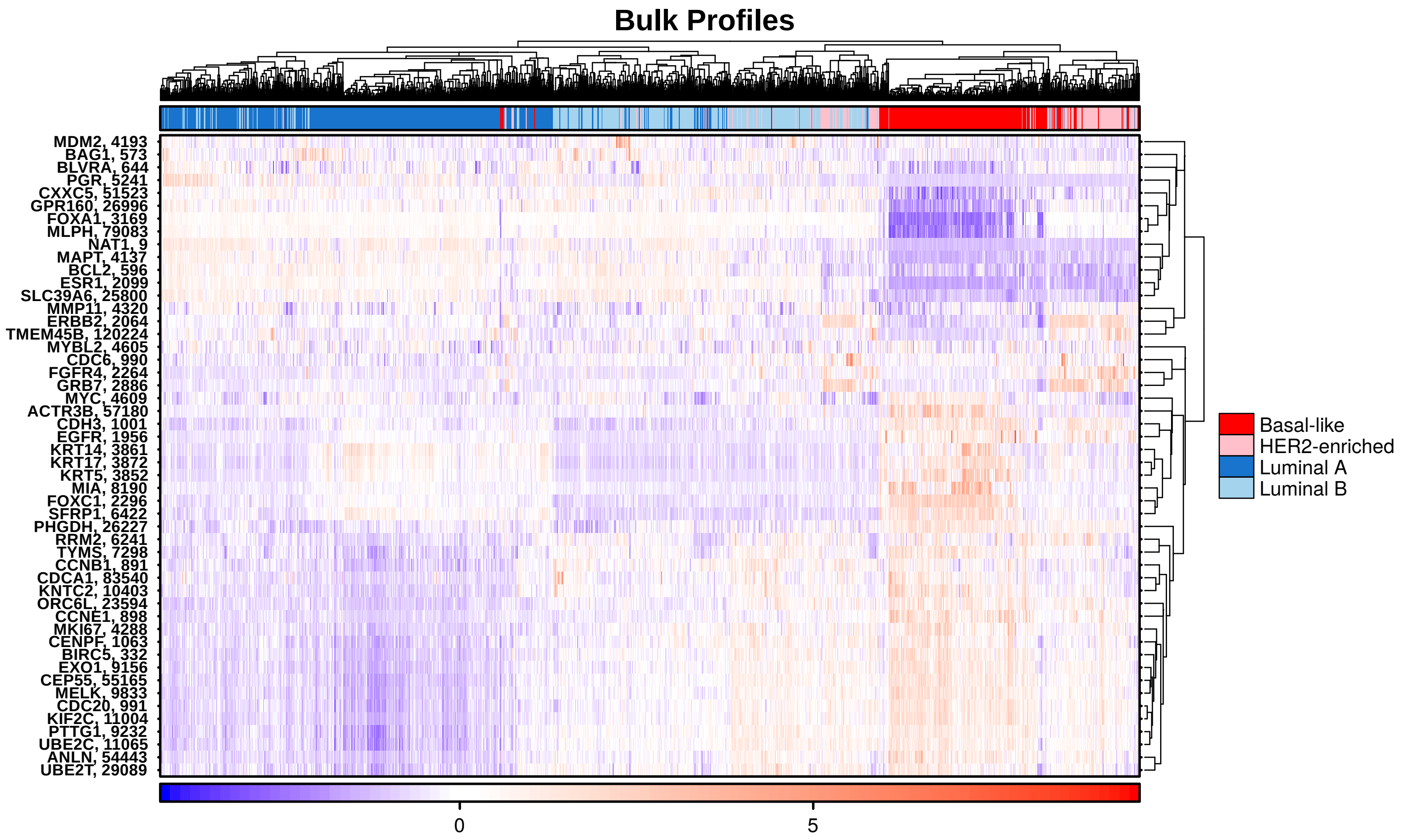
Bulk Profiles
MDM2, 4193
BAG1, 573
BLVRA, 644
PGR, 5241
CXXC5, 51523
GPR160, 26996
FOXA1, 3169
MLPH, 79083
NAT1, 9
MAPT, 4137
BCL2, 596
ESR1, 2099
SLC39A6, 25800
MMP11, 4320
ERBB2, 2064
TMEM45B, 120224
MYBL2, 4605
CDC6, 990
FGFR4, 2264
GRB7, 2886
MYC, 4609
ACTR3B, 57180
CDH3, 1001
EGFR, 1956
KRT14, 3861
KRT17, 3872
KRT5, 3852
MIA, 8190
FOXC1, 2296
SFRP1, 6422
PHGDH, 26227
RRM2, 6241
TYMS, 7298
CCNB1, 891
CDCA1, 83540
KNTC2, 10403
ORC6L, 23594
CCNE1, 898
MKI67, 4288
CENPF, 1063
BIRC5, 332
EXO1, 9156
CEP55, 55165
MELK, 9833
CDC20, 991
KIF2C, 11004
PTTG1, 9232
UBE2C, 11065
ANLN, 54443
UBE2T, 29089
Basal-like
HER2-enriched
Luminal A
Luminal B
0
5

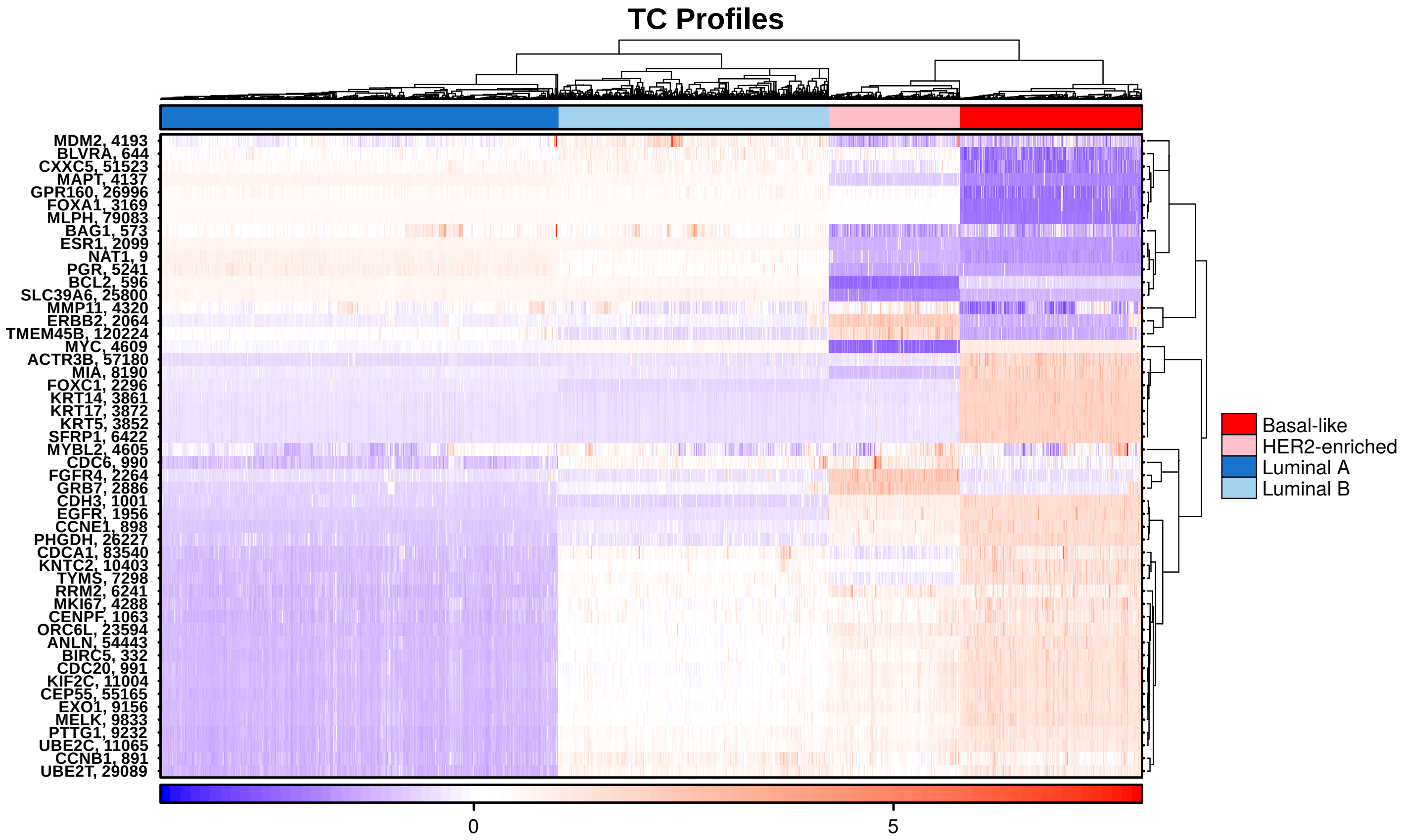
TC Profiles
MDM2, 4193
BLVRA, 644
CXXC5, 51523
MAPT, 4137
GPR160, 26996
FOXA1, 3169
MLPH, 79083
BAG1, 573
ESR1, 2099
NAT1, 9
PGR, 5241
BCL2, 596
SLC39A6, 25800
MMP11, 4320
ERBB2, 2064
TMEM45B, 120224
MYC, 4609
ACTR3B, 57180
MIA, 8190
FOXC1, 2296
KRT14, 3861
KRT17, 3872
KRT5, 3852
SFRP1, 6422
MYBL2, 4605
CDC6, 990
FGFR4, 2264
GRB7, 2886
CDH3, 1001
EGFR, 1956
CCNE1, 898
PHGDH, 26227
CDCA1, 83540
KNTC2, 10403
TYMS, 7298
RRM2, 6241
MKI67, 4288
CENPF, 1063
ORC6L, 23594
ANLN, 54443
BIRC5, 332
CDC20, 991
KIF2C, 11004
CEP55, 55165
EXO1, 9156
MELK, 9833
PTTG1, 9232
UBE2C, 11065
CCNB1, 891
UBE2T, 29089
Basal-like
HER2-enriched
Luminal A
Luminal B
0
5

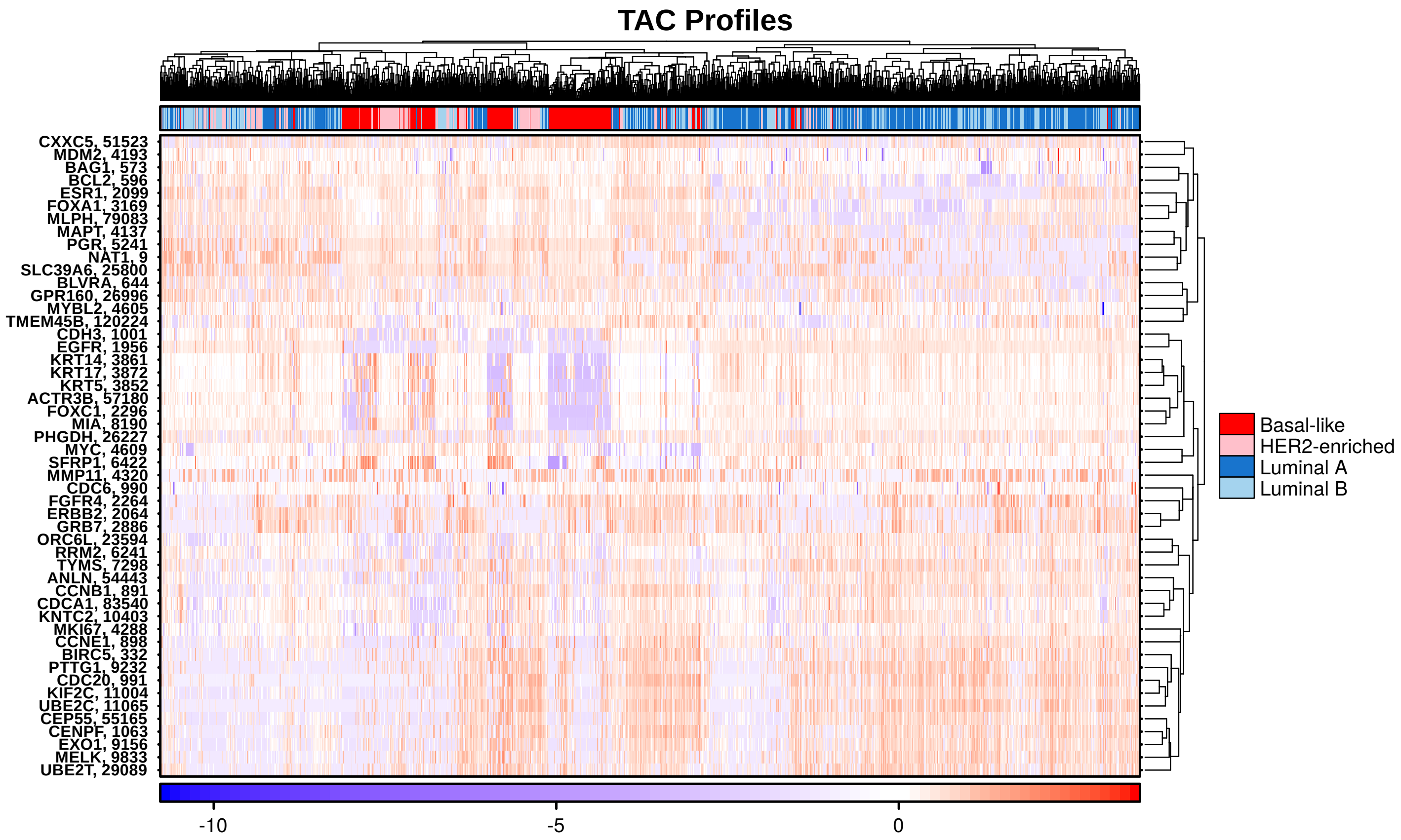

12 Supplementary Figure 4D - *BCL2* mRNA abundance per subtype

Set up the environment

```
library(BoutrosLab.plotting.general);
library(yaml);
dataset.name <- 'Metabric';
```

Yaml file name

```
yaml.file <- paste0(dataset.name,'.yaml');
```

Read yaml with file information

```
dataset.files <- yaml.load_file(yaml.file);
```

Load clinical annotation

```
patient.anno <- read.table(dataset.files$clinical.annotation.file,sep='\t',header=TRUE);
```

Load mRNA abundance profiles

```
profile.types <- c('bulk','tc','tac');
rna.data.matrix <- list();
for(profile.type in profile.types) {
```

```
  rna.data.matrix[[profile.type]] <- read.table(
    dataset.files[[paste0(profile.type,'.mrna.abundance.file')]],
    header=TRUE,
    sep='\t'
    );
  }
patient.anno <- patient.anno[colnames(rna.data.matrix$tc),];
```

Load PAM50 gene list

```
pam50.genes <- read.table(dataset.files$pam50.gene.list.file,sep='\t',header=TRUE,row.names=1);
pam50.genes$probe <- pam50.genes$EntrezID;
rownames(pam50.genes) <- as.character(pam50.genes$probe);
```

Subtype colour scheme

```
subtype.colours <- c('red','pink','dodgerblue3','lightskyblue2');
names(subtype.colours) <- c('Basal','Her2','LumA','LumB');
```

BCL2 analysis

```
for(profile.type in profile.types) {
  new.col <- t(rna.data.matrix[[profile.type]]['596_at',]);
  colnames(new.col) <- paste0('BCL2.mRNA.',profile.type);
  patient.anno[,paste0('BCL2.mRNA.',profile.type)] <- new.col;
  }
boxplot.ylimits <- list(
  bulk=c(-4,4),
  tc=c(-3,3),
  tac=c(-4,4)
  );
boxplots <- list();
for(profile.type in c('tac','tc','bulk')) {
  for(gene in 'BCL2') {
    boxplots <- append(boxplots,list(create.boxplot(
      mRNA ~ subtype,
      data.frame(
        mRNA = scale(as.numeric(
          patient.anno[patient.anno$Pam50Subtype %in% names(subtype.colours),
          paste0(gene,'.mRNA.',profile.type)]
          )),
        subtype = factor(
          patient.anno[patient.anno$Pam50Subtype %in% names(subtype.colours),'Pam50Subtype']
          )
        ),
      add.stripplot = TRUE,
      points.col = subtype.colours[sort(names(subtype.colours))][as.numeric(factor(
        patient.anno[patient.anno$Pam50Subtype %in% names(subtype.colours),'Pam50Subtype']
        ))],
      ylimits=boxplot.ylimits[[profile.type]],
```

```
      abline.h=c(-4,-2,0,2,4),
      abline.lty=2,
      abline.col='gray50'
      )));
    }
  }
create.multiplot(
  rev(boxplots),
  filename = './sfigure4d.pdf',
  resolution = 100,
  plot.layout=c(3,1),
  width=10,
  height=3,
  y.relation='same',
  xaxis.labels=NULL,
  ylimits = c(-3.5,3.5),
  yat=c(-2,0,2),
  xlab.label = c('Bulk','TC','TAC'),
  ylab.label = 'BCL2',
  main = 'Scaled mRNA Abundance            ',
  right.padding = 30,
  key = list(
    text = list(
      lab = c('Basal-like','HER2-enriched','Luminal A','Luminal B'),
      cex=2
      ),
    points = list(
      pch = 19,
      col = subtype.colours[sort(names(subtype.colours))]
      ),
    x = 1,
    y = 1,
    padding.text = 2
    ),
  use.legacy.settings=TRUE
  );
```

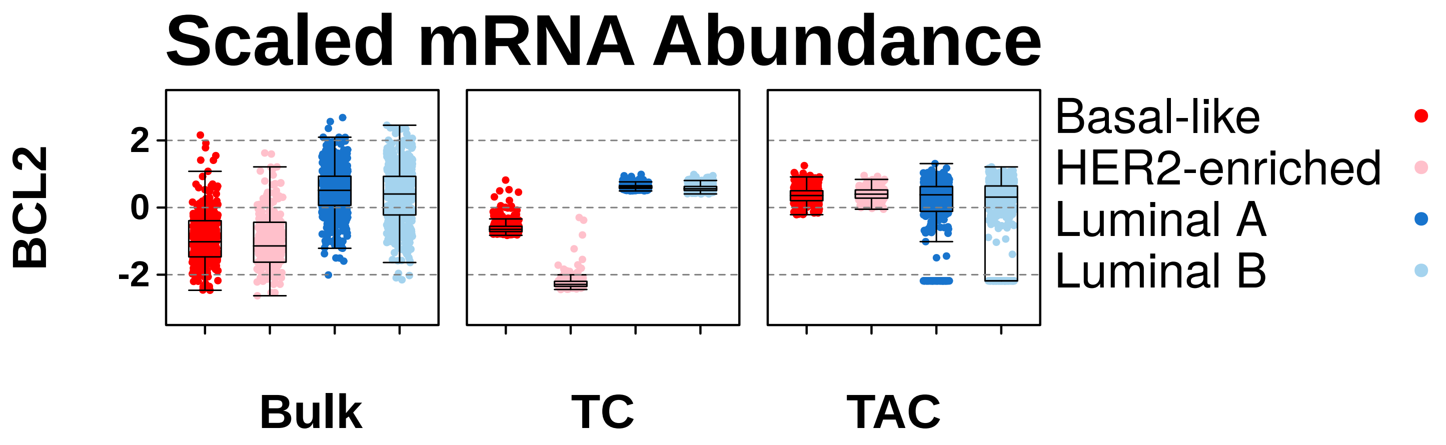

13 Supplmentary Figure 5A - Genes with TAC mRNA abundance associated with subtypes

Set up the environment

```
library(BoutrosLab.plotting.general);
library(limma);
library(yaml);
dataset.name <- 'Metabric';
```

Yaml file name

```
yaml.file <- paste0(dataset.name,'.yaml');
```

Read yaml with file information

```
dataset.files <- yaml.load_file(yaml.file);
```

Load clinical annotation

```
patient.anno <- read.table(dataset.files$clinical.annotation.file,sep='\t',header=TRUE);
```

Load mRNA abundance profiles

```
profile.types <- c('tc','tac');
rna.data.matrix <- list();
for(profile.type in profile.types) {
  rna.data.matrix[[profile.type]] <- read.table(
    dataset.files[[paste0(profile.type,'.mrna.abundance.file')]],
    header=TRUE,
    sep='\t'
    );
  }
```

Match up the patients/remove normal-like patients

```
common.patients <- intersect(colnames(rna.data.matrix$tc),rownames(patient.anno));
patient.anno <- patient.anno[common.patients,];
for(profile.type in profile.types) {
  rna.data.matrix[[profile.type]] <- rna.data.matrix[[profile.type]][,common.patients];
  }
patient.anno$Pam50Subtype <- as.character(patient.anno$Pam50Subtype);
```

Load gene symbols

```
mrna.conversion.info <- read.table(dataset.files$gene.symbol.file,header=TRUE,sep='\t');
```

Run differential mRNA abundance analysis to determine which genes associate with each subtype

```r
limma.results <- list();
for(subtype in c('Basal','Her2','LumA','LumB')) {
  targets <- rep(NA,ncol(rna.data.matrix$tac));
  targets[patient.anno$Pam50Subtype == subtype] <- 'subtype';
  targets[patient.anno$Pam50Subtype != subtype] <- 'not';
  gtargets <- unique(targets);
  m <- match(targets,gtargets);
  design <- model.matrix(~ -1 + factor(m));
  colnames(design) <- gtargets;
  contrast.matrix <- makeContrasts(not-subtype, levels=design);
  profile <- 'deconvovled';
  mrna <- rna.data.matrix$tc;
  rownames(mrna) <- 1:nrow(mrna);
  mrna <- as.matrix(mrna);
  fit <- lmFit(mrna, design);
  fit2 <- contrasts.fit(fit, contrast.matrix);
  fit3 <- eBayes(fit2);
  tc.top1 <- topTable(fit3,coef=1,number=nrow(fit3));
  tc.top1$GeneID <- rownames(rna.data.matrix$tc)[as.numeric(rownames(tc.top1))];
  profile <- 'tac';
  mrna <- rna.data.matrix[[profile]];
  rownames(mrna) <- 1:nrow(mrna);
  mrna <- as.matrix(mrna);
  fit <- lmFit(mrna, design);
  fit2 <- contrasts.fit(fit, contrast.matrix);
  fit3 <- eBayes(fit2);
  top1 <- topTable(fit3,coef=1,number=nrow(fit3));
  top1$GeneID <- rownames(rna.data.matrix[[profile]])[as.numeric(rownames(top1))];
  limma.results[[subtype]] <- top1;
  }
```

Select 10 genes associated with each subtype, try to maximize effect size and minimize p-values

```r
tac50genes <- c();
tac50sym <- c();
for(subtype in c('Basal','Her2','LumA','LumB')) {
  for(x in 10:100) {
    selected.genes <- intersect(
      limma.results[[subtype]]$GeneID[order(abs(limma.results[[subtype]]$logFC))[
        (nrow(limma.results[[subtype]])-x):nrow(limma.results[[subtype]])
        ]],
      limma.results[[subtype]]$GeneID[order(limma.results[[subtype]]$adj.P.Val)[1:x]]
      );
    selected.sym <- as.character(mrna.conversion.info$GeneSymbol)[
      paste0(as.character(mrna.conversion.info$EntrezID),'_at') %in% as.character(selected.genes)
      ];
    selected.genes.converted <- as.character(mrna.conversion.info$EntrezID[
      as.character(mrna.conversion.info$GeneSymbol) %in% as.character(selected.sym)
      ]);
    if(length(selected.sym) >= 10) {
```

```
      selected.genes <- limma.results[[subtype]][
        limma.results[[subtype]]$GeneID %in% paste0(selected.genes.converted,'_at'),
        'GeneID'
        ][1:10];
      selected.sym <- as.character(mrna.conversion.info$GeneSymbol)[
        paste0(as.character(mrna.conversion.info$EntrezID),'_at') %in% as.character(selected.genes)
        ];
      selected.genes.converted <- as.character(mrna.conversion.info$EntrezID[
        as.character(mrna.conversion.info$GeneSymbol) %in% as.character(selected.sym)
        ]);
      tac50genes <- c(tac50genes,selected.genes.converted);
      tac50sym <- c(tac50sym,selected.sym);
      break;
      }
    }
  }
tac50genes <- paste0(unique(tac50genes),'_at');
tac50sym <- unique(tac50sym);
pam50.data.matrix <- list();
gene.profile.averages <- list();
for(profile.type in profile.types) {
  pam50.data.matrix[[profile.type]] <- rna.data.matrix[[profile.type]][tac50genes,];
  gene.profile.averages[[profile.type]] <- sapply(
    c('Basal-like','HER2-enriched','Luminal A','Luminal B'),
    function(stype) {
      stype.patients <- which(
        colnames(rna.data.matrix[[profile.type]])
        %in%
        patient.anno$patient_id[patient.anno$subtype == stype]
        );
      rowSums(t(scale(t(pam50.data.matrix[[profile.type]])))[,stype.patients])/length(stype.patients)
      }
    );
  colnames(gene.profile.averages[[profile.type]]) <- paste(
    profile.type,
    colnames(gene.profile.averages[[profile.type]])
    );
  }
heatmap.data <- cbind(gene.profile.averages$tac,gene.profile.averages$tc);
rownames(heatmap.data) <- tac50sym;
heatmap.reordered <- NULL;
for(i in seq(1,nrow(heatmap.data),10)) {
  selected.rows <- i:min((i+9),nrow(heatmap.data));
  heatmap.reordered <- rbind(
    heatmap.reordered,
    heatmap.data[selected.rows[order.dendrogram(create.dendrogram(t(heatmap.data[selected.rows,])))],
    );
  }
```

Create the covariate legend

```
cov.legend <- list(
  legend = list(
    colours = c('dodgerblue','gold'),
    labels = c('TC','TAC'),
    title='Profile Type'
    ),
  legend = list(
    colours = c('red','pink','dodgerblue3','lightskyblue2'),
    labels = c('Basal-like','HER2-enriched','Luminal A','Luminal B'),
    title='PAM50 Subtype'
    )
  );
```

Create the covariate bar for the top of the TAC heatmap

```
profile.cov <- list(
  rect=list(
    col='transparent',
    fill=rep(c('red','pink','dodgerblue3','lightskyblue2'),3)[1:4],
    lwd=1.5
    ),
  rect=list(
    col='transparent',
    fill=rep(c('gold','dodgerblue','darkred'),each=4)[1:4],
    lwd=1.5
    )
  );
```

Create the TAC heatmap

```
create.heatmap(
  heatmap.reordered[,1:4],
  filename = './sfigure5a_left.pdf',
  colour.scheme=c('blue','white','red'),
  cluster.dimensions='none',
  resolution=200,
  same.as.matrix=TRUE,
  yaxis.lab=NA,
  yaxis.cex=0.75,
  colour.alpha=1,
  colourkey.cex=1,
  covariates.top=profile.cov,
  covariate.legend=cov.legend,
  legend.side='right',
  width=4.5,
  height=7,
  legend.title.just='left',
  style = 'BoutrosLab',
  at=seq(-2.9,2.9,0.05),
  use.legacy.settings=TRUE
  );
```

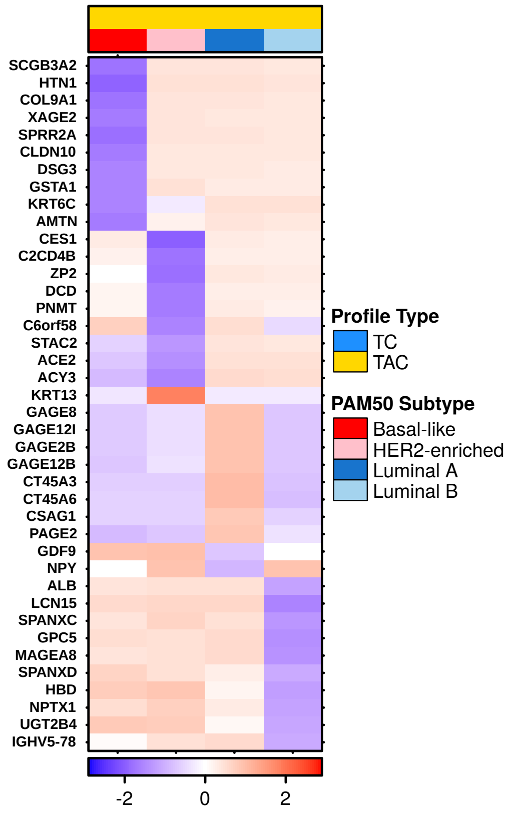

Create the covariate for the top of the TC heatmap

```
profile.cov <- list(
  rect=list(
    col='transparent',
    fill=rep(c('red','pink','dodgerblue3','lightskyblue2'),3)[5:8],
    lwd=1.5
    ),
  rect=list(
    col='transparent',
    fill=rep(c('gold','dodgerblue','darkred'),each=4)[5:8],
    lwd=1.5
    )
  );
```

Create the covariate for the TC heatmap

```
create.heatmap(
  heatmap.reordered[,5:8],
  filename = './sfigure5a_right.pdf',
  colour.scheme=c('blue','white','red'),
  cluster.dimensions='none',
  resolution=200,
  same.as.matrix=TRUE,
  yaxis.lab=NA,
  yaxis.cex=0.75,
  colour.alpha=1,
  colourkey.cex=1,
```

```
  covariates.top=profile.cov,
  covariate.legend=cov.legend,
  legend.side='right',
  width=4.5,
  height=7,
  legend.title.just='left',
  style = 'BoutrosLab',
  at=seq(-2.9,2.9,0.05),
  use.legacy.settings=TRUE
  );
```

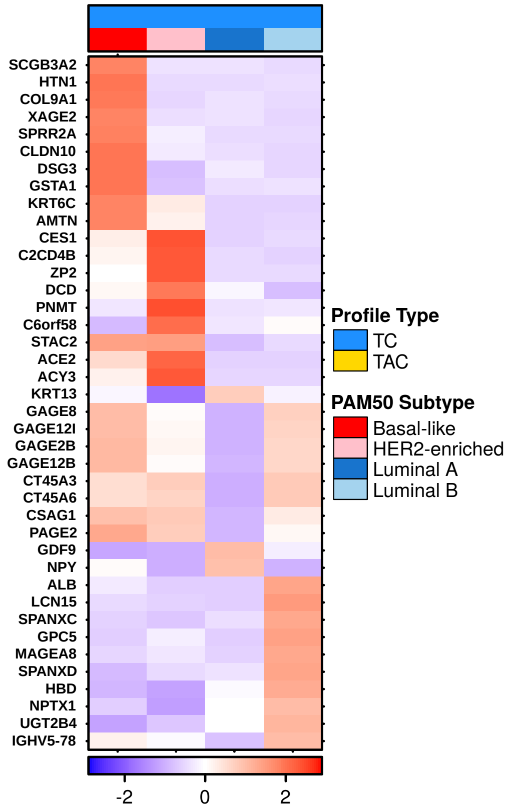

14 Supplementary Figure 5B-F - Subtype differences between TC-TAC correlations

Set up the environment

```
library(BoutrosLab.plotting.general);
library(yaml);
dataset.name <- 'Metabric';
```

Yaml file name

```
yaml.file <- paste0(dataset.name,'.yaml');
```

Read yaml with file information

```
dataset.files <- yaml.load_file(yaml.file);
```

Load clinical annotation

```
patient.anno <- read.table(dataset.files$clinical.annotation.file,sep='\t',header=TRUE);
```

Load mRNA abundance profiles

```
profile.types <- c('bulk','tc','tac');
rna.data.matrix <- list();
for(profile.type in profile.types) {
  rna.data.matrix[[profile.type]] <- read.table(
    dataset.files[[paste0(profile.type,'.mrna.abundance.file')]],
    header=TRUE,
    sep='\t'
    );
  }
patient.anno <- patient.anno[patient.anno$patient_id %in% colnames(rna.data.matrix$tc),];
patient.anno$Pam50Subtype <- as.character(patient.anno$Pam50Subtype);
```

Load gene names

```
gene.sym.mapping <- read.table(dataset.files$gene.symbol.file,sep='\t',header=TRUE);
```

Correlate TC and TAC mRNA abundance

```
tc.tac.cor <- matrix(NA,ncol=length(unique(patient.anno$Pam50Subtype)),nrow=nrow(rna.data.matrix$tac)
colnames(tc.tac.cor) <- unique(patient.anno$Pam50Subtype);
tc.tac.cor.p <- matrix(NA,ncol=length(unique(patient.anno$Pam50Subtype)),nrow=nrow(rna.data.matrix$ta
colnames(tc.tac.cor.p) <- unique(patient.anno$Pam50Subtype);
for(i in 1:nrow(rna.data.matrix$tac)) {
  for(subtype in unique(patient.anno$Pam50Subtype)) {
    cor.result <- cor.test(
      as.numeric(rna.data.matrix$tac[
        i,
        as.character(patient.anno$patient_id[patient.anno$Pam50Subtype == subtype])
        ]),
      as.numeric(rna.data.matrix$tc[
        i,
        as.character(patient.anno$patient_id[patient.anno$Pam50Subtype == subtype])
        ]),
      method='spearman'
      );
    tc.tac.cor[i,subtype] <- cor.result$estimate;
    tc.tac.cor.p[i,subtype] <- cor.result$p.value;
    }
  }
rownames(tc.tac.cor) <- rownames(rna.data.matrix$tac);
rownames(tc.tac.cor.p) <- rownames(rna.data.matrix$tac);
```

Plot all the genes with a correlation difference greater than 0.4 between any Pam50Subtypes

```
heatmap.data <- tc.tac.cor[(apply(tc.tac.cor,1,max) - apply(tc.tac.cor,1,min)) > 0.4
  & !apply(is.na(tc.tac.cor),1,any),sort(colnames(tc.tac.cor))];
create.heatmap(
  heatmap.data,
  filename = './sfigure5b.pdf',
  resolution=150,
  same.as.matrix = TRUE,
  xaxis.lab=NA,
  at = seq(-1,1,0.01),
  colourkey.labels.at=c(-1,0,1),
  colourkey.labels=c('-1','0','1'),
  colour.scheme=c('red','white','blue'),
  height=12.5,
  print.colour.key=FALSE,
  use.legacy.settings = TRUE
  );
```

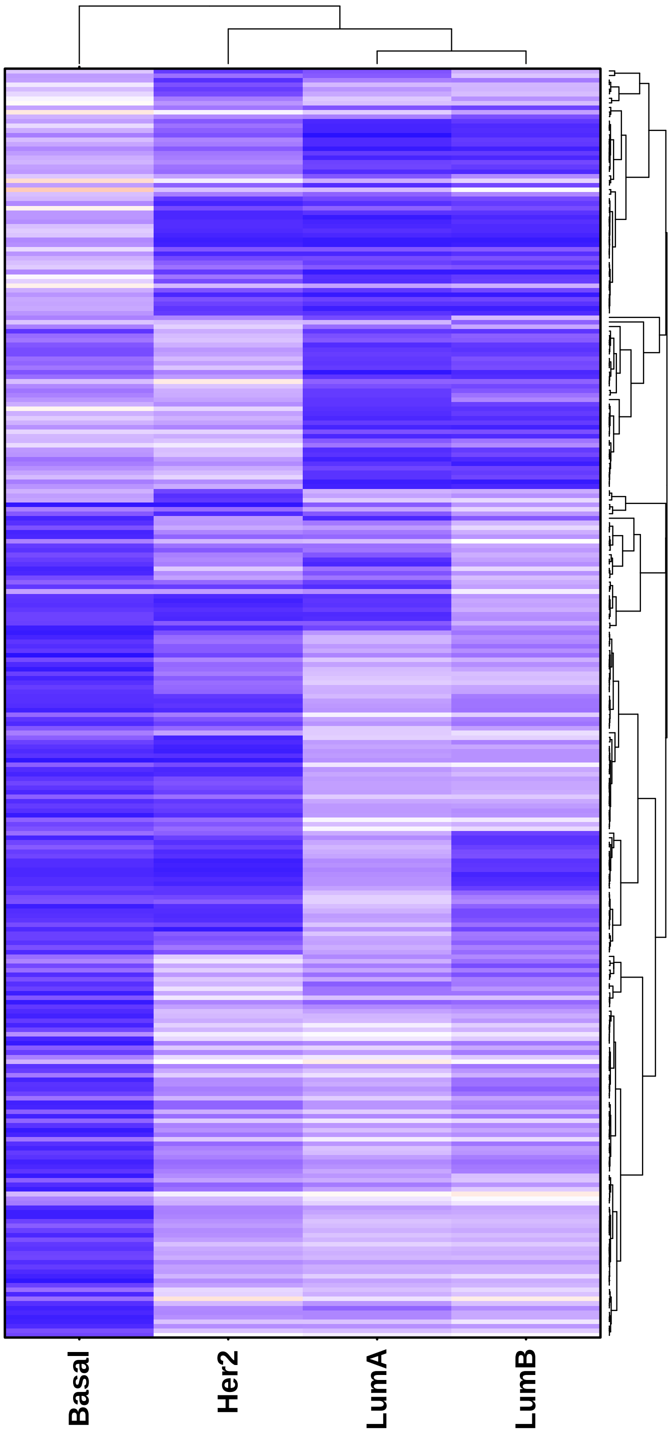

Plot the genes with different correlation compared to all other Pam50Subtypes for each Pam50Subtype

```
xaxis.lab.length <- 18;
for(subtype in setdiff(unique(patient.anno$Pam50Subtype),'unknown')) {
  selected.genes <- which(apply(
    abs(tc.tac.cor[,setdiff(colnames(tc.tac.cor),subtype)] - tc.tac.cor[,subtype]),
    1,
    min
    ) > 0.4);
  if(length(selected.genes) > 1) {
```

```
    gene.id <- sub('_at','',rownames(rna.data.matrix$tac)[selected.genes]);
    gene.sym <- as.character(gene.sym.mapping$GeneSymbol[match(gene.id, gene.sym.mapping$EntrezID)]);
    if(nchar(paste0(gene.sym[1],', ',gene.id[1])) < xaxis.lab.length) {
      gene.sym[1] <- paste(
        c(rep(' ',2*(xaxis.lab.length-nchar(paste0(gene.sym[1],', ',gene.id[1])))),gene.sym[1]),
        collapse=''
        );
      }
    create.heatmap(
      tc.tac.cor[selected.genes,sort(colnames(tc.tac.cor))],
      filename = paste0('./sfigure5cdef_',subtype,'.pdf'),
      resolution=100,
      same.as.matrix = TRUE,
      cluster.dimensions = 'rows',
      yaxis.lab=paste0(gene.sym,', ',gene.id),
      yat=seq(1,length(selected.genes)),
      xaxis.lab=NA,
      at = seq(-1,1,0.01),
      colourkey.labels.at=c(-1,0,1),
      colourkey.labels=c('-1','0','1'),
      colour.scheme=c('red','white','blue'),
      height=0.25*length(selected.genes)+2,
      use.legacy.settings = TRUE
      );
    }
  }
```

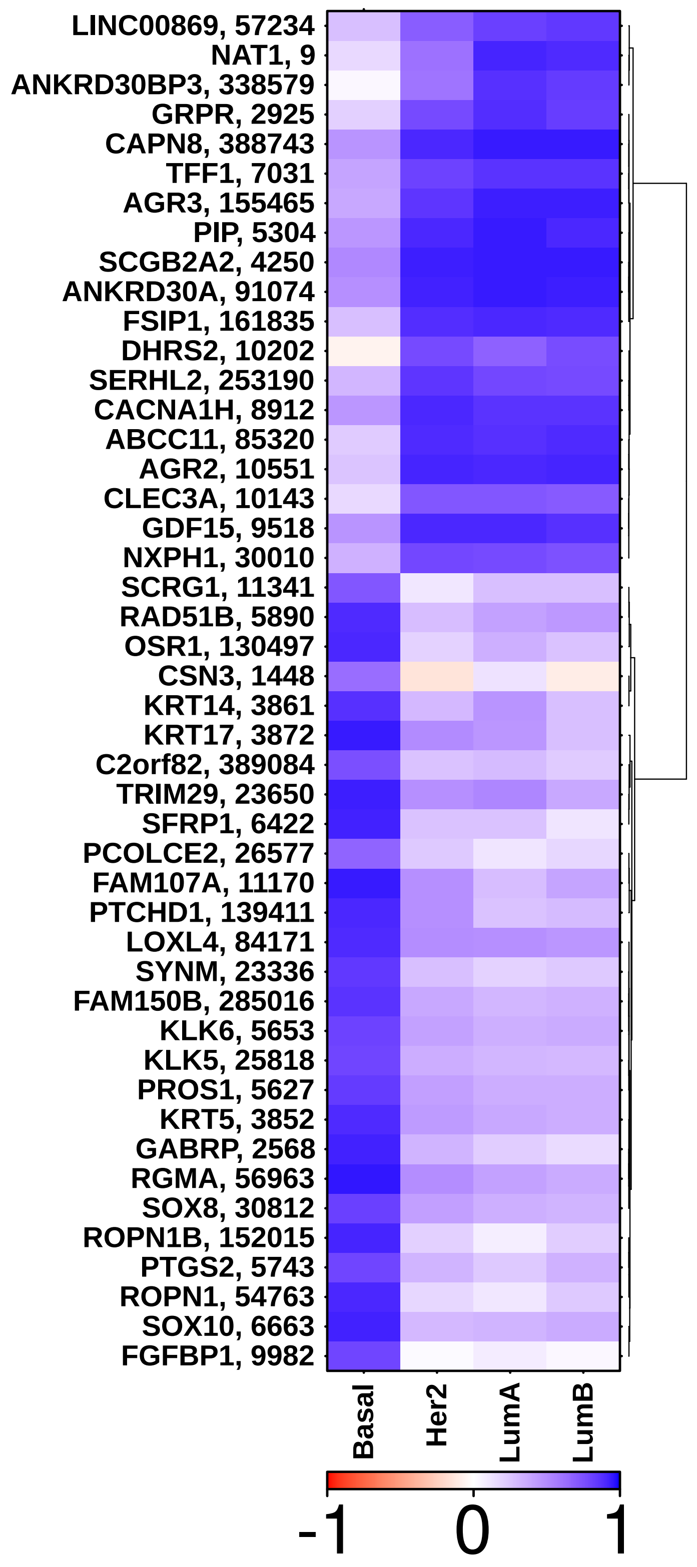
LINC00869, 57234
NAT1, 9
ANKRD30BP3, 338579
GRPR, 2925
CAPN8, 388743
TFF1, 7031
AGR3, 155465
PIP, 5304
SCGB2A2, 4250
ANKRD30A, 91074
FSIP1, 161835
DHRS2, 10202
SERHL2, 253190
CACNA1H, 8912
ABCC11, 85320
AGR2, 10551
CLEC3A, 10143
GDF15, 9518
NXPH1, 30010
SCRG1, 11341
RAD51B, 5890
OSR1, 130497
CSN3, 1448
KRT14, 3861
KRT17, 3872
C2orf82, 389084
TRIM29, 23650
SFRP1, 6422
PCOLCE2, 26577
FAM107A, 11170
PTCHD1, 139411
LOXL4, 84171
SYNM, 23336
FAM150B, 285016
KLK6, 5653
KLK5, 25818
PROS1, 5627
KRT5, 3852
GABRP, 2568
RGMA, 56963
SOX8, 30812
ROPN1B, 152015
PTGS2, 5743
ROPN1, 54763
SOX10, 6663
FGFBP1, 9982
Basal
Her2
LumA
LumB
-1
0
1

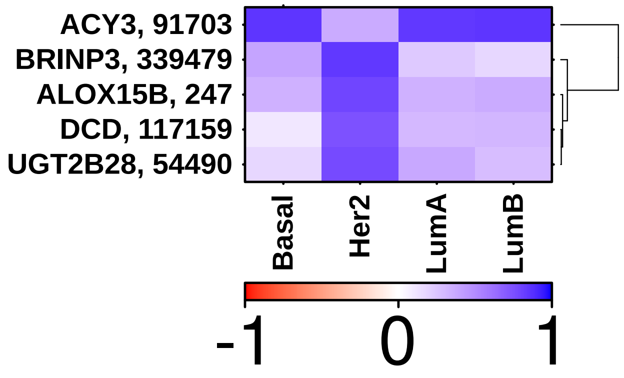
ACY3, 91703
BRINP3, 339479
ALOX15B, 247
DCD, 117159
UGT2B28, 54490
Basal
Her2
LumA
LumB
-1
0
1

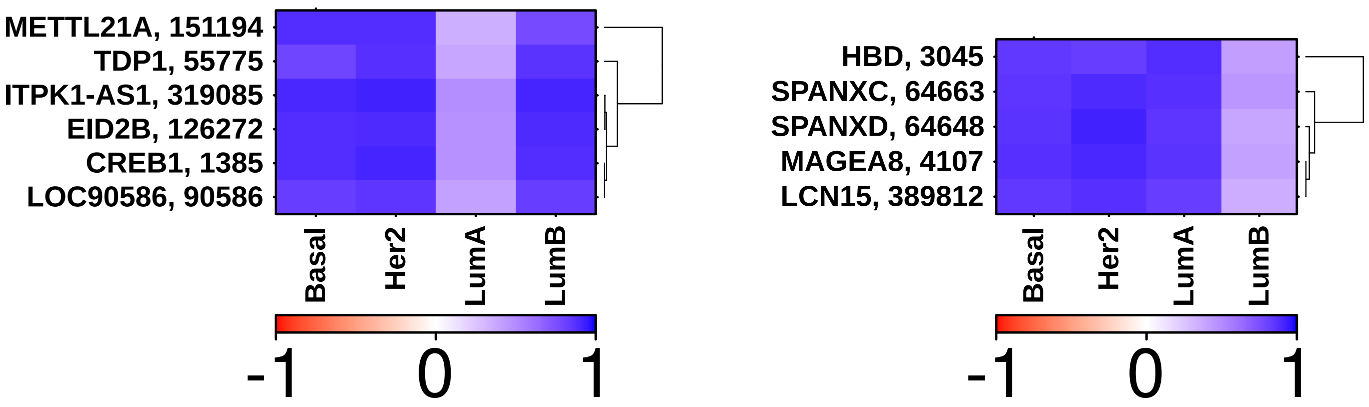

Create a file containing the differentially correlated genes

```
max.diff <- apply(
  tc.tac.cor,
  1,
  function(x) { max(abs(c(x[1] - x[2],x[1] - x[3],x[1] - x[4],x[2] - x[3],x[2] - x[4],x[3]-x[4])))}
  );
rownames(tc.tac.cor) <- rownames(rna.data.matrix$tac);
diff.genes <- round(tc.tac.cor[which(!is.na(max.diff) & max.diff > 0.4),],digits=5);
gene.id <- rownames(diff.genes);
gene.sym <- as.character(gene.sym.mapping$GeneSymbol[match(gene.id, gene.sym.mapping$EntrezID)]);
diff.genes <- cbind(diff.genes,gene.sym);
colnames(diff.genes)[5] <- 'symbol';
write.table(
  diff.genes,
  sep='\t',
  row.names=TRUE,
  col.names=TRUE,
  quote=FALSE,
  file=paste0('./',dataset.name,'_tac_tc_mrna_correlation_diff_gene_list.txt')
  );
```

15 Supplementary Figure 5G - Subtype labeling for profile clusters

Set up the environment

```
library(BoutrosLab.plotting.general);
library(yaml);
dataset.name <- 'Metabric';
profile.types <- c('bulk','tc','tac');
```

Yaml file name

```
yaml.file <- paste0(dataset.name,'.yaml');
```

Read yaml with file information

```
dataset.files <- yaml.load_file(yaml.file);
```

Load clinical annotation

```
patient.anno <- read.table(dataset.files$clinical.annotation.file,sep='\t',header=TRUE);
patient.anno <- patient.anno[which(patient.anno$subtype != 'unknown'),];
cluster.num <- 4;
```

Create list of all possible ways that the subtypes can be assigned to the clusters

```
possible.assignment.orders <- matrix(NA,ncol=4,nrow=24);
matrix.idx <- 1;
for(i1 in 1:4) {
  for(i2 in setdiff(1:4,i1)) {
    for(i3 in setdiff(1:4,c(i1,i2))) {
      for(i4 in setdiff(1:4,c(i1,i2,i3))) {
        possible.assignment.orders[matrix.idx,] <- c(i1,i2,i3,i4);
        matrix.idx <- matrix.idx+1;
        }
      }
    }
  }
```

Subtype colour scheme

```
subtype.options <- c('Basal-like','HER2-enriched','Luminal A','Luminal B');
subtype.col <- c('red','pink','dodgerblue3','lightskyblue2');
barplot.data <- list(
  cluster=rep(1:cluster.num,each=length(subtype.options)),
  subtype=rep(subtype.options,cluster.num)
  );
barplot.data$group <- paste(barplot.data$cluster,barplot.data$subtype,sep=':');
barplot.data <- as.data.frame(barplot.data);
```

Create a barplot for each profile type

```
plots <- list();
xaxis.labels <- list();
patient.assignments <- list();
for(profile.type in profile.types) {
  # load results from clustering profiles
  filename <- paste0(
    sub(paste0('/plots/',dataset.name,'/'),'',dataset.files$parent.output.dir),
    '/results/clustering/',
    dataset.name,
    '_',
    profile.type,
    '_ConsensusClusterPlus/',
    dataset.name,
    '_',
```

```
    profile.type,
    '_ConsensusClusterPlus.k=',
    cluster.num,
    '.consensusClass.csv'
    );
  cluster.results <- read.csv(
    filename,
    header=FALSE
    );
  colnames(cluster.results) <- c('patient','cluster');
  rownames(cluster.results) <- cluster.results$patient;
  cluster.results <- cluster.results[sort(rownames(cluster.results)),];
  # filter to patients that were in the dataset and have subtype information
  intersecting.patients <- intersect(rownames(cluster.results),rownames(patient.anno));
  cluster.results <- cluster.results[intersecting.patients,];
  cluster.results$subtype <- patient.anno[intersecting.patients,'subtype'];
  # initialize the right size for the barplot data
  barplot.data <- cbind(barplot.data, rep(0,cluster.num*length(subtype.options)));
  colnames(barplot.data)[ncol(barplot.data)] <- paste0('count_',profile.type);
  # add the counts for the number of patients from each subtype in each cluster to the barplot data
  barplot.data[
    match(names(table(paste(cluster.results$cluster,cluster.results$subtype,sep=':'))),barplot.data$g
    paste0('count_',profile.type)
    ] <- as.numeric(table(paste(cluster.results$cluster,cluster.results$subtype,sep=':')));
  # decide which subtype corresponses to each cluster
  subtype.cat <- c('Basal-like','HER2-enriched','Luminal A','Luminal B');
  possible.count.correct <- apply(
    possible.assignment.orders,
    1,
    function(x) {sum(barplot.data[as.character(barplot.data$subtype) == subtype.cat[x][barplot.data$c
    );
  cluster.subtype.allocations <- subtype.cat[
    possible.assignment.orders[which(possible.count.correct == max(possible.count.correct)),]
    ];
  barplot.data <- cbind(barplot.data, cluster.subtype.allocations[barplot.data$cluster]);
  colnames(barplot.data)[ncol(barplot.data)] <- paste0('cluster_class_',profile.type);
  patient.assignments[[paste0('cluster_class_',profile.type)]] <- cluster.subtype.allocations[cluster
  bars.order <- c();
  for(clust in 1:cluster.num) {
    clust.rows <- which(barplot.data$cluster == clust);
    clust.bars.order <- rev(order(barplot.data[clust.rows,paste0('count_',profile.type)]));
    bars.order <- c(
      bars.order,
      c(
        clust.bars.order[
          barplot.data$subtype[clust.rows][clust.bars.order] == cluster.subtype.allocations[clust]
          ],
        clust.bars.order[
          barplot.data$subtype[clust.rows][clust.bars.order] != cluster.subtype.allocations[clust]
          ])+(clust-1)*length(unique(barplot.data$subtype))
```

```
      );
    }
  # create the barplot showing subtype distribution across the clusters
  yaxis.lab <- rep('',cluster.num);
  if(profile.type == 'bulk') {
    yaxis.lab <- seq(0,800,200);
    }
  plots[[profile.type]] <- create.barplot(
    count ~ cluster,
    data.frame(
      count=barplot.data[,paste0('count_',profile.type)][bars.order],
      cluster=as.factor(barplot.data$cluster)[bars.order]
      ),
    stack=TRUE,
    groups=1:nrow(barplot.data),
    col=subtype.col[as.numeric(as.factor(barplot.data$subtype))[bars.order]],
    xaxis.lab=paste0('(',1:4,') ',cluster.subtype.allocations),
    xaxis.rot=90,
    ylimits=c(-10,810),
    yat=seq(0,800,200),
    yaxis.lab=yaxis.lab
    );
  xaxis.labels[[profile.type]] <- cluster.subtype.allocations;
  }
```

Combine the barplots to make the final figure

```
create.multiplot(
  plots,
  filename = './sfigure5g.pdf',
  ylab.label='Number of patients',
  xlab.label=paste(sub('tac','TAC',sub('bulk','Bulk',sub('tc','TC',profile.types))),'cluster'),
  resolution=200,
  width=11,
  height=7,
  print.new.legend=TRUE,
  plot.layout=c(3,1),
  xaxis.rot=90,
  xaxis.cex=1.5,
  yaxis.cex=1.5,
  x.relation='free',
  xaxis.labels=list(
    plots$bulk$x.scales$labels,
    plots$tc$x.scales$labels,
    plots$tac$x.scales$labels
    ),
  legend = list(
    right = list(
      fun = legend.grob(
        legends = list(
```

```
          legend = list(
            colours = subtype.col,
            labels = levels(as.factor(barplot.data$subtype)),
            title = 'Subtype'
            )
          ),
        label.cex=1.5,
        title.just = 'left',
        title.cex=1.5
        )
      )
    ),
  use.legacy.settings=TRUE
  );
```

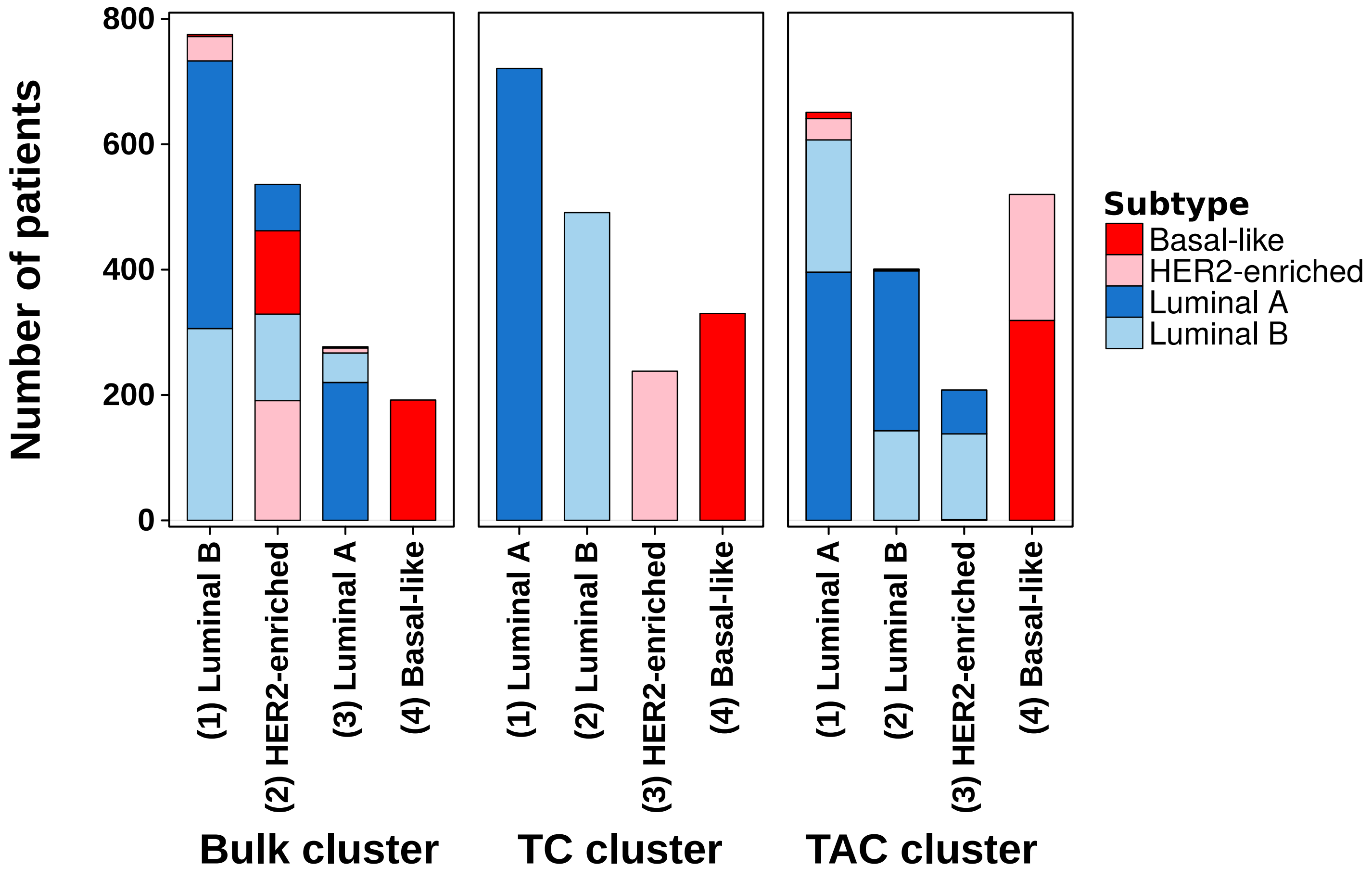

16 Running Univariate Survival Analysis

Set up the environment

```
library(BoutrosLab.plotting.survival);
library(yaml);
args <- commandArgs(trailingOnly = TRUE);
dataset.name <- 'Metabric';
```

```
profile.type <- args[1];
row.start <- as.numeric(args[2]);
row.end <- as.numeric(args[3]);
unexpressed.threshold <- 6.5;
num.rows <- length(row.start:row.end);
```

Yaml file name

```
yaml.file <- paste0(dataset.name,'.yaml');
```

Read yaml with file information

```
dataset.files <- yaml.load_file(yaml.file);
```

Load clinical annotation

```
survival.anno <- read.table(dataset.files$clinical.annotation.file,sep='\t',header=TRUE);
```

Load mRNA abundance profiles

```
profiles <- read.table(dataset.files[[paste0(profile.type,'.mrna.abundance.file')]],header=TRUE,sep='
```

Filter the survival data to match the mRNA data

```
patients <- intersect(colnames(profiles), rownames(survival.anno));
survival.anno <- survival.anno[patients,];
survival.anno <- survival.anno[!is.na(survival.anno$TimeToEvent),];
```

Filter mRNA data to match the survival data

```
profiles <- profiles[,rownames(survival.anno)];
```

Truncate follow-up and set up vectors for the survival object

```
time.to.event <- survival.anno$TimeToEvent[!is.na(survival.anno$TimeToEvent)];
event <- survival.anno$Event[!is.na(survival.anno$Event)];
```

Run univariate cox modelling for profiles independently

```
result.colnames <- c('coxzph.pvalue','dichotomized.mrna.value','num.patients.low','num.patients.high'
gene.survival <- matrix(NA, ncol=length(result.colnames),nrow=num.rows);
colnames(gene.survival) <- result.colnames;
for(i in row.start:row.end) {
  split <- median(as.numeric(profiles[i,]));
  patient.groups <- factor((as.numeric(profiles[i,]) > unexpressed.threshold),levels=c(FALSE,TRUE));
  if(split < unexpressed.threshold & sum(event[patient.groups == FALSE] == 1) > 0 & sum(event[patient
    split <- unexpressed.threshold;
    }
  patient.groups <- factor((as.numeric(profiles[i,]) > split),levels=c(FALSE,TRUE));
  survival.object <-Surv(time.to.event, event);
```

```
  gene.survival[i-row.start+1,'dichotomized.mrna.value'] <- split;
  gene.survival[i-row.start+1,'num.patients.low'] <- sum(patient.groups == FALSE);
  gene.survival[i-row.start+1,'num.patients.high'] <- sum(patient.groups == TRUE);
  gene.survival[i-row.start+1,'num.events.low'] <- sum(event[patient.groups == FALSE] == 1);
  gene.survival[i-row.start+1,'num.events.high'] <- sum(event[patient.groups == TRUE] == 1);
  if(gene.survival[i-row.start+1,'num.events.low'] > 0 & gene.survival[i-row.start+1,'num.events.high
    # check cox proportional hazards assumptions
    cox.model <- fit.coxmodel(
      groups = patient.groups,
      survobj = survival.object,
      return.cox.model=TRUE
      );
    gene.survival[i-row.start+1,'coxzph.pvalue'] <- signif(
      BoutrosLab.statistics.survival::ph.fails(cox.model,pvalues=TRUE),
      digits=5
      );
    # run cox model
    cox.model <- fit.coxmodel(
      groups = patient.groups,
      survobj = survival.object
      );
    gene.survival[i-row.start+1,c('hr','lower.95','upper.95')] <- cox.model[1:3];
    gene.survival[i-row.start+1,'wald.pvalue'] <- signif(cox.model[4],digits=5);
    }
  if(gene.survival[i-row.start+1,'num.patients.low'] > 0 & gene.survival[i-row.start+1,'num.patients.
    # run logrank test
    logrank.stats <- BoutrosLab.statistics.survival::logrank.analysis(
      survival.object,
      patient.groups
      );
    gene.survival[i-row.start+1,'logrank.pvalue'] <- signif(logrank.stats$pvalue[1],digits=5);
    }
  }
rownames(gene.survival) <- rownames(profiles)[row.start:row.end];
```

Print these results to file to later be combined with all the other results run in parallel

```
survival.result.file <- paste0(
  dataset.files$univariate.survival.output.dir,
  dataset.name,'_',profile.type,'_survival_result_rows_',row.start,'_',row.end,'.txt'
  );
write.table(
  x = gene.survival,
  file = survival.result.file,
  sep = '\t',
  row.names = TRUE,
  col.names = TRUE,
  quote=FALSE
  );
```

17 Figure 3 - Clustering highly variant genes to recapitulate breast cancer subtypes

Set up the environment

```
library(BoutrosLab.plotting.general);
library(VennDiagram);
library(yaml);
dataset.name <- 'Metabric';
```

Yaml file name

```
yaml.file <- paste0(dataset.name,'.yaml');
```

Read yaml with file information

```
dataset.files <- yaml.load_file(yaml.file);
```

Load clinical annotation

```
patient.anno <- read.table(dataset.files$clinical.annotation.file,sep='\t',header=TRUE);
```

Load mRNA abundance profiles

```
profile.types <- c('bulk','tc','tac');
```

Load univariate survival results

```
survival.results <- list();
for(profile.type in profile.types) {
  survival.results[[profile.type]] <- read.table(
    file=paste0(
      dataset.files$univariate.survival.output.dir,
      dataset.name,
      '_',
      profile.type,
      '_gene_univariate_survival_results.txt'
      ),
    header=TRUE,
    sep='\t'
    );
  }
```

Plot univariate results

```
pval.thres <- 0.05;
hr.thres <- 0.4;
venn.title <- bquote('q < '*0.05*' & |'*log[2]*'HR| > 0.4');
```

Create a venn diagram showing overlap between genes that are significantly prognostic in the different profile types

```
venn.diagram(
  x=list(
    'Bulk'=rownames(survival.results$bulk)[
      survival.results$bulk$wald.qvalue < pval.thres &
      abs(log2(survival.results$bulk$hr)) > hr.thres &
      survival.results$bulk$coxzph.pvalue > 0.01 &
      survival.results$bulk$num.events.high >= 79
      ],
    'TC  '=rownames(survival.results$tc)[
      survival.results$tc$wald.qvalue < pval.thres &
      abs(log2(survival.results$tc$hr)) > hr.thres &
      survival.results$tc$coxzph.pvalue > 0.01 &
      survival.results$tc$num.events.high >= 79
      ],
    'TAC'=rownames(survival.results$tac)[
      !is.na(survival.results$tac$hr) &
      survival.results$tac$wald.qvalue < pval.thres &
      abs(log2(survival.results$tac$hr)) > hr.thres &
      survival.results$tac$coxzph.pvalue > 0.01 &
      survival.results$tac$num.events.high >= 79
      ]
    ),
  filename = './figure3a.pdf',
  main=venn.title,
  fill=c('red','blue','yellow'),
  fontface='bold',
  sub.fontface='bold',
  main.cex=2,
  cat.cex=1.8,
  cex=2,
  cat.pos=c(-30,30,-180)
  );

[1] 1
```

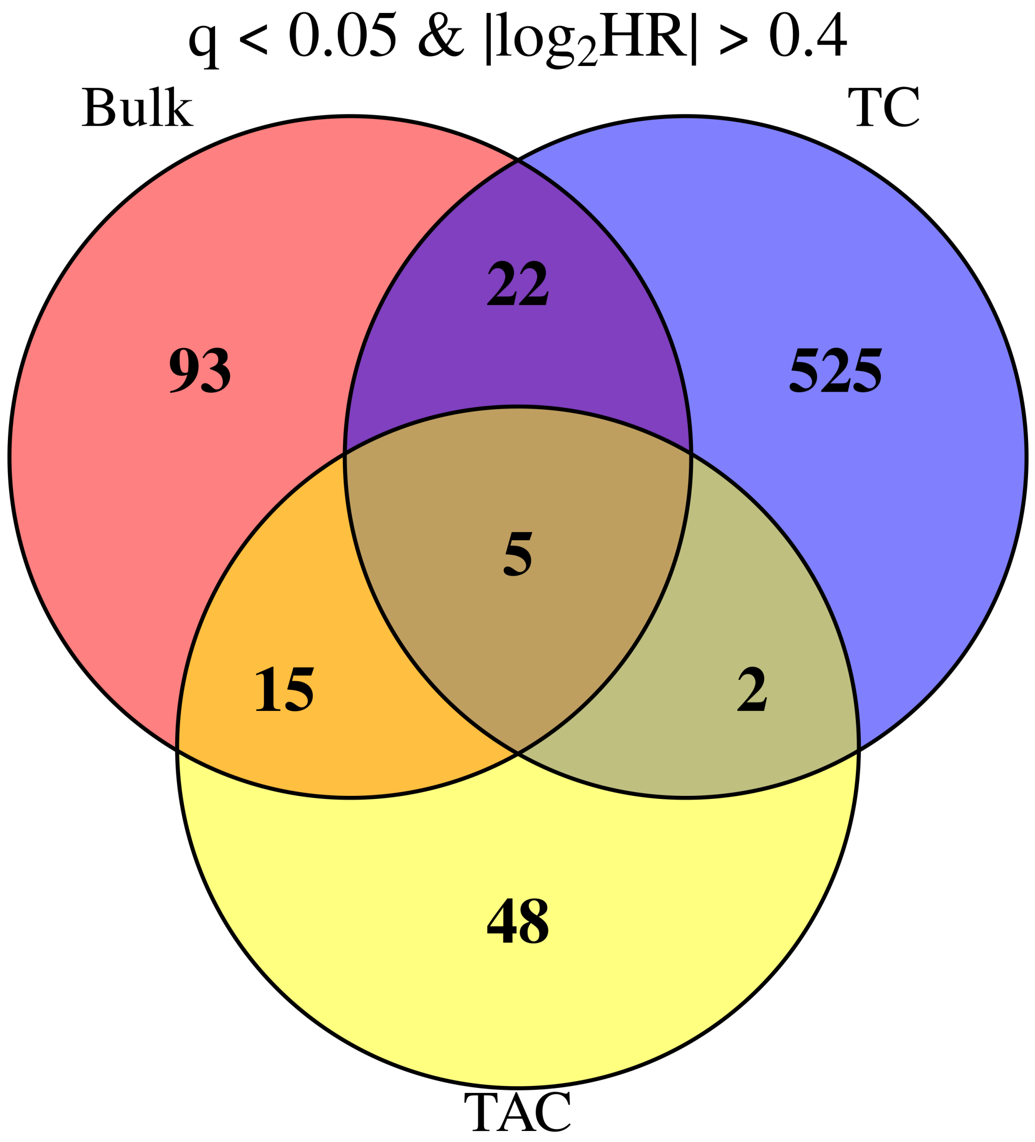

Plot pairwise comparison of the genes with significant prognosis

```
for(i in 1:(length(profile.types)-1)) {
  cond1 <- profile.types[i];
  for(j in (i+1):length(profile.types)) {
    cond2 <- profile.types[j];
    # selects genes for which the cox model makes sense in both profiles
    patients.both.not.na <- which(
      (!is.na(survival.results[[cond1]]$hr)) & (!is.na(survival.results[[cond2]]$hr)) &
      survival.results[[cond1]]$num.events.high >= 79 & survival.results[[cond2]]$num.events.high >=
      survival.results[[cond1]]$coxzph.pvalue > 0.01 & survival.results[[cond2]]$coxzph.pvalue > 0.01
      );
    # find prognostic genes
    significant.genes <- intersect(union(
      rownames(survival.results[[cond1]])[which(
        (survival.results[[cond1]]$wald.qvalue < pval.thres) &
        (abs(log2(survival.results[[cond1]]$hr)) > hr.thres) &
        (!is.na(survival.results[[cond1]]$hr)) &
```

```
      (survival.results[[cond1]]$coxzph.pvalue > 0.01) &
      survival.results[[cond1]]$num.events.high >= 79
      )],
    rownames(survival.results[[cond2]])[which(
      (survival.results[[cond2]]$wald.qvalue < pval.thres) &
      (abs(log2(survival.results[[cond2]]$hr)) > hr.thres) &
      (!is.na(survival.results[[cond2]]$hr)) &
      (survival.results[[cond2]]$coxzph.pvalue > 0.01) &
      survival.results[[cond2]]$num.events.high >= 79
      )]
    ),rownames(survival.results[[cond2]])[patients.both.not.na]);
  # pull out the significant genes from each profile type for point colouring
  significant.genes.bulk <- (survival.results$bulk[significant.genes,'wald.qvalue'] < pval.thres) &
    (abs(log2(survival.results$bulk[significant.genes,'hr'])) > hr.thres) &
    (!is.na(survival.results$bulk[significant.genes,'hr'])) &
    (survival.results$bulk[significant.genes,'coxzph.pvalue'] > 0.01) &
    (survival.results$bulk[significant.genes,'num.events.high'] >= 79);
  significant.genes.tc <- (survival.results$tc[significant.genes,'wald.qvalue'] < pval.thres) &
    (abs(log2(survival.results$tc[significant.genes,'hr'])) > hr.thres) & (!is.na(survival.results$
    (survival.results$tc[significant.genes,'coxzph.pvalue'] > 0.01) &
    (survival.results$tc[significant.genes,'num.events.high'] >= 79);
  significant.genes.tac <- (survival.results$tac[significant.genes,'wald.qvalue'] < pval.thres) &
    (abs(log2(survival.results$tac[significant.genes,'hr'])) > hr.thres) & (!is.na(survival.results
    (survival.results$tac[significant.genes,'coxzph.pvalue'] > 0.01) &
    (survival.results$tac[significant.genes,'num.events.high'] >= 79);
  if(length(significant.genes) > 0) {
    # assign colours to point to show which profile the gene is prognostic in
    significance.colours <- c('darkred','dodgerblue','orchid','gold','darkorange3','chartreuse4','b
    point.col <- rep('red',length(significant.genes));
    point.col[significant.genes.bulk & !significant.genes.tc & !significant.genes.tac] <-
      significance.colours[1];
    point.col[!significant.genes.bulk & significant.genes.tc & !significant.genes.tac] <-
      significance.colours[2];
    point.col[!significant.genes.bulk & !significant.genes.tc & significant.genes.tac] <-
      significance.colours[4];
    point.col[significant.genes.bulk & significant.genes.tc & !significant.genes.tac] <-
      significance.colours[3];
    point.col[significant.genes.bulk & !significant.genes.tc & significant.genes.tac] <-
      significance.colours[5];
    point.col[!significant.genes.bulk & significant.genes.tc & significant.genes.tac] <-
      significance.colours[6];
    point.col[significant.genes.bulk & significant.genes.tc & significant.genes.tac] <-
      significance.colours[7];
    # create the comparison plot
    create.scatterplot(
      y ~ x,
      data.frame(
        x=log2(survival.results[[cond1]][significant.genes,'hr']),
        y=log2(survival.results[[cond2]][significant.genes,'hr'])
        ),
```

```
      filename = paste0('./figure3_',cond1,'_',cond2,'.pdf'),
      xlab.label=bquote(.(sub('tac','TAC',sub('bulk','Bulk',sub('tc','TC',cond1))))*' '*log[2]*'HR'
      ylab.label=bquote(.(sub('tac','TAC',sub('bulk','Bulk',sub('tc','TC',cond2))))*' '*log[2]*'HR'
      xlimits=c(-1,1),
      ylimits=c(-1,1),
      xat=seq(-1,1,0.5),
      yat=seq(-1,1,0.5),
      abline.h=0,
      abline.v=0,
      abline.lty=2,
      resolution=400,
      alpha=0.6,
      cex=0.5,
      col=point.col,
      add.curves=TRUE,
      curves.exprs=list(
        function(x){x+hr.thres},
        function(x){x-hr.thres},
        function(x){-x+hr.thres},
        function(x){-x-hr.thres},
        function(x){x}
        ),
      curves.to=2,
      curves.from=-3,
      curves.col=c(rep('gray65',4),'black'),
      curves.lwd=rep(1,5),
      curves.lty=rep(2,5)
      );
    }
  }
}
```

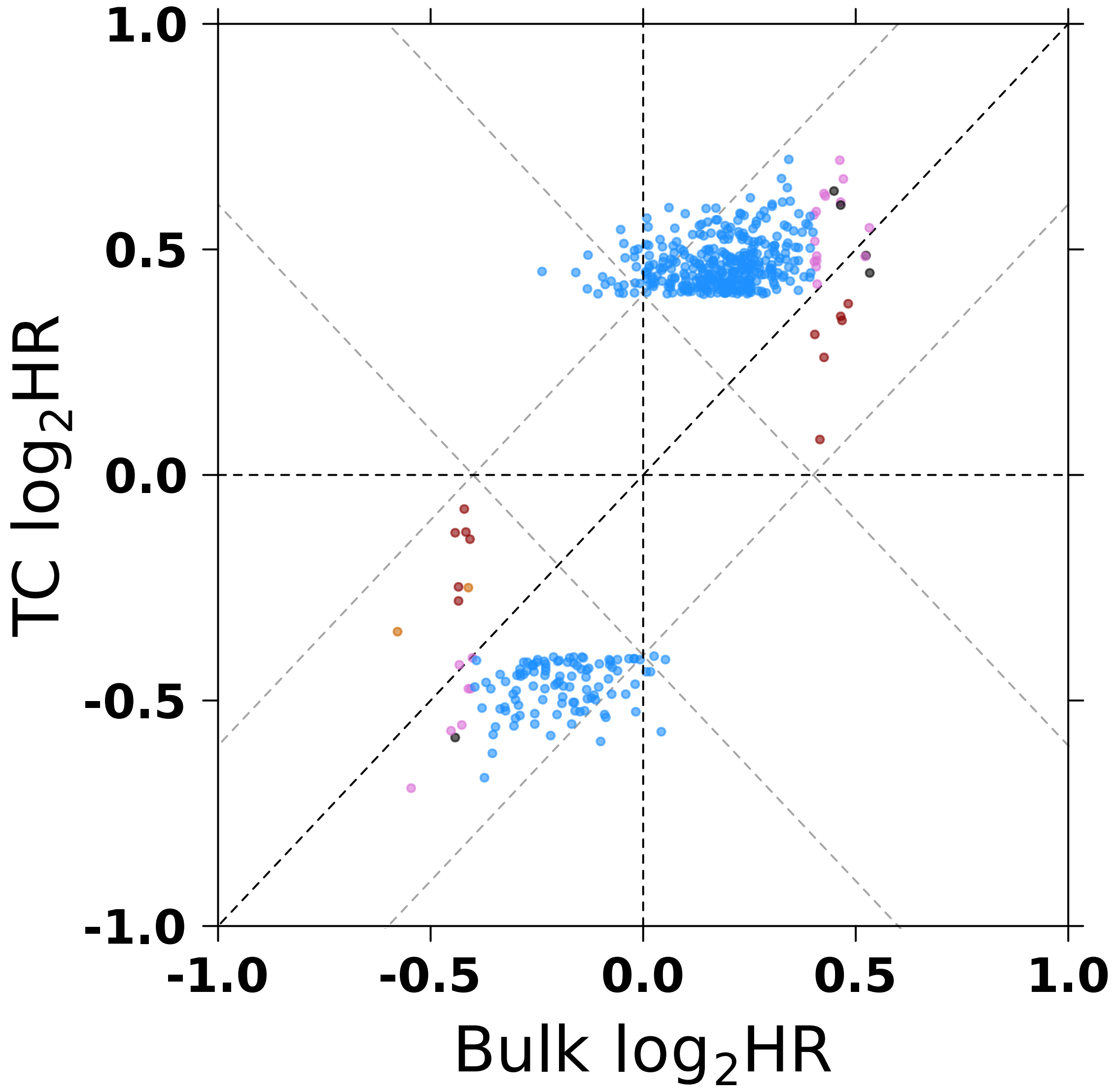
1.0
0.5
0.0
-0.5
-1.0
TC $\log_2$HR
-1.0
-0.5
0.0
0.5
1.0
Bulk $\log_2$HR

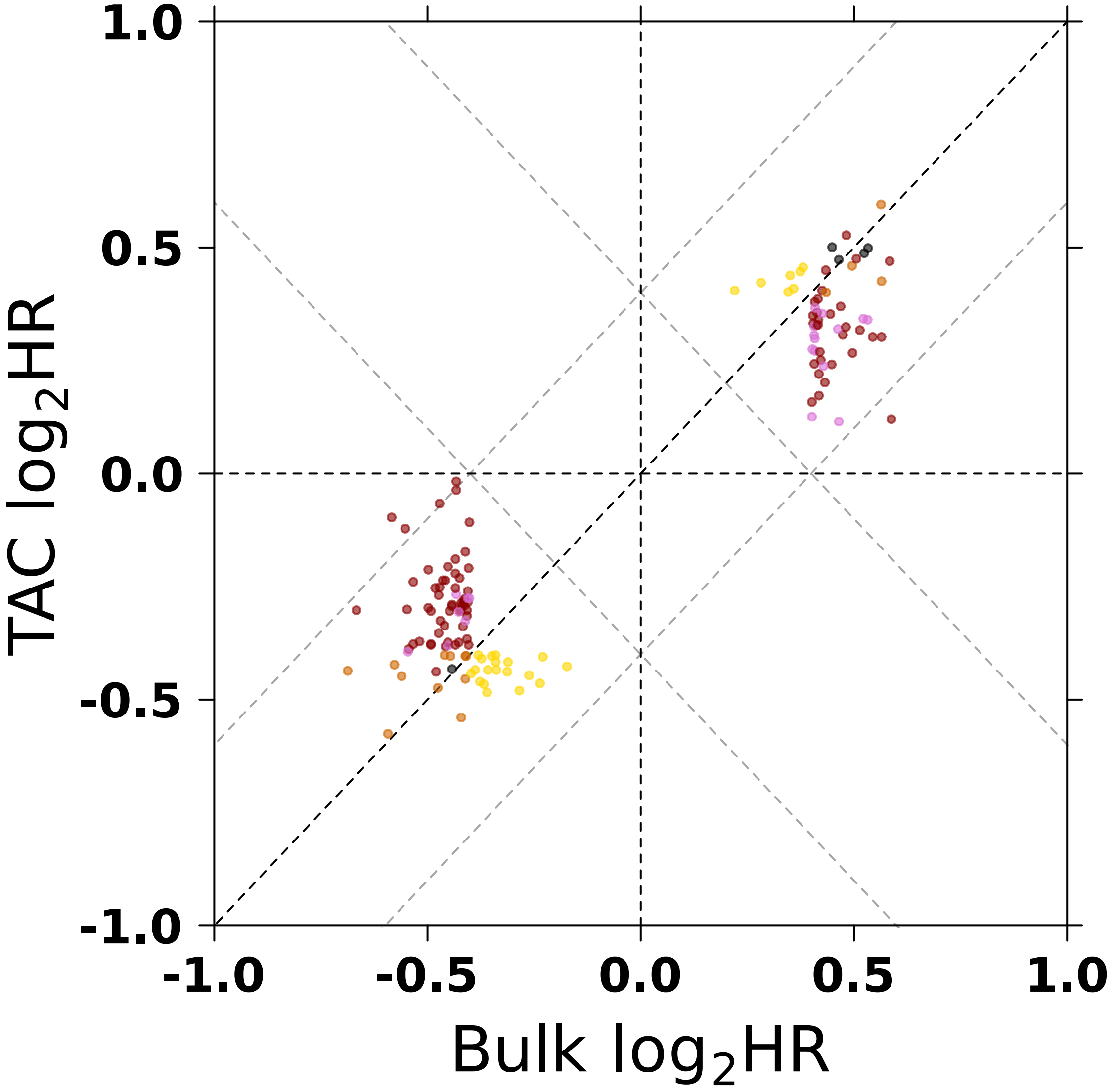

1.0
0.5
0.0
-0.5
-1.0
TAC $\log_2$HR
-1.0
-0.5
0.0
0.5
1.0
Bulk $\log_2$HR

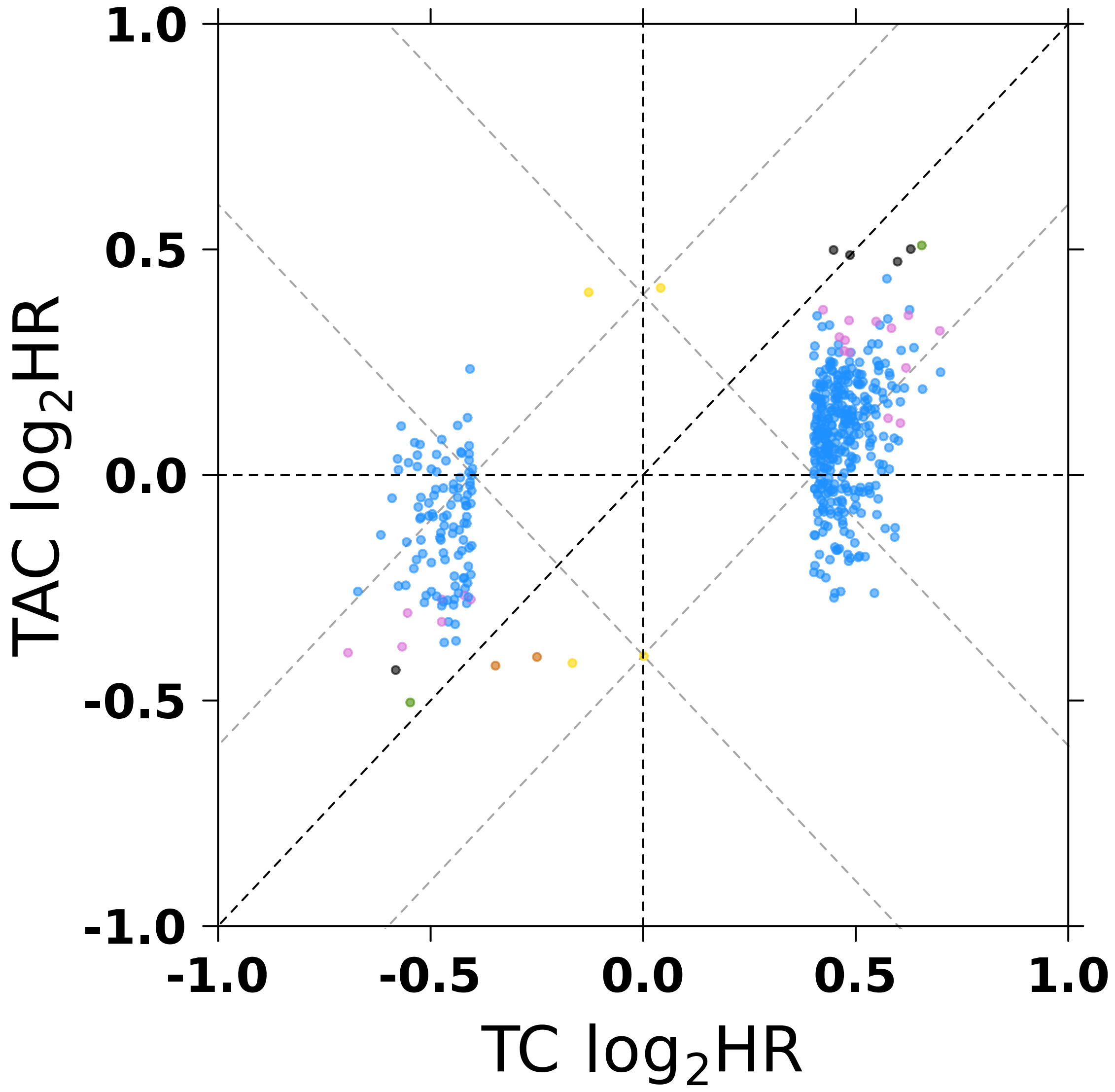

Create a legend for plot colours

```
create.scatterplot(
  y ~ x,
  data.frame(
    x=1:4,
    y=1:4
    ),
  col='white',
  filename = './figure3_legend.pdf',
  xat=NULL,
  yat=NULL,
  xlab.label=NULL,
  ylab.label=NULL,
  resolution=400,
  width=4,
  height=4,
  key= list(
```

```
    points = list(
      pch = 19,
      col = c('darkred','dodgerblue','gold','orchid','darkorange3','chartreuse4','black'),
      cex = 1
      ),
    text = list(
      lab = c(
        'Only Bulk',
        'Only TC',
        'Only TAC',
        'Bulk & TC',
        'Bulk & TAC',
        'TC & TAC',
        'All profiles'
        ),
      cex = 1,
      col = 'black'
      ),
    title='Gene significance',
    title.cex=1.2,
    x = 0.2,
    y = 0.9,
    padding.text = 2
    )
  );
```

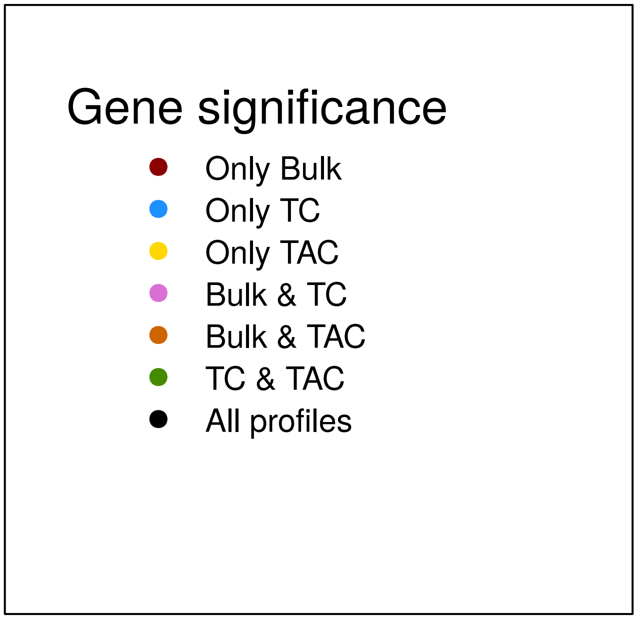

Pull out the information for supplementary table 8

```
stable <- cbind(rownames(survival.results$bulk),
  survival.results$bulk[,
    c('hr','lower.95','upper.95','wald.pvalue','wald.qvalue','coxzph.pvalue','num.patients.high','dic
    ],
  survival.results$tc[,
    c('hr','lower.95','upper.95','wald.pvalue','wald.qvalue','coxzph.pvalue','num.patients.high','dic
    ],
  survival.results$tac[,
    c('hr','lower.95','upper.95','wald.pvalue','wald.qvalue','coxzph.pvalue','num.patients.high','dic
    ]
  );
colnames(stable) <- c('entrez.gene.id',
  paste0(
    'bulk.',
    c('hr','hr.lower.95','hr.upper.95','ward.pvalue','ward.qvalue','coxzph.pvalue','num.patient.high.
    ),
  paste0(
    'tc.',
    c('hr','hr.lower.95','hr.upper.95','ward.pvalue','ward.qvalue','coxzph.pvalue','num.patient.high.
    ),
  paste0(
    'tac.',
    c('hr','hr.lower.95','hr.upper.95','ward.pvalue','ward.qvalue','coxzph.pvalue','num.patient.high.
    )
  );
write.table(
  stable[intersect(
    rownames(survival.results$tc)[
      survival.results$tc$wald.qvalue < pval.thres &
      abs(log2(survival.results$tc$hr)) > hr.thres &
      survival.results$tc$coxzph.pvalue > 0.01 &
      survival.results$tc$num.events.high >= 79
      ],
    rownames(survival.results$tac)[
      !is.na(survival.results$tac$hr) &
      survival.results$tac$wald.qvalue < pval.thres &
      abs(log2(survival.results$tac$hr)) > hr.thres &
      survival.results$tac$coxzph.pvalue > 0.01 &
      survival.results$tac$num.events.high >= 79
      ]
    ),],
  file='./3-univariate_prognosis_stable_genes_with_significant_univariate_cox_modelling.txt', ## Supp
  col.names=TRUE,
  row.names=FALSE,
  quote=FALSE,
  sep='\t'
  );
```

18 Supplementary Figure 6A-B - p-value sensitivity for univariate analysis

Set up the environment

```
library(BoutrosLab.plotting.general);
library(yaml);
dataset.name <- 'Metabric';
```

Yaml file name

```
yaml.file <- paste0(dataset.name,'.yaml');
```

Read yaml with file information

```
dataset.files <- yaml.load_file(yaml.file);
```

Load clinical annotation

```
patient.anno <- read.table(dataset.files$clinical.annotation.file,sep='\t',header=TRUE);
```

Load mRNA abundance profiles

```
profile.types <- c('bulk','tc','tac');
```

Load univariate survival results

```
survival.results <- list();
for(profile.type in profile.types) {
  survival.results[[profile.type]] <- read.table(
    file=paste0(dataset.files$univariate.survival.output.dir,dataset.name,'_',profile.type,'_gene_uni
    header=TRUE,
    sep='\t'
    );
  }
```

Specify the points to make a line - want to make sure there are enough points so that there aren't gaps and they forma line

```
pvalue.thresholds <- sort(unique(c(10^(-seq(0,0.75,0.0001)),10^(-seq(0.75,1,0.001)),10^(-seq(0,9.2,0.
```

Calculate that the number of significant genes at each hr threshold and different q-value thresholds

```
hr.thresholds <- c(0.5);
pvalue.threshold.count <- matrix(
  NA,
  ncol=length(pvalue.thresholds),nrow=length(profile.types)*(length(hr.thresholds)+1)
  );
colnames(pvalue.threshold.count) <- as.character(pvalue.thresholds);
rownames(pvalue.threshold.count) <- c(
  profile.types,
```

```
  sapply(profile.types,function(x) {paste0(x,'_hr',hr.thresholds) })
  );
pvalue.threshold.count.cox.zph.pass <- matrix(
  NA,
  ncol=length(pvalue.thresholds),nrow=length(profile.types)*(length(hr.thresholds)+1)
  );
colnames(pvalue.threshold.count.cox.zph.pass) <- as.character(pvalue.thresholds);
rownames(pvalue.threshold.count.cox.zph.pass) <- c(
  profile.types,
  sapply(profile.types,function(x) {paste0(x,'_hr',hr.thresholds) })
  );
for(profile.type in profile.types) {
  for(thres in pvalue.thresholds) {
    pvalue.threshold.count[profile.type,as.character(thres)] <- sum(
      (survival.results[[profile.type]]$wald.qvalue < thres) &
      (survival.results[[profile.type]]$coxzph.pvalue > 0.01) &
      (survival.results[[profile.type]]$num.patients.high >= 79));
    for(hr.thres in hr.thresholds) {
      pvalue.threshold.count[paste0(profile.type,'_hr',hr.thres),as.character(thres)] <- sum(
        (survival.results[[profile.type]]$wald.qvalue < thres) &
        (abs(log2(survival.results[[profile.type]]$hr)) > hr.thres) &
        (survival.results[[profile.type]]$coxzph.pvalue > 0.01) &
        (survival.results[[profile.type]]$num.patients.high >= 79));
      }
    }
  }
```

Create q-value sensitivity plot

```
cond.col <- c('firebrick3','dodgerblue3','gold','darkred','navy','darkgoldenrod4');
create.scatterplot(
  y ~ x,
  data.frame(
    x=c(-log10(rep(pvalue.thresholds,each=nrow(pvalue.threshold.count)))),
    y=c(as.numeric(pvalue.threshold.count[nrow(pvalue.threshold.count):1,]))
    ),
  groups= rep(1:nrow(pvalue.threshold.count),ncol(pvalue.threshold.count)),
  col=cond.col[rev(c(1:6))],
  filename = './sfigure6a.png',
  xlab.label=expression(bold(-log[10]*' q-value threshold')),
  ylab.label='Number of significant genes',
  type='p',
  cex=0.17,
  resolution=400,
  xlab.cex=1.5,
  xaxis.cex=1.5,
  ylab.cex=1.5,
  yaxis.cex=1.5,
  ylimits=c(0,5000),
  xlimits=c(0,9.2),
```

```
  xaxis.lab=as.character(sapply(seq(0,9,3),function(x) { c(x,'','')})),
  xat=seq(0,9,1),
  yat=seq(0,5000,1000),
  yaxis.lab=seq(0,5000,1000),
  key= list(
    points = list(
      pch = 19,
      col = cond.col,
      cex = 1
      ),
    text = list(
      lab = c('Bulk','TC','TAC',
        expression('Bulk, |'*log[2]*'HR| > 0.5'),
        expression('TC, |'*log[2]*'HR| > 0.5'),
        expression('TAC, |'*log[2]*'HR| > 0.5')
        ),
      cex = 1,
      col = 'black'
      ),
    x = 0.5,
    y = 0.95,
    padding.text = 2
    )
  );
```

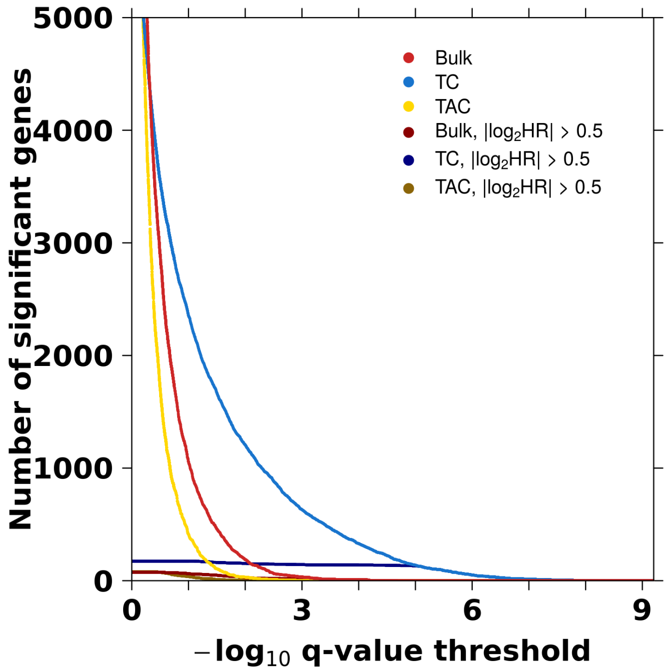

Determine the overlap between profile types at different q-value thresholds

```
pvalue.thresholds <- 10^(-seq(1,5,1));
venn.prop <- matrix(NA, nrow=length(pvalue.thresholds),ncol=7);
rownames(venn.prop) <- as.character(pvalue.thresholds);
colnames(venn.prop) <- c('nst','st','ns','nt','s','t','n');
```

```
venn.overlap <- matrix(NA, nrow=length(pvalue.thresholds),ncol=3);
rownames(venn.overlap) <- as.character(pvalue.thresholds);
colnames(venn.overlap) <- c('st','ns','nt');
for(pval.thres in pvalue.thresholds) {
  significant.genes.bulk <- survival.results$bulk$wald.qvalue < pval.thres &
    survival.results$bulk$coxzph.pvalue > 0.01 &
    survival.results$bulk$num.patients.high >= 79;
  significant.genes.tc <- survival.results$tc$wald.qvalue < pval.thres &
    survival.results$tc$coxzph.pvalue > 0.01 &
    survival.results$tc$num.patients.high >= 79;
  significant.genes.tac <- survival.results$tac$wald.qvalue < pval.thres &
    survival.results$tac$coxzph.pvalue > 0.01 &
    survival.results$tac$num.patients.high >= 79;
  total.size <- sum(significant.genes.bulk | significant.genes.tc | significant.genes.tac);
  venn.overlap[as.character(pval.thres),'nt'] <-
    sum(significant.genes.bulk & significant.genes.tc)/
    min(sum(significant.genes.bulk), sum(significant.genes.tc));
  venn.overlap[as.character(pval.thres),'st'] <-
    sum(significant.genes.tac & significant.genes.tc)/
    min(sum(significant.genes.tac),sum( significant.genes.tc));
  venn.overlap[as.character(pval.thres),'ns'] <-
    sum(significant.genes.bulk & significant.genes.tac)/
    min(sum(significant.genes.bulk),sum(significant.genes.tac));
  }
```

Create barplot showing overlap in the significant genes

```
create.barplot(
  prop ~ threshold,
  data = data.frame(
    prop = as.numeric(venn.overlap),
    threshold = rep(1:nrow(venn.overlap),ncol(venn.overlap))
    ),
  groups=as.factor(rep(1:ncol(venn.overlap),each=nrow(venn.overlap))),
  filename = './sfigure6b.pdf',
  resolution=300,
  col=c('chartreuse4','darkorange1','orchid4'),
  ylab.label='Proportion of significant\ngenes intersecting',
  xlab.label=expression(bold(-log[10]*' q-value threshold')),
  stack=FALSE,
  yat=seq(0,0.8,0.1),
  yaxis.lab=seq(0,0.8,0.1),
  ylab.cex=1.5,
  xlab.cex=1.5,
  xaxis.cex=1.5,
  yaxis.cex=1.5,
  width=5,
  legend= list(
    bottom=list(
      fun=legend.grob(
```

```r
        legends=list(
          legend=list(
            colours = rep('transparent',6),
            labels = rep('',6),
            border='transparent'
            ),
          legend=list(
            colours = c('chartreuse4','darkorange1','orchid4'),
            labels = rev(c(
              'Bulk & TC',
              'Bulk & TAC',
              'TC & TAC'
              )),
            title='Profiles Compared',
            border='black'
            )
          ),
        title.just='left'
        )
      )
    ),
  bottom.padding = 12
  );
```

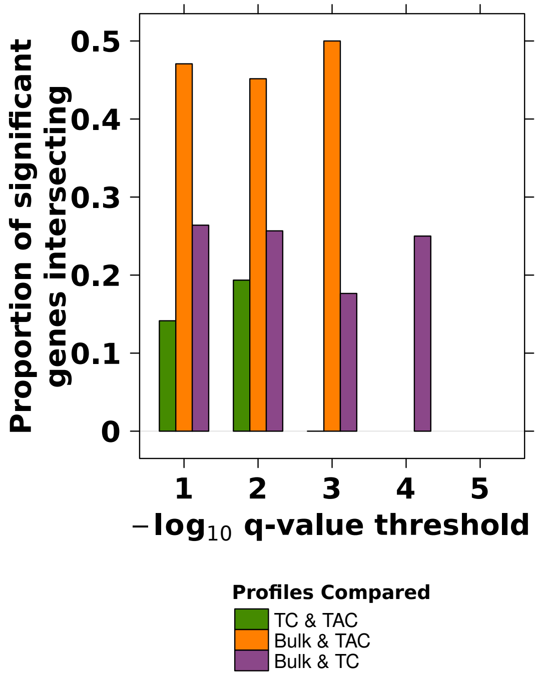

19 Supplementary Figure 6C-E - Univariate Cox modelling Hazard ratio correlation

Set up the environment

```
library(BoutrosLab.plotting.general);
library(yaml);
dataset.name <- 'Metabric';
```

Yaml file name

```
yaml.file <- paste0(dataset.name,'.yaml');
```

Read yaml with file information

```
dataset.files <- yaml.load_file(yaml.file);
```

Load clinical annotation

```
patient.anno <- read.table(dataset.files$clinical.annotation.file,sep='\t',header=TRUE);
```

Load mRNA abundance profiles

```
profile.types <- c('bulk','tc','tac');
```

Load univariate survival results

```
survival.results <- list();
for(profile.type in profile.types) {
  survival.results[[profile.type]] <- read.table(
    file=paste0(
      dataset.files$univariate.survival.output.dir,
      dataset.name,
      '_',
      profile.type,
      '_gene_univariate_survival_results.txt'
      ),
    header=TRUE,
    sep='\t'
    );
  }
```

Plot univariate results: create a hexbin plot comparing the HR between pairs of profile types without filtering based on p-value

```
for(i in 1:(length(profile.types)-1)) {
  cond1 <- profile.types[i];
  for(j in (i+1):length(profile.types)) {
    cond2 <- profile.types[j];
    patients.both.not.na <- which(
      (!is.na(survival.results[[cond1]]$hr)) & (!is.na(survival.results[[cond2]]$hr)) &
      survival.results[[cond1]]$num.events.high >= 79 & survival.results[[cond2]]$num.events.high >=
      survival.results[[cond1]]$coxzph.pvalue > 0.01 & survival.results[[cond2]]$coxzph.pvalue > 0.01
      );
    create.hexbinplot(
```

```
      formula=y ~ x,
      data=data.frame(
        x=log2(survival.results[[cond1]]$hr[patients.both.not.na]),
        y=log2(survival.results[[cond2]]$hr[patients.both.not.na])
        ),
      filename = paste0('./sfigure6de_',cond1,'_',cond2,'.pdf'),
      resolution=400,
      xlab.label=bquote(.(sub('tac','TAC',sub('bulk','Bulk',sub('tc','TC',cond1))))*' '*log[2]*'HR'),
      ylab.label=bquote(.(sub('tac','TAC',sub('bulk','Bulk',sub('tc','TC',cond2))))*' '*log[2]*'HR'),
      xlimits=c(-1,1),
      ylimits=c(-1,1),
      xat=seq(-1,1,0.5),
      yat=seq(-1,1,0.5),
      add.xyline=TRUE,
      abline.front=TRUE,
      add.axes=TRUE,
      aspect=1,
      width=9,
      legend = list(
        inside = list(
          fun = draw.key,
          args = list(
            key = get.corr.key(
              x = log2(survival.results[[cond1]]$hr[patients.both.not.na]),
              y = log2(survival.results[[cond2]]$hr[patients.both.not.na]),
              label.items = c('spearman','spearman.p'),
              alpha.background = 0,
              key.cex = 1
              )
            ),
          x = 0.65,
          y = 0.15,
          corner = c(0,1)
          )
        )
      );
    }
  }
```

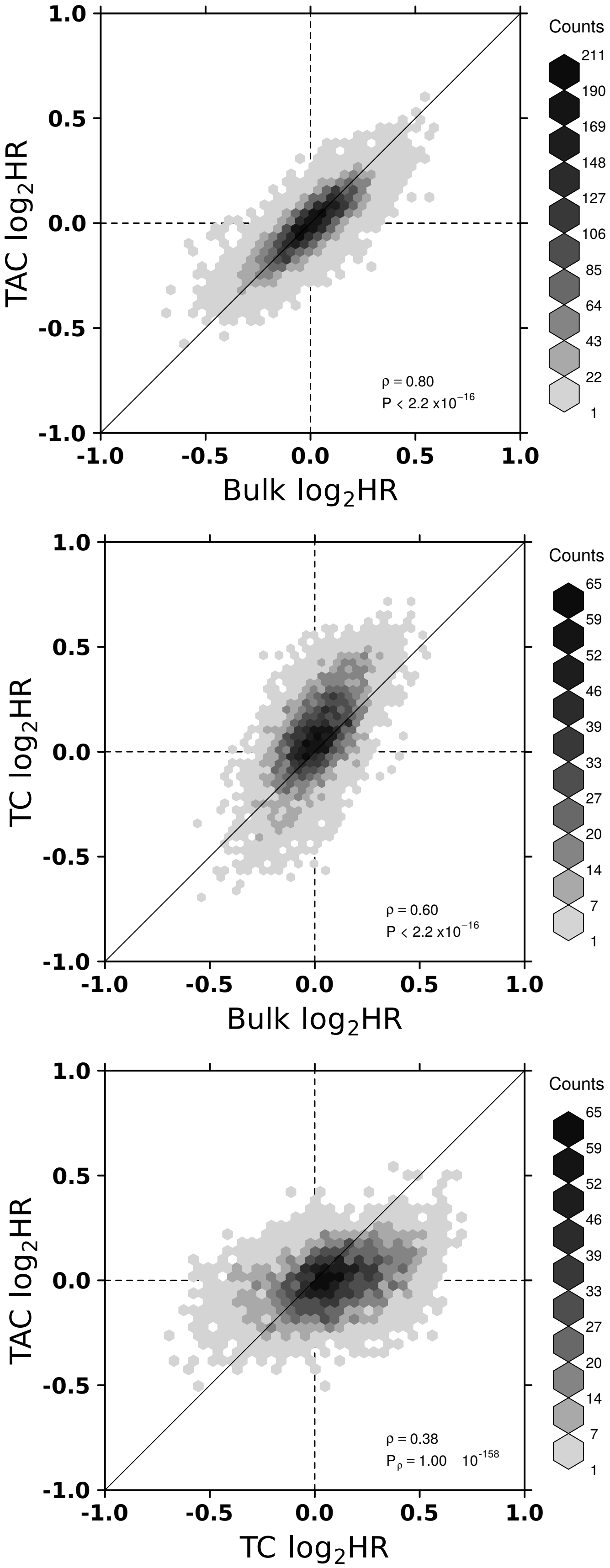
TAC log2HR
Bulk log2HR
Counts
211
190
169
148
127
106
85
64
43
22
1
ρ = 0.80
P < 2.2 x10^-16
TC log2HR
Bulk log2HR
Counts
65
59
52
46
39
33
27
20
14
7
1
ρ = 0.60
P < 2.2 x10^-16
TAC log2HR
TC log2HR
Counts
65
59
52
46
39
33
27
20
14
7
1
ρ = 0.38
Pρ = 1.00 10^-158

20 Supplementary Figure 6F - mRNA distributions

Set up the environment

```
library(BoutrosLab.plotting.general);
library(yaml);
dataset.name <- 'Metabric';
```

Yaml file name

```
yaml.file <- paste0(dataset.name,'.yaml');
```

Read yaml with file information

```
dataset.files <- yaml.load_file(yaml.file);
```

Load gene annotation

```
gene.info <- read.table(dataset.files$gene.symbol.file,header=TRUE,sep='\t');
```

Load clinical annotation

```
patient.anno <- read.table(dataset.files$clinical.annotation.file,sep='\t',header=TRUE);
```

Load mRNA abundance profiles

```
profile.types <- c('bulk','tc','tac');
rna.data.matrix <- list();
for(profile.type in profile.types) {
  rna.data.matrix[[profile.type]] <- read.table(
    dataset.files[[paste0(profile.type,'.mrna.abundance.file')]],
    header=TRUE,
    sep='\t'
    );
  }
rna.normals <- read.table(dataset.files$normal.panel.bulk.mrna.abundance.file,header=TRUE,sep='\t');
rna.normals <- rna.normals[which(rownames(rna.normals) != 'NA_at'),];
```

Split the genes into chr Y and other genes

```
chry.genes <- rownames(gene.info)[gene.info$Chr == 'chrY'];
other.genes <- rownames(gene.info)[gene.info$Chr %in% paste0('chr',c(1:22,'X'))];
```

Create the density plot

```
create.densityplot(
  list(
    bulk_not=as.numeric(as.matrix(rna.data.matrix$bulk[rownames(rna.data.matrix$bulk) %in% other.gene
    bulk_y=as.numeric(as.matrix(rna.data.matrix$bulk[rownames(rna.data.matrix$bulk) %in% chry.genes,]
    tac_not=as.numeric(as.matrix(rna.data.matrix$tac[rownames(rna.data.matrix$tac) %in% other.genes,]
```

```
  tac_y=as.numeric(as.matrix(rna.data.matrix$tac[rownames(rna.data.matrix$tac) %in% chry.genes,])),
  tc_not=as.numeric(as.matrix(rna.data.matrix$tc[rownames(rna.data.matrix$tc) %in% other.genes,])),
  tc_y=as.numeric(as.matrix(rna.data.matrix$tc[rownames(rna.data.matrix$tc) %in% chry.genes,])),
  normals_not=as.numeric(as.matrix(rna.normals[rownames(rna.normals) %in% other.genes,])),
  normals_y=as.numeric(as.matrix(rna.normals[rownames(rna.normals) %in% chry.genes,]))
),
filename = './sfigure6f.pdf',
resolution=300,
col=c('firebrick3','darkred','gold','darkgoldenrod4','dodgerblue3','navy','gray61','gray19'),
xlimits=c(3,13),
width=10,
xat=seq(3,13,0.5),
xaxis.lab=as.character(sapply(seq(3,13),function(x) { c(x,'')}))[1:21],
lty=rep(c('solid','dashed'),4),
abline.v=6.5,
legend = list(
  inside = list(
    fun = draw.key,
    args = list(
      key = list(
        lines = list(
          col = c(
            'firebrick3',
            'darkred',
            'gold',
            'darkgoldenrod4',
            'dodgerblue3',
            'navy',
            'gray61',
            'gray19'
            ),
          cex = 1.5,
          lty = rep(c('solid','dashed'),4),
          lwd=2
          ),
        text = list(
          lab = c(
            'bulk mRNA from non-Y chromosomes',
            'bulk mRNA from Y chromosome',
            'TAC mRNA from non-Y chromosomes',
            'TAC mRNA from Y chromosome',
            'TC mRNA from non-Y chromosomes',
            'TC mRNA from Y chromosome',
            'non-maligant mRNA from non-Y chromosomes',
            'non-malignant mRNA from Y chromosome'
            )
          ),
        cex = 1.2
        )
      ),
```

```
    x = 0.4,
    y = 0.97,
    draw = FALSE
    )
  )
);
```

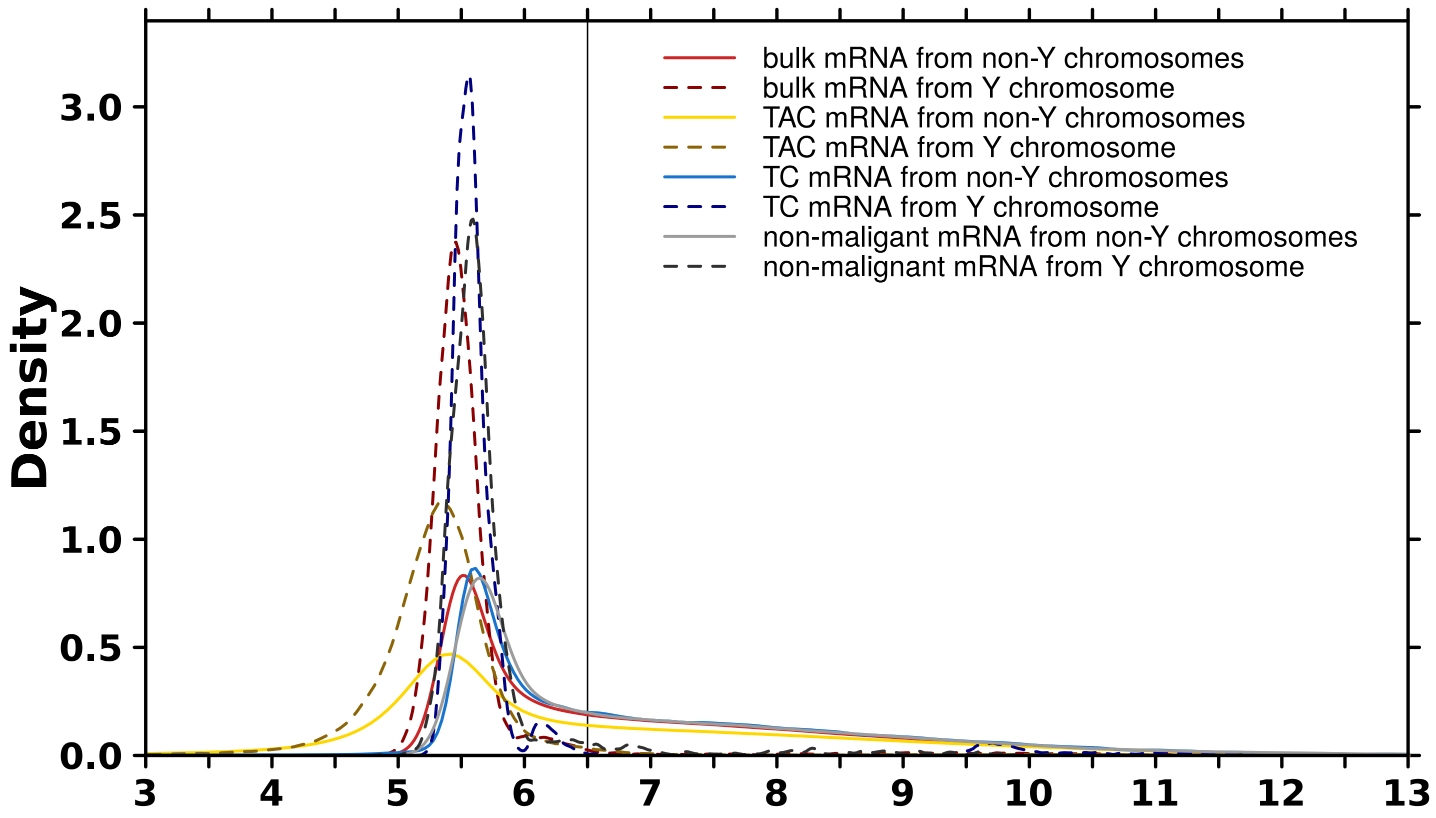

21 Supplementary Figure 7 - Correlation of univariate Cox modelling hazard ratio between subtype specific analysis and all patients

Set up the environment

```
library(BoutrosLab.plotting.general);
library(yaml);
dataset.name <- 'Metabric';
```

Yaml file name

```
yaml.file <- paste0(dataset.name,'.yaml');
```

Read yaml with file information

```
dataset.files <- yaml.load_file(yaml.file);
```

Load clinical annotation

```
patient.anno <- read.table(dataset.files$clinical.annotation.file,sep='\t',header=TRUE);
```

Load mRNA abundance profiles

```
profile.types <- c('bulk','tc','tac');
```

Load univariate survival results

```
survival.results <- list();
for(profile.type in profile.types) {
  survival.results[[profile.type]] <- list();
  survival.results[[profile.type]][['all']] <- read.table(
    file=paste0(
      dataset.files$univariate.survival.output.dir,
      dataset.name,
      '_',
      profile.type,
      '_gene_univariate_survival_results.txt'
      ),
    header=TRUE,
    sep='\t'
    );
  for(subtype in c('Basal','Her2','LumA','LumB')) {
    survival.results[[profile.type]][[subtype]] <- read.table(
      file=paste0(
        '/.mounts/labs/boutroslab/private/Biomarkers/PanCancer/TumourPurityEffects/results/juri_univa
        dataset.name,
        '_',
        subtype,
        '_gene_survival_result_tc_mrna.txt'
        ),
      header=TRUE,
      sep='\t'
      );
    }
  }
```

Plot univariate results

```
pval.thres <- 0.05;
hr.thres <- 0.4;
for(subtype in c('Basal','Her2','LumA','LumB')) {
  for(profile.type in profile.types) {
    # find prognostic genes
    significant.genes.all.subtypes <- rownames(survival.results[[profile.type]]$all)[which(
      (survival.results[[profile.type]]$all$wald.qvalue < pval.thres) &
      (abs(log2(survival.results[[profile.type]]$all$hr)) > hr.thres) &
      (!is.na(survival.results[[profile.type]]$all$hr)) &
      (survival.results[[profile.type]]$all$coxzph.pvalue > 0.01) &
      survival.results[[profile.type]]$all$num.events.high >= 79
```

```
    )];
  significant.genes.both <- intersect(
    significant.genes.all.subtypes,
    rownames(survival.results[[profile.type]][[subtype]])[which(
      (survival.results[[profile.type]][[subtype]]$wald.pvalue < pval.thres) &
      (abs(log2(survival.results[[profile.type]][[subtype]]$hr)) > hr.thres) &
      (!is.na(survival.results[[profile.type]][[subtype]]$hr)) &
      (survival.results[[profile.type]][[subtype]]$coxzph.pvalue > 0.01) &
      survival.results[[profile.type]][[subtype]]$num.events.high >= 79
      )]
    );
  plot.data <- data.frame(
    x = log2(survival.results[[profile.type]]$all[significant.genes.all.subtypes,'hr']),
    y = log2(survival.results[[profile.type]][[subtype]][significant.genes.all.subtypes,'hr'])
    );
  plot.min <- min(plot.data$y);
  plot.max <- max(plot.data$y);
  create.scatterplot(
    y ~ x,
    plot.data,
    filename = paste0('./sfigure7_',subtype,'_',profile.type,'.pdf'),
    ylab.label=bquote('Subtype specific '*log[2]*'HR'),
    xlab.label=bquote('Subtypes together '*log[2]*'HR'),
    abline.h=0,
    abline.v=0,
    abline.lty=2,
    resolution=150,
    alpha=0.4,
    cex=0.6,
    add.curves=TRUE,
    curves.exprs=list(function(x){x}),
    curves.to=3,
    curves.from=-3,
    curves.col='black',
    curves.lwd=1,
    curves.lty=2,
    xlimits=c(-1.1,1.1),
    ylimits=c(-2.1,2.1),
    yat=seq(-2,2,1),
    yaxis.lab=c('< -2',seq(-1,1),'> 2'),
    xaxis.cex=1,
    yaxis.cex=1,
    xlab.cex=1.5,
    ylab.cex=1.5,
    add.points=length(significant.genes.both) > 0,
    points.x=log2(survival.results[[profile.type]]$all[significant.genes.both,'hr']),
    points.y=log2(survival.results[[profile.type]][[subtype]][significant.genes.both,'hr']),
    points.col='red',
    points.cex=0.6,
    use.legacy.settings=TRUE,
```

```
      legend = list(
        inside = list(
          fun = draw.key,
          args = list(
            key = get.corr.key(
              y = log2(plot.data$y),
              x = log2(plot.data$x),
              label.items = c('spearman','spearman.p'),
              alpha.background = 0,
              key.cex = 1.75
              )
            ),
          x = 0.45,
          y = 0.2,
          corner = c(0,1)
          )
        )
      );
    }
  }
stable <- NULL;
for(profile.type in profile.types) {
  stable <- rbind(
    stable,
    cbind(
      rownames(survival.results[[profile.type]][[1]]),
      rep(profile.type,nrow(survival.results[[profile.type]][[1]])),
      survival.results[[profile.type]]$all,
      survival.results[[profile.type]]$Basal,
      survival.results[[profile.type]]$Her2,
      survival.results[[profile.type]]$LumA,
      survival.results[[profile.type]]$LumB
      )
    );
  }
colnames(stable)[c(1,2)] <- c('entrez.gene.id','profile.type');
colnames(stable)[(1:ncol(survival.results[[profile.type]]$all))+2] <- paste0(
  'all.subtypes.',
  colnames(stable)[(1:ncol(survival.results[[profile.type]]$all))+2]
  );
colnames(stable)[
  (1:ncol(survival.results[[profile.type]]$Basal))
  +max(grep('all.subtypes.',colnames(stable)))
  ] <- paste0(
    'Basal.',
    colnames(stable)[
      (1:ncol(survival.results[[profile.type]]$Basal))
      +max(grep('all.subtypes.',colnames(stable)))
      ]
    );
```

```r
colnames(stable)[
  (1:ncol(survival.results[[profile.type]]$Her2))
  +max(grep('Basal.',colnames(stable)))
  ] <- paste0(
    'Her2.',
    colnames(stable)[
      (1:ncol(survival.results[[profile.type]]$Her2))
      +max(grep('Basal.',colnames(stable)))
      ]
    );
colnames(stable)[
  (1:ncol(survival.results[[profile.type]]$LumA))
  +max(grep('Her2.',colnames(stable)))
  ] <- paste0(
    'LumA.',
    colnames(stable)[
      (1:ncol(survival.results[[profile.type]]$LumA))
      +max(grep('Her2.',colnames(stable)))
      ]
    );
colnames(stable)[
  (1:ncol(survival.results[[profile.type]]$LumB))
  +max(grep('LumA.',colnames(stable)))
  ] <- paste0(
    'LumB.',
    colnames(stable)[
      (1:ncol(survival.results[[profile.type]]$LumB))
      +max(grep('LumA.',colnames(stable)))
      ]
    );
stable <- stable[,c(grep('sym',colnames(stable))[1],grep('sym',colnames(stable),invert=TRUE))];
colnames(stable)[1] <- 'gene.sym';
for(subtype in c('Basal','Her2','LumA','LumB')) {
  gene.filter <- (!is.na(stable[,paste0(subtype,'.hr')])) &
    (stable[,paste0(subtype,'.coxzph.pvalue')] > 0.01) &
    stable[,paste0(subtype,'.num.events.high')] >= 79;
  stable[!gene.filter,paste0(subtype,c('.wald.pvalue','.hr','.lower.95','.upper.95'))] <- NA;
  }
stable <- stable[apply(
  stable[,intersect(grep('wald.pvalue',colnames(stable)),grep('all',colnames(stable),invert=TRUE))] <
  abs(log2(stable[,intersect(grep('hr',colnames(stable)),grep('all',colnames(stable),invert=TRUE))]))
  1,
  any,
  na.rm =TRUE
  ),];
stable$all.subtypes.logrank.pvalue <- signif(stable$all.subtypes.logrank.pvalue,digits=5);
stable$all.subtypes.wald.pvalue <- signif(stable$all.subtypes.wald.pvalue,digits=5);
stable$all.subtypes.logrank.qvalue <- signif(stable$all.subtypes.logrank.qvalue,digits=5);
stable$all.subtypes.wald.qvalue <- signif(stable$all.subtypes.wald.qvalue,digits=5);
write.table(
```

```
stable,
file=paste0('./',dataset.name,'_univariate_survival_per_subtype_results.txt'
  ),
quote=FALSE,
row.names=FALSE,
col.names=TRUE,
sep='\t'
);
```

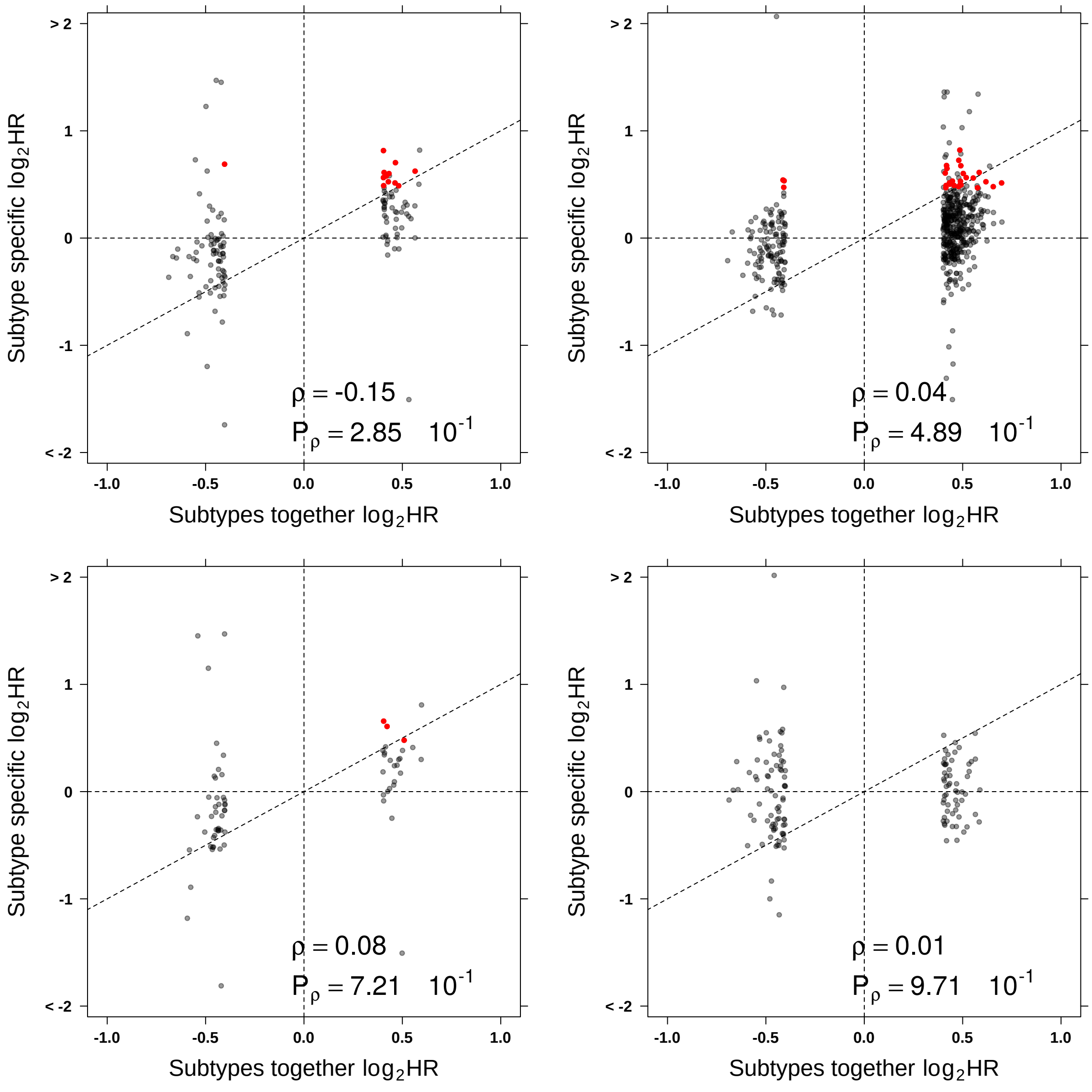

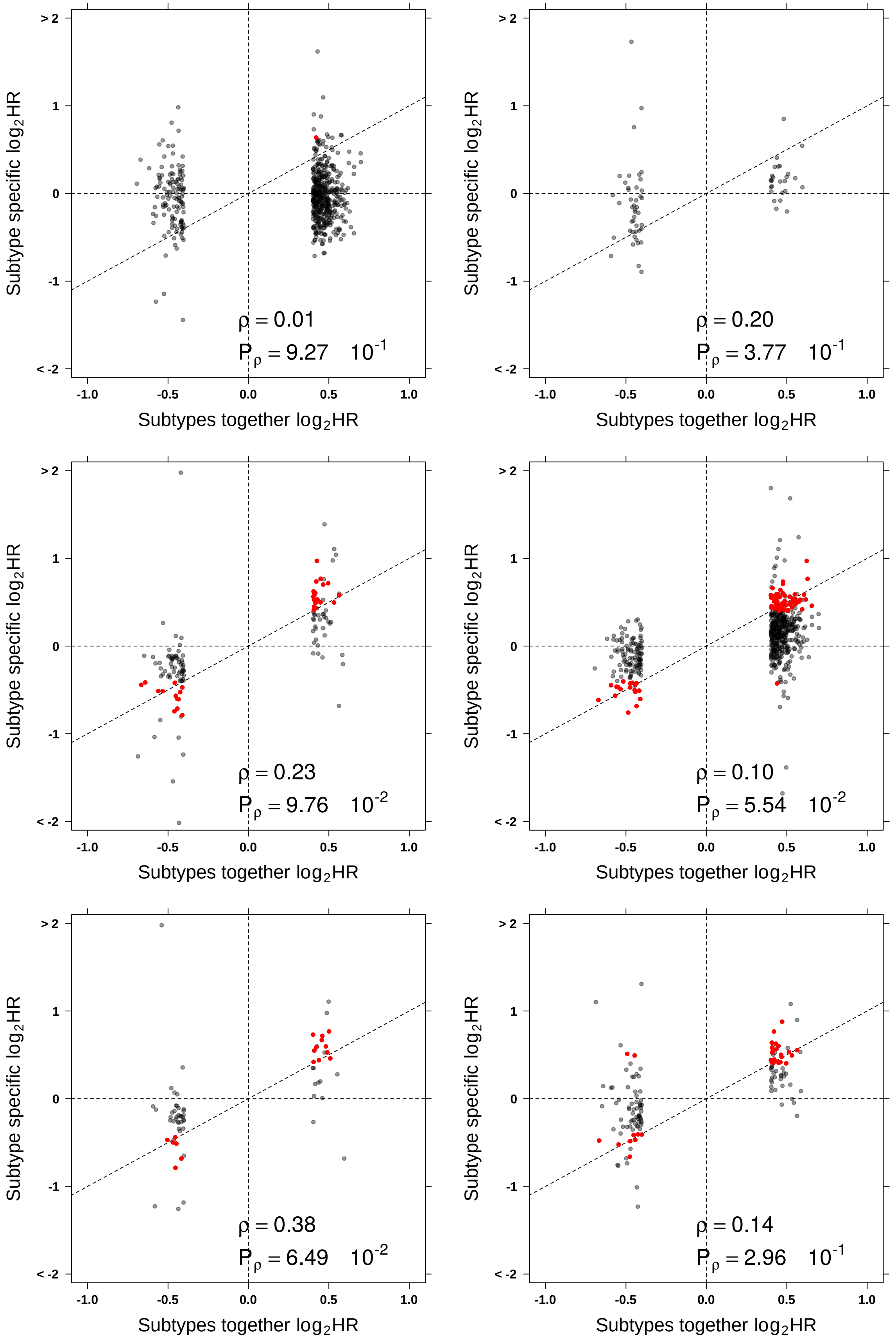
Subtype specific log2HR
Subtypes together log2HR
ρ = 0.01
Pρ = 9.27 10^-1
ρ = 0.20
Pρ = 3.77 10^-1
ρ = 0.23
Pρ = 9.76 10^-2
ρ = 0.10
Pρ = 5.54 10^-2
ρ = 0.38
Pρ = 6.49 10^-2
ρ = 0.14
Pρ = 2.96 10^-1

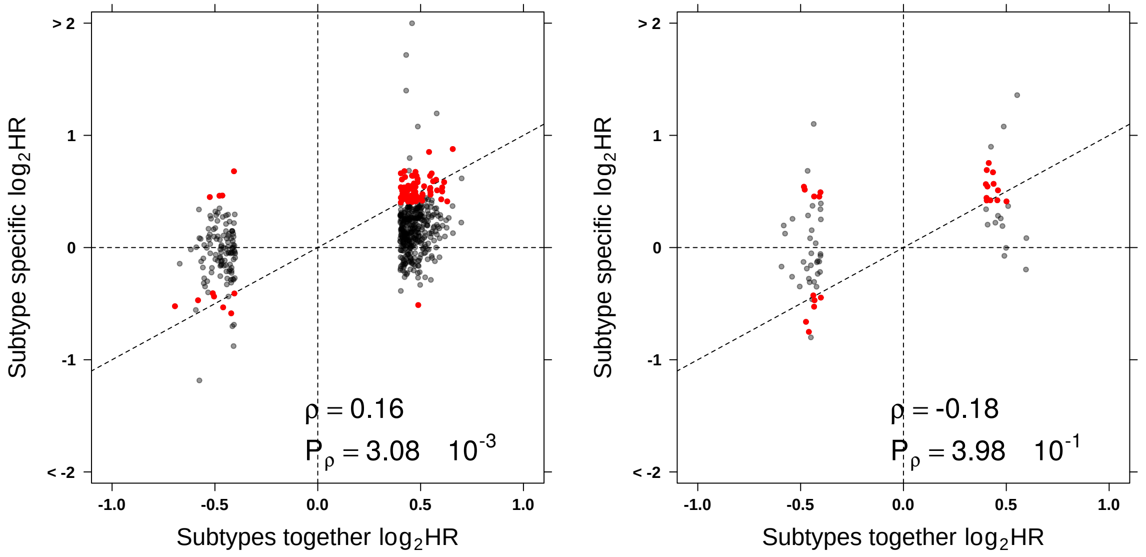

22 Running TC-TAC Interaction Survival Analysis

Set up the environment

```
library(BoutrosLab.plotting.survival);
library(yaml);
args <- commandArgs(trailingOnly = TRUE);
dataset.name <- 'Metabric';
row.start <- as.numeric(args[1]);
row.end <- as.numeric(args[2]);
unexpressed.threshold <- 6.5;
num.rows <- length(row.start:row.end);
```

Yaml file name

```
yaml.file <- paste0(dataset.name,'.yaml');
```

Read yaml with file information

```
dataset.files <- yaml.load_file(yaml.file);
```

Load clinical annotation

```
survival.anno <- read.table(dataset.files$clinical.annotation.file,sep='\t',header=TRUE);
```

Load mRNA abundance profiles

```
profiles <- list();
profiles[['tc']] <- read.table(dataset.files[['tc.mrna.abundance.file']],header=TRUE,sep='\t');
profiles[['tac']] <- read.table(dataset.files[['tac.mrna.abundance.file']],header=TRUE,sep='\t');
```

Filter the survival data to match the mRNA data

```
patients <- intersect(colnames(profiles$tc), rownames(survival.anno));
survival.anno <- survival.anno[patients,];
survival.anno <- survival.anno[!is.na(survival.anno$TimeToEvent),];
```

Filter mRNA data to match the survival data

```
profiles$tc <- profiles$tc[,rownames(survival.anno)];
profiles$tac <- profiles$tac[,rownames(survival.anno)];
```

Truncate follow-up and set up vectors for the survival object

```
time.to.event <- survival.anno$TimeToEvent[!is.na(survival.anno$TimeToEvent)];
event <- survival.anno$Event[!is.na(survival.anno$Event)];
```

Run univariate cox modelling for profiles independently

```
result.colnames <- c('coxzph.pvalue.global',
  'dichotomized.tc.mrna.value','dichotomized.tac.mrna.value',
  'num.patients.tc.low.tac.low','num.patients.tc.high.tac.low',
  'num.patients.tc.low.tac.high','num.patients.tc.high.tac.high',
  'num.events.tc.low.tac.low','num.events.tc.high.tac.low',
  'num.events.tc.low.tac.high','num.events.tc.high.tac.high',
  'surv.5.years.tc.low.tac.low','surv.5.years.tc.high.tac.low',
  'surv.5.years.tc.low.tac.high','surv.5.years.tc.high.tac.high',
  'hr.tc','hr.tac','hr.interaction',
  'wald.pvalue.tc','wald.pvalue.tac','wald.pvalue.interaction',
  'coxzph.pvalue.tc','coxzph.pvalue.tac','coxzph.pvalue.interaction',
  'logrank.pvalue'
  );
gene.survival <- matrix(NA, ncol=length(result.colnames),nrow=num.rows);
colnames(gene.survival) <- result.colnames;
for(i in row.start:row.end) {
  split.tc <- median(as.numeric(profiles$tc[i,]));
  patient.groups <- factor((as.numeric(profiles$tc[i,]) > unexpressed.threshold),levels=c(FALSE,TRUE)
  if(
    split.tc < unexpressed.threshold &
    sum(event[patient.groups == FALSE] == 1) > 0 &
    sum(event[patient.groups == TRUE] == 1) > 0
    ) {
    split.tc <- unexpressed.threshold;
    }
  split.tac <- median(as.numeric(profiles$tac[i,]));
  patient.groups <- factor((as.numeric(profiles$tac[i,]) > unexpressed.threshold),levels=c(FALSE,TRUE
  if(
    split.tac < unexpressed.threshold &
    sum(event[patient.groups == FALSE] == 1) > 0 &
    sum(event[patient.groups == TRUE] == 1) > 0
    ) {
    split.tac <- unexpressed.threshold;
    }
```

```
patient.groups <- factor(
  paste((as.numeric(profiles$tc[i,]) > split.tc),(as.numeric(profiles$tac[i,]) > split.tac)),
  levels=c('FALSE FALSE','FALSE TRUE','TRUE FALSE','TRUE TRUE')
  );
survival.object <- Surv(time.to.event, event);
gene.survival[i-row.start+1,'dichotomized.tc.mrna.value'] <- split.tc;
gene.survival[i-row.start+1,'dichotomized.tac.mrna.value'] <- split.tac;
gene.survival[i-row.start+1,'num.patients.tc.low.tac.low'] <- sum(patient.groups == 'FALSE FALSE');
gene.survival[i-row.start+1,'num.patients.tc.high.tac.low'] <- sum(patient.groups == 'TRUE FALSE');
gene.survival[i-row.start+1,'num.patients.tc.low.tac.high'] <- sum(patient.groups == 'FALSE TRUE');
gene.survival[i-row.start+1,'num.patients.tc.high.tac.high'] <- sum(patient.groups == 'TRUE TRUE');
gene.survival[i-row.start+1,'num.events.tc.low.tac.low'] <- sum(event[patient.groups == 'FALSE FALS
gene.survival[i-row.start+1,'num.events.tc.high.tac.low'] <- sum(event[patient.groups == 'TRUE FALS
gene.survival[i-row.start+1,'num.events.tc.low.tac.high'] <- sum(event[patient.groups == 'FALSE TRU
gene.survival[i-row.start+1,'num.events.tc.high.tac.high'] <- sum(event[patient.groups == 'TRUE TRU
low.high.groups <- c('tc.low.tac.low','tc.high.tac.low','tc.low.tac.high','tc.high.tac.high');
if(all(gene.survival[i-row.start+1,paste0('num.events.',low.high.groups)] > 0)) {
  # check cox proportional hazards assumptions
  cox.model <- coxph(
    surv ~ tac + tc + tc*tac,
    data=data.frame(
      surv=survival.object,
      tc=factor((as.numeric(profiles$tc[i,]) > split.tc), levels=c(FALSE,TRUE)),
      tac=factor((as.numeric(profiles$tac[i,]) > split.tac),levels=c(FALSE,TRUE))
      )
    );
  gene.survival[i-row.start+1,'coxzph.pvalue.global'] <- signif(
    BoutrosLab.statistics.survival::ph.fails(cox.model,pvalues=TRUE),
    digits=5
    )[4];
  gene.survival[i-row.start+1,c('coxzph.pvalue.tac','coxzph.pvalue.tc','coxzph.pvalue.interaction')
    BoutrosLab.statistics.survival::ph.fails(cox.model,pvalues=TRUE),
    digits=5
    )[1:3];
  # run cox model
  gene.survival[i-row.start+1,c('hr.tac','hr.tc','hr.interaction')] <- signif(
    summary(cox.model)$coefficients[,2],
    digits=2
    );
  gene.survival[i-row.start+1,c('wald.pvalue.tac','wald.pvalue.tc','wald.pvalue.interaction')] <- s
    summary(cox.model)$coefficients[,5],
    digits=5);
  # get 5 year survival for each group
  survfit.result <- summary(survfit(
    surv ~ patient_class,
    data=data.frame(
      surv=survival.object,
      patient_class=as.factor(patient.groups)
      )
    ));
```

```
    gene.survival[i-row.start+1,c('surv.5.years.tc.low.tac.low')] <- round(
      survfit.result$surv[survfit.result$strata == 'patient_class=FALSE FALSE'][
        which(
          abs(survfit.result$time[survfit.result$strata == 'patient_class=FALSE FALSE'] - 5) ==
          min(abs(survfit.result$time[survfit.result$strata == 'patient_class=FALSE FALSE'] - 5))
          )
        ],
      digits=2
      );
    gene.survival[i-row.start+1,c('surv.5.years.tc.high.tac.low')] <- round(
      survfit.result$surv[survfit.result$strata == 'patient_class=TRUE FALSE'][
        which(
          abs(survfit.result$time[survfit.result$strata == 'patient_class=TRUE FALSE'] - 5) ==
          min(abs(survfit.result$time[survfit.result$strata == 'patient_class=TRUE FALSE'] - 5))
          )
        ],
      digits=2
      );
    gene.survival[i-row.start+1,c('surv.5.years.tc.low.tac.high')] <- round(
      survfit.result$surv[survfit.result$strata == 'patient_class=FALSE TRUE'][
        which(
          abs(survfit.result$time[survfit.result$strata == 'patient_class=FALSE TRUE'] - 5) ==
          min(abs(survfit.result$time[survfit.result$strata == 'patient_class=FALSE TRUE'] - 5))
          )
        ],
      digits=2
      );
    gene.survival[i-row.start+1,c('surv.5.years.tc.high.tac.high')] <- round(
      survfit.result$surv[survfit.result$strata == 'patient_class=TRUE TRUE'][
        which(
          abs(survfit.result$time[survfit.result$strata == 'patient_class=TRUE TRUE'] - 5) ==
          min(abs(survfit.result$time[survfit.result$strata == 'patient_class=TRUE TRUE'] - 5))
          )
        ],
      digits=2
      );
    }
  if(sum(gene.survival[i-row.start+1,paste0('num.patients.',low.high.groups)] > 0) > 1) {
    # run logrank test
    logrank.stats <- BoutrosLab.statistics.survival::logrank.analysis(
      survival.object,
      factor(paste((as.numeric(profiles$tc[i,]) > split.tc),(as.numeric(profiles$tac[i,]) > split.tac
      );
    gene.survival[i-row.start+1,'logrank.pvalue'] <- signif(logrank.stats$pvalue[1],digits=5);
    }
  }
rownames(gene.survival) <- rownames(profiles$tc)[row.start:row.end];
```

Print these results to file to later be combined with all the other results run in parallel

```
survival.result.file <- paste0(
  dataset.files$univariate.survival.output.dir,,
  dataset.name,'_tc_tac_interaction_survival_result_rows_',row.start,'_',row.end,'.txt'
  );
write.table(
  x = gene.survival,
  file = survival.result.file,
  sep = '\t',
  row.names = TRUE,
  col.names = TRUE,
  quote=FALSE
  );
```

23 Figure 4 - TC-TAC gene interactions

Set up the environment

```
library(BoutrosLab.plotting.general);
library(yaml);
dataset.name <- 'Metabric';
profile.types <- c('bulk','tc','tac');
```

Yaml file name

```
yaml.file <- paste0(dataset.name,'.yaml');
```

Read yaml with file information

```
dataset.files <- yaml.load_file(yaml.file);
```

Load clinical annotation

```
patient.anno <- read.table(dataset.files$clinical.annotation.file,sep='\t',header=TRUE);
```

Load interaction survival results

```
survival.results <- read.table(
  file=paste0(
    dataset.files$univariate.survival.output.dir,
    dataset.name,'_tc_tac_interaction_per_gene_survival_results.txt'
    ),
    header=TRUE,
    sep='\t'
    );
```

Determine the which terms are significant for each genes from the interaction models

```
km.class <- list();
for(term in c('tc','tac','interaction')) {
  survival.results[[paste0('km.class.',term)]] <- rep('?',nrow(survival.results));
  survival.results[[paste0('km.class.',term)]][which(
    survival.results[,paste0('coxzph.pvalue.',term)] > 0.01
    )] <- '~';
  survival.results[[paste0('km.class.',term)]][which(
    survival.results[,paste0('wald.qvalue.',term)] < 0.1 &
    survival.results[,paste0('coxzph.pvalue.',term)] > 0.01 &
    apply(
      survival.results[
      c('num.patients.tc.low.tac.low','num.patients.tc.high.tac.low','num.patients.tc.low.tac.high','
      ] > 35,
      1,
      all
      ) &
    log2(survival.results[,paste0('hr.',term)]) > 0.4
    )] <- '<';
  survival.results[[paste0('km.class.',term)]][which(
    survival.results[,paste0('wald.qvalue.',term)] < 0.1 &
    survival.results[,paste0('coxzph.pvalue.',term)] > 0.01 &
    apply(survival.results[,c('num.patients.tc.low.tac.low','num.patients.tc.high.tac.low','num.patie
    log2(survival.results[,paste0('hr.',term)]) < -0.4)
    ] <- '>';
  }
survival.results$km.pattern <- paste0(
  '0',
  survival.results$km.class.tac,
  'tac,0',
  survival.results$km.class.tc,
  'tc,0',
  survival.results$km.class.interaction,
  'int'
  );
survival.results$km.pattern <- gsub('~','?',survival.results$km.pattern);
```

Remove genes that don't have a significant term from the interaction model

```
survival.results <- survival.results[survival.results$km.pattern != '0?tac,0?tc,0?int',];
```

Remove genes that don't have a significant term from the interaction model

```
survival.results <- survival.results[apply(
  survival.results[,c('km.class.tc','km.class.tac','km.class.interaction')] != '?',
  1,
  all
  ),];
```

Select the unique km significance patterns

```
km.pattern.uniq <- names(rev(sort(table(survival.results$km.pattern))));
```

Order the survival results by logrank q-values

```
survival.results <- survival.results[order(survival.results$logrank.pvalue),];
survival.results <- survival.results[order(survival.results$logrank.qvalue),];
```

Select two genes from each interaction model significance

```
genes.idx <- sort(as.numeric(sapply(
  km.pattern.uniq,
  function(x) {
    which(survival.results$km.pattern == x)[order(survival.results$logrank.qvalue[which(survival.resu
    }
  )));
num.genes.to.show <- length(genes.idx);
group.col <- c('gray60','gold','navy','forestgreen');
```

Figure 4G - barpplot of the proportion of patients

```
total.num.patients <- sum(survival.results[
  1,
  c(
    'num.patients.tc.low.tac.low',
    'num.patients.tc.low.tac.high',
    'num.patients.tc.high.tac.low',
    'num.patients.tc.high.tac.high'
    )
  ]);
proportion.barplot <- create.barplot(
  y ~ x,
  data.frame(
    y = as.numeric(as.matrix(survival.results[
      genes.idx,
      c(
        'num.patients.tc.low.tac.low',
        'num.patients.tc.low.tac.high',
        'num.patients.tc.high.tac.low',
        'num.patients.tc.high.tac.high'
        )
      ]))/total.num.patients,
    x = rep(1:num.genes.to.show,4)
    ),
  groups=rep(1:4,each=num.genes.to.show),
  col=group.col,
  stack=TRUE,
  yaxis.lab=seq(0,1,0.2),
  yat=seq(0,1,0.2),
  xaxis.lab=NULL,
  xat=-5,
```

```
  xlimits=c(0.5,num.genes.to.show+0.5),
  ylimits=c(0,1)
  );
```

Figure 4F - scatterplot showing 5 year survival of each group for each gene/column

```
y.values <- as.numeric(as.matrix(survival.results[
  genes.idx,
  c(
    'surv.5.years.tc.low.tac.low',
    'surv.5.years.tc.low.tac.high',
    'surv.5.years.tc.high.tac.low',
    'surv.5.years.tc.high.tac.high'
    )
  ]));
survival.5.year.plot <- create.scatterplot(
  y ~ x,
  data=data.frame(
    x=rep(1:num.genes.to.show,4),
    y=y.values
    ),
  groups=rep(1:4,each=num.genes.to.show),
  col=group.col,
  pch='-',
  cex=8,
  resolution=300,
  width=10,
  xaxis.lab=NULL,
  xaxis.rot=90,
  ylimits=c(min(y.values)-(max(y.values)-min(y.values))/10,1),
  yat=seq(ceiling((min(y.values)-(max(y.values)-min(y.values))/10)*10)/10,1,0.1),
  yaxis.lab=seq(ceiling((min(y.values)-(max(y.values)-min(y.values))/10)*10)/10,1,0.1),
  xat=-5,
  xlab.label='genes',
  ylab.label='5 year survival',
  xlimits=c(0.5,num.genes.to.show+0.5),
  xgrid.at=1:num.genes.to.show,
  ygrid.at=0,
  type=c('p','g'),
  alpha=0.8
  );
```

Figure 4H - dotmap showing the coefficient and significance of each of the terms (tc, tac, interaction)

```
spot.size.function <- function(x) { 1.15 * abs(x); };
spot.colour.function <- function(x) {
  colours <- rep('white', length(x));
  colours[sign(x) == -1] <- default.colours(2, palette.type = 'dotmap')[1];
  colours[sign(x) ==  1] <- default.colours(2, palette.type = 'dotmap')[2];
  return(colours);
  };
```

Get the gene symbols

```
gene.sym.mapping <- read.table(dataset.files$gene.symbol.file,sep='\t',header=TRUE);
gene.id <- sub('_at','',rownames(survival.results)[genes.idx]);
gene.sym <- as.character(gene.sym.mapping$GeneSymbol[match(gene.id, gene.sym.mapping$EntrezID)]);
spots.failed <- survival.results[genes.idx,c('km.class.tac','km.class.tc','km.class.interaction')] ==
hr.dotmap.data <-log2(survival.results[genes.idx,c('hr.tac','hr.tc','hr.interaction')]);
hr.dotmap.data[spots.failed] <- NA;
pval.dotmap.data <- log10(survival.results[
  genes.idx,
  c('wald.qvalue.tac','wald.qvalue.tc','wald.qvalue.interaction')
  ]);
pval.dotmap.data[spots.failed] <- 0;
pattern.dotmap <- create.dotmap(
  t(hr.dotmap.data),
  t(pval.dotmap.data),
  spot.size.function = spot.size.function,
  spot.colour.function = spot.colour.function,
  colour.scheme = c('black', 'white'),
  at=c(-7,-4,log10(0.05),-1,1),
  width=10,
  resolution=300,
  yaxis.lab=rep('',6),
  yaxis.tck=0,
  xaxis.lab=gene.sym,
  bg.alpha=1,
  xaxis.rot=90,
  pch=21,
  col.colour='white',
  row.colour='white',
  na.spot.size=2.5
  );
```

Create the labels for the left hand side y-axis labels

```
num.inside.cells <- 32;
cell.values <- rep(0,3*num.inside.cells^2);
for(i in 1:num.inside.cells) {
  cell.values <- c(
    cell.values,
    rep(1,num.inside.cells),
    rep(2,num.inside.cells),
    rep(1,num.inside.cells-i),
    rep(2,i)
    );
  }
pairs.matrix <- matrix(cell.values,ncol=2*num.inside.cells,nrow=3*num.inside.cells);
line.pairs.heatmap.labelled <- create.heatmap(
  t(pairs.matrix[nrow(pairs.matrix):1,]),
  at=c(-0.5,0.5,1.5,2.5,3.5),
  colour.scheme=group.col,
```

```
  cluster.dimensions='none',
  print.colour.key=FALSE,
  xaxis.lab=c('baseline','comparison'),
  xat=c(num.inside.cells/2,num.inside.cells*3/2)+0.5,
  xaxis.cex=0.5,
  yaxis.lab=NULL,
  yat=NULL,
  grid.row=TRUE,
  grid.col=TRUE,
  force.grid.row=TRUE,
  force.grid.col=TRUE,
  col.lines=num.inside.cells+0.5,
  row.lines=(1:2)*num.inside.cells+0.5
  );
```

Figure 4E - scatterplot showing the logrank q-value for survival difference of the group

```
pvalue.lineplot <- create.scatterplot(
  y ~ x,
  data=data.frame(
    y=-log10(survival.results[genes.idx,'logrank.qvalue']),
    x=as.factor(1:num.genes.to.show)
    ),
  resolution=300,
  width=10,
  xaxis.lab=NULL,
  xaxis.rot=90,
  ylimits=c(0,11),
  yat=seq(0,10,5),
  yaxis.lab=seq(0,10,5),
  xlimits=c(0.5,num.genes.to.show+0.5),
  xat=-5,
  xlab.label='genes',
  ylab.label='-log10 fdr adjusted p-value',
  xgrid.at=1:num.genes.to.show,
  ygrid.at=0,
  type=c('p','g')
  );
```

Combine parts E through H in one plot

```
create.multiplot(
  list(
    line.pairs.heatmap.labelled,
    pattern.dotmap,
    proportion.barplot,
    survival.5.year.plot,
    pvalue.lineplot
    ),
  plot.layout=c(2,4),
```

```
layout.skip=c(F,F,T,F,T,F,T,F),
filename = './figure4_e_h.pdf',
resolution=300,
y.relation='free',
x.relation='free',
width=16,
height=10,
ylab.label=c(expression('            -'*log[10]*' qvalue'),'\n\n         5 year survival','proporti
xlab.label=NULL,
panel.heights=c(0.5,1,1,0.5),
panel.widths=c(0.025,1),
y.spacing=-5,
x.spacing=-0.5,
main='   ',
ylab.padding=2,
xaxis.rot=90,
xaxis.cex=1.2,
yaxis.cex=1.5,
ylab.cex = 1.6,
left.padding=6.5,
bottom.padding=-2,
use.legacy.settings=TRUE
);
```

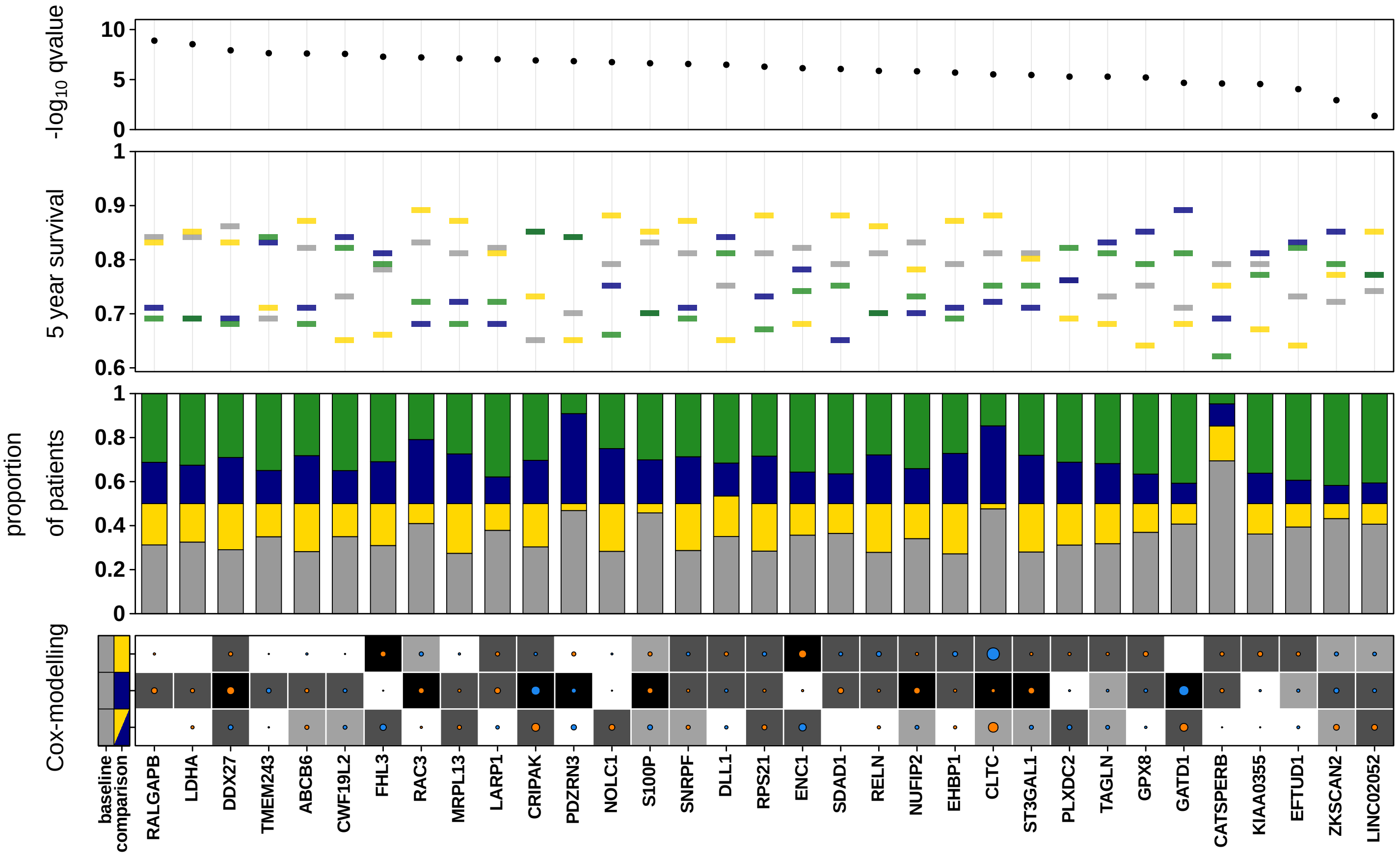

A-D

```
km.descriptors <- km.pattern.uniq;
```

Figure 4C - scatterplot showing 5 year survival

```
num.km.class.to.show <- 70;
if(length(km.descriptors) < num.km.class.to.show) {
  num.km.class.to.show <- length(km.descriptors);
  }
y.median.values <-as.numeric(sapply(
  1:num.km.class.to.show,
  function(x) {
    apply(
      survival.results[
        survival.results$km.pattern == km.descriptors[x],
        c(
          'surv.5.years.tc.low.tac.low',
          'surv.5.years.tc.low.tac.high',
          'surv.5.years.tc.high.tac.low',
          'surv.5.years.tc.high.tac.high'
          )
        ],
      2,
      median
      )
    }
  ));
y.values <- as.numeric(as.matrix(survival.results[,
  c(
    'surv.5.years.tc.low.tac.low',
    'surv.5.years.tc.low.tac.high',
    'surv.5.years.tc.high.tac.low',
    'surv.5.years.tc.high.tac.high'
    )
  ]));
random.point.plotting.order <- sample(1:(nrow(survival.results)*4));
survival.5.year.plot <- create.scatterplot(
  y ~ x,
  data=data.frame(
    y=y.values,
    x=rep(as.numeric(factor(survival.results$km.pattern,levels=km.descriptors)),4)
      +sample(1:(nrow(survival.results)*4))/((nrow(survival.results)+1)*4)*0.28-0.14
    )[random.point.plotting.order,],
  col=rep(group.col,each=nrow(survival.results))[random.point.plotting.order],
  pch=19,
  cex=0.3,
  resolution=300,
  width=10,
  xaxis.lab=NULL,
  xaxis.rot=90,
  ylimits=c(min(y.values)-(max(y.values)-min(y.values))/30,1),
```

```
  yat=seq(ceiling((min(y.values)-(max(y.values)-min(y.values))/30)*10)/10,1,0.2),
  yaxis.lab=seq(ceiling((min(y.values)-(max(y.values)-min(y.values))/30)*10)/10,1,0.2),
  xlimits=c(0.5,num.km.class.to.show+0.5),
  xat=NULL,
  xlab.label='most common km plot pattern',
  ylab.label='5 year survival    ',
  add.text=TRUE,
  text.x=as.numeric(sapply(
    rep(1:num.km.class.to.show,each=4),
    function(x){x+c(-0.16,-0.12,-0.08,-0.04,0,0.04,0.08,0.12,0.16)}
    )),
  text.y=rep(y.median.values,each=9),
  text.cex=2,
  text.labels='-',
  text.alpha=0.7,
  text.col=rep(group.col[rep(1:4,num.km.class.to.show)],each=9),
  add.rectangle=TRUE,
  xleft.rectangle=0.55,
  ybottom.rectangle=min(y.values)-(max(y.values)-min(y.values))/11,
  xright.rectangle=num.km.class.to.show+0.45,
  ytop.rectangle=max(y.values)+(max(y.values)-min(y.values))/11,
  alpha.rectangle=0.5,
  yaxis.alternating = 3,
  col.rectangle='white',
  foreground.rectangle =TRUE
  );
```

Figure 4A - barplot of the number of genes in each survival pattern

```
cat.count <- table(survival.results$km.pattern)[km.descriptors[1:num.km.class.to.show]];
pattern.count.plot <- create.barplot(
  y ~ x,
  data.frame(
    x=1:num.km.class.to.show,
    y=as.numeric(cat.count)
    ),
  width=10,
  resolution=300,
  ylab.label='Number of genes with km pattern',
  xat=-5,
  xlimits=c(0.5,num.km.class.to.show+0.5),
  xaxis.lab=NULL,
  yaxis.lab=c(0,200,400),
  yat=c(0,200,400)
  );
```

Figure 4D - heatmap labelling the survival patterns

```
km.patterns.heatmap <- unique(
  1*(survival.results[,c('km.class.tac','km.class.tc','km.class.interaction')] == '>')
```

```
  + 2*(survival.results[,c('km.class.tac','km.class.tc','km.class.interaction')] == '<')
  );
rownames(km.patterns.heatmap) <- unique(survival.results$km.pattern);
pattern.heatmap <- create.heatmap(
  km.patterns.heatmap[km.descriptors[1:num.km.class.to.show],3:1],
  at=c(-0.5,0.5,1.5,2.5),
  colour.scheme=c('white','dodgerblue2','darkorange1'),
  total.colours=3,
  resolution=300,
  cluster.dimensions='none',
  print.colour.key=FALSE,
  xaxis.lab=NULL,
  yaxis.lab=NULL,
  xat=NULL,
  yat=NULL
  );
pairs.matrix <- as.matrix(as.data.frame(list(baseline=c(0,0,0,1,1,2),otherline=c(1,2,3,2,3,3))));
num.inside.cells <- 32;
cell.values <- rep(0,3*num.inside.cells^2);
for(i in 1:num.inside.cells) {
  cell.values <- c(
    cell.values,
    rep(1,num.inside.cells),
    rep(2,num.inside.cells),
    rep(1,num.inside.cells-i),
    rep(2,i)
    );
  }
pairs.matrix <- matrix(cell.values,ncol=2*num.inside.cells,nrow=3*num.inside.cells);
line.pairs.heatmap.labelled <- create.heatmap(
  t(pairs.matrix[nrow(pairs.matrix):1,]),
  at=c(-0.5,0.5,1.5,2.5,3.5),
  colour.scheme=group.col,
  cluster.dimensions='none',
  print.colour.key=FALSE,
  xaxis.lab=c('baseline','comparison'),
  xat=c(num.inside.cells/2,num.inside.cells*3/2)+0.5,
  xaxis.cex=0.5,
  yaxis.lab=NULL,
  yat=NULL,
  grid.row=TRUE,
  grid.col=TRUE,
  force.grid.row=TRUE,
  force.grid.col=TRUE,
  col.lines=num.inside.cells+0.5,
  row.lines=(1:2)*num.inside.cells+0.5
  );
```

Figure 4B - boxplots of the logrank q-values for genes in each survival pattern

```
pvalue.boxplot <- create.boxplot(
  y ~ x,
  data=data.frame(
    y=-log10(survival.results$logrank.qvalue),
    x=factor(survival.results$km.pattern,levels=km.descriptors[1:num.km.class.to.show])
    ),
  resolution=300,
  width=10,
  xaxis.lab=NULL,
  xaxis.rot=90,
  ylimits=c(0,max(-log10(survival.results$logrank.qvalue))+0.5),
  xlimits=c(0.5,num.km.class.to.show+0.5),
  xat=-5,
  xlab.label='most common km plot pattern',
  ylab.label='-log10 fdr adjusted p-value'
  );
```

Create the legend for the entire figure

```
legend <- legend.grob(
  list(
    legend = list(
      colours = rep('transparent',1),
      labels = rep(' ',1),
      size = 3,
      label.cex = 1,
      border = 'transparent'
      ),
    legend = list(
      colours = rev(group.col),
      title = 'Patient Group',
      labels = rev(c(
        'mRNA(TAC) < median(TAC) & \nmRNA(TC) < median(TC)',
        'mRNA(TAC) > median(TAC) & \nmRNA(TC) < median(TC)',
        'mRNA(TAC) < median(TAC) & \nmRNA(TC) > median(TC)',
        'mRNA(TAC) > median(TAC) & \nmRNA(TC) > median(TC)'
        )),
      size = 3,
      border = 'black'
      ),
    legend = list(
      colours = c('white','dodgerblue2','darkorange1'),
      title = 'Cox-Modelling Classifications',
      labels = c(
        expression('q > 0.1 or |'*log[2]*'HR| < 0.4'),
        expression('q < 0.1 & '*log[2]*'HR > 0.4'),
        expression('q < 0.1 & '*log[2]*'HR < -0.4')
        ),
      size = 3,
      border = 'black'
```

```
      ),
    legend = list(
      colours = rep('transparent',6),
      title = expression(bold('Cox-Modelling '*log[2]*'HR')),
      labels = rep(' ',6),
      size = 3,
      title.cex = 1,
      label.cex = 1,
      border = 'transparent'
      ),
    legend = list(
      colours = colorRampPalette(c('white', 'black'))(4),
      title = 'Cox-Modelling q-values',
      labels = c(
        '0.05 to 1',
        '0.01 to 0.05',
        expression(10^{-4}*' to 0.01'),
        expression('< '*10^{-4})
        ),
      size = 3,
      title.cex = 1,
      label.cex = 1,
      border = 'black'
      )
    ),
  title.just = 'left',
  title.cex=1.5,
  label.cex=1.5
  );
```

Combine parts A through D in one plot

```
create.multiplot(
  list(line.pairs.heatmap.labelled,pattern.heatmap,survival.5.year.plot,pvalue.boxplot,pattern.count.
  plot.layout=c(2,4),
  layout.skip=c(F,F,T,F,T,F,T,F),
  filename = './figure4_a_d.pdf',
  use.legacy.settings=TRUE,
  resolution=300,
  y.relation='free',
  x.relation='free',
  width=16,
  height=8.9,
  ylab.label=c(
    ' number of genes',
    expression('-'*log[10]*' qvalue        '),
    '5 year survival              ',
    'patterns            '
    ),
  panel.heights=c(1,1,1,0.5),
```

```
  panel.widths=c(0.04,1),
  y.spacing=-5.5,
  x.spacing=-0.5,
  legend = list(right = list(fun = legend)),
  print.new.legend = TRUE,
  main=' ',
  ylab.padding=2,
  xaxis.rot=90,
  xaxis.cex=1.2,
  yaxis.cex=1.5,
  ylab.cex = 1.6,
  left.padding=6.5,
  key.right.padding=1.15,
  key = list(
    points = list(
      cex = spot.size.function(c(2,1,0,-1,-2)),
      col = 'black',
      fill = spot.colour.function(c(2,1,0,-1,-2)),
      pch = 21
      ),
    text = list(
      lab = c('+2.0','+1.0',' 0.0','-1.0','-2.0'),
      cex = 1.5,
      adj = 1
      ),
    padding.text = 3,
    x=1.033,
    y=0.37
    ));
```

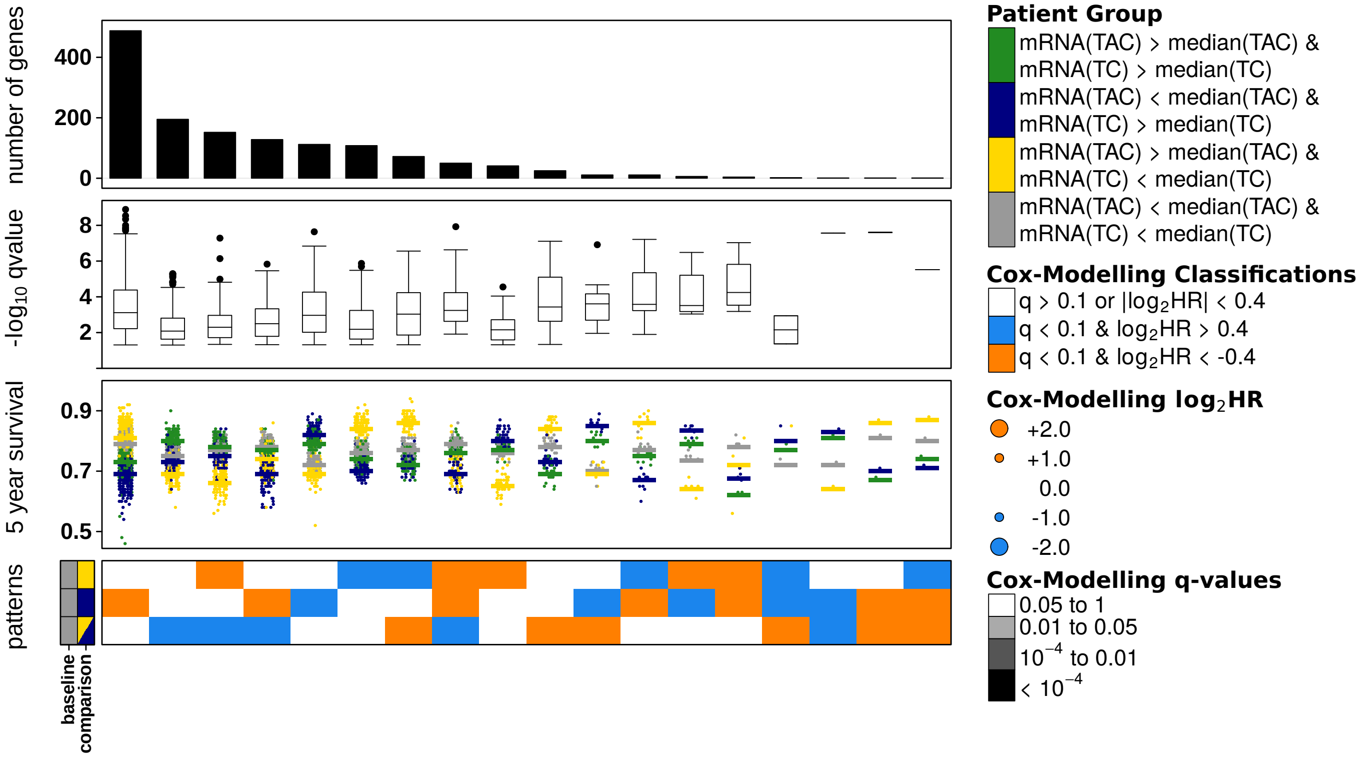

Pull out the information for supplementary table

```
write.table(
  survival.results[
    survival.results$km.class.interaction %in% c('>','<'),
    c('hr.interaction','wald.pvalue.interaction','wald.qvalue.interaction','coxzph.pvalue.interactio
    ],
  './4-survival_interactions_stable_genes_with_significant_tc_tac_interactions.txt',
  col.names=TRUE,
  row.names=TRUE,
  quote=FALSE,
  sep='\t'
  );
```

24 Supplementary Figure 8 - *FHL3*, *SDAD1*, *CRIPAK* prognosis

Set up the environment

```
library(BoutrosLab.plotting.survival);
library(yaml);
dataset.name <- 'Metabric';
```

Yaml file name

```
yaml.file <- paste0(dataset.name,'.yaml');
```

Read yaml with file information

```
dataset.files <- yaml.load_file(yaml.file);
```

Load clinical annotation

```
survival.anno <- read.table(dataset.files$clinical.annotation.file,sep='\t',header=TRUE);
```

Load mRNA abundance profiles

```
profile.types <- c('bulk','tc','tac');
rna.data.matrix <- list();
for(profile.type in profile.types) {
  rna.data.matrix[[profile.type]] <- read.table(
    dataset.files[[paste0(profile.type,'.mrna.abundance.file')]],
    header=TRUE,
    sep='\t'
    );
  }
common.patients <- intersect(
  colnames(rna.data.matrix$tc),
  rownames(survival.anno)[!is.na(survival.anno$Event)]
  );
survival.anno <- survival.anno[common.patients,];
for(profile.type in profile.types) {
  rna.data.matrix[[profile.type]] <- rna.data.matrix[[profile.type]][,common.patients];
  }
```

Collect survival data

```
time.to.event <- survival.anno$TimeToEvent[!is.na(survival.anno$TimeToEvent)];
censoring <- survival.anno$Event[!is.na(survival.anno$Event)];
censoring.time <- 5;
censoring[which(time.to.event > censoring.time)] <- 0;
time.to.event[which(time.to.event > censoring.time)] <- censoring.time;
time.to.event[time.to.event < 0] <- 0;
gene.ids <- c('2275_at','55153_at','285464_at');
gene.syms <- c('FHL3','SDAD1','CRIPAK');
for(j in 1:length(gene.ids)) {
  gene.id <- gene.ids[j];
  gene.sym <- gene.syms[j];
  i <- which(rownames(rna.data.matrix[['tc']]) == gene.id);
  tc.split <- median(as.numeric(rna.data.matrix$tc[i,]));
  if(tc.split < 6.5) {
    tc.split <- 6.5;
    }
  tac.split <- median(as.numeric(rna.data.matrix$tac[i,]));
  if(tac.split < 6.5) {
    tac.split <- 6.5;
    }
  bulk.split <- median(as.numeric(rna.data.matrix$bulk[i,]));
  if(bulk.split < 6.5) {
```

```
    bulk.split <- 6.5;
    }
  cox.model <- coxph(
    surv ~ tac + tumour+tumour*tac,
    data=data.frame(
      surv=Surv(time.to.event, censoring),
      tumour=factor((as.numeric(rna.data.matrix$tc[i,]) > tc.split), levels=c(FALSE,TRUE)),
      tac=factor((as.numeric(rna.data.matrix$tac[i,]) > tac.split),levels=c(FALSE,TRUE))
      )
    );
  patient.groups <- 2*(as.numeric(rna.data.matrix$tc[i,]) > tc.split) +
    (as.numeric(rna.data.matrix$tac[i,]) > tac.split);
  survival.result <- create.km.plot(
    survival.object = Surv(time.to.event, censoring),
    patient.groups = factor(patient.groups,level=3:0),
    filename = paste0('./sfigure8_',gene.sym,'_tc_tac.pdf'),
    xlab.label = 'Time (Years)',
    ylab.label = 'Survival',
    statistical.method = 'none',
    resolution = 100,
    show.risktable = FALSE,
    main=paste0(gene.sym,', ',gene.id),
    main.cex=1.5,
    line.colours=rev(c('gray60','gold','navy','forestgreen')),
    key.groups.labels=rev(c('TC low, TAC low','TC low, TAC high','TC high, TAC low','TC high, TAC hig
    risk.labels=rev(c('low','s high','t high','high'))
    );
  patient.groups <- as.numeric(rna.data.matrix$bulk[i,]) > bulk.split;
  survival.result <- create.km.plot(
    survival.object = Surv(time.to.event, censoring),
    patient.groups = factor(patient.groups,levels=c(TRUE,FALSE)),
    filename = paste0('./sfigure8_',gene.sym,'_bulk.pdf'),
    xlab.label = 'Time (Years)',
    ylab.label = 'Survival',
    statistical.method = 'none',
    ph.assumption.check='warning.and.plot',
    resolution = 100,
    show.risktable = FALSE,
    main=paste0(gene.sym,', ',gene.id),
    main.cex=1.5,
    line.colours=rev(c('gray60','red4')),
    key.groups.labels=c('Bulk high','Bulk low'),
    risk.labels=c('high','low'),
    cox.zph.threshold = 0.01
    );
  }
```

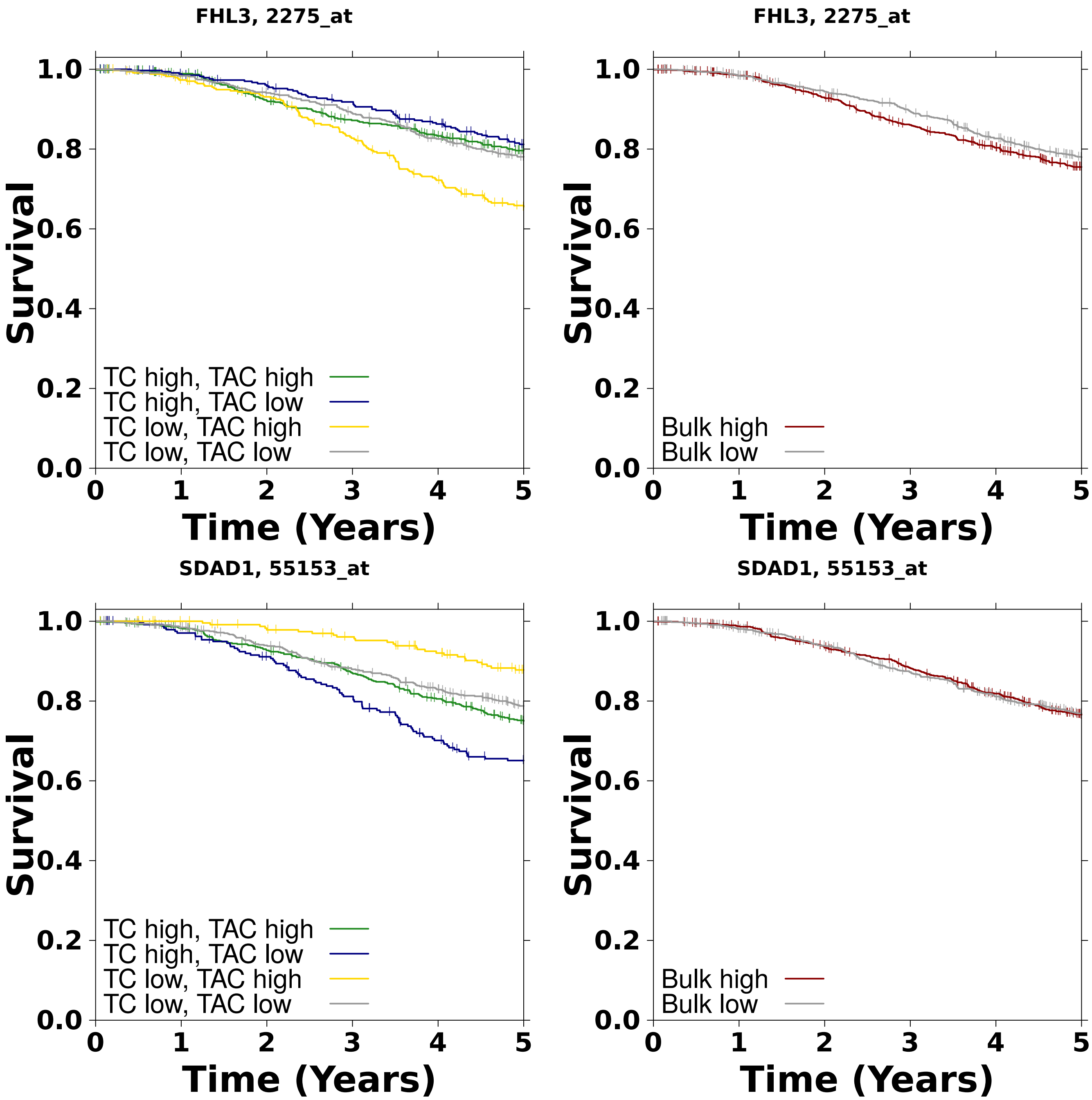
FHL3, 2275_at
Survival
Time (Years)
TC high, TAC high
TC high, TAC low
TC low, TAC high
TC low, TAC low
FHL3, 2275_at
Survival
Time (Years)
Bulk high
Bulk low
SDAD1, 55153_at
Survival
Time (Years)
TC high, TAC high
TC high, TAC low
TC low, TAC high
TC low, TAC low
SDAD1, 55153_at
Survival
Time (Years)
Bulk high
Bulk low

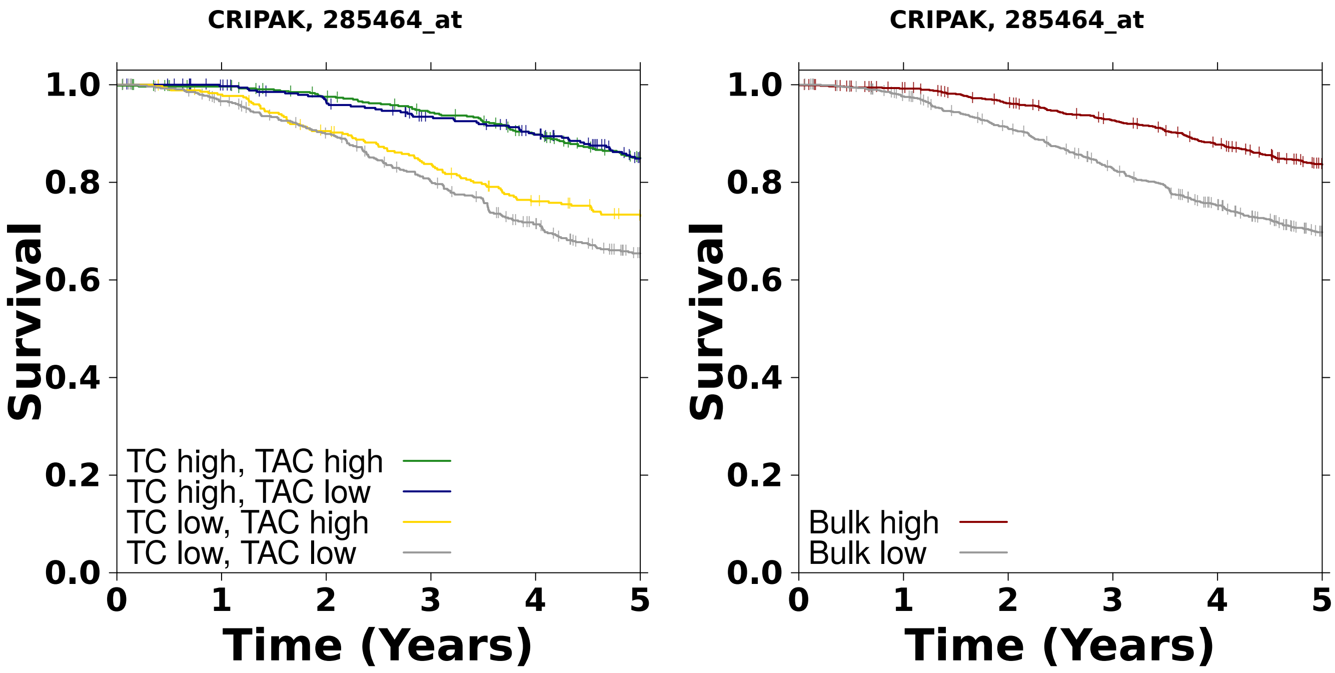

25 Supplementary Figure 9 - *FHL3*, *SDAD1*, *CRIPAK* prognosis per subtype

Set up the environment

```
library(BoutrosLab.plotting.survival);
library(yaml);
dataset.name <- 'Metabric';
```

Yaml file name

```
yaml.file <- paste0(dataset.name,'.yaml');
```

Read yaml with file information

```
dataset.files <- yaml.load_file(yaml.file);
```

Load clinical annotation

```
patient.anno <- read.table(dataset.files$clinical.annotation.file,sep='\t',header=TRUE);
```

Load mRNA abundance profiles

```
profile.types <- c('bulk','tc','tac');
rna.data.matrix <- list();
for(profile.type in profile.types) {
  rna.data.matrix[[profile.type]] <- read.table(
    dataset.files[[paste0(profile.type,'.mrna.abundance.file')]],
    header=TRUE,
    sep='\t'
```

```
    );
  }
common.patients <- intersect(
  colnames(rna.data.matrix$tc),
  rownames(patient.anno)[!is.na(patient.anno$Event)]
  );
patient.anno <- patient.anno[common.patients,];
for(profile.type in profile.types) {
  rna.data.matrix[[profile.type]] <- rna.data.matrix[[profile.type]][,common.patients];
  }
```

Collect survival data

```
time.to.event <- patient.anno$TimeToEvent[!is.na(patient.anno$TimeToEvent)];
censoring <- patient.anno$Event[!is.na(patient.anno$Event)];
censoring.time <- 5;
censoring[which(time.to.event > censoring.time)] <- 0;
time.to.event[which(time.to.event > censoring.time)] <- censoring.time;
time.to.event[time.to.event < 0] <- 0;
gene.ids <- c('2275_at','55153_at','285464_at');
gene.syms <- c('FHL3','SDAD1','CRIPAK');
for(subtype in unique(patient.anno$subtype)) {
  for(j in 1:3) {
    gene.id <- gene.ids[j];
    gene.sym <- gene.syms[j];
    i <- which(rownames(rna.data.matrix[['tc']]) == gene.id);
    tc.split <- median(as.numeric(rna.data.matrix$tc[i,]));
    if(tc.split < 6.5) {
      tc.split <- 6.5;
      }
    tac.split <- median(as.numeric(rna.data.matrix$tac[i,]));
    if(tac.split < 6.5) {
      tac.split <- 6.5;
      }
    bulk.split <- median(as.numeric(rna.data.matrix$bulk[i,]));
    if(bulk.split < 6.5) {
      bulk.split <- 6.5;
      }
    cox.model <- coxph(
      surv ~ tac + tumour+tumour*tac,
      data=data.frame(
        surv=Surv(time.to.event, censoring)[which(patient.anno$subtype == subtype)],
        tumour=factor(
          (as.numeric(rna.data.matrix$tc[i,]) > tc.split),
          levels=c(FALSE,TRUE)
          )[which(patient.anno$subtype == subtype)],
        tac=factor(
          (as.numeric(rna.data.matrix$tac[i,]) > tac.split),
          levels=c(FALSE,TRUE)
          )[which(patient.anno$subtype == subtype)]
```

```
        )
      );
    patient.groups <- 2*(as.numeric(rna.data.matrix$tc[i,]) > tc.split)+(as.numeric(rna.data.matrix$t
    survival.result <- create.km.plot(
      survival.object = Surv(time.to.event, censoring)[which(patient.anno$subtype == subtype)],
      patient.groups = factor(patient.groups,level=3:0)[which(patient.anno$subtype == subtype)],
      filename = paste0('./sfigure9_',gene.sym,'_',subtype,'.pdf'),
      xlab.label = 'Time (Years)',
      ylab.label = 'Survival',
      statistical.method = 'logrank',
      resolution = 100,
      show.risktable = FALSE,
      line.colours=rev(c('gray60','gold','navy','forestgreen'))[
        which(table(factor(patient.groups,level=3:0)[which(patient.anno$subtype == subtype)]) != 0)
        ],
      key.groups.labels=rev(c('TC low, TAC low','TC low, TAC high','TC high, TAC low','TC high, TAC h
        which(table(factor(patient.groups,level=3:0)[which(patient.anno$subtype == subtype)]) != 0)
        ],
      risk.labels=rev(c('low','s high','t high','high'))[
        which(table(factor(patient.groups,level=3:0)[which(patient.anno$subtype == subtype)]) != 0)
        ]
      );
    }
  }
```

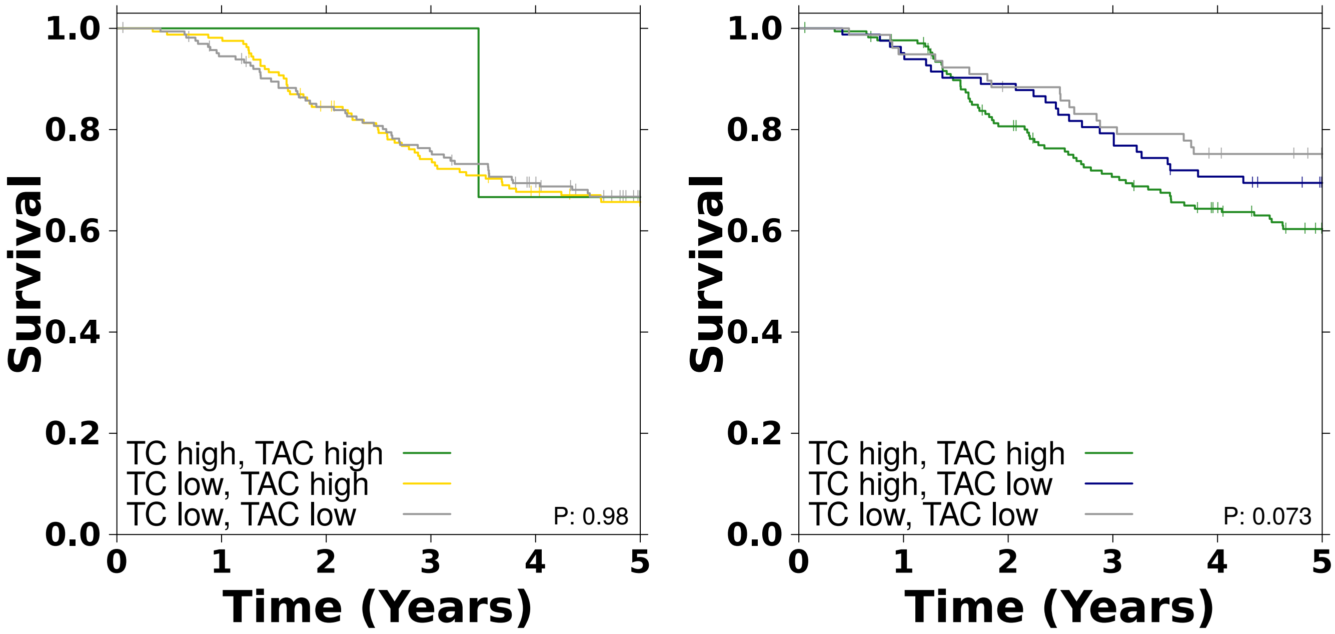

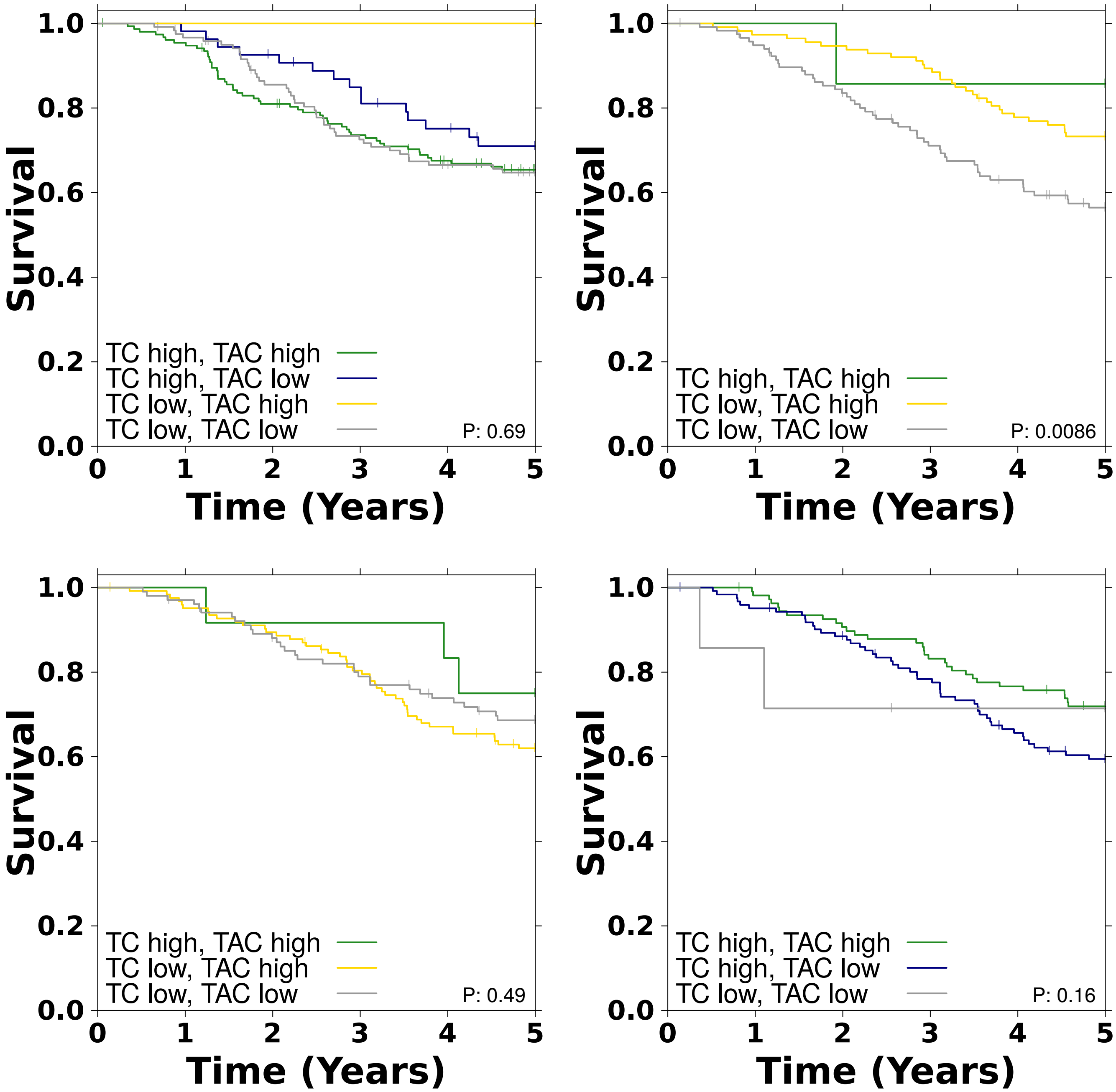
Survival
Time (Years)
TC high, TAC high
TC high, TAC low
TC low, TAC high
TC low, TAC low
P: 0.69
Survival
Time (Years)
TC high, TAC high
TC low, TAC high
TC low, TAC low
P: 0.0086
Survival
Time (Years)
TC high, TAC high
TC low, TAC high
TC low, TAC low
P: 0.49
Survival
Time (Years)
TC high, TAC high
TC high, TAC low
TC low, TAC low
P: 0.16

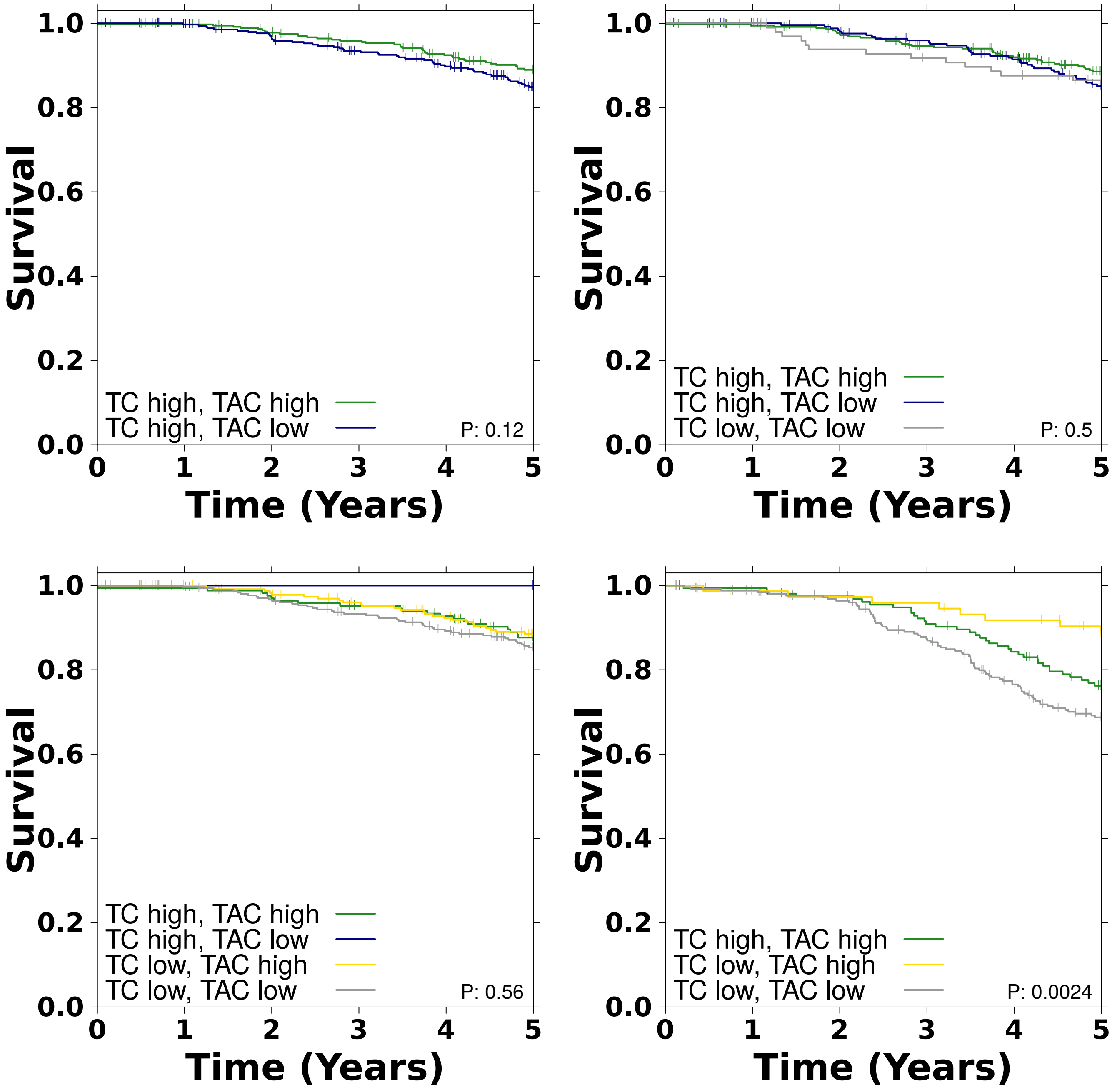
Survival
Time (Years)
TC high, TAC high
TC high, TAC low
P: 0.12
TC high, TAC high
TC high, TAC low
TC low, TAC low
P: 0.5
TC high, TAC high
TC high, TAC low
TC low, TAC high
TC low, TAC low
P: 0.56
TC high, TAC high
TC low, TAC high
TC low, TAC low
P: 0.0024

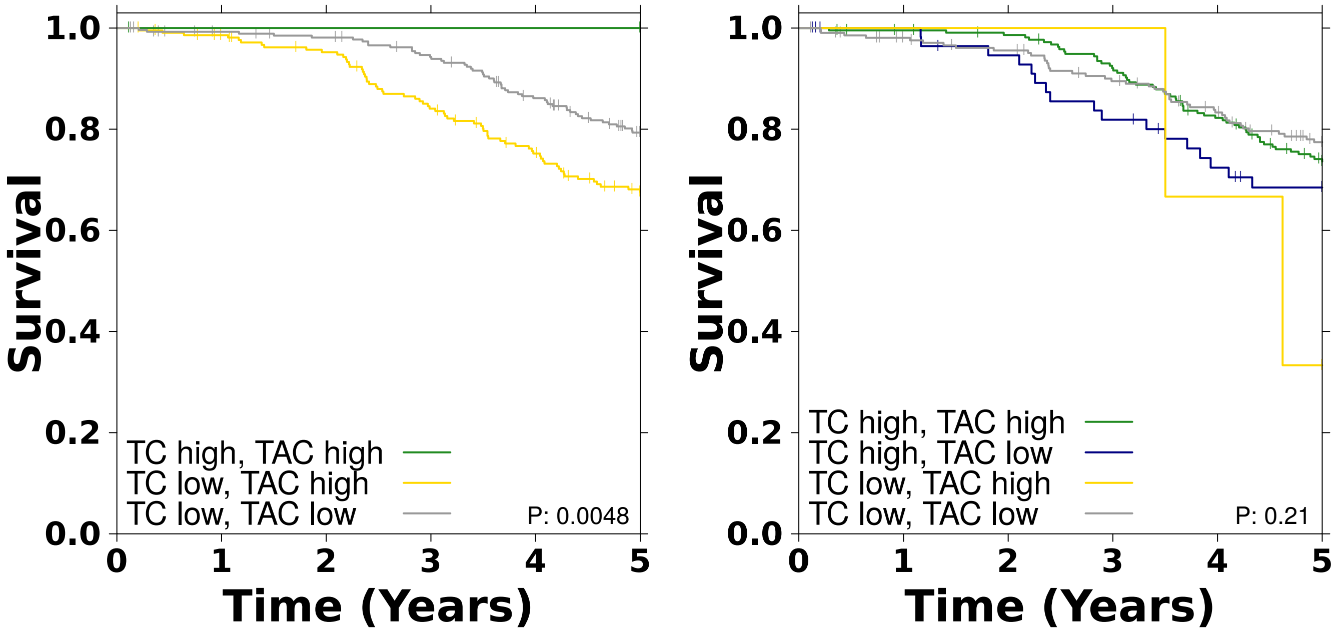

26 Create Random Multi-gene Biomarkers

Set up the environment

```
library(yaml);
library(randomForest);
library(pROC);
library(BoutrosLab.plotting.survival);
args <- commandArgs(trailingOnly = TRUE);
dataset.name <- args[1];
fold <- as.numeric(args[2]);
seed <- as.numeric(args[3]);
num.folds <- 3;
signature.size <- 50;
print(Sys.time());
```

Yaml file name

```
yaml.file <- paste0(dataset.name,'.yaml');
```

Read yaml with file information

```
dataset.files <- yaml.load_file(yaml.file);
```

Load clinical annotation

```
survival.anno <- read.table(dataset.files$clinical.annotation.file,sep='\t',header=TRUE);
survival.anno[['alive_at_5_years']] <- rep(NA,nrow(survival.anno));
survival.anno[['alive_at_5_years']][survival.anno$TimeToEvent <= 5 & survival.anno$Event == 1] <- FAI
survival.anno[['alive_at_5_years']][survival.anno$TimeToEvent > 5] <- TRUE;
survival.anno <- survival.anno[!is.na(survival.anno$alive_at_5_years),];
```

Load mRNA abundance profiles

```
rna.data.matrix <- list();
profile.types <- c('bulk','tc','tac');
rna.data.matrix[['bulk']] <- read.table(dataset.files$bulk.mrna.abundance.file,header=TRUE,sep='\t');
```

Filter patients to make sure the datasets match and filter out patients that were censored or have na for the survival time

```
common.patients <- intersect(colnames(rna.data.matrix[[1]]), rownames(survival.anno));
survival.anno <- survival.anno[common.patients,];
rna.data.matrix$bulk <- rna.data.matrix$bulk[,common.patients];
```

Generate the random signature, ie. select random rows from the RNA matrix to make the signature

```
set.seed(seed);
signature.genes <- sample(1:nrow(rna.data.matrix[[1]]),signature.size);
```

Load the TC and TAC profiles for the training/validation folds

```
for(cv.step in c('train','valid')) {
  # load TC profiles
  rna.data.matrix[[paste0('tc_',cv.step,'_fold',fold)]] <- read.table(
    paste0(
      dataset.files$isopure.output.dir,
      'mRNA_abundance_tc_results_',
      cv.step,'_fold',fold,'.txt.gz'
      ),
    header=TRUE,
    sep='\t'
    );
  rna.data.matrix[[paste0('tc_',cv.step,'_fold',fold)]] <-
    rna.data.matrix[[paste0('tc_',cv.step,'_fold',fold)]][
      signature.genes,
      intersect(common.patients,colnames(rna.data.matrix[[paste0('tc_',cv.step,'_fold',fold)]]))
      ];
  # load TAC profiles
  rna.data.matrix[[paste0('tac_',cv.step,'_fold',fold)]] <- read.table(
    paste0(
      dataset.files$isopure.output.dir,
      'mRNA_abundance_tac_results_',
      cv.step,'_fold',fold,'.txt.gz'
      ),
    header=TRUE,
    sep='\t'
    );
  rna.data.matrix[[paste0('tac_',cv.step,'_fold',fold)]] <-
    rna.data.matrix[[paste0('tac_',cv.step,'_fold',fold)]][
      signature.genes,
      intersect(common.patients,colnames(rna.data.matrix[[paste0('tac_',cv.step,'_fold',fold)]]))
```

```
      ];
  # create the bulk "fold" and the matching survival data for training/validation folds
  rna.data.matrix[[paste0('bulk_',cv.step,'_fold',fold)]] <-
    rna.data.matrix$bulk[
      signature.genes,
      colnames(rna.data.matrix[[paste0('tc_',cv.step,'_fold',fold)]])
      ];
  }
testing.actual.grouped <- survival.anno[colnames(rna.data.matrix[[paste0('bulk_valid_fold',fold)]]),'
if(sum(testing.actual.grouped) == 0 | sum(!testing.actual.grouped) == 0) {
  warning('There are not two groups in the actual classes');
  }
```

Generation some mtry values to parameterize on

```
mtry.options <- sort(unique(round(c(1,sqrt(signature.size)/seq(10,2,-3),sqrt(signature.size),sqrt(sig
mtry.options <- mtry.options[mtry.options <= signature.size];
mtry.options <- mtry.options[mtry.options >= 1];
```

Train and test the random forests for parameterization

```
data.to.record <- c('seed','mtry','fold','max.mtry','lower.auc','auc','upper.auc','vote.threshold','t
cv.results <- list();
for(profile.type in profile.types) {
  cv.results[[profile.type]] <- matrix(NA,nrow=length(mtry.options), ncol=length(data.to.record));
  colnames(cv.results[[profile.type]]) <- data.to.record;
  for(i in 1:length(mtry.options)) {
    mtry <- mtry.options[i];
    # train the random forest
    rf.result <- randomForest(
      formula = alive_at_5_years ~ .,
      y=factor(survival.anno[colnames(rna.data.matrix[[paste0(profile.type,'_train_fold',fold)]]),'al
      x=t(rna.data.matrix[[paste0(profile.type,'_train_fold',fold)]]),
      ntree=10000,
      mtry=mtry
      );
    # assess how good the random forest is
    valid.predictions <- predict(
      rf.result,
      t(rna.data.matrix[[paste0(profile.type,'_valid_fold',fold)]]),
      type='vote'
      )[,2];
    # calculate the AUC
    roc.result <- roc(response=testing.actual.grouped, predictor=valid.predictions);
    cv.results[[profile.type]][i,c('seed','mtry','fold','max.mtry','lower.auc','auc','upper.auc')] <-
    # calculate the TP and FP rates for each threshold
    possible.different.thresholds <- sort(unique(valid.predictions));
    tpr <- rep(NA,length(possible.different.thresholds));
    fpr <- rep(NA,length(possible.different.thresholds));
    for(j in 1:length(possible.different.thresholds)) {
```

```
      testing.predicted.grouped <- as.numeric(valid.predictions) > possible.different.thresholds[j];
      tpr[j] <- sum(testing.actual.grouped & testing.predicted.grouped)/sum(testing.actual.grouped);
      fpr[j] <- 1-sum(!testing.actual.grouped & !testing.predicted.grouped)/sum(!testing.actual.group
      }
    average.rates <- (tpr + 1 - fpr)/2;
    chosen.threshold <- which(average.rates == max(average.rates))[1];
    cv.results[[profile.type]][i,'vote.threshold'] <- round(possible.different.thresholds[chosen.thre
    valid.predictions.dichotomized <- as.numeric(valid.predictions) > cv.results[[profile.type]][i,'v
    tp <- sum(testing.actual.grouped & valid.predictions.dichotomized);
    tn <- sum(!testing.actual.grouped & !valid.predictions.dichotomized);
    fp <- sum(!testing.actual.grouped & valid.predictions.dichotomized);
    fn <- sum(testing.actual.grouped & !valid.predictions.dichotomized);
    cv.results[[profile.type]][i,c('tp','tn','fp','fn')] <- c(tp,tn,fp,fn);
    # calculate the HR at chosen therhold for dichotomizing the prediction score
    survobj <- Surv(survival.anno[colnames(rna.data.matrix[[paste0(profile.type,'_valid_fold',fold)]]
    cv.results[[profile.type]][i,c('hr','lower.hr','upper.hr','pvalue')] <- fit.coxmodel(
      groups = valid.predictions.dichotomized,
      survobj = survobj
      )[1:4];
    cox.model <- fit.coxmodel(
      groups = valid.predictions.dichotomized,
      survobj = survobj,
      return.cox.model=TRUE
      );
    cv.results[[profile.type]][i,'coxzph'] <- BoutrosLab.statistics.survival::ph.fails(cox.model,pval
    }
  # round the pvalue results to 5 decimals to help keep the files smaller. Generally we only report 2
  cv.results[[profile.type]][,c('pvalue','coxzph')] <- signif(cv.results[[profile.type]][,c('pvalue',
  # mark which mtry valid led to the highest AUC
  cv.results[[profile.type]][,'max.mtry'][cv.results[[profile.type]][,'auc'] == max(cv.results[[profi
  # save which genes are in the signature
  cv.results[[profile.type]][,'signature.probes'] <- rep(paste(rownames(rna.data.matrix[[paste0(profi
  # write results for the one profile type to file
  output.filename <- paste0(
    dataset.files$random.signatures.output.dir,
    profile.type,'_cv_results_fold',fold,'_random_signature',seed,'_size',signature.size,'.txt'
    );
  write.table(
    cv.results[[profile.type]],
    output.filename,
    sep='\t',
    row.names=FALSE,
    col.names=TRUE,
    quote=FALSE
    );
  # compress the output file
  system(paste0('gzip -9 ',output.filename));
  }
```

Adjust the tc and tac probe names so that they are unique

```
for(cv.step in c('train','valid')) {
  for(profile.type in profile.types) {
    rownames(rna.data.matrix[[paste0(profile.type,'_',cv.step,'_fold',fold)]]) <- paste0(profile.type
    }
  }
```

Set up the data and results matrix for combining the tc and tac datasets

```
profile.type <- 'tc_tac';
cv.results[[profile.type]] <- matrix(NA,nrow=length(mtry.options), ncol=length(data.to.record));
colnames(cv.results[[profile.type]]) <- data.to.record;
for(i in 1:length(mtry.options)) {
  mtry <- mtry.options[i];
  # train the random forest
  rf.result <- randomForest(
    formula = alive_at_5_years ~ .,
    y=factor(survival.anno[colnames(rna.data.matrix[[paste0('tc_train_fold',fold)]]),'alive_at_5_year
    x=t(rbind(rna.data.matrix[[paste0('tc_train_fold',fold)]],rna.data.matrix[[paste0('tac_train_fold
    ntree=10000,
    mtry=mtry
    );
  # assess how good the random forest is
  valid.predictions <- predict(
    rf.result,
    t(rbind(rna.data.matrix[[paste0('tc_valid_fold',fold)]],rna.data.matrix[[paste0('tac_valid_fold',
    type='vote'
    )[,2];
  # calculate the AUC
  roc.result <- roc(response=survival.anno[colnames(rna.data.matrix[[paste0('tc_valid_fold',fold)]]),
  cv.results[[profile.type]][i,c('seed','mtry','fold','max.mtry','lower.auc','auc','upper.auc')] <- c
  # calculate the TP and FP rates for each threshold
  possible.different.thresholds <- sort(unique(valid.predictions));
  tpr <- rep(NA,length(possible.different.thresholds));
  fpr <- rep(NA,length(possible.different.thresholds));
  for(j in 1:length(possible.different.thresholds)) {
    testing.predicted.grouped <- as.numeric(valid.predictions) > possible.different.thresholds[j];
    tpr[j] <- sum(testing.actual.grouped & testing.predicted.grouped)/sum(testing.actual.grouped);
    fpr[j] <- 1-sum(!testing.actual.grouped & !testing.predicted.grouped)/sum(!testing.actual.grouped
    }
  average.rates <- (tpr + 1 - fpr)/2;
  chosen.threshold <- which(average.rates == max(average.rates))[1];
  cv.results[[profile.type]][i,'vote.threshold'] <- round(possible.different.thresholds[chosen.thresh
  valid.predictions.dichotomized <- as.numeric(valid.predictions) > cv.results[[profile.type]][i,'vot
  tp <- sum(testing.actual.grouped & valid.predictions.dichotomized);
  tn <- sum(!testing.actual.grouped & !valid.predictions.dichotomized);
  fp <- sum(!testing.actual.grouped & valid.predictions.dichotomized);
  fn <- sum(testing.actual.grouped & !valid.predictions.dichotomized);
  cv.results[[profile.type]][i,c('tp','tn','fp','fn')] <- c(tp,tn,fp,fn);
  # calculate the HR at chosen threshold for dichotomizing the prediction score
  survobj <- Surv(survival.anno[colnames(rna.data.matrix[[paste0('tc_valid_fold',fold)]]),'TimeToEven
```

```
  cv.results[[profile.type]][i,c('hr','lower.hr','upper.hr','pvalue')] <- fit.coxmodel(
    groups = valid.predictions.dichotomized,
    survobj = survobj
    )[1:4];
  cox.model <- fit.coxmodel(
    groups = valid.predictions.dichotomized,
    survobj = survobj,
    return.cox.model=TRUE
    );
  cv.results[[profile.type]][i,'coxzph'] <- BoutrosLab.statistics.survival::ph.fails(cox.model,pvalue
  }
```

Round the pvalue results to 5 decimals to help keep the files smaller. Generally we only report 2 in the manuscript anyway

```
cv.results[[profile.type]][,c('pvalue','coxzph')] <- signif(cv.results[[profile.type]][,c('pvalue','c
```

Mark which mtry valid led to the highest AUC

```
cv.results[[profile.type]][,'max.mtry'][cv.results[[profile.type]][,'auc'] == max(cv.results[[profile
cv.results[[profile.type]][,'signature.probes'] <- rep(
  paste(
    c(
      rownames(rna.data.matrix[[paste0('tc_train_fold',fold)]]),
      rownames(rna.data.matrix[[paste0('tac_train_fold',fold)]])
      ),
    collapse=':'),
  nrow(cv.results[[profile.type]])
  );
```

Write results to file

```
output.filename <- paste0(
  dataset.files$random.signatures.output.dir,
  profile.type,'_cv_results_fold',fold,'_random_signature',seed,'_size',signature.size,'.txt'
  );
write.table(
  cv.results[[profile.type]],
  output.filename,
  sep='\t',
  row.names=FALSE,
  col.names=TRUE,
  quote=FALSE
  );
```

Compress the output file

```
system(paste0('gzip -9 ',output.filename));
message('finished successfully!');
```

27 Run Test Set Performance Random Multi-gene Biomarkers

Set up the environment

```
library(yaml);
library(randomForest);
library(pROC);
library(BoutrosLab.plotting.survival);
args <- commandArgs(trailingOnly = TRUE);
dataset.name <- args[1];
start.seed <- as.numeric(args[2]);
end.seed <- as.numeric(args[3]);
signature.size <- 50;
profile.types <- c('bulk','tc','tac','tc_tac');
```

Load cv run results and figure out which mtry value per random signature

```
cv.results <- list();
for(profile.type in profile.types) {
  combined.result <- NULL;
  for(i in seq(start.seed,end.seed,100)) {
    seed.min <- i;
    seed.max <- i+99;
    filename <- paste0(
      dataset.files$random.signatures.output.dir,
      profile.type,'_cv_results_combined_random_signatures_',
      seed.min,'_',seed.max,'_size',signature.size,'.txt.gz'
      );
    if(file.exists(filename)) {
      run.result <- read.table(filename,header=TRUE,sep='\t');
      combined.result <- rbind(combined.result,run.result);
      }
    }
  combined.result <- unique(combined.result);
  cv.results[[profile.type]] <- combined.result;
  }
for(profile.type in profile.types) {
  cv.results[[profile.type]]$avg.mtry.auc <- rep(NA,nrow(cv.results[[profile.type]]));
  cv.results[[profile.type]]$max.mtry.across.folds <- rep(0,nrow(cv.results[[profile.type]]));
  for(seed in unique(cv.results[[profile.type]]$seed)) {
    for(mtry in unique(cv.results[[profile.type]][cv.results[[profile.type]]$seed == seed,'mtry'])) {
      cv.results[[profile.type]]$avg.mtry.auc[
        cv.results[[profile.type]]$seed == seed &
        cv.results[[profile.type]]$mtry == mtry
        ] <-
          mean(cv.results[[profile.type]]$auc[
            cv.results[[profile.type]]$seed == seed &
            cv.results[[profile.type]]$mtry == mtry
            ]);
      }
    cv.results[[profile.type]]$max.mtry.across.folds[cv.results[[profile.type]]$seed == seed][which(c
```

```
    }
  }
best.parameters <- list();
for(profile.type in profile.types) {
  best.parameters[[profile.type]] <- cv.results[[profile.type]][cv.results[[profile.type]]$max.mtry.a
  best.parameters[[profile.type]] <- best.parameters[[profile.type]][order(best.parameters[[profile.t
  }
```

Yaml file name

```
yaml.file <- paste0(dataset.name,'.yaml');
```

Read yaml with file information

```
dataset.files <- yaml.load_file(yaml.file);
```

Load clinical annotation

```
survival.anno <- read.table(dataset.files$clinical.annotation.file,sep='\t',header=TRUE);
survival.anno[['alive_at_5_years']] <- rep(NA,nrow(survival.anno));
survival.anno[['alive_at_5_years']][survival.anno$TimeToEvent <= 5 & survival.anno$Event == 1] <- FAI
survival.anno[['alive_at_5_years']][survival.anno$TimeToEvent > 5] <- TRUE;
survival.anno <- survival.anno[!is.na(survival.anno$alive_at_5_years),];
```

Load mRNA abundance profiles

```
rna.data.matrix <- list();
profile.types <- c('bulk','tc','tac');
rna.data.matrix[['bulk']] <- read.table(dataset.files$bulk.mrna.abundance.file,header=TRUE,sep='\t');
```

Filter patients to make sure the datasets match and filter out patients that were censored or have na for the survival time

```
common.patients <- intersect(colnames(rna.data.matrix[[1]]), rownames(survival.anno));
survival.anno <- survival.anno[common.patients,];
rna.data.matrix$bulk <- rna.data.matrix$bulk[,common.patients];
```

Generate the random signature, ie. select random rows from the RNA matrix to make the signature

```
set.seed(seed);
signature.genes <- sample(1:nrow(rna.data.matrix[[1]]),signature.size);
```

Load the TC and TAC profiles for the test set

```
rna.data.matrix[['tc_test_set_orig']] <- read.table(
  paste0(
    dataset.files$isopure.output.dir,
    'mRNA_abundance_tc_results_test.txt.gz'
    ),
  header=TRUE,
```

```
  sep='\t'
  );
rna.data.matrix[['tac_test_set_orig']] <- read.table(
  paste0(
    dataset.files$isopure.output.dir,
    'mRNA_abundance_tac_results_test.txt.gz'
    ),
  header=TRUE,
  sep='\t'
  );
rna.data.matrix[['tc_train_set_orig']] <- read.table(
  paste0(
    dataset.files$isopure.output.dir,
    'mRNA_abundance_tc_results_train_all.txt.gz'
    ),
  header=TRUE,
  sep='\t'
  );
rna.data.matrix[['tac_train_set_orig']] <- read.table(
  paste0(
    dataset.files$isopure.output.dir,
    'mRNA_abundance_tac_results_train.txt.gz'
    ),
  header=TRUE,
  sep='\t'
  );
data.to.record <- c(
  'seed','mtry',
  'cv.avg.auc','lower.auc','auc','upper.auc',
  'vote.threshold',
  'tp','tn','fp','fn',
  'hr','lower.hr','upper.hr','pvalue','coxzph',
  'signature.probes'
  );
cv.results <- list();
for(profile.type in c('bulk','tc','tac')) {
  cv.results[[profile.type]] <- matrix(NA,nrow=length(best.parameters[[profile.type]]$seed), ncol=len
  colnames(cv.results[[profile.type]]) <- data.to.record;
  signatures <- rep(NA,length(best.parameters[[profile.type]]$seed));
  for(seed in best.parameters[[profile.type]]$seed) {
    # generate the random signature, ie. select random rows from the RNA matrix to make the signature
    set.seed(seed);
    signature.genes <- sample(1:nrow(rna.data.matrix[[1]]),signature.size);
    rna.data.matrix[['tc_train_set']] <- rna.data.matrix$tc_train_set_orig[signature.genes,intersect(
    rna.data.matrix[['tac_train_set']] <- rna.data.matrix$tac_train_set_orig[signature.genes,intersec
    rna.data.matrix[['bulk_train_set']] <- rna.data.matrix$bulk[signature.genes,colnames(rna.data.mat
    rna.data.matrix[['tc_test_set']] <- rna.data.matrix$tc_test_set_orig[signature.genes,intersect(co
    rna.data.matrix[['tac_test_set']] <- rna.data.matrix$tac_test_set_orig[signature.genes,intersect(
    rna.data.matrix[['bulk_test_set']] <- rna.data.matrix$bulk[signature.genes,colnames(rna.data.matr
    signatures[best.parameters[[profile.type]]$seed == seed] <- paste(rownames(rna.data.matrix[['tc_t
```

```
testing.actual.grouped <- survival.anno[colnames(rna.data.matrix$bulk_test_set),'alive_at_5_years
# train and test the random forests for parameterization
mtry <- best.parameters[[profile.type]]$mtry[best.parameters[[profile.type]]$seed == seed];
# train the random forest
rf.result <- randomForest(
  formula = alive_at_5_years ~ .,
  y=factor(survival.anno[colnames(rna.data.matrix[[paste0(profile.type,'_train_set')]]),'alive_at
  x=t(rna.data.matrix[[paste0(profile.type,'_train_set')]]),
  ntree=10000,
  mtry=mtry
  );
# assess how good the random forest is
valid.predictions <- predict(
  rf.result,
  t(rna.data.matrix[[paste0(profile.type,'_test_set')]]),
  type='vote'
  )[,2];
# calculate the AUC
roc.result <- roc(response=testing.actual.grouped, predictor=valid.predictions);
cv.results[[profile.type]][best.parameters[[profile.type]]$seed == seed,c('seed','mtry','cv.avg.a
# calculate the TP and FP rates for each threshold
possible.different.thresholds <- sort(unique(valid.predictions));
tpr <- rep(NA,length(possible.different.thresholds));
fpr <- rep(NA,length(possible.different.thresholds));
for(j in 1:length(possible.different.thresholds)) {
  testing.predicted.grouped <- as.numeric(valid.predictions) > possible.different.thresholds[j];
  tpr[j] <- sum(testing.actual.grouped & testing.predicted.grouped)/sum(testing.actual.grouped);
  fpr[j] <- 1-sum(!testing.actual.grouped & !testing.predicted.grouped)/sum(!testing.actual.group
  }
average.rates <- (tpr + 1 - fpr)/2;
chosen.threshold <- which(average.rates == max(average.rates))[1];
cv.results[[profile.type]][best.parameters[[profile.type]]$seed == seed,'vote.threshold'] <- rour
valid.predictions.dichotomized <- as.numeric(valid.predictions) > cv.results[[profile.type]][best
tp <- sum(testing.actual.grouped & valid.predictions.dichotomized);
tn <- sum(!testing.actual.grouped & !valid.predictions.dichotomized);
fp <- sum(!testing.actual.grouped & valid.predictions.dichotomized);
fn <- sum(testing.actual.grouped & !valid.predictions.dichotomized);
cv.results[[profile.type]][best.parameters[[profile.type]]$seed == seed,c('tp','tn','fp','fn')] <
# calculate the HR at chosen therhold for dichotomizing the prediction score
survobj <- Surv(survival.anno[colnames(rna.data.matrix[[paste0(profile.type,'_test_set')]]),'Time
cv.results[[profile.type]][best.parameters[[profile.type]]$seed == seed,c('hr','lower.hr','upper.
  groups = valid.predictions.dichotomized,
  survobj = survobj
  )[1:4];
cox.model <- fit.coxmodel(
  groups = valid.predictions.dichotomized,
  survobj = survobj,
  return.cox.model=TRUE
  );
cv.results[[profile.type]][best.parameters[[profile.type]]$seed == seed,'coxzph'] <- BoutrosLab.s
```

```
    }
  # round the pvalue results to 5 decimals to help keep the files smaller. Generally we only report 2
  cv.results[[profile.type]][,c('pvalue','coxzph')] <- signif(cv.results[[profile.type]][,c('pvalue',
  # save which genes are in the signature
  cv.results[[profile.type]][,'signature.probes'] <- signatures;
  # write results for the one profile type to file
  output.filename <- paste0(
    dataset.files$random.signatures.output.dir,
    profile.type,'_test_set_results_random_signature_combined_',
    seed.min,'_',seed.max,'_size',signature.size,'.txt'
    );
  write.table(
    cv.results[[profile.type]],
    output.filename,
    sep='\t',
    row.names=FALSE,
    col.names=TRUE,
    quote=FALSE
    );
  # compress the output file
  system(paste0('gzip -9 ',output.filename));
  }
```

Set up the data and results matrix for combining the tc and tac datasets

```
profile.type <- 'tc_tac';
cv.results[[profile.type]] <- matrix(NA,nrow=length(best.parameters[[profile.type]]$seed), ncol=lengt
colnames(cv.results[[profile.type]]) <- data.to.record;
signatures <- rep(NA,length(best.parameters[[profile.type]]$seed));
for(seed in best.parameters[[profile.type]]$seed) {
  # generate the random signature, ie. select random rows from the RNA matrix to make the signature
  set.seed(seed);
  signature.genes <- sample(1:nrow(rna.data.matrix[[1]]),signature.size);
  rna.data.matrix[['tc_train_set']] <- rna.data.matrix$tc_train_set_orig[signature.genes,intersect(co
  rownames(rna.data.matrix[['tc_train_set']]) <- paste0('tc_',rownames(rna.data.matrix[['tc_train_set
  rna.data.matrix[['tac_train_set']] <- rna.data.matrix$tac_train_set_orig[signature.genes,intersect(
  rownames(rna.data.matrix[['tac_train_set']]) <- paste0('tac_',rownames(rna.data.matrix[['tac_train_
  rna.data.matrix[['tc_test_set']] <- rna.data.matrix$tc_test_set_orig[signature.genes,intersect(comm
  rownames(rna.data.matrix[['tc_test_set']]) <- paste0('tc_',rownames(rna.data.matrix[['tc_test_set']
  rna.data.matrix[['tac_test_set']] <- rna.data.matrix$tac_test_set_orig[signature.genes,intersect(co
  rownames(rna.data.matrix[['tac_test_set']]) <- paste0('tac_',rownames(rna.data.matrix[['tac_test_se
  signatures[best.parameters[[profile.type]]$seed == seed] <- paste(c(rownames(rna.data.matrix[['tc_t
  testing.actual.grouped <- survival.anno[colnames(rna.data.matrix$bulk_test_set),'alive_at_5_years']
  # train and test the random forests for parameterization
  mtry <- best.parameters[[profile.type]]$mtry[best.parameters[[profile.type]]$seed == seed];
  # train the random forest
  rf.result <- randomForest(
    formula = alive_at_5_years ~ .,
    y=factor(survival.anno[colnames(rna.data.matrix$tc_train_set),'alive_at_5_years'],levels=c(FALSE,
    x=t(rbind(rna.data.matrix$tc_train_set,rna.data.matrix$tac_train_set)),
```

```
    ntree=10000,
    mtry=mtry
    );
  # assess how good the random forest is
  valid.predictions <- predict(
    rf.result,
    t(rbind(rna.data.matrix$tc_test_set,rna.data.matrix$tac_test_set)),
    type='vote'
    )[,2];
  # calculate the AUC
  roc.result <- roc(response=survival.anno[colnames(rna.data.matrix$tc_test_set),'alive_at_5_years'],
  cv.results[[profile.type]][best.parameters[[profile.type]]$seed == seed,c('seed','mtry','cv.avg.auc
  # calculate the TP and FP rates for each threshold
  possible.different.thresholds <- sort(unique(valid.predictions));
  tpr <- rep(NA,length(possible.different.thresholds));
  fpr <- rep(NA,length(possible.different.thresholds));
  for(j in 1:length(possible.different.thresholds)) {
    testing.predicted.grouped <- as.numeric(valid.predictions) > possible.different.thresholds[j];
    tpr[j] <- sum(testing.actual.grouped & testing.predicted.grouped)/sum(testing.actual.grouped);
    fpr[j] <- 1-sum(!testing.actual.grouped & !testing.predicted.grouped)/sum(!testing.actual.grouped
    }
  average.rates <- (tpr + 1 - fpr)/2;
  chosen.threshold <- which(average.rates == max(average.rates))[1];
  cv.results[[profile.type]][best.parameters[[profile.type]]$seed == seed,'vote.threshold'] <- round(
  valid.predictions.dichotomized <- as.numeric(valid.predictions) > cv.results[[profile.type]][best.p
  tp <- sum(testing.actual.grouped & valid.predictions.dichotomized);
  tn <- sum(!testing.actual.grouped & !valid.predictions.dichotomized);
  fp <- sum(!testing.actual.grouped & valid.predictions.dichotomized);
  fn <- sum(testing.actual.grouped & !valid.predictions.dichotomized);
  cv.results[[profile.type]][best.parameters[[profile.type]]$seed == seed,c('tp','tn','fp','fn')] <-
  # calculate the HR at chosen therhold for dichotomizing the prediction score
  survobj <- Surv(survival.anno[colnames(rna.data.matrix$tc_test_set),'TimeToEvent'], survival.anno[c
  cv.results[[profile.type]][best.parameters[[profile.type]]$seed == seed,c('hr','lower.hr','upper.hr
    groups = valid.predictions.dichotomized,
    survobj = survobj
    )[1:4];
  cox.model <- fit.coxmodel(
    groups = valid.predictions.dichotomized,
    survobj = survobj,
    return.cox.model=TRUE
    );
  cv.results[[profile.type]][best.parameters[[profile.type]]$seed == seed,'coxzph'] <- BoutrosLab.sta
  }
```

Round the pvalue results to 5 decimals to help keep the files smaller. Generally we only report 2 in the manuscript anyway

```
cv.results[[profile.type]][,c('pvalue','coxzph')] <- signif(cv.results[[profile.type]][,c('pvalue','c
```

Save which genes are in the signature

```
cv.results[[profile.type]][,'signature.probes'] <- signatures;
```

Write results for the one profile type to file

```
output.filename <- paste0(
  dataset.files$random.signatures.output.dir,
  profile.type,'_test_set_results_random_signature_combined_',
  seed.min,'_',seed.max,'_size',signature.size,'.txt'
  );
write.table(
  cv.results[[profile.type]],
  output.filename,
  sep='\t',
  row.names=FALSE,
  col.names=TRUE,
  quote=FALSE
  );
```

Compress the output file

```
system(paste0('gzip -9 ',output.filename));
message('finished successfully!');
```

28 Figure 5 - Biomarker generation

Set up the environment

```
library(BoutrosLab.plotting.general);
library(VennDiagram);
library(yaml);
dataset.name <- 'Metabric';
signature.size <- 50;
```

Yaml file name

```
yaml.file <- paste0(dataset.name,'.yaml');
```

Read yaml with file information

```
dataset.files <- yaml.load_file(yaml.file);
```

Load clinical annotation

```
patient.anno <- read.table(dataset.files$clinical.annotation.file,sep='\t',header=TRUE);
```

Load mRNA abundance profiles

```r
profile.types <- c('bulk','tc','tac','tc_tac');
```

Load test set survival results

```r
cv.results <- list();
cv.results.auc <- list();
cv.results.log2.hr <- list();
for(profile.type in profile.types) {
  combined.result <- NULL;
  for(i in seq(1,5000,100)) {
    seed.min <- i;
    seed.max <- i+99;
    filename <- paste0(
      dataset.files$random.signatures.output.dir,
      dataset.name,
      '/',
      profile.type,
      '_test_set_results_random_signature_combined_',
      seed.min,
      '_',
      seed.max,
      '_size50.txt.gz'
      );
    if(file.exists(filename)) {
      run.result <- read.table(filename,header=TRUE,sep='\t');
      combined.result <- rbind(combined.result,run.result);
      }
    }
  combined.result <- unique(combined.result);
  cv.results[[profile.type]] <- combined.result;
  cv.results.auc[[profile.type]] <- combined.result$auc;
  cv.results.log2.hr[[profile.type]] <- -log2(combined.result$hr);
  }
```

Create a density plot for the AUCs

```r
create.densityplot(
  cv.results.auc,
  filename = './figure5a.pdf',
  resolution=300,
  col=c('firebrick','dodgerblue','gold','chartreuse4'),
  xlimits=c(0.5,0.7),
  xat=seq(0.5,0.8,0.1),
  xlab.label='AUC',
  type=c('l','g'),
  xgrid.at=0.5,
  ygrid.at=0,
  legend = list(
    inside = list(
      fun = draw.key,
```

```
      args = list(
        key = list(
          lines = list(
            col = c('firebrick','dodgerblue','gold','chartreuse4'),
            lwd=2.5
            ),
          text = list(
            lab = c('Bulk','TC','TAC','TC-TAC')
            ),
          cex = 1.5
          )
        ),
      x = 0.05,
      y = 0.97,
      draw = FALSE
      )
    )
  );
```

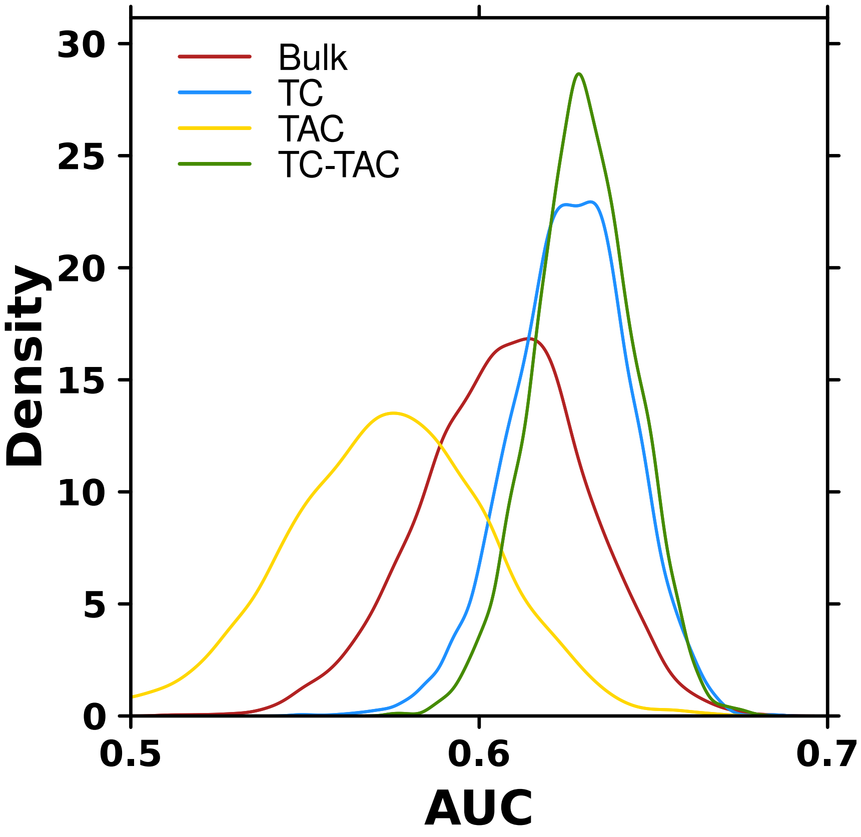

Create a zoom in of the density plot

```
create.densityplot(
  cv.results.auc,
  filename = './figure5b.pdf',
  resolution=300,
  col=c('firebrick','dodgerblue','gold','chartreuse4'),
```

```
  xlimits=c(0.65,0.7),
  ylimits=c(0,4),
  yat=seq(0,4,1),
  xat=seq(0.6,0.7,0.02),
  xlab.label='AUC',
  type=c('l','g'),
  xgrid.at=0.5,
  ygrid.at=0,
  right.padding=0.6
  );
```

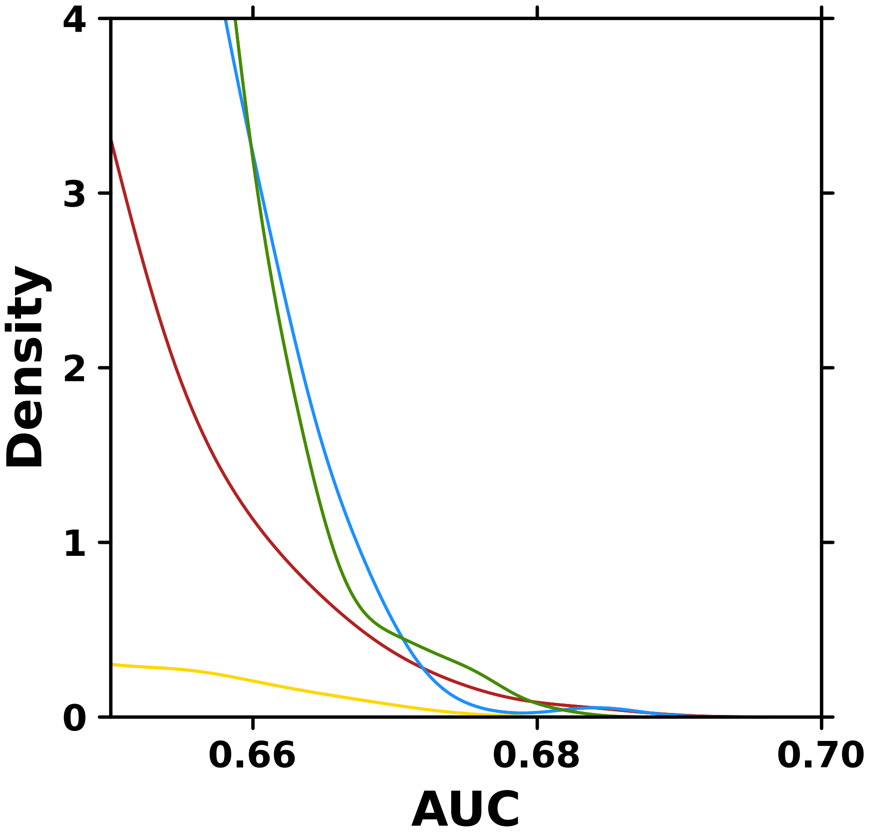

Calculate counts for AUC bins

```
bin.x <- seq(0.65,0.68,0.01);
barplot.data <- data.frame(
  count=c(
    sapply(bin.x,function(x) {sum(cv.results.auc[[1]] > x & cv.results.auc[[1]] <= x+0.01)}),
    sapply(bin.x,function(x) {sum(cv.results.auc[[2]] > x & cv.results.auc[[2]] <= x+0.01)}),
    sapply(bin.x,function(x) {sum(cv.results.auc[[3]] > x & cv.results.auc[[3]] <= x+0.01)}),
    sapply(bin.x,function(x) {sum(cv.results.auc[[4]] > x & cv.results.auc[[4]] <= x+0.01)})
    ),
  profile.type=factor(rep(c('firebrick','dodgerblue','gold','chartreuse4'),each=length(bin.x)),levels
  bin=factor(rep(bin.x,4))
  );
barplot.data;

   count profile.type  bin
```

```
1      90    firebrick 0.65
2      31    firebrick 0.66
3       8    firebrick 0.67
4       2    firebrick 0.68
5     266   dodgerblue 0.65
6      79   dodgerblue 0.66
7       5   dodgerblue 0.67
8       2   dodgerblue 0.68
9      13         gold 0.65
10      8         gold 0.66
11      0         gold 0.67
12      0         gold 0.68
13    335  chartreuse4 0.65
14     64  chartreuse4 0.66
15     13  chartreuse4 0.67
16      1  chartreuse4 0.68
```

Create a density plot for the HRs

```
create.densityplot(
  cv.results.log2.hr,
  filename = './figure5c.pdf',
  resolution=300,
  col=c('firebrick','dodgerblue','gold','chartreuse4'),
  xlab.label=expression(bold(log[2]*'HR')),
  xlimits=c(-0.5,2.5),
  xat=seq(-0.5,2.5,0.5),
  type=c('l','g'),
  xgrid.at=0,
  ygrid.at=0
  );
```

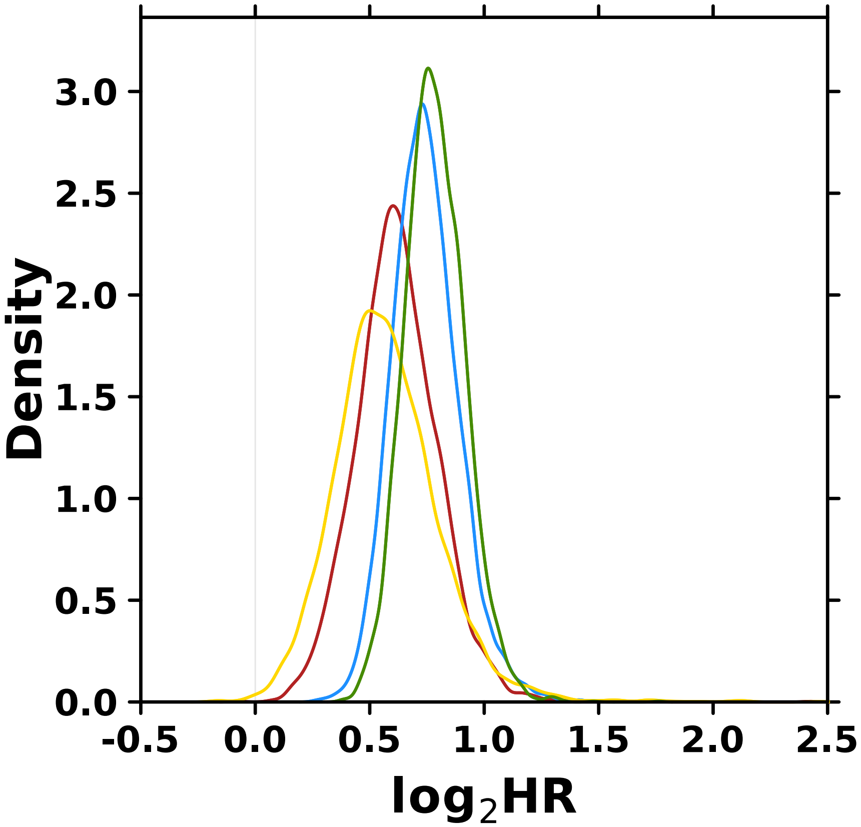

Create a zoom in of the density plot

```
create.densityplot(
  cv.results.log2.hr,
  filename = './figure5d.pdf',
  resolution=300,
  col=c('firebrick','dodgerblue','gold','chartreuse4'),
  xlab.label=expression(bold(log[2]*'HR')),
  xlimits=c(1,2.5),
  ylimits=c(0,0.2),
  xat=seq(-0.5,2.5,0.5),
  yat=seq(0,0.2,0.05),
  type=c('l','g'),
  xgrid.at=0,
  ygrid.at=0,
  right.padding=0.6
  );
```

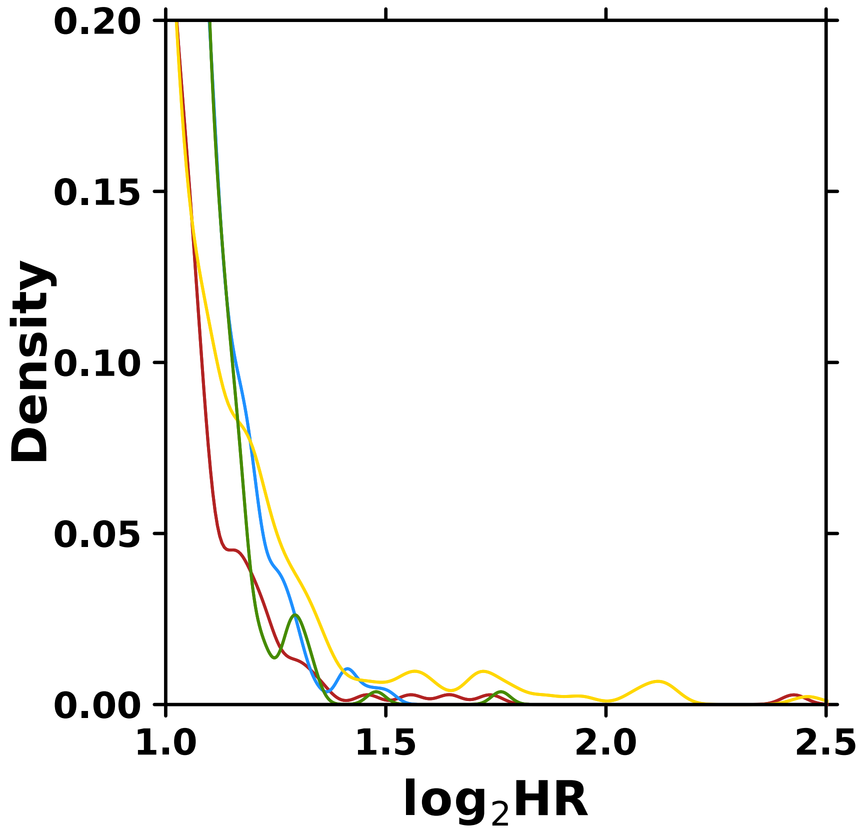

Calculate counts for HR bins

```
bin.x <- seq(1,2.25,0.5);
barplot.data <- data.frame(
  count=c(
    sapply(bin.x,function(x) {sum(cv.results.log2.hr[[1]] > x & cv.results.log2.hr[[1]] <= x+0.5)}),
    sapply(bin.x,function(x) {sum(cv.results.log2.hr[[2]] > x & cv.results.log2.hr[[2]] <= x+0.5)}),
    sapply(bin.x,function(x) {sum(cv.results.log2.hr[[3]] > x & cv.results.log2.hr[[3]] <= x+0.5)}),
    sapply(bin.x,function(x) {sum(cv.results.log2.hr[[4]] > x & cv.results.log2.hr[[4]] <= x+0.5)})
    ),
  profile.type=factor(rep(c('firebrick','dodgerblue','gold','chartreuse4'),each=length(bin.x)),levels
  bin=factor(rep(bin.x,4))
  );
barplot.data;

   count profile.type bin
1    107    firebrick   1
2      3    firebrick 1.5
3      1    firebrick   2
4    238   dodgerblue   1
5      1   dodgerblue 1.5
6      0   dodgerblue   2
7    152         gold   1
8     14         gold 1.5
9      5         gold   2
10   263  chartreuse4   1
```

```
11      1  chartreuse4 1.5
12      0  chartreuse4   2
```

29 Supplementary Figure 10 - Gene contribution to multi-gene biomarkers

Set up the environment

```
library(BoutrosLab.plotting.general);
library(VennDiagram);
library(yaml);
dataset.name <- 'Metabric';
signature.size <- 50;
```

Yaml file name

```
yaml.file <- paste0(dataset.name,'.yaml');
```

Read yaml with file information

```
dataset.files <- yaml.load_file(yaml.file);
```

Load clinical annotation

```
patient.anno <- read.table(
  dataset.files$clinical.annotation.file,
  sep='\t',
  header=TRUE
  );
```

Load mRNA abundance profiles

```
profile.types <- c('bulk','tc','tac','tc_tac');
```

Load test set survival results

```
cv.results <- list();
cv.results.auc <- list();
cv.results.log2.hr <- list();
for(profile.type in profile.types) {
  combined.result <- NULL;
  for(i in seq(1,5000,100)) {
    seed.min <- i;
    seed.max <- i+99;
    filename <- paste0(
      dataset.files$random.signatures.output.dir,
      dataset.name,
      '/',
      profile.type,
      '_test_set_results_random_signature_combined_',
```

```
      seed.min,
      '_',
      seed.max,
      '_size50.txt.gz'
      );
    if(file.exists(filename)) {
      run.result <- read.table(filename,header=TRUE,sep='\t');
      combined.result <- rbind(combined.result,run.result);
      }
    }
  combined.result <- unique(combined.result);
  cv.results[[profile.type]] <- combined.result;
  cv.results.auc[[profile.type]] <- combined.result$auc;
  cv.results.log2.hr[[profile.type]] <- -log2(combined.result$hr);
  }
signature.genes <- list();
signature.genes.top.50.auc <- list();
genes.top.signatures.50 <- list();
for(profile.type in profile.types) {
  signatures <- as.data.frame(sapply(
    as.character(cv.results[[profile.type]]$signature.probes),
    function(x) {sort(strsplit(x,':')[[1]])}
    ),stringsAsFactors=FALSE);
  rownames(signatures) <- paste0('gene',1:nrow(signatures))
  colnames(signatures) <- paste0('signature',1:ncol(signatures));
  signatures <- t(signatures);
  signature.genes[[profile.type]] <- cbind(
    cv.results[[profile.type]][,c('seed','mtry','cv.avg.auc','auc')],
    as.data.frame(signatures,stringsAsFactors=FALSE)
    );
  signature.genes.top.50.auc[[profile.type]] <- signature.genes[[profile.type]][
    rev(order(signature.genes[[profile.type]]$auc))[1:50],
    ];
  genes.top.signatures.50[[profile.type]] <- rev(sort(table(as.character(as.matrix(signature.genes.to
  }
```

Get the median AUC for the signatures that contain each gene

```
all.genes <- sort(unique(as.character(as.matrix(signature.genes$bulk[,paste0('gene',1:50)]))));
gene.auc.median <- matrix(NA,ncol=4,nrow=length(all.genes));
colnames(gene.auc.median) <- c('bulk','tc','tac','tc_tac');
rownames(gene.auc.median) <- all.genes;
gene.auc.max <- gene.auc.median;
for(i in all.genes) {
  which.signatures <- which(apply(signature.genes$bulk[,grep('gene',colnames(signature.genes$bulk))]
  for(profile.type in c('bulk','tc','tac','tc_tac')) {
    aucs <- signature.genes[[profile.type]]$auc[which.signatures];
    gene.auc.median[i,profile.type] <- median(aucs);
    gene.auc.max[i,profile.type] <- max(aucs);
    }
```

```
  }
```

Plot median AUC comparisons

```
profile.types <- c('bulk','tc','tac','tc_tac');
for(i in 1:3) {
  for(j in (i+1):4) {
    create.hexbinplot(
      y ~ x,
      data.frame(
        y=gene.auc.median[,j],
        x=gene.auc.median[,i]
        ),
      filename=paste0('sfigure10_',profile.types[i],'_',profile.types[j],'_other_legend.pdf'),
      resolution=300,
      xlimits=c(0.51,0.66),
      ylimits=c(0.51,0.66),
      xlab.label=paste0('Median AUC from \n',sub('bulk','Bulk',sub('tac','TAC',sub('tc','TC',sub('tc_
      ylab.label=paste0('Median AUC from \n',sub('bulk','Bulk',sub('tac','TAC',sub('tc','TC',sub('tc_
      add.xyline=TRUE,
      xyline.lty=2,
      aspect=1,
      legend = list(
        inside = list(
          fun = draw.key,
          args = list(
            key = get.corr.key(
              x = gene.auc.median[,i],
              y = gene.auc.median[,j],
              label.items = c('spearman','spearman.p'),
              alpha.background = 0,
              key.cex = 1.5
              )
            ),
            x = 0.45,
            y = 0.25,
            corner = c(0,1)
          )
        )
      );
    create.hexbinplot(
      y ~ x,
      data.frame(
        y=gene.auc.median[,j],
        x=gene.auc.median[,i]
        ),
      filename=paste0('sfigure10_',profile.types[i],'_',profile.types[j],'.pdf'),
      resolution=300,
      xlimits=c(0.51,0.66),
      ylimits=c(0.51,0.66),
```

```
  xlab.label=paste0('Median AUC from \n',sub('bulk','Bulk',sub('tac','TAC',sub('tc','TC',sub('tc_
  ylab.label=paste0('Median AUC from \n',sub('bulk','Bulk',sub('tac','TAC',sub('tc','TC',sub('tc_
  add.xyline=TRUE,
  xyline.lty=2,
  aspect=1,
  legend = list(
   inside = list(
    fun = draw.key,
    args = list(
     key = get.corr.key(
      x = gene.auc.median[,i],
      y = gene.auc.median[,j],
      label.items = c('spearman','spearman.p'),
      alpha.background = 0,
      key.cex = 1.5
      )
     ),
     x = 0,
     y = 0.98,
     corner = c(0,1)
    )
   )
  );
 }
}
```

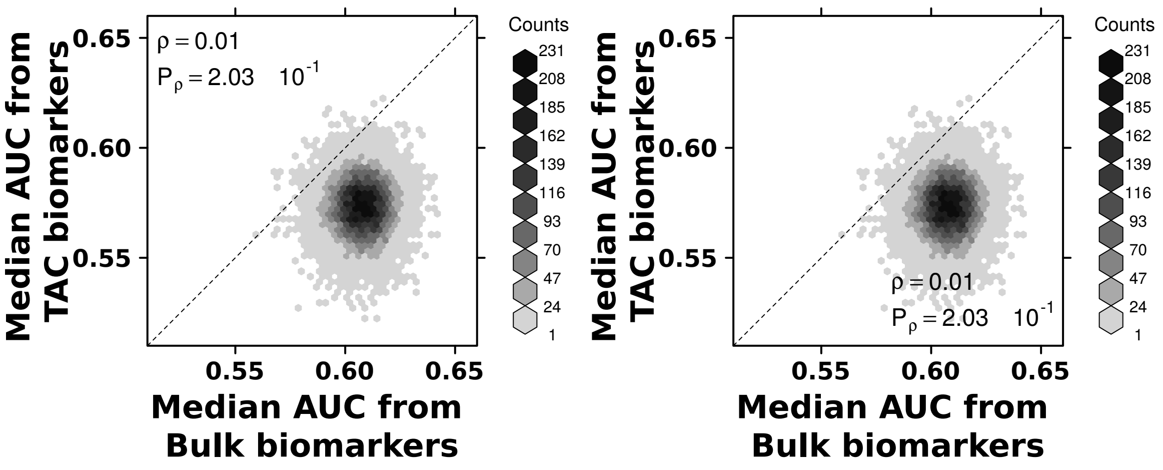

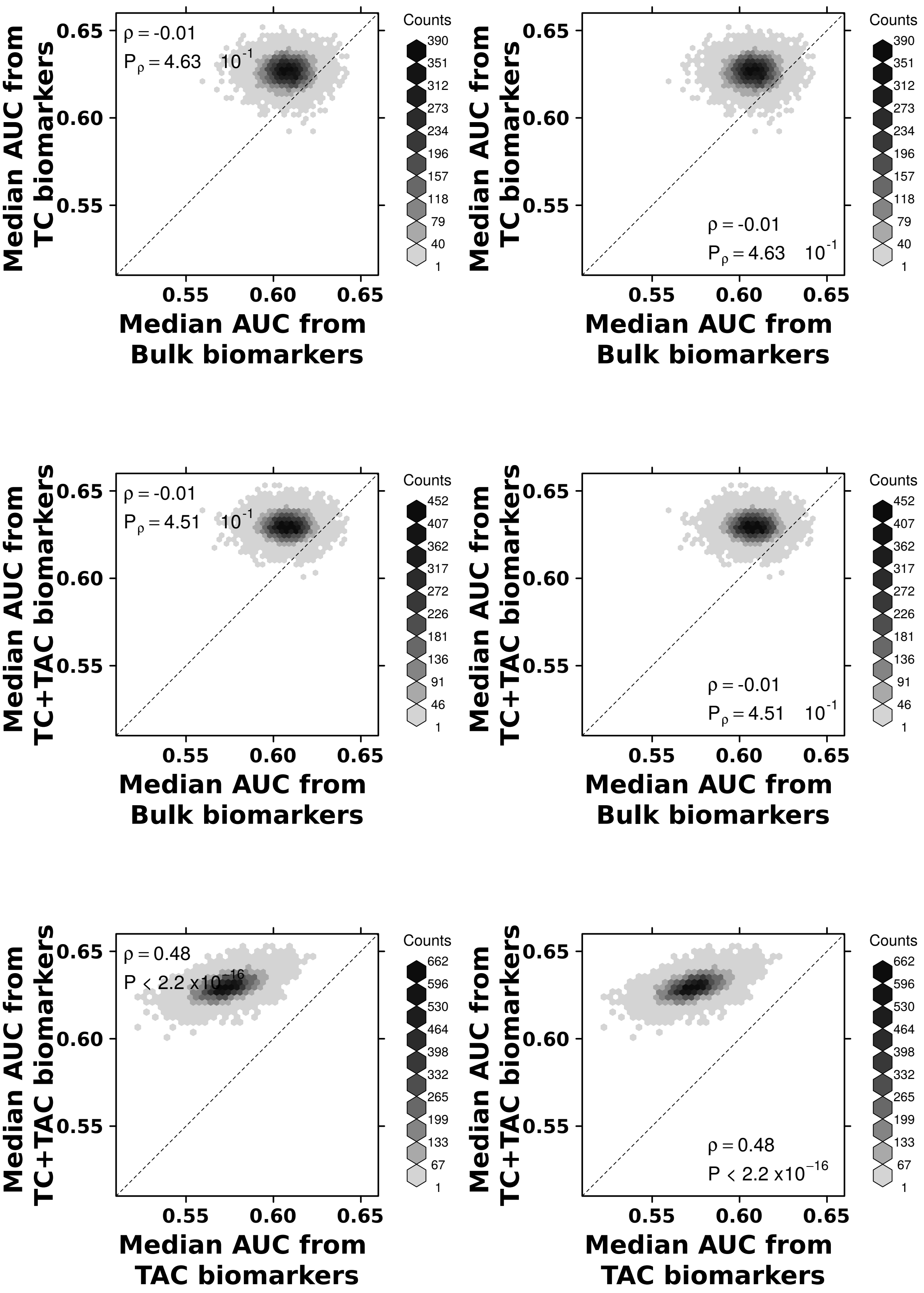
Median AUC from TC biomarkers
Median AUC from Bulk biomarkers
ρ = -0.01
P_ρ = 4.63 10^-1
Counts
390 351 312 273 234 196 157 118 79 40 1
Median AUC from TC+TAC biomarkers
Median AUC from Bulk biomarkers
ρ = -0.01
P_ρ = 4.51 10^-1
Counts
452 407 362 317 272 226 181 136 91 46 1
Median AUC from TC+TAC biomarkers
Median AUC from TAC biomarkers
ρ = 0.48
P < 2.2 x10^-16
Counts
662 596 530 464 398 332 265 199 133 67 1
0.55 0.60 0.65

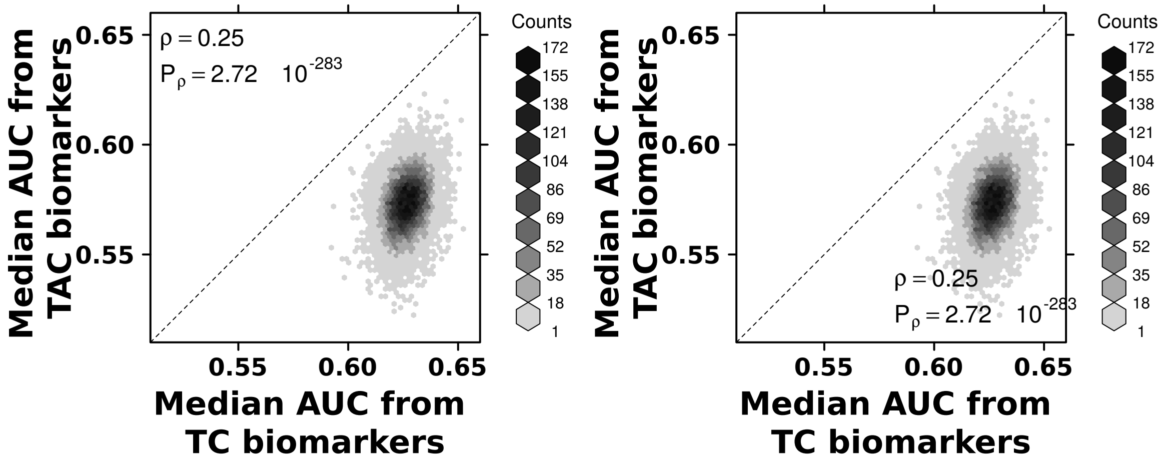

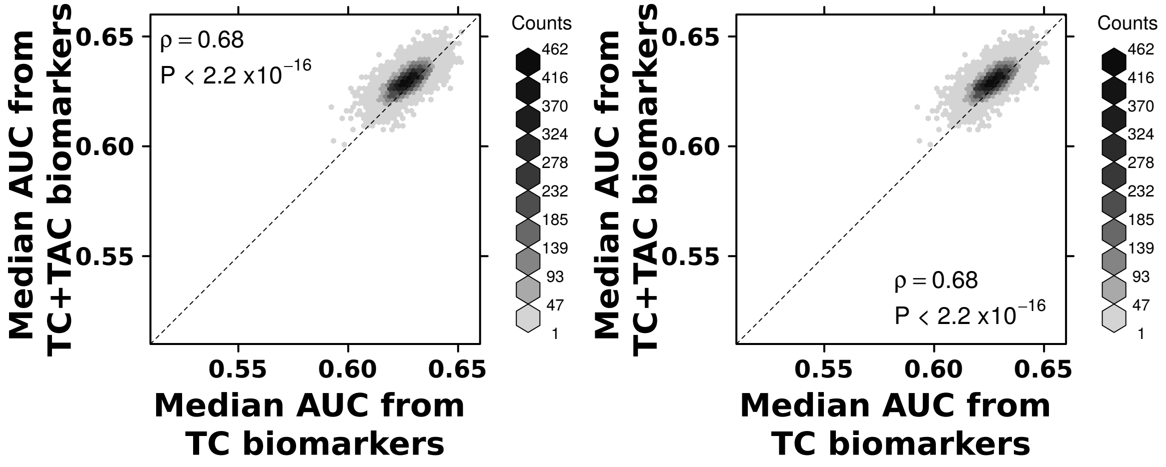

30 Figure 6 - Differential mRNA abundance associated with mutated genes

Set up the environment

```
library(BoutrosLab.plotting.general);
library(yaml);
library(limma);
dataset.name <- 'Metabric';
```

Yaml file name

```
yaml.file <- paste0(dataset.name,'.yaml');
```

Read yaml with file information

```
dataset.files <- yaml.load_file(yaml.file);
```

Load clinical annotation

```
patient.anno <- read.table(dataset.files$clinical.annotation.file,sep='\t',header=TRUE);
```

Load mRNA abundance profiles

```
profile.types <- c('bulk','tc','tac');
rna.data.matrix <- list();
for(profile.type in profile.types) {
  rna.data.matrix[[profile.type]] <- read.table(
    dataset.files[[paste0(profile.type,'.mrna.abundance.file')]],
    header=TRUE,
    sep='\t'
    );
  }
```

Intrinsic breast cancer subtype colours

```
subtype.options <- c('Basal','Her2','LumA','LumB');
subtype.col <- c('red','pink','dodgerblue3','lightskyblue2');
```

Load SNVs

```
snvs <- read.table(dataset.files$snv.file,header=TRUE);
snvs[snvs > 1] <- 1;
```

Filter to patients that have both snv and mrna data

```
overlapping.patients <- intersect(colnames(rna.data.matrix$bulk),colnames(snvs));
for(profile.type in profile.types) {
  rna.data.matrix[[profile.type]] <- rna.data.matrix[[profile.type]][,overlapping.patients];
  }
snvs <- snvs[,overlapping.patients];
patient.anno <- patient.anno[overlapping.patients,];
```

Create variables for the plots and results

```
plot.diff.counts <- list();
plot.diff.counts.s.b <- list();
plot.diff.counts.t.b <- list();
plot.mutated.count <- list();
subtype.limma.results <- list();
cis.function <- NULL;
```

The corresponding gene symbols and entrez ids which are commonly mutated and will be checked for cis function

```
sym.to.id.mapping <- data.frame(
  sym=c('TP53','MUC16','AHNAK2','SYNE1','PIK3CA','GATA3','MAP3K1','KMT2C','CDH1','AHNAK','CBFB','MLL2
  id= c('7157','94025','113146','23345','5290',  '2625', '4214',  '58508','999', '79026','865', '8085
  );
```

For each subtype, determine the number of differential mRNAs corresponding to commonly mutated genes

```
for(subtype in c('Basal','Her2','LumA','LumB')) {
  # pull out the genes that have more than 50 patients with a mutations
  genes.ordered.by.recurrence <- names(which(rowSums(snvs[
    order(rowSums(snvs)),
    rownames(patient.anno)[patient.anno$Pam50Subtype == subtype]
    ]) > 50));
  if(length(genes.ordered.by.recurrence) == 0) {
    next;
    }
  genes.ordered.by.recurrence <- genes.ordered.by.recurrence[rev(order(rowSums(snvs[
    genes.ordered.by.recurrence,
    rownames(patient.anno)[patient.anno$Pam50Subtype == subtype]
    ])))];
  # create empty matrices for the differential mRNA analysis
  tc.fold.changes <- matrix(NA, ncol=length(genes.ordered.by.recurrence),nrow=nrow(rna.data.matrix[[1
  colnames(tc.fold.changes) <- genes.ordered.by.recurrence;
  tac.fold.changes <- tc.fold.changes;
  sig.genes.data <- list();
  for(gene.snv in genes.ordered.by.recurrence) {
    # calculate differential mRNA abundance associated with the gene mutations
    targets <- rep(NA,sum(patient.anno$Pam50Subtype == subtype));
    targets[snvs[gene.snv,rownames(patient.anno)[patient.anno$Pam50Subtype == subtype]] == 1] <- 'mut
    targets[snvs[gene.snv,rownames(patient.anno)[patient.anno$Pam50Subtype == subtype]] == 0] <- 'not
    gtargets <- unique(targets);
    m <- match(targets,gtargets);
    design <- model.matrix(~ -1 + factor(m));
    colnames(design) <- gtargets;
    contrast.matrix <- makeContrasts(not-mutated, levels=design);
    limma.results <- list();
    for(profile in profile.types) {
      mrna <- rna.data.matrix[[profile]][,rownames(patient.anno)[patient.anno$Pam50Subtype == subtype
      rownames(mrna) <- 1:nrow(mrna);
      mrna <- as.matrix(mrna);
      fit <- lmFit(mrna, design);
      fit2 <- contrasts.fit(fit, contrast.matrix);
      fit3 <- eBayes(fit2);
      top1 <- topTable(fit3,coef=1,number=nrow(fit3));
      top1$GeneID <- rownames(rna.data.matrix[[profile]])[as.numeric(rownames(top1))];
      limma.results[[profile]] <- top1[order(top1$GeneID),];
      }
    sig.genes.data[[gene.snv]] <- cbind(
      limma.results$bulk[,c('GeneID','logFC','adj.P.Val')],
      limma.results$tc[,c('logFC','adj.P.Val')],
```

```
      limma.results$tac[,c('logFC','adj.P.Val')]
      )[limma.results$bulk$adj.P.Val < 0.05 | limma.results$tc$adj.P.Val < 0.05 | limma.results$tac$a
    colnames(sig.genes.data[[gene.snv]]) <- c(
      'GeneID','bulk.logFC','bulk.adj.P.Val',
      'tc.logFC','tc.adj.P.Val',
      'tac.logFC','tac.adj.P.Val'
      );
    cis.function <- rbind(cis.function,c(
      subtype,
      gene.snv,
      limma.results$bulk[
        limma.results$bulk$GeneID == paste0(sym.to.id.mapping$id[sym.to.id.mapping$sym == gene.snv],'
        c('logFC','adj.P.Val')
        ],
      limma.results$tc[
        limma.results$tc$GeneID == paste0(sym.to.id.mapping$id[sym.to.id.mapping$sym == gene.snv],'_a
        c('logFC','adj.P.Val')
        ],
      limma.results$tac[
        limma.results$tac$GeneID == paste0(sym.to.id.mapping$id[sym.to.id.mapping$sym == gene.snv],'_
        c('logFC','adj.P.Val')
        ]
      ));
    }
  subtype.limma.results[[subtype]] <- sig.genes.data;
  signif.counts <- matrix(NA,nrow=length(genes.ordered.by.recurrence),ncol=3);
  rownames(signif.counts) <- genes.ordered.by.recurrence;
  signif.counts.s.b <- signif.counts;
  signif.counts.t.b <- signif.counts;
  for(gene.snv in genes.ordered.by.recurrence) {
    signif.counts[gene.snv,1] <- sum(
      sig.genes.data[[gene.snv]]$tc.adj.P.Val < 0.05 &
      !sig.genes.data[[gene.snv]]$tac.adj.P.Val < 0.05
      );
    signif.counts[gene.snv,2] <- sum(
      sig.genes.data[[gene.snv]]$tac.adj.P.Val < 0.05 &
      !sig.genes.data[[gene.snv]]$tc.adj.P.Val < 0.05
      );
    signif.counts[gene.snv,3] <- sum(
      sig.genes.data[[gene.snv]]$tac.adj.P.Val < 0.05 &
      sig.genes.data[[gene.snv]]$tc.adj.P.Val < 0.05
      );
    signif.counts.s.b[gene.snv,1] <- sum(
      sig.genes.data[[gene.snv]]$tac.adj.P.Val < 0.05 &
      ! sig.genes.data[[gene.snv]]$bulk.adj.P.Val < 0.05
      );
    signif.counts.s.b[gene.snv,2] <- sum(
      sig.genes.data[[gene.snv]]$bulk.adj.P.Val < 0.05 &
      ! sig.genes.data[[gene.snv]]$tac.adj.P.Val < 0.05
      );
```

```
    signif.counts.s.b[gene.snv,3] <- sum(
      sig.genes.data[[gene.snv]]$tac.adj.P.Val < 0.05 &
      sig.genes.data[[gene.snv]]$bulk.adj.P.Val < 0.05
      );
    signif.counts.t.b[gene.snv,1] <- sum(
      sig.genes.data[[gene.snv]]$tc.adj.P.Val < 0.05 &
      ! sig.genes.data[[gene.snv]]$bulk.adj.P.Val < 0.05
      );
    signif.counts.t.b[gene.snv,2] <- sum(
      sig.genes.data[[gene.snv]]$bulk.adj.P.Val < 0.05 &
      ! sig.genes.data[[gene.snv]]$tc.adj.P.Val < 0.05
      );
    signif.counts.t.b[gene.snv,3] <- sum(
      sig.genes.data[[gene.snv]]$tc.adj.P.Val < 0.05 &
      sig.genes.data[[gene.snv]]$bulk.adj.P.Val < 0.05
      );
    }
  plot.diff.counts[[subtype]] <- create.barplot(
    count ~ gene,
    data.frame(
      count=as.numeric(signif.counts[,c(3,1,2)]),
      gene=rep(1:nrow(signif.counts),3)
      ),
    groups=rep(1:3,each=nrow(signif.counts)),
    resolution=300,
    xlab.label='mutated gene',
    ylab.label='number of genes with\ndifferent mRNA abundance',
    xaxis.lab=rownames(signif.counts),
    col=c('dodgerblue','gold','chartreuse4')[c(3,1,2)],
    stack=TRUE,
    xaxis.rot=90,
    width=10,
    xaxis.cex=1
    );
  plot.diff.counts.s.b[[subtype]] <- create.barplot(
    count ~ gene,
    data.frame(
      count=as.numeric(signif.counts.s.b[,c(3,1)]),
      gene=rep(1:nrow(signif.counts.s.b),2)
      ),
    groups=rep(1:2,each=nrow(signif.counts.s.b)),
    resolution=300,
    xlab.label='mutated gene',
    ylab.label='number of genes with\ndifferent mRNA abundance',
    xaxis.lab=rownames(signif.counts.s.b),
    col=c('gold','firebrick3','darkorange1')[c(3,1)],
    stack=TRUE,
    xaxis.rot=90,
    width=10,
    xaxis.cex=1
```

```
    );
  plot.diff.counts.t.b[[subtype]] <- create.barplot(
    count ~ gene,
    data.frame(
      count=as.numeric(signif.counts.t.b[,c(3,1)]),
      gene=rep(1:nrow(signif.counts.t.b),2)
      ),
    groups=rep(1:2,each=nrow(signif.counts.t.b)),
    resolution=300,
    xlab.label='mutated gene',
    ylab.label='number of genes with\ndifferent mRNA abundance',
    xaxis.lab=rownames(signif.counts.t.b),
    col=c('dodgerblue','firebrick3','orchid4')[c(3,1)],
    stack=TRUE,
    xaxis.rot=90,
    width=10,
    xaxis.cex=1
    );
  plot.mutated.count[[subtype]] <- create.barplot(
    count ~ gene,
    data.frame(
      count=as.numeric(rowSums(snvs[
        rownames(signif.counts),
        rownames(patient.anno)[patient.anno$Pam50Subtype == subtype]
        ])),
      gene=1:nrow(signif.counts)
      ),
    resolution=300,
    xlab.label='mutated gene',
    ylab.label='number of patients\nwith mutated gene',
    xaxis.lab=rownames(signif.counts),
    xaxis.rot=90,
    width=10,
    xaxis.cex=1
    );
  }
```

Output the cis mRNA abundance differences results

```
colnames(cis.function) <- c(
  'subtype','gene',
  'bulk.logFC','bulk.adj.P.Val',
  'tc.logFC','tc.adj.P.Val',
  'tac.logFC','tac.adj.P.Val'
  );
write.table(
  cis.function,
  paste0(dataset.files$parent.output.dir,'6-snvs/',dataset.name,'_mutated_gene_mRNA_cis_relationships
  col.names=TRUE,
  row.names=FALSE,
```

```
  quote=FALSE,
  sep='\t'
  );
```

Combine the panels into one plot

```
create.multiplot(
  list(
    plot.diff.counts.t.b[['Basal']],
    plot.diff.counts.t.b[['Her2']],
    plot.diff.counts.t.b[['LumA']],
    plot.diff.counts.t.b[['LumB']],
    plot.diff.counts.s.b[['Basal']],
    plot.diff.counts.s.b[['Her2']],
    plot.diff.counts.s.b[['LumA']],
    plot.diff.counts.s.b[['LumB']],
    plot.diff.counts[['Basal']],
    plot.diff.counts[['Her2']],
    plot.diff.counts[['LumA']],
    plot.diff.counts[['LumB']],
    plot.mutated.count[['Basal']],
    plot.mutated.count[['Her2']],
    plot.mutated.count[['LumA']],
    plot.mutated.count[['LumB']]
    ),
  filename = './figure6a.pdf',
  use.legacy.settings=TRUE,
  resolution=300,
  plot.layout=c(4,4),
  width=10,
  height=9,
  panel.widths=c(4,3,12,8)/(4+3+12+8),
  y.relation='free',
  x.relation='free',
  xlab.label='Mutated gene used to separate profiles for differential mRNA abundance analysis',
  ylab.label=c(
    '\n         # of patients\n         with mutated gene\n',
    '# of genes\nwith differential\nTC or TAC\nmRNA abundance\n',
    '# of genes with\ndifferential TAC\nmRNA abundance\n',
    '# of genes with         \ndifferential TC         \nmRNA abundance         \n'
    ),
  xaxis.rot=90,
  xaxis.cex=1,
  xlab.cex=1,
  ylab.cex=1,
  ylimits=list(
    c(-100,6000),c(-100,6000),c(-100,6000),c(-100,6000),
    c(-100,6000),c(-100,6000),c(-100,6000),c(-100,6000),
    c(-100,6000),c(-100,6000),c(-100,6000),c(-100,6000),
    c(0,450),c(0,450),c(0,450),c(0,450)),
```

```
  yat=list(
    seq(0,6000,1000),NULL,NULL,NULL,
    seq(0,6000,1000),NULL,NULL,NULL,
    seq(0,6000,1000),NULL,NULL,NULL,
    seq(0,400,100),NULL,NULL,NULL),
  xat=list(
    NA,NA,NA,NA,
    NULL,NULL,NULL,NULL,
    NULL,NULL,NULL,NULL,
    NULL,NULL,NULL,NULL),
  y.spacing=-0.5,
  x.spacing=-0.5,
    legend = list(
    inside = list(
      fun = draw.key,
      args = list(
        key = list(
          points = list(
            col = 'black',
            pch = 22,
            cex = 1.5,
            fill = c('gold','dodgerblue','chartreuse4')
            ),
          text = list(
            lab = c(
              'significantly different in TAC profiles',
              'significantly different in TC profiles',
              'significantly different in both TC and TAC profiles'
              )
            ),
          padding.text = 1,
          cex = 1
          )
        ),
      x = 0.3,
      y = 0.8
      ),
    inside = list(
      fun = draw.key,
      args = list(
        key = list(
          points = list(
            col = 'black',
            pch = 22,
            cex = 1.5,
            fill = c('gold','darkorange1')
            ),
          text = list(
            lab = c(
              'significantly different in TAC profiles',
```

```
            'significantly different in TAC and bulk profiles'
            )
          ),
        padding.text = 1,
        cex = 1
        )
      ),
    x = 0.3,
    y = 0.52
    ),
  inside = list(
    fun = draw.key,
    args = list(
      key = list(
        points = list(
          col = 'black',
          pch = 22,
          cex = 1.5,
          fill = c('dodgerblue','orchid4')
          ),
        text = list(
          lab = c(
            'significantly different in TC profiles',
            'significantly different in TC and bulk profiles'
            )
          ),
        padding.text = 1,
        cex = 1
        )
      ),
    x = 0.3,
    y = 0.24
    )
  ),
print.new.legend=TRUE
);
```

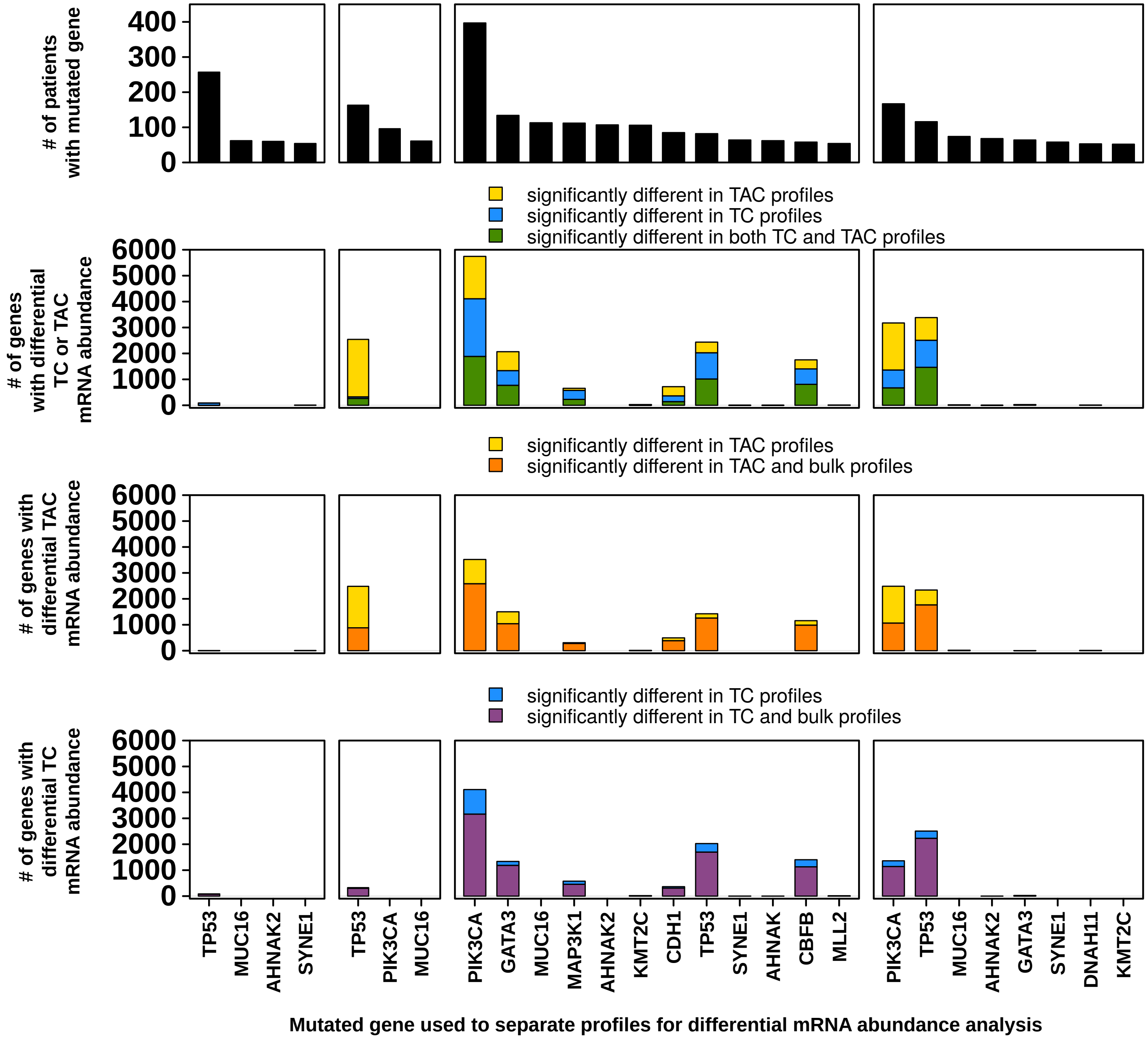

Load the annotation linking entrez IDs and gene symbols

```
gene.sym <- read.table(dataset.files$gene.symbol.file,header=TRUE,sep='\t');
```

Create a scatterplot comparing TC and TAC results for TP53 mutations in Her2-enriched breast cancer

```
gene.snv <- 'TP53';
subtype <- 'Her2';
points.sig <- as.numeric(subtype.limma.results[[subtype]][[gene.snv]]$tc.adj.P.Val < 0.05) +
  2*as.numeric(subtype.limma.results[[subtype]][[gene.snv]]$tac.adj.P.Val < 0.05);
her2.tp53.fc <- subtype.limma.results[[subtype]][[gene.snv]][points.sig != 0,];
her2.tp53.fc <- her2.tp53.fc[order(her2.tp53.fc$GeneID),];
genes.to.label <- sort(c('3543','6280','3854','5284','6259','290','8614','2099','133','5179','10140',
points.labels <- data.frame(
```

```
  id=as.character(genes.to.label),
  label=as.character(gene.sym$GeneSymbol[match(genes.to.label,gene.sym$EntrezID)]),
  tc_logfc=her2.tp53.fc$tc.logFC[her2.tp53.fc$GeneID %in% paste0(genes.to.label,'_at')],
  tac_logfc=her2.tp53.fc$tac.logFC[her2.tp53.fc$GeneID %in% paste0(genes.to.label,'_at')],
  y_offset=rep(0,length(genes.to.label)),
  x_offset=rep(0,length(genes.to.label)),
  stringsAsFactors=FALSE
  );
points.labels$label[points.labels$id == '290'] <- 'ANPEP';
points.labels[points.labels$label == 'ANPEP',c('x_offset','y_offset')] <- c(-0.55,0.02);
points.labels[points.labels$label == 'STC2','y_offset'] <- 0.027;
points.labels[points.labels$label == 'RYK','y_offset'] <- -0.027;
points.labels[points.labels$label == 'PIGR',c('x_offset','y_offset')] <- c(0.2,-0.027);
points.labels[points.labels$label == 'IGLL1','x_offset'] <- -0.5;
points.labels[points.labels$label == 'KRT6B','x_offset'] <- -0.65;
points.labels[points.labels$label == 'PHB','x_offset'] <- 0.4;
points.labels[points.labels$label == 'TOB1','x_offset'] <- 0.5;
points.labels[points.labels$label == 'NME1','x_offset'] <- 0.5;
points.labels[points.labels$label == 'PENK','x_offset'] <- -0.5;
points.labels[points.labels$label == 'S100A9',c('x_offset','y_offset')] <- c(-0.4,-0.027);
points.labels[points.labels$label == 'AKR7A3',c('x_offset','y_offset')] <- c(0.4,0.027);
create.scatterplot(
  tc_fc ~ tac_fc,
  data.frame(
    tc_fc = subtype.limma.results[[subtype]][[gene.snv]]$tc.logFC[points.sig != 0],
    tac_fc = subtype.limma.results[[subtype]][[gene.snv]]$tac.logFC[points.sig != 0]
    ),
  filename = './figure6b.pdf',
  resolution=300,
  abline.h=0,
  abline.v=0,
  abline.lty=2,
  col=c('dodgerblue','gold','chartreuse4')[points.sig[points.sig != 0]],
  alpha=0.5,
  xlab.label=expression('TAC log'[2]*' Fold Change'),
  ylab.label=expression('TC log'[2]*' Fold Change'),
  main=paste0(gene.snv,' mutations in ',sub('Lum','Luminal ',sub('Her2','HER2-enriched',subtype)),' p
  main.cex=1.75,
  add.text=TRUE,
  text.labels=points.labels$label,
  text.x=points.labels$tac_logfc+points.labels$x_offset,
  text.y=points.labels$tc_logfc+points.labels$y_offset,
  text.cex=0.8,
  use.legacy.settings=TRUE
  );
```

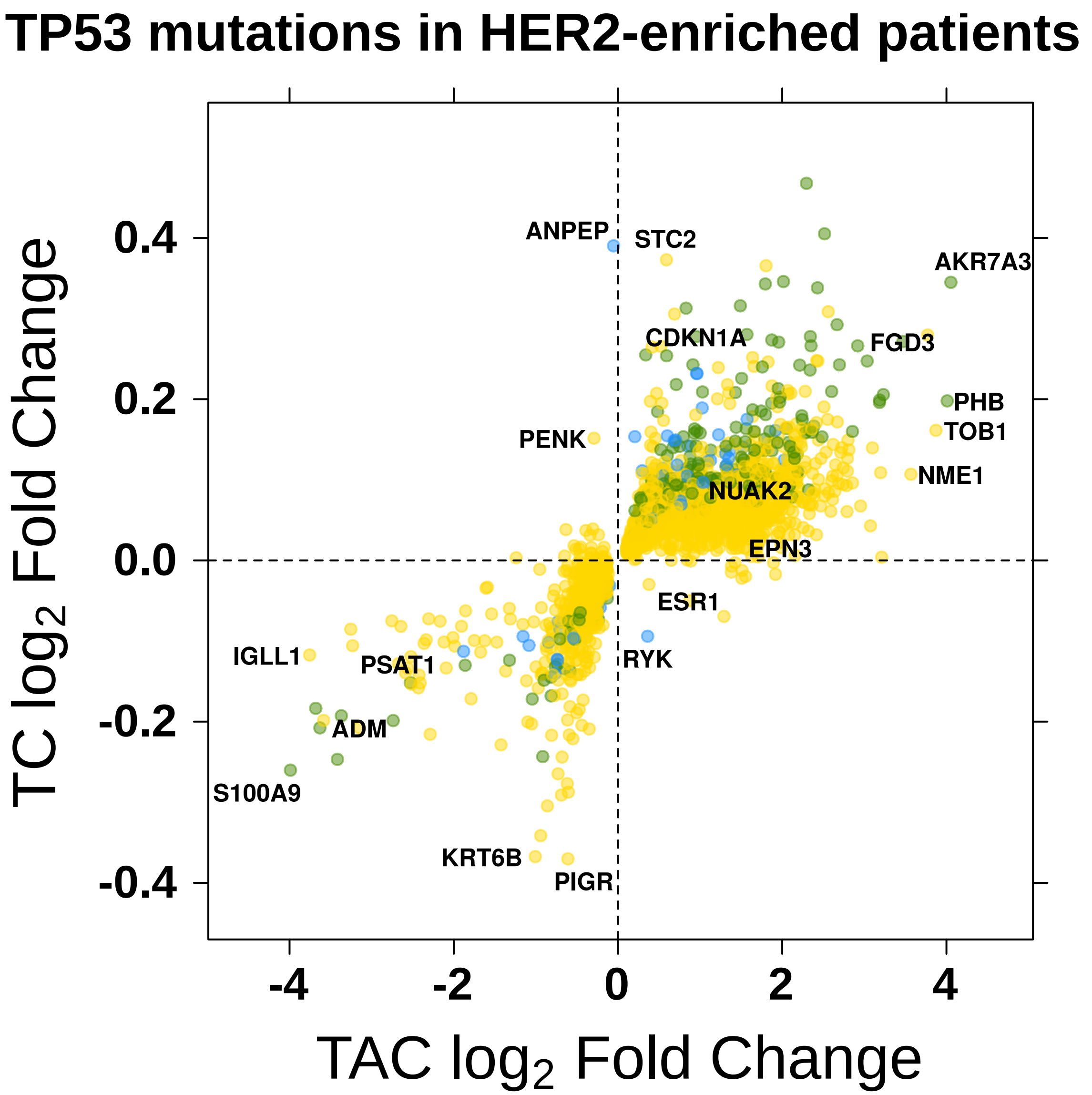

Create a scatterplot comparing TC and TAC results for CDH1 mutations in Luminal A breast cancer

```
gene.snv <- 'CDH1';
subtype <- 'LumA';
points.sig <- as.numeric(subtype.limma.results[[subtype]][[gene.snv]]$tc.adj.P.Val < 0.05) +
  2*as.numeric(subtype.limma.results[[subtype]][[gene.snv]]$tac.adj.P.Val < 0.05);
cdh1.luma.fc <- subtype.limma.results[[subtype]][[gene.snv]][points.sig != 0,];
cdh1.luma.fc <- cdh1.luma.fc[order(cdh1.luma.fc$GeneID),];
genes.to.label <- sort(c('999','11012','1893','1917','2891','3158','3918','5265','65266'));
points.labels <- data.frame(
  id=as.character(genes.to.label),
  label=as.character(gene.sym$GeneSymbol[match(genes.to.label,gene.sym$EntrezID)]),
  tc_logfc=cdh1.luma.fc$tc.logFC[cdh1.luma.fc$GeneID %in% paste0(genes.to.label,'_at')],
  tac_logfc=cdh1.luma.fc$tac.logFC[cdh1.luma.fc$GeneID %in% paste0(genes.to.label,'_at')],
  y_offset=rep(0,length(genes.to.label)),
  x_offset=rep(0,length(genes.to.label)),
  stringsAsFactors=FALSE
  );
```

```r
points.labels[points.labels$label == 'KLK11',c('x_offset','y_offset')] <- c(0.8,-0.01);
points.labels[points.labels$label == 'CDH1','y_offset'] <- 0.015;
points.labels[points.labels$label == 'GRIA2','y_offset'] <- 0.015;
points.labels[points.labels$label == 'LAMC2','y_offset'] <- 0.015;
points.labels[points.labels$label == 'ECM1','y_offset'] <- 0.015;
points.labels[points.labels$label == 'EEF1A2','x_offset'] <- 1.07;
points.labels[points.labels$label == 'SERPINA1','x_offset'] <- 1.4;
points.labels[points.labels$label == 'WNK4','x_offset'] <- -1;
points.labels[points.labels$label == 'HMGCS2','x_offset'] <- -1.2;
create.scatterplot(
  tc_fc ~ tac_fc,
  data.frame(
    tc_fc = subtype.limma.results[[subtype]][[gene.snv]]$tc.logFC[points.sig != 0],
    tac_fc = subtype.limma.results[[subtype]][[gene.snv]]$tac.logFC[points.sig != 0]
    ),
  filename = './figure6c.pdf',
  resolution=300,
  abline.h=0,
  abline.v=0,
  abline.lty=2,
  col=c('dodgerblue','gold','chartreuse4')[points.sig[points.sig != 0]],
  alpha=0.5,
  xlab.label=expression('TAC log'[2]*' Fold Change'),
  ylab.label=expression('TC log'[2]*' Fold Change'),
  main=paste0(gene.snv,' mutations in ',sub('Lum','Luminal ',sub('Her2','HER2-enriched',subtype)),' p
  main.cex=1.75,
  add.text=TRUE,
  text.labels=points.labels$label,
  text.x=points.labels$tac_logfc+points.labels$x_offset,
  text.y=points.labels$tc_logfc+points.labels$y_offset,
  text.cex=0.8,
  use.legacy.settings=TRUE
  );
```

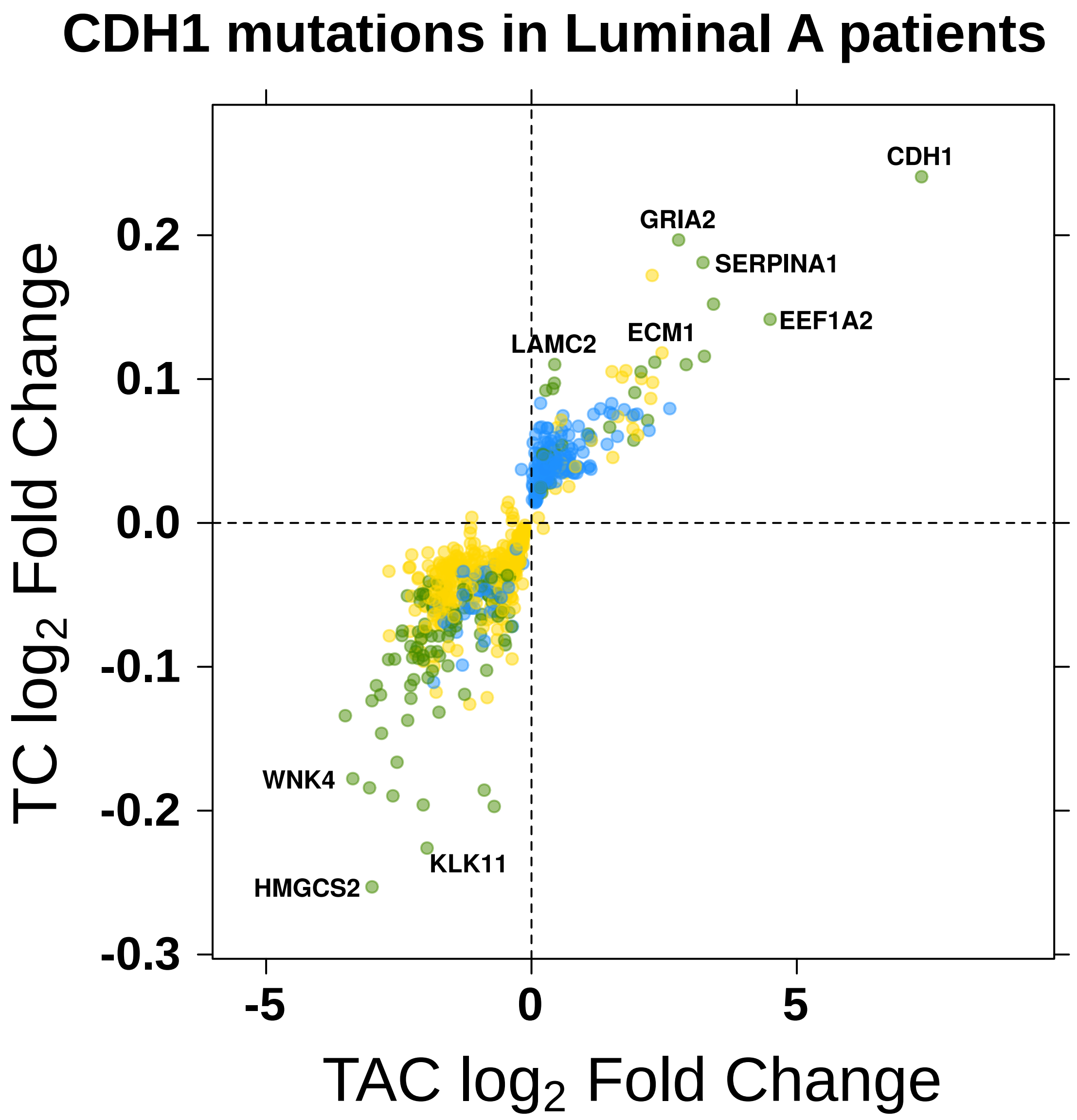

31 Supplementary Figure 11 - Differential mRNA abundance validation

Set up the environment

```
library(BoutrosLab.plotting.general);
library(yaml);
library(limma);
dataset.names <- c('Metabric','BRCA');
profile.types <- c('bulk','tc','tac');
dataset.files <- list();
patient.anno <- list();
rna.data.matrix <- list();
snvs <- list();
```

Load the data corresponding to each dataset

```
for(dataset.name in dataset.names) {
  # read yaml with file information
  dataset.files[[dataset.name]] <- yaml.load_file(paste0(dataset.name,'.yaml'));
  # load clinical annotation
  patient.anno[[dataset.name]] <- read.table(
    dataset.files[[dataset.name]]$clinical.annotation.file,
    sep='\t',
    header=TRUE
    );
  # remove Normal-like patients
  patient.anno[[dataset.name]] <- patient.anno[[dataset.name]][
    which(!patient.anno[[dataset.name]]$subtype %in% c('Normal-like','unknown')),
    ];
  # load mRNA abundance results
  rna.data.matrix[[dataset.name]] <- list();
  for(profile.type in profile.types) {
    rna.data.matrix[[dataset.name]][[profile.type]] <- read.table(
      dataset.files[[dataset.name]][[paste0(profile.type,'.mrna.abundance.file')]],
      header=TRUE,
      sep='\t'
      );
    rna.data.matrix[[dataset.name]][[profile.type]] <- rna.data.matrix[[dataset.name]][[profile.type]
      intersect(
        colnames(rna.data.matrix[[dataset.name]][[profile.type]]),
        patient.anno[[dataset.name]]$patient_id
        )
      ];
    }
  # load SNVs
  snvs[[dataset.name]] <- read.table(dataset.files[[dataset.name]]$snv.file,header=TRUE);
  snvs[[dataset.name]][snvs[[dataset.name]] > 1] <- 1;
  }
```

The entrez ids for the mutated genes beign assessed

```
sym.to.id.mapping <- data.frame(
  sym=c('TP53','MUC16','AHNAK2','SYNE1','PIK3CA','GATA3','MAP3K1','KMT2C','CDH1','AHNAK','CBFB','MLL2
  id= c('7157','94025','113146','23345','5290','2625','4214','58508','999','79026','865','8085','8701
  );
```

Run the differential mRNA abundance analysis for each dataset

```
subtype.limma.results <- list();
common.genes <- intersect(
  rownames(rna.data.matrix$Metabric[[profile.type]]),
  rownames(rna.data.matrix$BRCA[[profile.type]])
  );
for(dataset.name in dataset.names) {
  # check patient ordering matches for all the data
  overlapping.patients <- intersect(
```

```
    intersect(
      colnames(rna.data.matrix[[dataset.name]]$bulk),
      colnames(snvs[[dataset.name]])
      ),
    patient.anno[[dataset.name]]$patient_id
    );
  for(profile.type in profile.types) {
    rna.data.matrix[[dataset.name]][[profile.type]] <- rna.data.matrix[[dataset.name]][[profile.type]
    }
  snvs[[dataset.name]] <- snvs[[dataset.name]][,overlapping.patients];
  patient.anno[[dataset.name]] <- patient.anno[[dataset.name]][overlapping.patients,];
  # run analysis for each subtype
  subtype.limma.results[[dataset.name]] <- list();
  for(subtype in c('Basal','Her2','LumA','LumB')) {
    # select the genes with more than 50 patients with a mutation
    genes.ordered.by.recurrence <- names(which(rowSums(snvs[[dataset.name]][
      order(rowSums(snvs[[dataset.name]])),
      rownames(patient.anno[[dataset.name]])[patient.anno[[dataset.name]]$Pam50Subtype == subtype]
      ]) > 50));
    if(length(genes.ordered.by.recurrence) == 0) {
      next;
      }
    genes.ordered.by.recurrence <- genes.ordered.by.recurrence[rev(order(rowSums(snvs[[dataset.name]]
      genes.ordered.by.recurrence,
      rownames(patient.anno[[dataset.name]])[patient.anno[[dataset.name]]$Pam50Subtype == subtype]
      ])))];
    # evaluate differential mRNA abundance for each frequently mutated gene
    subtype.limma.results[[dataset.name]][[subtype]] <- list();
    for(gene.snv in genes.ordered.by.recurrence) {
      # assign patients into groups based on whether they have the gene mutated
      targets <- rep(NA,sum(patient.anno$Pam50Subtype == subtype));
      targets[snvs[[dataset.name]][
        gene.snv,rownames(patient.anno[[dataset.name]])[patient.anno[[dataset.name]]$Pam50Subtype ==
        ] == 1] <- 'mutated';
      targets[snvs[[dataset.name]][
        gene.snv,rownames(patient.anno[[dataset.name]])[patient.anno[[dataset.name]]$Pam50Subtype ==
        ] == 0] <- 'not';
      gtargets <- unique(targets);
      m <- match(targets,gtargets);
      design <- model.matrix(~ -1 + factor(m));
      colnames(design) <- gtargets;
      contrast.matrix <- makeContrasts(not-mutated, levels=design);
      limma.results <- list();
      for(profile in profile.types) {
        # load the mRNA abundance to test for differential mRNA abundance using limma
        mrna <- rna.data.matrix[[dataset.name]][[profile]][,
          rownames(patient.anno[[dataset.name]])[patient.anno[[dataset.name]]$Pam50Subtype == subtype
          ];
        rownames(mrna) <- 1:nrow(mrna);
        mrna <- as.matrix(mrna);
```

```
        # run the limma model
        fit <- lmFit(mrna, design);
        fit2 <- contrasts.fit(fit, contrast.matrix);
        fit3 <- eBayes(fit2);
        top1 <- topTable(fit3,coef=1,number=nrow(fit3));
        top1$GeneID <- rownames(rna.data.matrix[[dataset.name]][[profile]])[as.numeric(rownames(top1)
        limma.results[[profile]] <- top1[order(top1$GeneID),];
        rownames(limma.results[[profile]]) <- limma.results[[profile]]$GeneID;
        }
      # save the results
      subtype.limma.results[[dataset.name]][[subtype]][[gene.snv]] <- cbind(
        limma.results$bulk[sort(rownames(limma.results$bulk)),c('GeneID','logFC','P.Value','adj.P.Va
        limma.results$tc[sort(rownames(limma.results$bulk)),c('logFC','P.Value','adj.P.Val')],
        limma.results$tac[sort(rownames(limma.results$bulk)),c('logFC','P.Value','adj.P.Val')]
        );
      colnames(subtype.limma.results[[dataset.name]][[subtype]][[gene.snv]]) <- c(
        'GeneID','bulk.logFC','bulk.P.Value','bulk.adj.P.Val',
        'tc.logFC','tc.P.Value','tc.adj.P.Val',
        'tac.logFC','tac.P.Value','tac.adj.P.Val'
        );
      rownames(subtype.limma.results[[dataset.name]][[subtype]][[gene.snv]]) <-
        subtype.limma.results[[dataset.name]][[subtype]][[gene.snv]]$GeneID;
      }
    }
  }
```

Create the comparison plot for each case (subtype - gene combo)

```
for(subtype in names(subtype.limma.results$BRCA)) {
  for(gene.snv in names(subtype.limma.results$BRCA[[subtype]])) {
    for(profile.type in profile.types) {
      plot.data <- data.frame(
        metabric=subtype.limma.results$Metabric[[subtype]][[gene.snv]][,paste0(profile.type,'.logFC')
        tcga=subtype.limma.results$BRCA[[subtype]][[gene.snv]][,paste0(profile.type,'.logFC')]
        );
      plot.min <- floor(min(c(plot.data$metabric,plot.data$tcga)))-0.1;
      plot.max <- ceiling(max(c(plot.data$metabric,plot.data$tcga)))+0.1;
      create.scatterplot(
          metabric ~ tcga,
          plot.data,
          resolution=200,
          filename=paste0('./sfigure11_',subtype,'_',gene.snv,'_',profile.type,'.pdf'),
          add.xyline=TRUE,
          xyline.lty=2,
          xlab.label=expression('TCGA log'['2']*'Fold Change'),
          ylab.label=expression('METABRIC log'['2']*'Fold Change'),
          xlimits=c(plot.min, plot.max),
          ylimits=c(plot.min, plot.max),
          xlab.cex = 2,
          ylab.cex = 2,
```

```
          legend = list(
            inside = list(
            fun = draw.key,
            args = list(
              key = get.corr.key(
                x = plot.data$tcga,
                y = plot.data$metabric,
                label.items = c('spearman','spearman.p'),
                alpha.background = 0,
                key.cex = 1.5
                )
              ),
            x = 0.95,
            y = 0.01,
            corner = c(1,0)
            )
          )
        );
      }
    }
  }
```

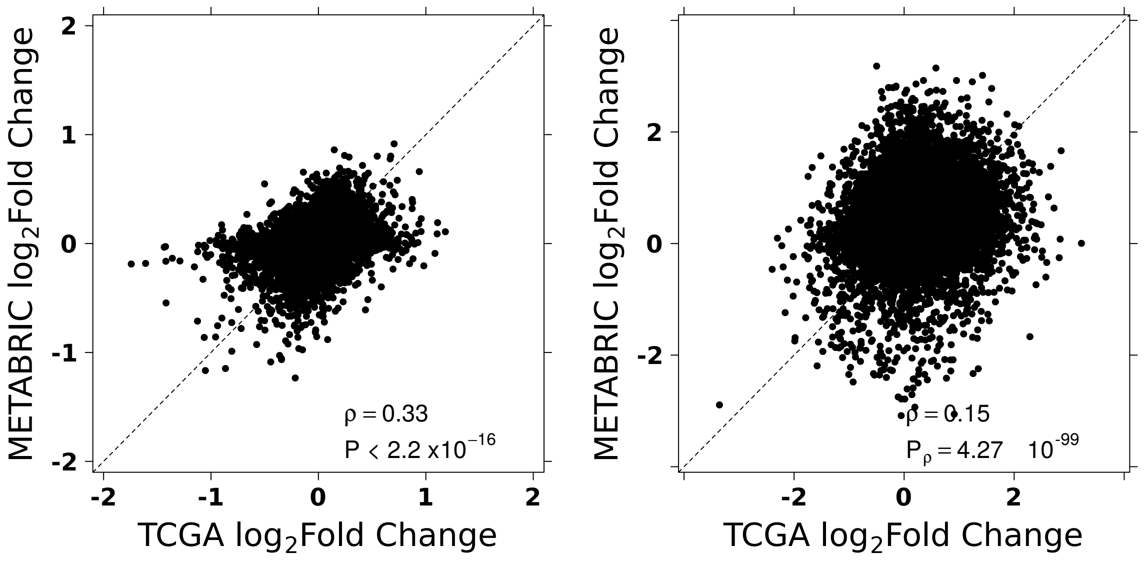

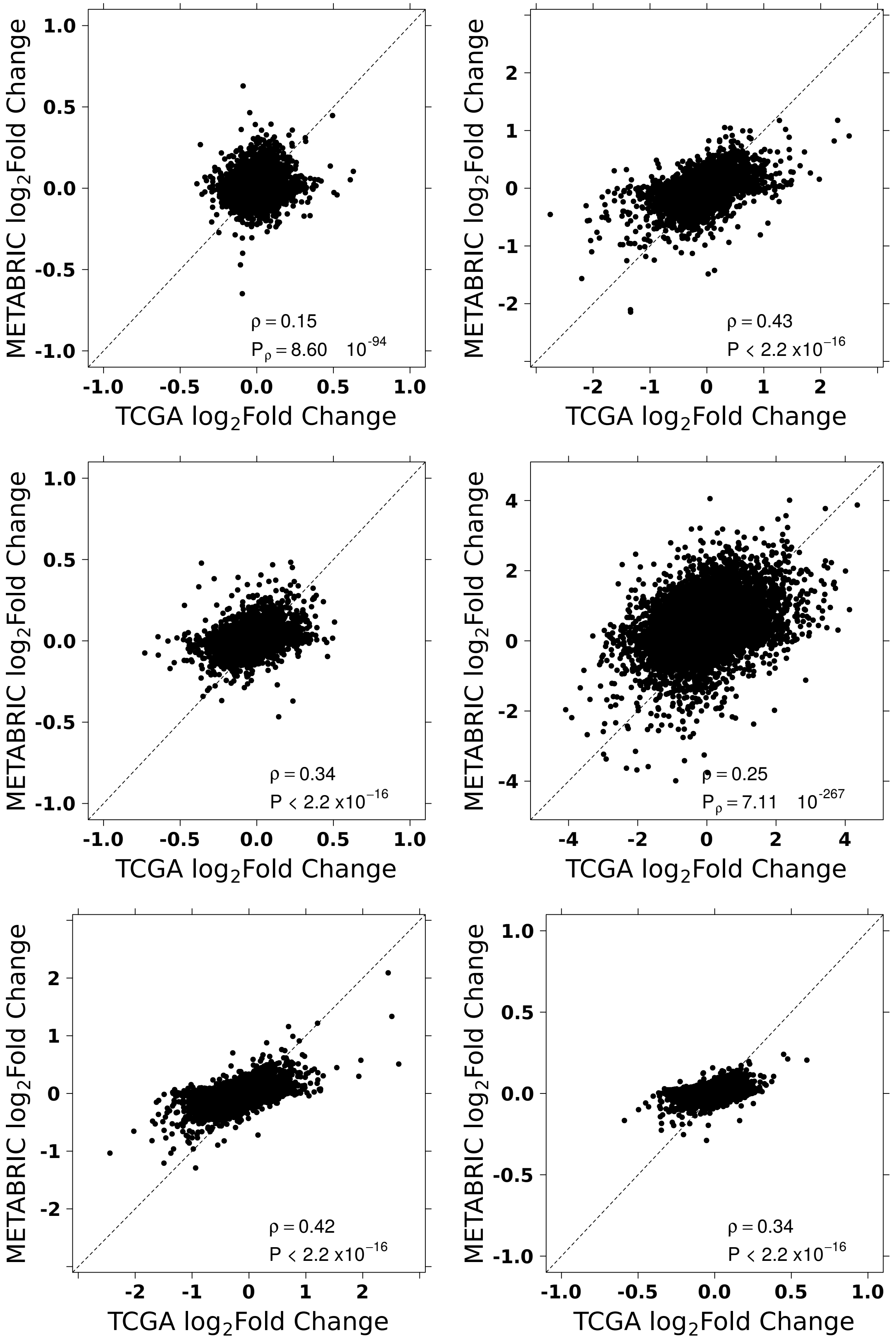
METABRIC log2Fold Change
TCGA log2Fold Change
ρ = 0.15
Pρ = 8.60 10-94
ρ = 0.43
P < 2.2 x10−16
ρ = 0.34
P < 2.2 x10−16
ρ = 0.25
Pρ = 7.11 10-267
ρ = 0.42
P < 2.2 x10−16
ρ = 0.34
P < 2.2 x10−16

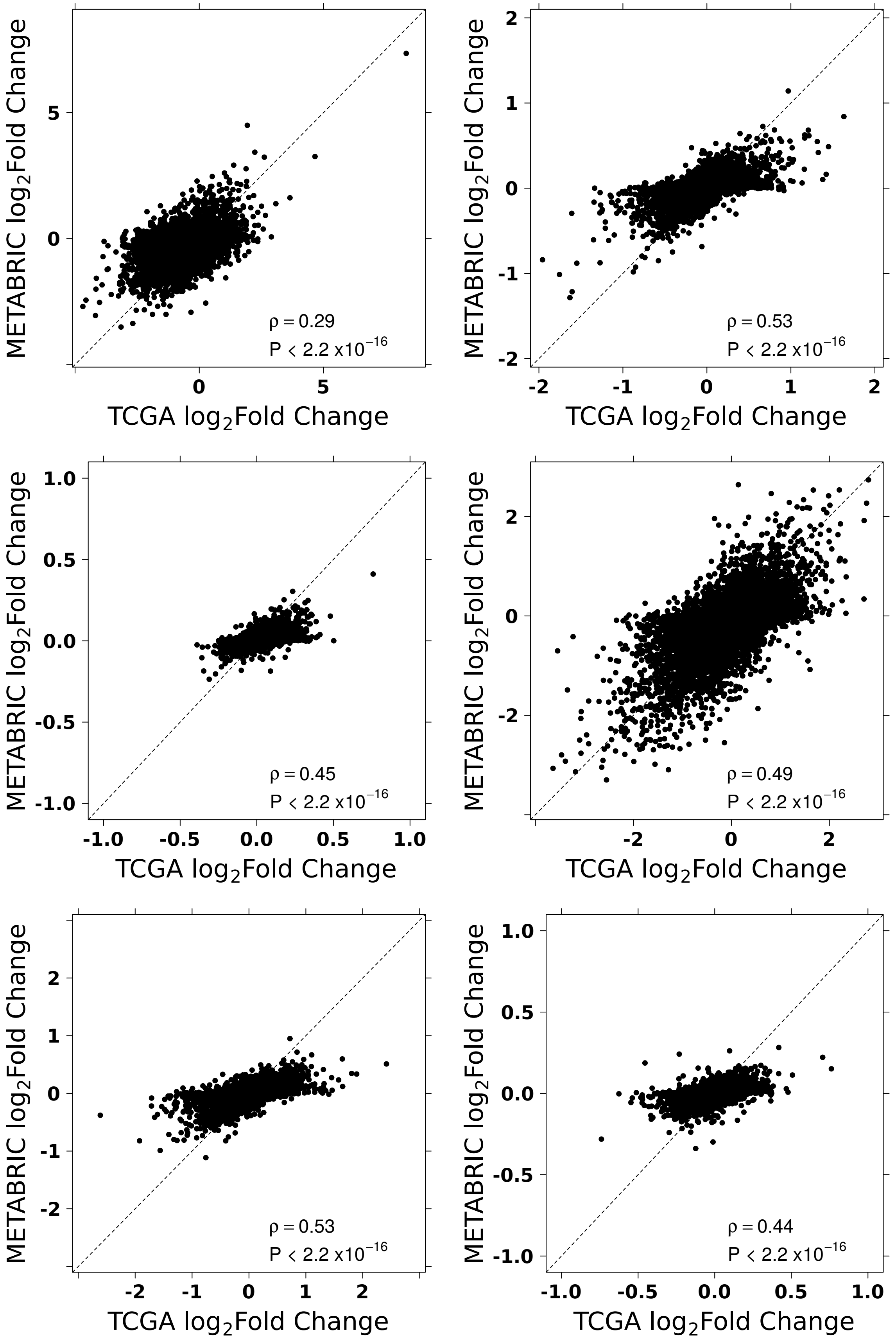
METABRIC $\log_2$Fold Change
TCGA $\log_2$Fold Change
$\rho = 0.29$
$P < 2.2 \times 10^{-16}$
$\rho = 0.53$
$P < 2.2 \times 10^{-16}$
$\rho = 0.45$
$P < 2.2 \times 10^{-16}$
$\rho = 0.49$
$P < 2.2 \times 10^{-16}$
$\rho = 0.53$
$P < 2.2 \times 10^{-16}$
$\rho = 0.44$
$P < 2.2 \times 10^{-16}$

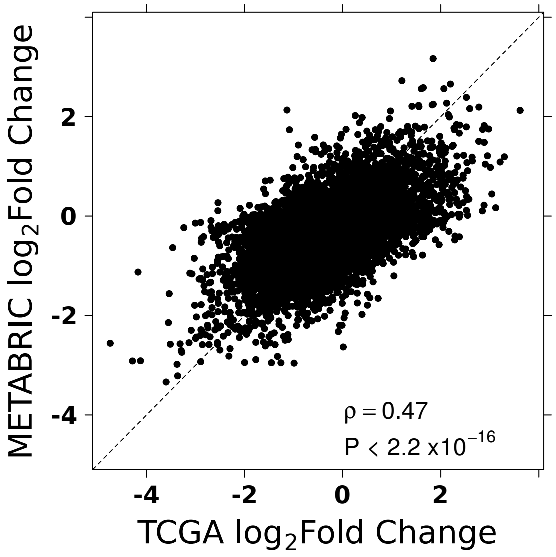

32 Supplementary Figure 12A,B,C,E,F - mRNA abundance differences associated with *TP53* and *CDH1*

Set up the environment

```
library(BoutrosLab.plotting.general);
library(yaml);
library(limma);
dataset.name <- 'Metabric';
```

Yaml file name

```
yaml.file <- paste0(dataset.name,'.yaml');
```

Read yaml with file information

```
dataset.files <- yaml.load_file(yaml.file);
```

Load clinical annotation

```
patient.anno <- read.table(dataset.files$clinical.annotation.file,sep='\t',header=TRUE);
```

Load mRNA abundance profiles

```
profile.types <- c('bulk','tc','tac');
rna.data.matrix <- list();
for(profile.type in profile.types) {
  rna.data.matrix[[profile.type]] <- read.table(
    dataset.files[[paste0(profile.type,'.mrna.abundance.file')]],
    header=TRUE,
    sep='\t'
    );
  }
```

Load SNVs

```
snvs <- read.table(dataset.files$snv.file,header=TRUE);
```

Make sure all the data is ordered the same

```
overlapping.patients <- intersect(colnames(rna.data.matrix[[1]]),colnames(snvs));
for(profile.type in profile.types) {
  rna.data.matrix[[profile.type]] <- rna.data.matrix[[profile.type]][,overlapping.patients];
  }
snvs <- snvs[,overlapping.patients];
patient.anno <- patient.anno[overlapping.patients,];
```

Specify the subtype to plot for each mutated gene

```
gene.subtype <- list(
  'TP53'='Her2',
  'CDH1'='LumA'
  );
```

Specify the mRNA abundances to plot for each mutated gene

```
gene.mrna.entrez.id <- list(
  'TP53'=c('2099_at','5245_at','7157_at'),
  'CDH1'=c('999_at','8073_at')
  );
```

For each gene split the patients into mutated and not and the create a boxplot showing mRNA abundance differences

```
for(gene.snv in c('TP53','CDH1')) {
  subtype <- gene.subtype[[gene.snv]];
  for(expression.entrez.id in gene.mrna.entrez.id[[gene.snv]]) {
    mutated.samples <- rownames(patient.anno)[patient.anno$Pam50Subtype == subtype][
      snvs[gene.snv,rownames(patient.anno)[patient.anno$Pam50Subtype == subtype]] == 1
      ];
    not.mutated.samples <- rownames(patient.anno)[patient.anno$Pam50Subtype == subtype][
      snvs[gene.snv,rownames(patient.anno)[patient.anno$Pam50Subtype == subtype]] == 0
      ];
    for(profile.type in profile.types) {
      # create boxplot
      create.boxplot(
        y ~ x,
        data.frame(
          x=factor(c(
            rep(3,length(mutated.samples)),
            rep(4,length(not.mutated.samples))
            )),
          y=c(
            as.numeric(rna.data.matrix[[profile.type]][expression.entrez.id,mutated.samples]),
```

```
          as.numeric(rna.data.matrix[[profile.type]][expression.entrez.id,not.mutated.samples])
          )
        ),
      filename = paste0('./sfigure12abcef_',gene.snv,'_mrna_',expression.entrez.id,'_',profile.type
      resolution=300,
      add.stripplot=TRUE,
      points.cex=0.2,
      xlab.label=NULL,
      ylab.label=paste0(sub('tc','TC',sub('tac','TAC',profile.type)),' mRNA abundance'),
      xaxis.lab=c(
        paste0('mutated ',gene.snv),
        'not mutated'
        ),
      xaxis.rot=90,
      width=3,
      use.legacy.settings=TRUE
      );
    }
  }
}
```

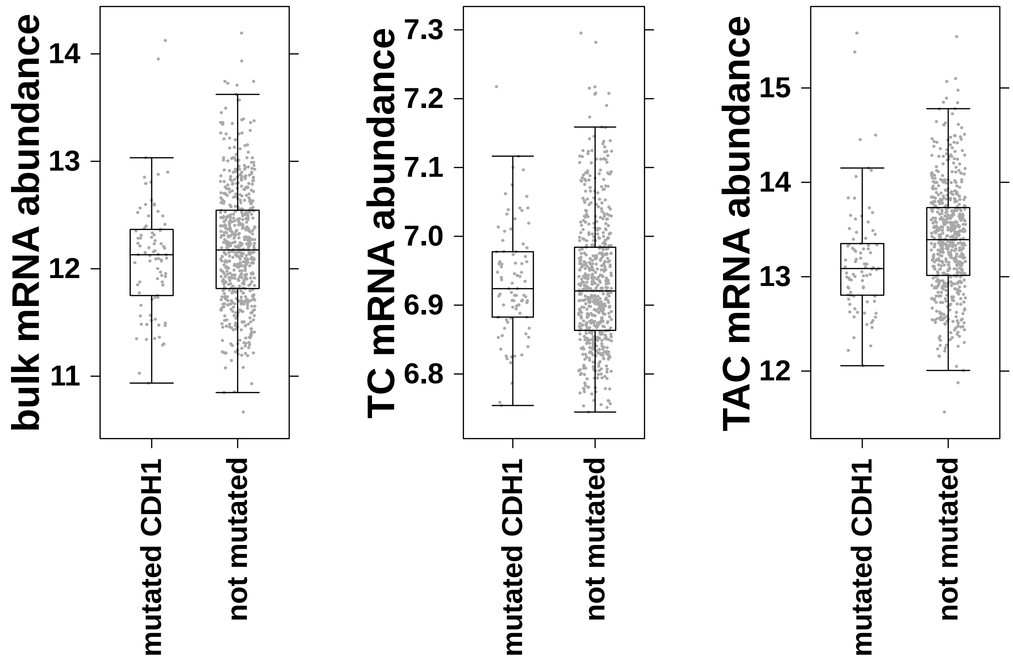

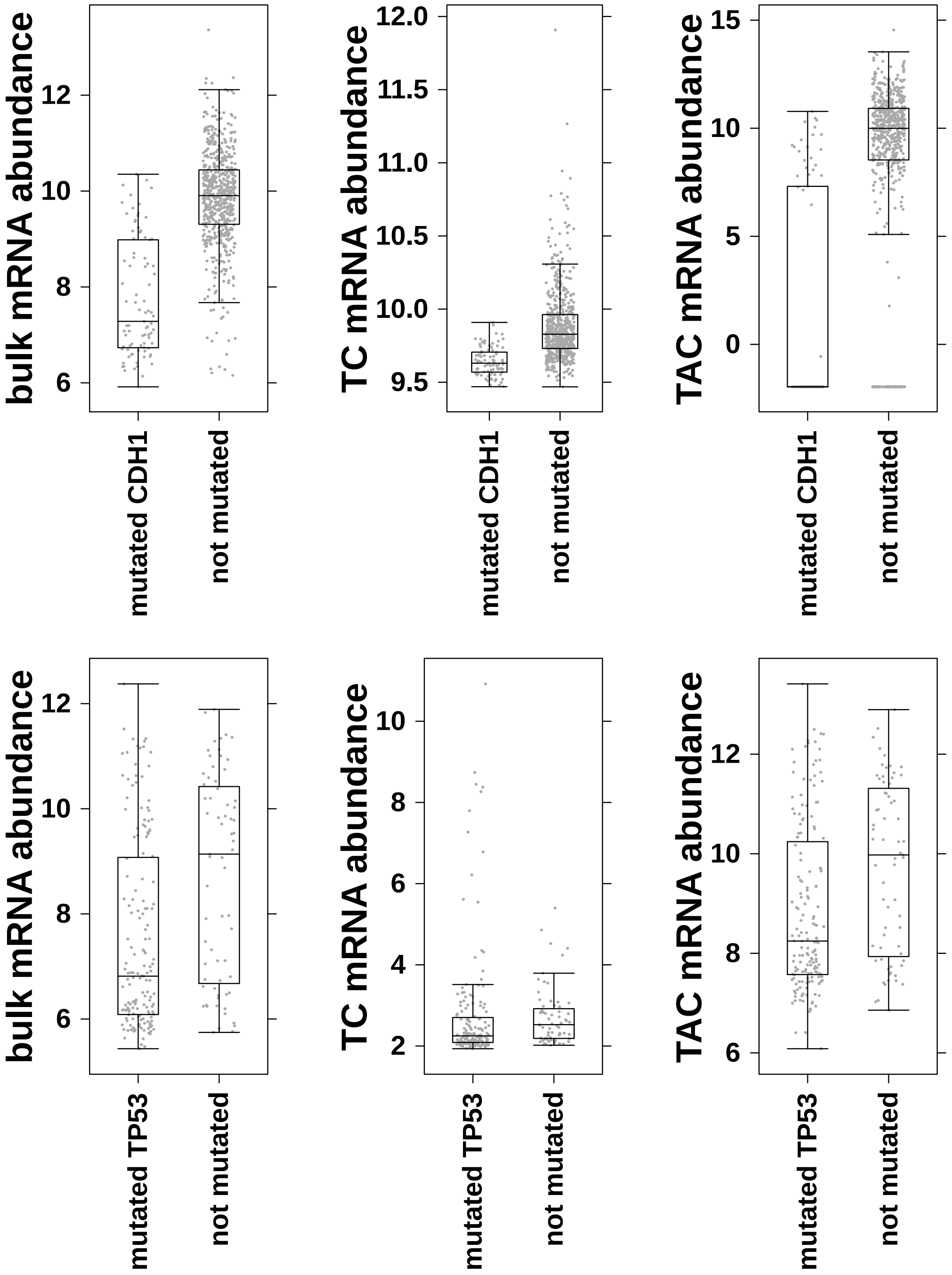
bulk mRNA abundance
TC mRNA abundance
TAC mRNA abundance
mutated CDH1
not mutated
mutated TP53

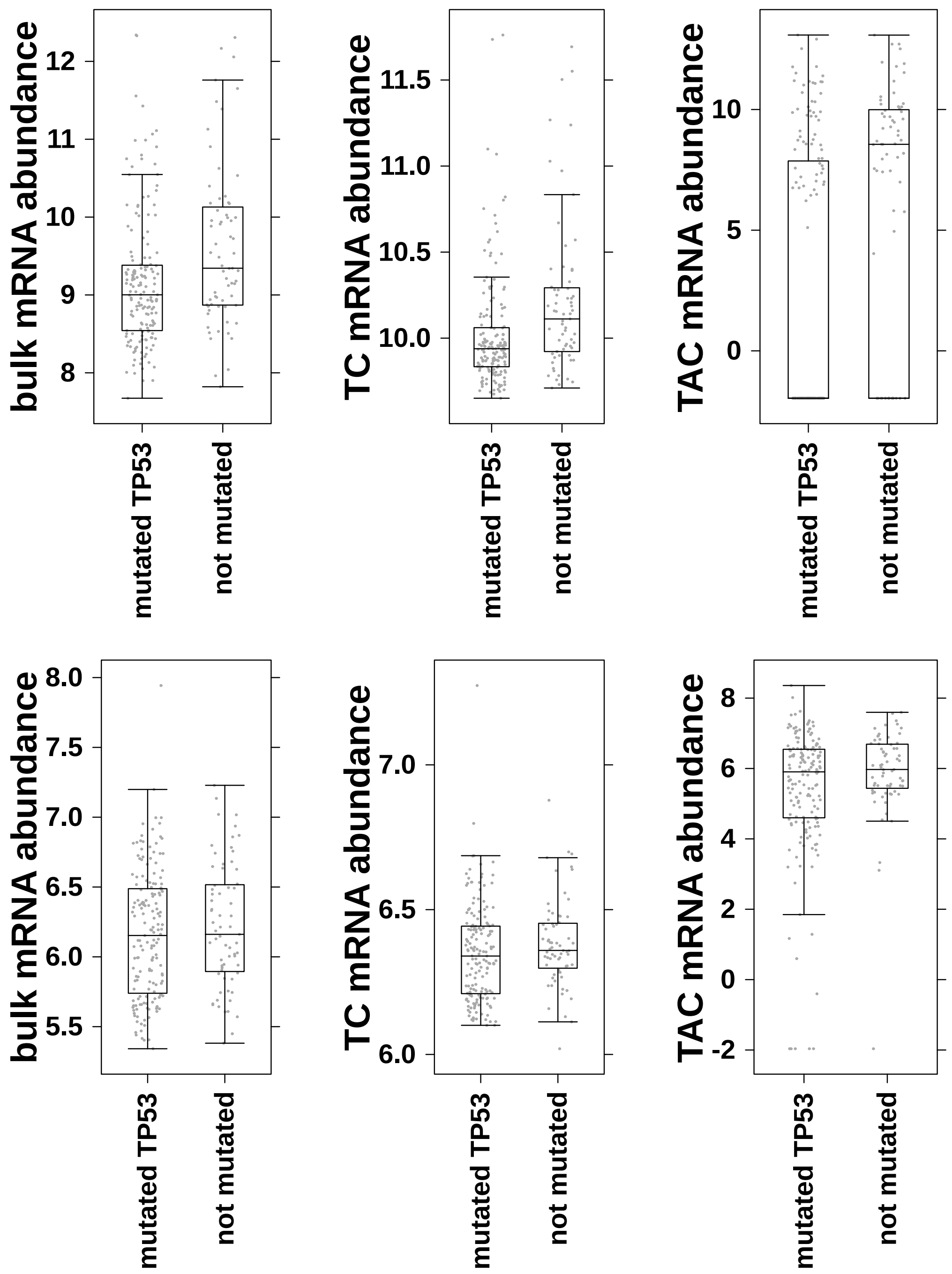

33 Supplmentary Figure 12D - Overlap between biological processes associated *TP53* mutations

Set up the environment

```
library(BoutrosLab.plotting.general);
library(yaml);
library(limma);
dataset.name <- 'Metabric';
```

Yaml file name

```
yaml.file <- paste0(dataset.name,'.yaml');
```

Read yaml with file information

```
dataset.files <- yaml.load_file(yaml.file);
```

Load clinical annotation

```
patient.anno <- read.table(dataset.files$clinical.annotation.file,sep='\t',header=TRUE);
```

Load mRNA abundance profiles

```
profile.types <- c('bulk','tc','tac');
rna.data.matrix <- list();
for(profile.type in profile.types) {
  rna.data.matrix[[profile.type]] <- read.table(
    dataset.files[[paste0(profile.type,'.mrna.abundance.file')]],
    header=TRUE,
    sep='\t'
    );
  }
```

Load SNVs

```
snvs <- read.table(dataset.files$snv.file,header=TRUE);
```

Make sure all the data is ordered the same

```
overlapping.patients <- intersect(colnames(rna.data.matrix$bulk),colnames(snvs));
for(profile.type in profile.types) {
  rna.data.matrix[[profile.type]] <- rna.data.matrix[[profile.type]][,overlapping.patients];
  }
snvs <- snvs[,overlapping.patients];
patient.anno <- patient.anno[overlapping.patients,];
```

Run limma to determine differential mRNA abundance between mutated genes

```
subtype.limma.results <- list();
for(subtype in c('Basal','Her2','LumA','LumB')) {
  genes.ordered.by.recurrence <- names(which(rowSums(snvs[
    order(rowSums(snvs)),rownames(patient.anno)[patient.anno$Pam50Subtype == subtype]
    ]) > 50));
  genes.ordered.by.recurrence <- genes.ordered.by.recurrence[rev(order(rowSums(snvs[
    genes.ordered.by.recurrence,rownames(patient.anno)[patient.anno$Pam50Subtype == subtype]
    ])))];
  tumour.fold.changes <- matrix(
    NA,
    ncol=length(genes.ordered.by.recurrence),nrow=nrow(rna.data.matrix[[1]])
    );
  colnames(tumour.fold.changes) <- genes.ordered.by.recurrence;
```

```
  tac.fold.changes <- tumour.fold.changes;
  sig.genes.data <- list();
  for(gene.snv in genes.ordered.by.recurrence) {
    targets <- rep(NA,sum(patient.anno$Pam50Subtype == subtype));
    targets[snvs[gene.snv,rownames(patient.anno)[patient.anno$Pam50Subtype == subtype]] == 1] <- 'mut
    targets[snvs[gene.snv,rownames(patient.anno)[patient.anno$Pam50Subtype == subtype]] == 0] <- 'not
    gtargets <- unique(targets);
    m <- match(targets,gtargets);
    design <- model.matrix(~ -1 + factor(m));
    colnames(design) <- gtargets;
    contrast.matrix <- makeContrasts(not-mutated, levels=design);
    limma.results <- list();
    for(profile in profile.types) {
      mrna <- rna.data.matrix[[profile]][,rownames(patient.anno)[patient.anno$Pam50Subtype == subtype
      rownames(mrna) <- 1:nrow(mrna);
      mrna <- as.matrix(mrna);
      fit <- lmFit(mrna, design);
      fit2 <- contrasts.fit(fit, contrast.matrix);
      fit3 <- eBayes(fit2);
      top1 <- topTable(fit3,coef=1,number=nrow(fit3));
      top1$GeneID <- rownames(rna.data.matrix[[profile]])[as.numeric(rownames(top1))];
      limma.results[[profile]] <- top1[order(top1$GeneID),];
      }
    sig.genes.data[[gene.snv]] <- cbind(
      limma.results$bulk[,c('GeneID','logFC','adj.P.Val')],
      limma.results$tc[,c('logFC','adj.P.Val')],
      limma.results$tac[,c('logFC','adj.P.Val')]
      )[limma.results$bulk$adj.P.Val < 0.05 | limma.results$tc$adj.P.Val < 0.05 | limma.results$tac$a
    colnames(sig.genes.data[[gene.snv]]) <- c(
      'GeneID','bulk.logFC','bulk.adj.P.Val',
      'tc.logFC','tc.adj.P.Val',
      'tac.logFC','tac.adj.P.Val'
      );
    }
  subtype.limma.results[[subtype]] <- sig.genes.data;
  }
```

Load gene symbols

```
mrna.conversion.info <- read.table(dataset.files$gene.symbol.file,header=TRUE,sep='\t');
```

Copy the gene symbols to the web pathway tool gprofiler (https://biit.cs.ut.ee/gprofiler/) and the save the results for creating the venn diagram next

```
gene.id <- subtype.limma.results$Her2$TP53$GeneID[subtype.limma.results$Her2$TP53$tac.adj.P.Val < 0.0
gene.id <- sub('_at','',gene.id);
paste(
  sort(unique(as.character(mrna.conversion.info$GeneSymbol[match(gene.id, mrna.conversion.info$Entrez
  collapse=' '
  );
```

```
[1] "AARSD1 AATK ABAT ABCA11P ABCA3 ABCA6 ABCA8 ABCA9 ABCB9 ABCG1 ABHD1 ABHD14A ABHD15 ABHD16A ABHD3
ERP44 ERVMER34-1 ERVV-1 ESPN ESR1 ETNK1 ETV3L ETV5 EVA1C EVL EVX1 EXOC2 EXOC3L4 EXOC6 EXOSC5 EXTL2 EZ
MRC2 MRE11A MRGPRD MRGPRF MRGPRX2 MRPL21 MRPL24 MRPL27 MRPL43 MRPS12 MRPS36 MS4A15 MS4A8 MST1 MT1M MT
STYX SUCLG2 SUDS3 SULF1 SULT2B1 SUN5 SUPT4H1 SUPT6H SURF2 SUSD3 SUV39H1 SVEP1 SYN2 SYNDIG1L SYNE4 SYN

gene.id <- subtype.limma.results$Basal$TP53$GeneID[subtype.limma.results$Basal$TP53$tac.adj.P.Val < 0
gene.id <- sub('_at','',gene.id);
paste(
  sort(unique(as.character(mrna.conversion.info$GeneSymbol[match(gene.id, mrna.conversion.info$Entrez
  collapse=' '
  );

[1] "FRMD6-AS1 ITIH5 JAM3 MARCH2 RNF39 TMEM30B"

gene.id <- subtype.limma.results$LumB$TP53$GeneID[subtype.limma.results$LumB$TP53$tac.adj.P.Val < 0.0
gene.id <- sub('_at','',gene.id);
paste(
  sort(unique(as.character(mrna.conversion.info$GeneSymbol[match(gene.id, mrna.conversion.info$Entrez
  collapse=' '
  );

[1] "A2ML1 AARS AARSD1 ABAT ABCA11P ABCA2 ABCA3 ABCA5 ABCC8 ABHD14A ABHD14B ABHD15 ABHD8 ABI3BP ABL1
ERCC1 ERGIC1 ERLEC1 ERP44 ESD ESPL1 ESR1 ESRRG ETNK1 EVA1B EVI5L EVPL EXD2 EXD3 EXO1 EXOC6B EXOC7 EXO
NEDD8 NEIL1 NEIL2 NEK11 NELFCD NELL2 NEU1 NFIB NFIL3 NFS1 NFU1 NHLRC3 NICN1 NIPA1 NISCH NKAIN1 NKG7 N
TMC4 TMCC1 TMCO1 TMCO3 TMED10 TMEM101 TMEM115 TMEM132A TMEM140 TMEM144 TMEM154 TMEM175 TMEM177 TMEM20

gene.id <- subtype.limma.results$LumA$TP53$GeneID[subtype.limma.results$LumA$TP53$tac.adj.P.Val < 0.0
gene.id <- sub('_at','',gene.id);
paste(
  sort(unique(as.character(mrna.conversion.info$GeneSymbol[match(gene.id, mrna.conversion.info$Entrez
  collapse=' '
  );

[1] "AARS AARSD1 AATF ABAT ABCC2 ABHD1 ABHD14A ABHD14B ABHD15 ABR ACAA1 ACAD10 ACADSB ACADVL ACE2 ACE
KRT81 KRT86 KRTCAP3 KTN1-AS1 LAG3 LAGE3 LAIR2 LAMB2 LAMP2 LAMTOR4 LAPTM4B LASS2 LBP LCMT2 LDLRAD3 LEP
ZBTB47 ZC3H10 ZC3H14 ZC3H5 ZCCHC17 ZCRB1 ZCWPW1 ZCWPW2 ZDHHC1 ZDHHC20 ZFP3 ZFX ZG16B ZHX1 ZIC2 ZMYM2
```

Read in gprofiler results

```
gprofiler.results <- list();
for(subtype in c('Her2','LumA','LumB')) {
  gprofiler.results[[subtype]] <- read.table(
    paste0(dataset.files$parent.output.dir,'6-snvs/gprofiler_results_',subtype,'.txt'),
    header=FALSE,
    sep='\t'
    );
  gprofiler.results[[subtype]] <- gprofiler.results[[subtype]][gprofiler.results[[subtype]]$V10 == 'B
  }
```

Create the venn diagram showing overlap between go terms Set up the environment

```
library(VennDiagram);
venn.diagram(
  x=list(
    'HER2-enriched'=as.character(gprofiler.results[['Her2']]$V12),
    '    Luminal A'=as.character(gprofiler.results[['LumA']]$V12),
    'Luminal B'=as.character(gprofiler.results[['LumB']]$V12)
    ),
  filename = './sfigure12d.pdf',
  );
```

```
[1] 1
```

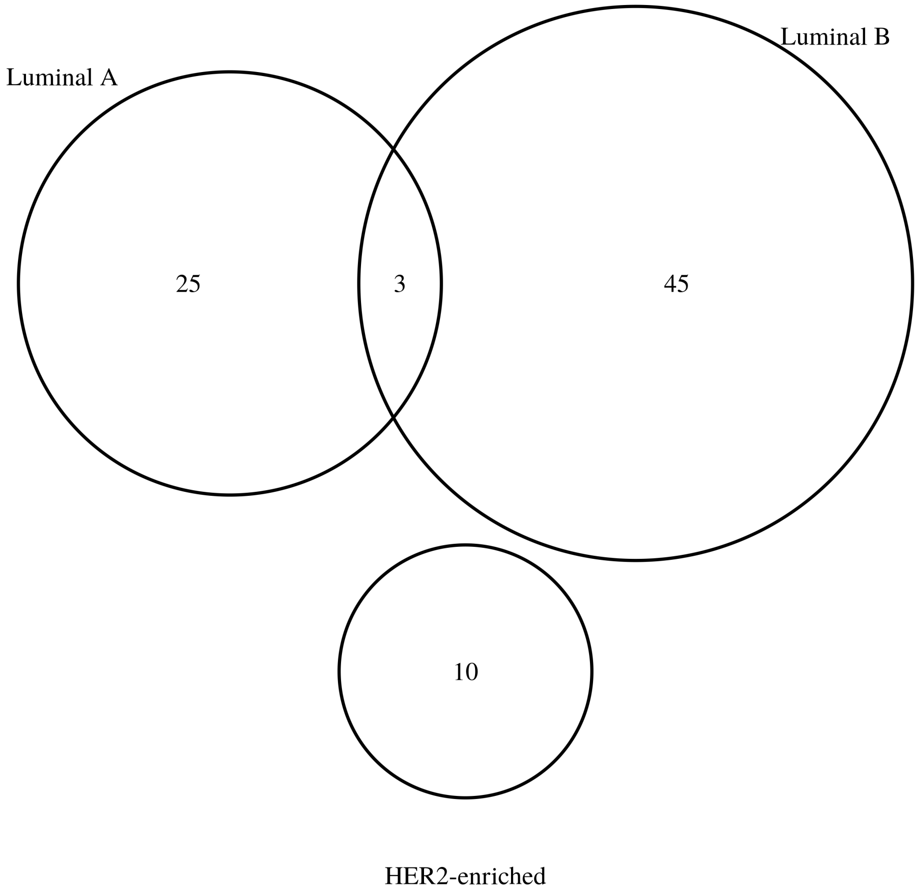

Supplement: Supplementary file 11 — Supplementary Code 1 [file 41467_2019_10929_MOESM11_ESM.zip › NCOMMS-18-06488B Supplementary Code 1.pdf]
