# Supplementary material for: Landscape of transcriptomic interactions between breast cancer and its microenvironment
Source: Nat Commun. 2019 Jul 15;10:3116. doi: 10.1038/s41467-019-10929-z (PMC6629667; doi:10.1038/s41467-019-10929-z)
Supplement: Supplementary file 12 — Reporting Summary [file 41467_2019_10929_MOESM12_ESM.pdf]

nature research

Corresponding author(s): Natalie Fox

Last updated by author(s): Apr 22, 2019

Reporting Summary

Nature Research wishes to improve the reproducibility of the work that we publish. This form provides structure for consistency and transparency in reporting. For further information on Nature Research policies, see Authors & Referees and the Editorial Policy Checklist.

Statistics

For all statistical analyses, confirm that the following items are present in the figure legend, table legend, main text, or Methods section.

n/a	Confirmed	
☐	☒	The exact sample size (*n*) for each experimental group/condition, given as a discrete number and unit of measurement
☐	☒	A statement on whether measurements were taken from distinct samples or whether the same sample was measured repeatedly
☐	☒	The statistical test(s) used AND whether they are one- or two-sided<br>*Only common tests should be described solely by name; describe more complex techniques in the Methods section.*
☐	☒	A description of all covariates tested
☐	☒	A description of any assumptions or corrections, such as tests of normality and adjustment for multiple comparisons
☐	☒	A full description of the statistical parameters including central tendency (e.g. means) or other basic estimates (e.g. regression coefficient) AND variation (e.g. standard deviation) or associated estimates of uncertainty (e.g. confidence intervals)
☐	☒	For null hypothesis testing, the test statistic (e.g. *F*, *t*, *r*) with confidence intervals, effect sizes, degrees of freedom and *P* value noted<br>*Give P values as exact values whenever suitable.*
☒	☐	For Bayesian analysis, information on the choice of priors and Markov chain Monte Carlo settings
☐	☒	For hierarchical and complex designs, identification of the appropriate level for tests and full reporting of outcomes
☐	☒	Estimates of effect sizes (e.g. Cohen's *d*, Pearson's *r*), indicating how they were calculated

Our web collection on statistics for biologists contains articles on many of the points above.

Software and code

Policy information about availability of computer code

Data collection	No software with used for data collection
Data analysis	ISOpure and ISOpureR were used to create TC and TAC profiles. g:Profiler (version r1732_e89_eg36) was used for functional profiling. Within the R statistical environment: limma (v 3.32.10), randomForest (v4.6-12), pROC (v1.8) and survival (v2.38-3) packages were used along with custom code.

For manuscripts utilizing custom algorithms or software that are central to the research but not yet described in published literature, software must be made available to editors/reviewers. We strongly encourage code deposition in a community repository (e.g. GitHub). See the Nature Research guidelines for submitting code & software for further information.

Data

Policy information about availability of data

All manuscripts must include a data availability statement. This statement should provide the following information, where applicable:

- Accession codes, unique identifiers, or web links for publicly available datasets
- A list of figures that have associated raw data
- A description of any restrictions on data availability

The data this analysis is built upon is from the Curtis et al.22 (doi: 10.1038/nature10983) and Pereira et al.31 (doi: 10.1038/ncomms11479) for the METABRIC data and as part of release 2016-01-28 from the Broad GDAC Firehose (https://gdac.broadinstitute.org/) for the TCGA data.

Field-specific reporting

Please select the one below that is the best fit for your research. If you are not sure, read the appropriate sections before making your selection.

☒ Life sciences ☐ Behavioural & social sciences ☐ Ecological, evolutionary & environmental sciences

For a reference copy of the document with all sections, see nature.com/documents/nr-reporting-summary-flat.pdf

Life sciences study design

All studies must disclose on these points even when the disclosure is negative.

Sample size	We are working with previously published data so sample size was limited to available published data. Using the METABRIC (Curtis et al.) and breast cancer TCGA (realease 2016-01-28), all available mRNA and SNV profiles were used. Power calculations were not performed but these samples sizes and smaller samples sizes have previously been used for similar analyses.
Data exclusions	Patients samples with undefined and normal-like breast cancer were excluded to focus on the better characterized PAM50 subtypes.
Replication	TC and TAC mRNA abundance profiles were analyzed in two independent datasets (1 - METABRIC and 2 - TCGA) . There was general concordance between the two datasets.
Randomization	For cross validation, patients were randomly assigned to folds for each subtype.
Blinding	Blinding was not used because this is exploratory analysis so it's not relevant.

Reporting for specific materials, systems and methods

We require information from authors about some types of materials, experimental systems and methods used in many studies. Here, indicate whether each material, system or method listed is relevant to your study. If you are not sure if a list item applies to your research, read the appropriate section before selecting a response.

Materials & experimental systems

n/a	Involved in the study	
☒	☐	Antibodies
☒	☐	Eukaryotic cell lines
☒	☐	Palaeontology
☒	☐	Animals and other organisms
☒	☐	Human research participants
☒	☐	Clinical data

Methods

n/a	Involved in the study	
☒	☐	ChIP-seq
☒	☐	Flow cytometry
☒	☐	MRI-based neuroimaging
